# A Non-Coding Fc Gamma Receptor Cis-Regulatory Variant within the 1q23 Gene Cluster Is Associated with *Plasmodium falciparum* Infection in Children Residing in Burkina Faso

**DOI:** 10.3390/ijms242115711

**Published:** 2023-10-28

**Authors:** Jules Cretin, Mathieu Adjemout, Christelle Dieppois, Frederic Gallardo, Magali Torres, Zachary Merard, Serge Aimé Sawadogo, Christophe Picard, Pascal Rihet, Pascale Paul

**Affiliations:** 1 INSERM 1090, TAGC Theories and Approaches of Genomic Complexity, Campus de Luminy, Aix Marseille University, 13288 Marseille, Francemathieu.adjemout@univ-amu.fr (M.A.); christelle.dieppois@outlook.fr (C.D.); frederic.gallardo@inserm.fr (F.G.); magali.torres@univ-amu.fr (M.T.); 2 Institut MarMaRa, 13288 Marseille, France; 3 ADES UMR, Aix Marseille University, 13288 Marseille, Francechristophe.picard@efs.sante.fr (C.P.); 4 Unité de Formation en Sciences de la Santé (UFR/SDS), Université Joseph KI-ZERBO, Ouagadougou 03 BP 7021, Burkina Faso; serge.sawadogo@ujkz.bf; 5 Centre PrïmO-Nelson Mandela, 84 rue Sao Tomé et Principe, Ouagadougou 09 BP 706, Burkina Faso; 6 Immunogenetics Laboratory, Etablissement Français du Sang PACA-Corse, 13001 Marseille, France

**Keywords:** Fc-gamma receptors/FcγRs, regulatory variants, malaria, *Plasmodium falciparum* parasitemia, Fc-gamma receptor (FCGR) gene polymorphism, FCGR2A gene polymorphism, FCGR2B gene polymorphism, Burkina Faso, FCGR ex-pression quantitative trait loci (eQTL)

## Abstract

Antibodies play a crucial role in activating protective immunity against malaria by interacting with Fc-gamma receptors (FcγRs). Genetic variations in genes encoding FcγRs can affect immune cell responses to the parasite. In this study, our aim was to investigate whether non-coding variants that regulate FcγR expression could influence the prevalence of *Plasmodium falciparum* infection. Through bioinformatics approaches, we selected expression quantitative trait loci (eQTL) for *FCGR2A, FCGR2B, FCGR2C, FCGR3A*, and *FCGR3B* genes encoding FcγRs (*FCGR*), in whole blood. We prioritized two regulatory variants, rs2099684 and rs1771575, located in open genomic regions. These variants were identified using RegVar, ImmuNexUT, and transcription factor annotations specific to immune cells. In addition to these, we genotyped the coding variants *FCGR2A*/rs1801274 and *FCGR2B*/rs1050501 in 234 individuals from a malaria-endemic area in Burkina Faso. We conducted age and family-based analyses to evaluate associations with the prevalence of malarial infection in both children and adults. The analysis revealed that the regulatory rs1771575-CC genotype was predicted to influence *FCGR2B/FCGR2C/FCGR3A* transcripts in immune cells and was the sole variant associated with a higher prevalence of malarial infection in children. In conclusion, this study identifies the rs1771575 cis-regulatory variant affecting several FcγRs in myeloid and neutrophil cells and associates it with the inter-individual capacity of children living in Burkina Faso to control malarial infection.

## 1. Introduction

Malaria caused by *Plasmodium falciparum* (*Pf*) remains a significant global health burden, particularly for children living in endemic regions [1]. The disease’s severity and outcomes are influenced by a complex interplay of environmental and genetic factors. Repeated exposures of children to *Pf* and other pathogens allow individuals in endemic African regions to gradually develop immunity against clinical malaria. The high mortality rate associated with malarial infection has exerted strong selective pressure, shaping the human genome in endemic regions throughout recent history [2]. Several studies have documented the prevalence of *Pf* infection in children from diverse geographical regions. These investigations have highlighted the disproportionate burden faced by children living in sub-Saharan Africa and Southeast Asia. While genome-wide association [3,4] and prospective transcriptomics studies [5,6] have explored host genetic susceptibility to control parasitemia and fever following *Pf* infection, research on the functional association is still needed. Monocytes and neutrophils play a crucial role in controlling parasite burden and protecting the host from malaria [7,8,9,10,11,12,13,14], and a low monocyte to neutrophil ratio has been associated with an increased risk of developing complicated malaria [15]. As demonstrated via passive transfer experiments with immunoglobulin G (IgG) in humans, antibodies drive immune responses that enable the control of the parasite via direct agglutination of the parasite or interaction with a wide variety of Fc-gamma receptors (FcγRs) expressed on immune cells [16]. FcγR recognition of the Fc-domains of antibodies bound to pathogenic antigens leads to immune activation of neutrophils, monocytes, and natural killer (NK) cells, which participate in the elimination of the malaria parasite via antibody-dependent cell phagocytosis (ADCP) and cytotoxicity (ADCC), antibody-dependent cellular inhibition (ADCI), or respiratory burst [10,17,18,19,20]. The *FCGR* genes are clustered on chromosome 1q23 and encode FcγR-activating receptors (FcγRI/CD64, FcγRIIA/CD2a, FcγRIIC/CD32c, FcγRIIIA/CD16a, and FcγRIIIB/CD16b) that bear an immunoreceptor tyrosine-based activation motif (ITAM) [21]. Activating FcγRIIA/CD32a and FcγRIIIB/CD16b can facilitate early activation of neutrophil-mediated opsonic phagocytosis of sporozoites or FcγRIIA/CD32a monocyte-driven *P. falciparum* elimination of infected erythrocytes [22]. The *FCGR2B* gene is highly homologous to the *FCGR2A/FCGR2C* genes but encodes a FcγRIIB (CD32b) inhibitory isoform with an intracellular immunoreceptor tyrosine-based inhibitory motif (ITIM), expressed at high levels on B cells and basophils and at low levels on a fraction of circulating monocytes and neutrophils [23]. Several polymorphisms have been detected in the genes encoding these activating receptors and are further associated with host susceptibility or resistance to malaria in different populations endemic for malaria. The rs1801274 coding mutation in the *FCGR2A* gene, which encodes the FcγRIIA/CD32a for the Fc fragment of IgG, has been implicated in various diseases, including malaria [22,24,25,26,27]. The C allelic variant of rs1801274 encodes the arginine allele(R), while the T allele encodes the variant histidine (H) with higher binding affinity for IgG2 and IgG3 than the (R) isoform of the FcγRIIA/CD32a activating isoform. A study involving over 1800 individuals in India found that rs1801274-TT homozygotes were significantly associated with protection from disease manifestation, with a stronger association observed in the malaria non-endemic region [28]. However, conflicting results have been reported regarding the impact of this polymorphism on malaria susceptibility [22,24,25,26,29,30,31,32,33,34].

The complexity of interindividual variation in FcγR-mediated phagocytic activity is enhanced by the co-expression of the activating receptor alongside the inhibitory receptor FcγRIIB/CD32b in monocytes. This interplay functions as a unique counterpoint to activating *FCGR*; FcγRIIB signaling differs from that of activating FcγR due to the presence of an intracellular immunoreceptor tyrosine-based inhibition motif (ITIM). Upon phosphorylation, this motif acts as a checkpoint inhibitory pathway, reversing immune activation initiated by ITAM-bearing Fc receptors. Additionally, FcγRIIB has been shown to participate in antibody-mediated target cell depletion through ITIM-independent mechanisms [35,36]. FcγRIIB/CD32b expression on B lymphocytes is also involved in the BCR-dependent differentiation of B lymphocytes into antibody-producing cells, thus potentially impacting IgG production. The rs1050501 mutation refers to a single nucleotide change located in exon 5 of the *FCGR2B* gene, resulting in an amino acid substitution from isoleucine (I) to threonine (T) at position 232 (I232T) in the transmembrane domain of the protein. This mutation has been associated with various infectious or autoimmune disease traits [31,37,38]. The frequency of the homozygous FcγRIIBT232 variant varies considerably among different ethnic populations, being higher in Africans (8–11%) or Southeast Asians (5–7%) than in Caucasians (1%) [39]. These differences in allelic frequencies suggest recent positive selection of this protective mutation in endemic areas, potentially accounting for varying susceptibility to infectious or autoimmune diseases in different geographic regions [31,40]. The FcγRIIB-232TT genotype has notably been associated with an increased risk of vertical acquisition of HIV-1 [41], but it has also shown a strong protective effect against severe malaria in Kenya. However, the underlying mechanisms involving *FCGR2A* and *FCGRB* coding mutations remain complex and require further investigation. The I232T substitution reduces the in situ two-dimensional binding affinities and association rates of FcγRIIB with its ligands, IgG1, IgG2, and IgG3, by three to four folds [42]. This protective effect of the rs1050501 T allelic variant against malaria infection has been associated with a lower capacity to impair FcγRIIA-mediated antibody response, which affects individuals’ ability to eliminate parasites through monocyte-driven FcγRIIA phagocytosis of *P. falciparum*-infected human erythrocytes [31]. However, other studies have shown no association between this polymorphism and malaria susceptibility.

The relationship between functional *FCGR* variants and malaria immunization and severity has been extensively explored [29]. However, their correlation with parasitemia has received less attention. Additionally, while certain coding FcγR variants have been associated with malaria susceptibility, there is a lack of research focused on investigating the impact of FcγR regulatory variants on immune activation potential. Causality in associations with complex malaria phenotypes poses challenges, particularly due to the fact that approximately 90% of GWAS variants that regulate cell-specific gene expression are located in non-coding regions, which have been insufficiently studied. Various studies have suggested that non-coding trait-associated variants are enriched for expression quantitative trait loci (eQTLs), which can influence target gene expression. Consequently, these eQTLs may impact the FcγR-dependent innate cell immune activation threshold, affecting the host’s ability to eliminate parasites via the antibody-dependent activation of innate immune cells.

Given the limited understanding of how regulatory genetic variants influence immune-related traits and their association with the progressive acquisition of protection, our study aims to investigate whether *FCGR* eQTLs identified within the *FCGR* gene cluster can be linked to the individual ability of children to control *P. falciparum* infection.

## 2. Results

### 2.1. Prioritization and Annotation of Putative Regulatory SNPs in the Non-Coding FCGR Gene Cluster

**Figure 1:**
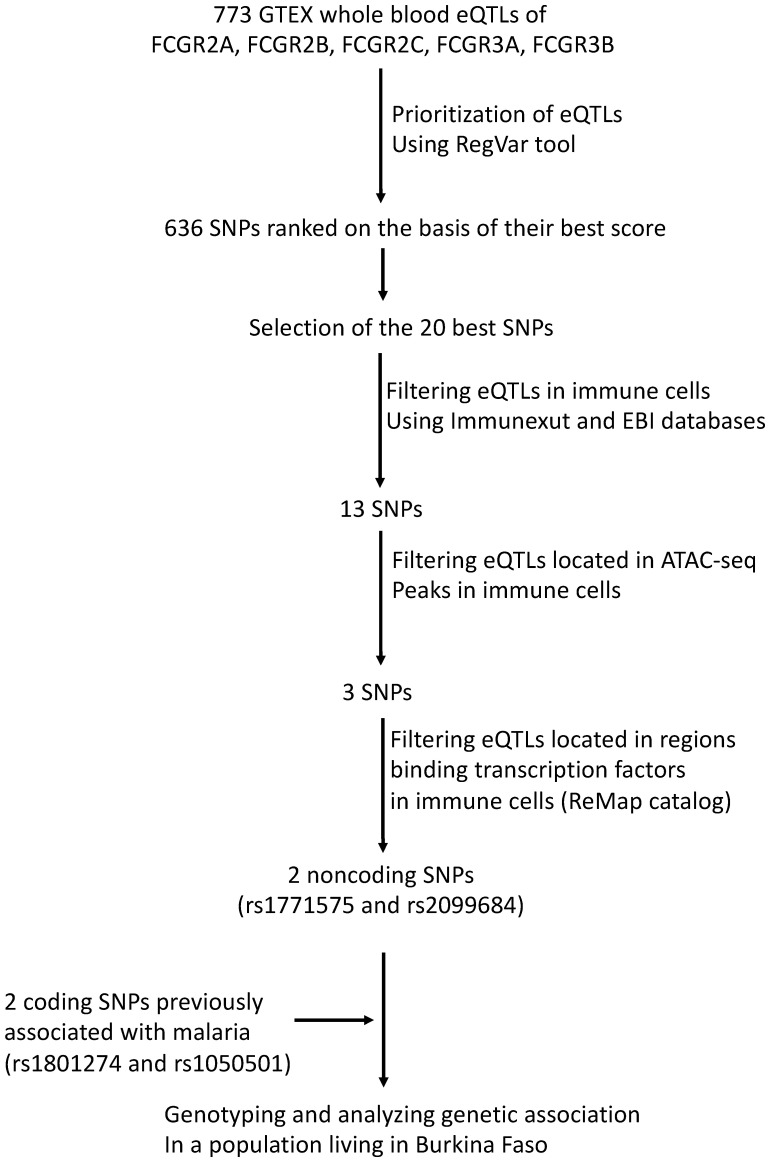
Work flow used to prioritize regulatory variants. A total of 773 SNPs carrying an eQTL annotation in whole blood for *FCGR2A*, *FCGR2B*, *FCGR2C*, *FCGR3A*, and *FCGR3B* genes were searched by querying the current release of GTEX (V8) database. The RegVar tool further allowed gene-specific annotation and tissue-specific annotation to prioritize 636 SNPs. The 20 SNPs that displayed the best scores were selected using RegVar; eQTL annotations were retained to select variants that have annotation in 2 independent immune cell eQTL databases, ImmuNexUT and ebi eQTL catalog. The position of eQTL candidates was cross-referenced with the position of ATAC-seq regions in immune cells and positioning in regions that exhibit transcription factor binding activity in immune cells, using the ReMap catalog. ReMap data were filtered on primary NK, T and B lymphocytes, monocytes, macrophages, neutrophils, peripheral blood cells, and monocytic and lymphocytic cell lines to allow selection of 2 FCGR regulatory variants further used for association studies.

The SNP selection procedure, summarized in Figure 1, involved several steps. Initially, we identified 773 SNPs with eQTL annotations for *FCGR2A*, *FCGR2B*, *FCGR2C, FCGR3A*, and *FCGR3B* genes in the GTEX version 8 database [43]. Subsequently, we prioritized 636 SNPs using the RegVar tool [44], which is based on the GTEX version 7 database (Supplemental Table S1). Among these, we further annotated the 20 SNPs with the best RegVar scores, which were higher than the 0.8 threshold (Supplemental Table S2). Out of the selected SNPs, 13 were found to be eQTLs in immune cells, as indicated by both the ImmuNexUT database [45] and the ebi eQTL catalog [46]. Among these, only 2 intergenic eQTLs (rs1771575 and rs2099684) were located in open genomic regions binding transcription factors in immune cells, as shown by the ATAC-seq Database [47] and the ReMap ChIP-seq catalog [48]. These immune cells include neutrophils, monocytes, macrophages, dendritic, and NK cells, as stated in Table 1. Additionally, we examined two coding SNPs (rs1801274 and rs1050501) previously associated with malaria phenotypes and provided their structural annotations in Table 1. While rs1050501 was not identified as an expression quantitative trait locus (eQTL), the coding variant rs1801274 was found to be an eQTL for *FCGR* genes in whole blood (Supplemental Table S2). We also cross-referenced the positions of eQTL candidates with enhancer regions identified in the ENCODE database (Table 1) [49]. However, this eQTL was not located within an open genomic region in immune cells (Table 1 and Figure 1).

In contrast, the rs1771575 variant was situated in open genomic regions in various immune cells, including monocytes, macrophages, neutrophils, and dendritic cells (Table 1). It was also identified as an eQTL for *FCGR2B* and *FCGR3A*, as well as *FCGR3B* target genes in neutrophils, using data from the ImmuNexUT database (Table 2). However, its effects on gene expression were found to be less pronounced in naive monocytes when eQTL data from the EBI catalog were utilized. Individuals with the rs1771575-TT genotype exhibited significantly enhanced gene expression of *FCGR3A* and *FCGR2B* in neutrophils compared to those with the CC/CT genotype. However, this variant did not show any significant impact on *FCGR3A* expression in NK cells or *FCGR2B* gene expression in B cells. Furthermore, the rs1771575 mutation did not have a substantial effect on *FCGR3B* or *FCGR2A* transcripts, which encode activating receptors, in neutrophils. Similarly, the rs2099684 variant was found within open regions in B lymphocytes, dendritic cells, monocytes, and NK cells (Table 1), and it was identified as an eQTL for *FCGR2A, FCGR2B, FCGR2C*, and *FCGR3B* in neutrophils, monocytes, macrophages (Table 2), and NK cells. The rs2099684 GG genotype was highly associated with enhanced expression of *FCGR2A* transcripts in neutrophils and monocytes and was also significantly associated with enhanced *FCGR2C* and *FCGR3B* transcript expression in NK cells, according to the ImmuNexUT database.

**Figure 2:**
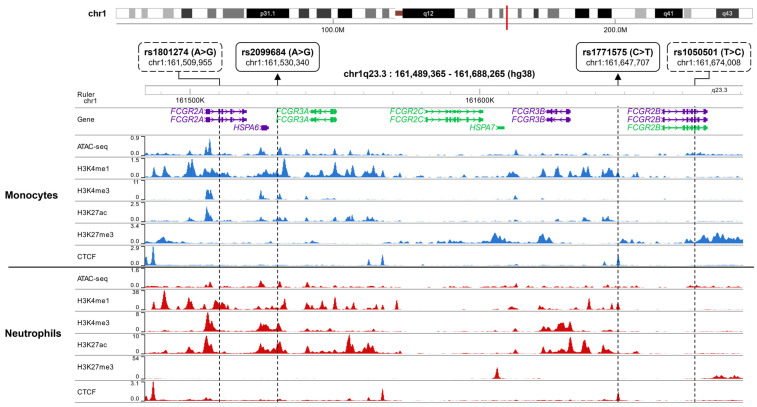
Epigenetic Landscape of the FCGR Locus in Monocytes and Neutrophils. The presence of epigenetic marks (ATAC-seq, H3K4me1, H3K4me3, H3K27ac, and H3K27me3) and CTCF transcription factor binding profiles for rs1771575, rs2099684, rs1801274, and rs1050501 in blood monocytes and neutrophils were visualized using the CistromeDB database in conjunction with the WashU epigenome browser. The genomic location of the FCGR locus on chromosome 1q23 is depicted as a red line. For both monocytes (**upper** panel) and neutrophils (**lower** panel), the following data is displayed: ATAC-Seq peaks, CTCF (CCCTC-binding factor) binding sites, ChIP-seq peaks, and Histone modifications associated with active regulatory regions: H3K4me1, H3K4me3, H3K27ac, and H3K27me3. The variant rs2099684 is situated within an active enhancer, whereas rs1771575 is found within both an active enhancer and a CTCF binding site. Additionally, the known coding variants rs1801274 and rs1050501 are also represented.

To gain deeper insights, we further investigated the presence of epigenetic markers (ATAC-seq, H3K4me1, H3K4me3, H3K27ac, and H3K27me3) and the transcription factor binding profile of CCCTC-binding factor (CTCF) for rs1771575, rs2099684, rs1801274, and rs1050501 in blood monocytes and neutrophils (Figure 2) as well as in B and NK lymphocytes (Supplemental Figure S1). These cell-specific profiles were visualized using the CistromeDB database with the WashU epigenome browser [37].

Interestingly, rs1771575 coincided with a peak of CTCF binding in monocytes, neutrophils, and NK cells, while rs2099684 was situated within peaks of H3K27ac, particularly in neutrophils. In contrast, rs1801274 and rs1050501 displayed weak associations with epigenomic marks linked to gene expression regulation.

Furthermore, we conducted an in-depth analysis to assess the impact of the rs1771575 and rs2099684 mutations on transcription factor binding. The RSAT analyses revealed that rs1771575 specifically influences the binding of several transcription factors, including the Nuclear Receptor Subfamily 3, Group C, Member 1 (NRC31), and the zinc finger protein 24 (ZNF24) (Supplemental Table S4).

**Figure 3:**
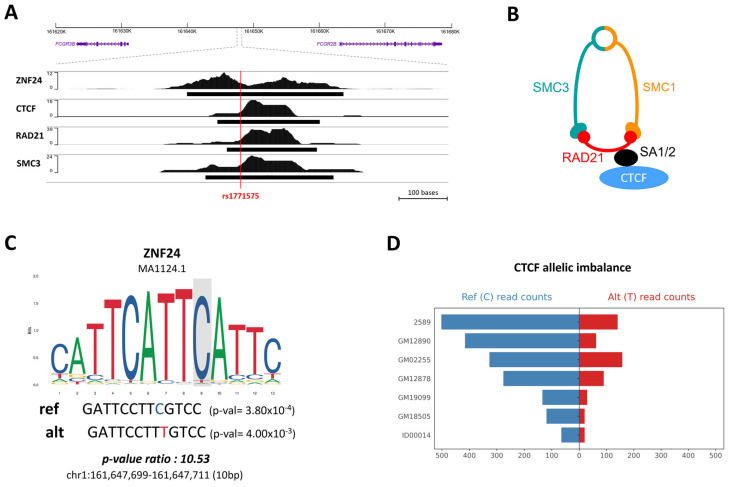
Alteration of transcription factor binding sites at position rs1771575. (**A**) ChIP-seq experiments in GM12878 (B-LCL Cell Line). ChIP-seq experiments were conducted in the GM12878 (B-LCL) cell line, revealing that ZNF24, CTCF, and cohesin subunits RAD21 and SMC3 bind to rs1771575. (**B**) Schematic interaction between CTCF and the cohesin complex. A schematic representation demonstrates the interaction between CTCF and the cohesin complex. (**C**) Position weight matrix of ZNF24 binding. The position weight matrix analysis of ZNF24 binding indicates a preference for binding to chromatin in the presence of the reference allele (**C**) of rs1771575. *p*-values for each putative site and their ratio (pval_ratio = worst_pval/best_pval) were calculated using the RSAT ‘scan variations’ tool. JASPAR matrix ID and genomic coordinates (GRCh38) are also provided. (**D**) CTCF binding ChIP-seq analysis. Analysis of ChIP-seq data for CTCF binding using read counts from several immune cell lines demonstrates allele-specific binding. The number of mapped reads carrying rs1771575-C is significantly different in the presence of the alternative allele, indicating an allele-specific binding event. These data were obtained from the ADASTRA database.

Continuing our investigation, ChIP-seq experiments were extended to the GM12878 (B-LCL) cell line, unveiling the binding of ZNF24, CTCF, and cohesin subunits RAD21 and SMC3 at the rs1771575 site (Figure 3A). The interaction between CTCF and the cohesin complex is visually depicted in Figure 3B. Moreover, the analysis of ZNF24 binding via a position weight matrix reaffirmed a preference for binding to chromatin in the presence of the reference allele of rs1771575 (Figure 3C). We further augmented our understanding by conducting additional analysis of ChIP-seq data for CTCF binding, utilizing read counts from diverse immune cell lines as sourced from the ADASTRA database (Supplemental Table S3). This meticulous examination revealed that the count of mapped reads carrying the rs1771575-C allele significantly differed when the alternative allele was present, indicating an allele-specific binding event. This observation underscores the preferential binding of CTCF to chromatin in the presence of the C allelic variant of rs1771575 (Figure 3D).

### 2.2. Analysis of Allelic and Genotypic Frequencies of Non-Coding Regulatory and Coding FCGR2A/2B Variants in Burkina Faso Population

Volunteer families were randomly selected from 3500 inhabitants residing in a malaria-endemic area in Burkina Faso. The analyzed cohort available for genotyping comprised 136 siblings and 98 parents from 49 families. All the participants belong to the Bobo ethnic group. Table 3 shows age and gender distribution.

**Figure 4:**
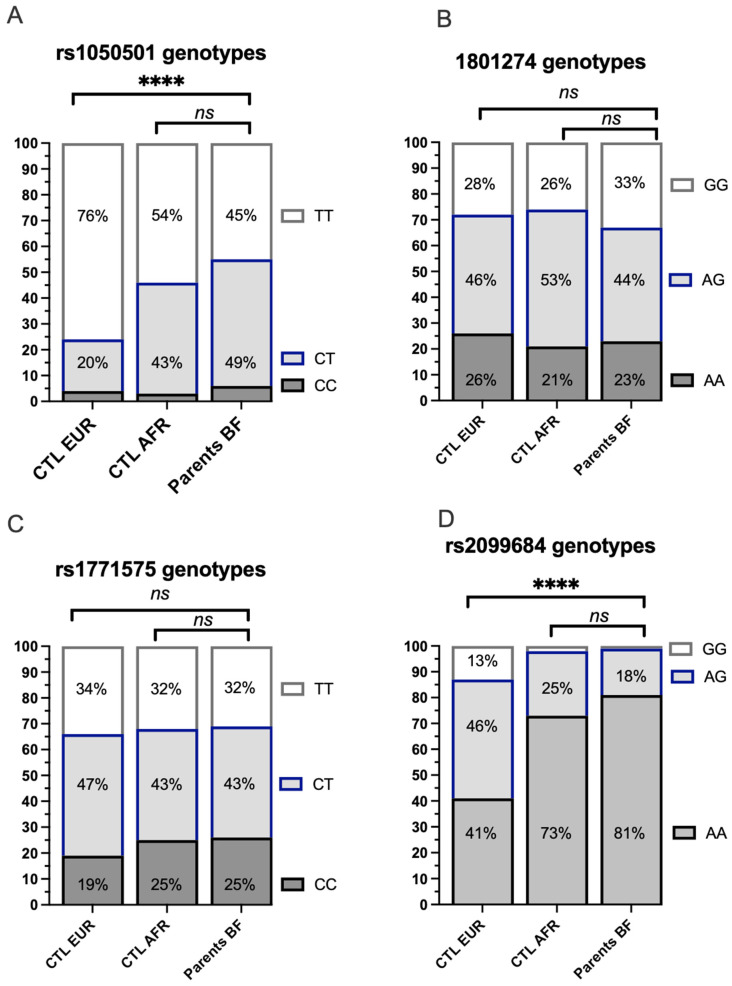
Comparison of genotype frequencies of the analyzed variants among individuals from Europe, Africa (1000 Genomes Project), and the study population from Burkina Faso (Bobo ethnic group). (**A**) Distribution of the coding *FCGR2B* rs1050501 genotype. The distribution of the coding *FCGR2B* rs1050501 genotype was analyzed in individuals from European (CTL EUR), African (CTL AFR), and unique parents from the study cohort enrolled in Burkina Faso. CC: The individual has two copies of the C allele at rs10505501, encoding an isoleucine (I) in the transmembrane domain of the FCgR2B receptor. CT: The individual has one copy of the C allele and one copy of the T allele at rs10505501.TT: The individual has two copies of the T allele at rs10505501, which encodes a threonine (T) at position 232. **** *p* < 10^−4^; Ns: non significative. (**B**) Distribution of the coding *FCGR2A* rs1801274 genotype. The distribution of the coding *FCGR2A* rs1801274 genotype was examined in individuals from European (CTL EUR), African (CTL AFR), and unique parents from the study cohort enrolled in Burkina Faso. AA: The individual has two copies of the A allele, encoding a histidine (I) in exon 4 of the Fc*γ*R2A receptor. AG: The individual has one copy of the A allele and one copy of the G allele. GG: The individual has two copies of the G allele, resulting in an arginine substitution (R) at position 131. (**C**) Distribution of the rs1771575 regulatory variant genotype. The distribution of the rs1771575 regulatory variant genotype was assessed in individuals from European (CTL EUR), African (CTL AFR), and unique parents from the study cohort enrolled in Burkina Faso. CC: The individual has two copies of the C allele. CT: The individual has one copy of the C allele and one copy of the T allele. TT: The individual has two copies of the T allele. (**D**) Distribution of the rs2099684 regulatory variant genotype. The distribution of the rs2099684 regulatory variant genotype was investigated in individuals from European (CTL EUR), African (CTL AFR), and unique parents from the study cohort enrolled in Burkina Faso. AA: The individual has two copies of the A allele. AG: The individual has one copy of the A allele and one copy of the G allele. GG: The individual has two copies of the G allele.

The frequency of heterozygous or homozygous genotypes for the 4 target SNPs was analyzed among the 98 unique parents in the study conducted in Burkina Faso and illustrated with reference to frequencies observed in African and European populations analyzed within the 1000 Genomes Project (Figure 4). The genotype frequencies observed in Burkina Faso for the 4 SNPs of interest do not differ significantly when compared to those available for African populations from the 1000 Genomes Project. The genotypes of the coding variant rs1050501 (Figure 4A) and the regulatory variant FCGR rs2099684 (Figure 4D) are significantly different from the Caucasian controls of the 1000 Genomes Project. However, the distribution of genotypes corresponding to the coding variant FCGR2A rs1801274 or the regulatory variant rs1771575 is comparable across the three populations (Figure 4B,C).

We conducted a haploview analysis to assess the linkage disequilibrium in the study population between the non-coding regulatory variants rs1771575 and rs2099684 on one hand and the coding variants rs1801274 and rs10505501 on the other hand. The results revealed no significant linkage disequilibrium between these variants in the study population (Supplemental Figure S2). Similar results were obtained when comparing them with the African 1000 genome population; the linkage disequilibrium coefficients were found to be very low (r2 < 0.001) for pairs containing a coding variant and a non-coding variant. Furthermore, there were no deviations from the Hardy-Weinberg equilibrium for rs1771575, rs2099684, rs1801274, or rs1050501 (*p* > 0.175).

Given the potential impact of rs1771575 and rs2099684 on both IgG receptors and antibody-dependent immune cell activation, we proceeded to evaluate whether these non-coding variants could be associated with the control of *P. falciparum* infection in the study cohort.

### 2.3. The Non-Coding rs1771575 Regulatory Variant Is Associated with Plasmodium falciparum Infection

The distribution of non-coding variants (rs2099684 and rs1771575) and coding variants (rs1801274 and rs1050501) was analyzed in relation to the prevalence of *P. falciparum* infection (Prev*Pf*I) in a cohort of 136 children and their parents, with parasite measurements taken at different time points. It should be stressed that parasitemia was measured for all the participants during the dry and rainy seasons, which correspond to the seasons of low and high transmission.

**Figure 5:**
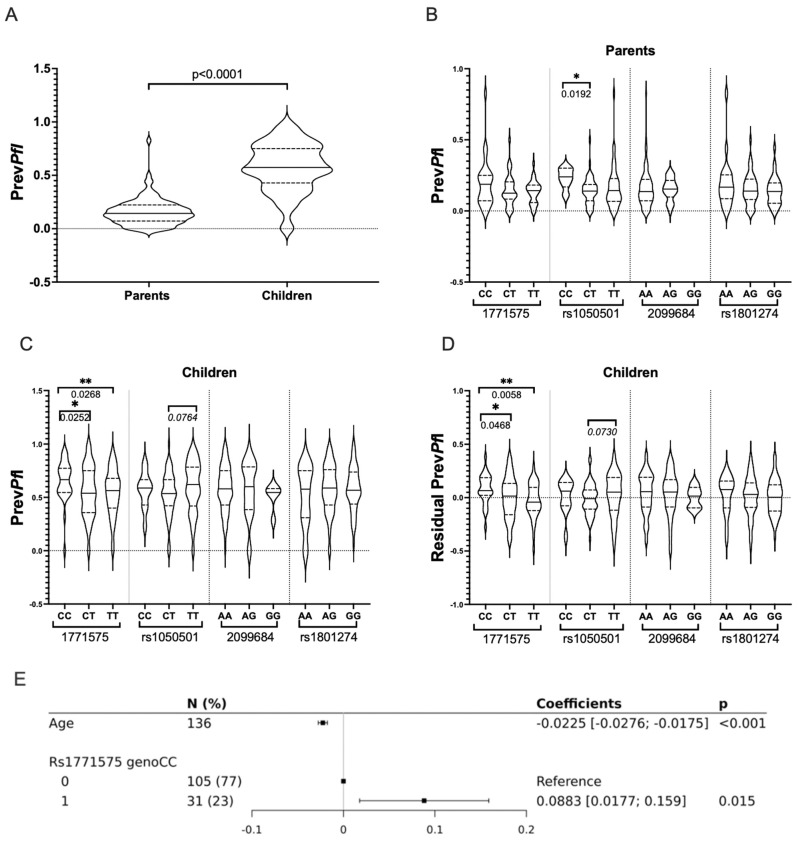
Prevalence of *P. falciparum* Infection (Prev*Pf*I) Across Variant Genotypes in Infants and Adults from Burkina Faso. (**A**) Comparative analysis of *P. falciparum* infection prevalence (Prev*Pf*I) in parents and children. A comparative analysis of *P. falciparum* infection prevalence (median Prev*Pf*I values) was conducted in parents and children using the nonparametric Mann–Whitney U-test for non-normally distributed data. Significant *p*-values (≤0.05) are indicated. The prevalence of *P. falciparum* infection (Prev*Pf*I) for each individual was calculated as the number of positive blood samples divided by the total number of blood samples evaluated for each individual. This was considered the quantitative phenotype outcome variable. (**B**) Comparative analysis of *P. falciparum* infection prevalence (Prev*Pf*I) in parents across rs1771575, rs1050501, rs2099684, and rs1801274 genotypes. A comparative analysis of *P. falciparum* infection prevalence (median Prev*Pf*I values) in parents was performed across rs1771575, rs1050501, rs2099684, and rs1801274 genotypes. Significant *p*-values (<0.05) are indicated, and trends (*p* < 0.100) are indicated in italics. * refers to *p* values ≤ 0.05. (**C**) Comparative analysis of *P. falciparum* infection prevalence (Prev*Pf*I) in children across rs1771575, rs1050501, rs2099684, and rs1801274 genotypes. A comparative analysis of *P. falciparum* infection prevalence (median Prev*Pf*I values) in children was conducted across rs1771575, rs1050501, rs2099684, and rs1801274 genotypes. Significant *p*-values (<0.05) are indicated, and trends (*p* < 0.100) are indicated in italics. * refers to pvalues ≤ 0.05, ** refers to *p* values ≤ 0.01 (**D**) Comparative analysis of residual *P. falciparum* infection prevalence (Prev*Pf*I adjusted for age effect) in children across rs1771575, rs1050501, rs2099684, and rs1801274 genotypes. A comparative analysis of residual *P. falciparum* infection prevalence (median Prev*Pf*I values) adjusted for age effect was performed in children across rs1771575, rs1050501, rs2099684, and rs1801274 genotypes. Residuals of Prev*Pf*I were calculated for each person after conducting a linear regression analysis between Prev*Pf*I and age. * refers to *p* values ≤ 0.05, ** refers to pvalues ≤ 0.01 (**E**) Multiple linear regression analysis to test effect on outcome variable Prev*Pf*I. Multiple linear regression was carried out to assess the effect on the outcome variable Prev*Pf*I, taking into account age and the recessive model (CC: encoded 1) vs. (CT/TT: encoded 0) explanatory variables.

It was found that Prev*Pf*I was not influenced by gender, but it showed an inverse correlation with age in both adults and children (*p* < 0.0001). Consequently, higher Prev*Pf*I values were observed in children compared to their parents (Figure 5A).

In the univariate analysis, it was observed that the CC genotype of the rs1050501 *FCGR2B* coding variant was associated with higher Prev*Pf*I in adults. However, the genotypes of the non-coding variants (rs2099684 and rs1771575) or the coding variant (rs1801274 *FCGR2A*) did not show any significant association with Prev*Pf*I when considering the parent’s group (Figure 5B).

In contrast, while the rs1050501 and rs1801274 coding variants, as well as the rs2099684 non-coding variant, were not significantly associated with Prev*Pf*I in children, the regulatory rs1771575 genotypes were found to be associated with infant Prev*Pf*I. Particularly, the rs1771575 CC genotype was significantly associated with enhanced Prev*Pf*I in children compared to those carrying the T allele (*p* = 0.012, Table 4 and Figure 5C). Similar association results were obtained for rs1771575 CC when Prev*Pf*I values were adjusted for age in children (Figure 5D).

Taking into account the impact of children’s age on parasitemia, an association of rs1771575 (and rs1050501) genotypes with Prev*Pf*I was further tested using multiple linear regression models. When using a recessive model, testing rs1771575 CC versus CT and TT genotypes in children, a significant association between rs1771575 and Prev*Pf*I was confirmed (Table 4, illustrated in Figure 5E). Using an additive genetic model, a significant association was found between rs1771575 and Prev*Pf*I (Table 4), while no significant association was observed with rs1050501; however, a trend for a higher Prev*Pf*I was seen in children with the TT genotype. This result was consistent with the univariate analysis based on Prev*Pf*I adjusted for age (Figure 5D), which showed a higher infection value for rs1771575 CC individuals. It was thus demonstrated that the CC genotype of rs1771575 was significantly associated with lower Prev*Pf*I in children, even when taking into account rs1050501.

To account for the family effect, multivariate family-based association tests were conducted. Using a mixed model that incorporated the family effect, the association between Prev*Pf*I and rs1771575 in children was detected based on both the additive and recessive models (Table 4). The QTDT approach confirmed this association (*p* = 0.009) using the additive model. However, Prev*Pf*I was not associated with rs2099689, rs1801274, or rs1050501 using family-based association tests.

After correcting for all the statistical tests performed, the association between rs1771575 and Prev*Pf*I remained significant using the multiple linear regression and mixed model methods, as well as the QTDT approach.

## 3. Discussion

Interaction between FcγR and the Fc domain of IgG plays a significant role in activating innate immune cells against parasites. Several studies have explored the association between FCGR gene variants in coding regions and protection against malaria [29]. However, discrepancies have been observed, indicating different associations in children and adults and variations across ethnic groups, both within and outside Africa [50,51]. Among the commonly investigated coding mutations is *FCGR2A*-H131R (rs1801274), which affects FcγRIIA/CD32a-mediated activation of monocytes and leads to changes in IgG recognition and IgG parasite phagocytosis. While a meta-analysis suggested that the H allelic variants resulting from the rs101274 variation may be protective against blood-stage malaria infection [28], our study in adults and infants from Burkina Faso did not observe such an association. This finding aligns with previous reports by Cherif et al., who found no significant association between the rs1801274 variant and *P. falciparum* prevalence in children from the Mossi ethnic group in Burkina Faso [52]. In that study, a frequency of 14.6% was documented for the FCGR2A-131HH (rs1801274-AA) homozygous genotype. These divergences in the correlation between FcγR haplotypes and malaria susceptibility might be partially attributed to discrepancies in the prevalence of the FCGR2A-131HH homozygous genotype across various populations. For instance, the Yoruba group in Niger, which shares geographical proximity with the Burkina Faso ethnic group (unavailable in the 1000 Genomes African project), displayed a frequency of 20.5% for this genotype. The observed frequency of 23% for the rs1801274-AA homozygous genotype in our current study of Burkinabe populations aligns with the prevalence of the same combination observed in the Yoruba subpopulation analyzed within the 1000 Genomes African project. In contrast, the Luhya African population in Kenya, as analyzed in the 1000 Genomes project, showed a higher frequency of 32% for the *FCGR2A*-131HH homozygous genotype [50].

In this study, we observed that the CC-genotype at position rs1050501 in the *FCGR2B* gene was associated with enhanced parasitemia in adults. However, this association did not remain significant when considering age as a covariable. Our findings align with the previously described protective effect of the *FCGR2B* rs1050501-TT genotype against malaria and infant susceptibility to HIV infection [40,41], but we did not find evidence of its protective role in controlling parasitemia in children analyzed in Burkina Faso, mainly belonging to the Bobo ethnic subgroup. These discrepancies may partly depend on age-related factors, which manifest differently in adults with a more mature immune response controlling the parasite.

Malaria has significantly shaped FCGR variability in Africans in endemic regions. The lack of association between *FCGR2A* and *FCGR2B* coding variants with parasitemia could also be attributed to ethnic variations [51], sample size, or the diversity of observed malaria phenotypes, including parameters such as socioeconomic status, access to healthcare, and control measures in previously described cohorts. Studies on the population diversity of FcγR variants have shown differences in ethnic variation, allele distribution, and linkage disequilibrium at the FCGR gene locus, especially for *FCGR2B* and *FCGR2C*, among Kenyan, Nigerian, South African, and Caucasian populations [40,41]. The rs1050501 SNP in *FCGR2B* has notably been shown to be under malaria-driven selective evolutionary pressure [27], and minor allele frequencies of FcγRIIb-232T and FcγRIIIb-HNA1 FCGR variants associated with protection against clinical malaria were reported to be more prevalent in malaria-endemic regions than in non-endemic regions.

In addition to these ethnic variations, the lack of strong associations with coding mutations that alter the function of FcγR in controlling parasitemia may suggest the involvement of other regulatory genetic and epigenetic mechanisms that could potentially fine-tune cell- and individual-specific acquisition of host immune responsiveness to *P. falciparum* exposure in a given environment.

These population-specific variations in genotype frequencies may play a significant role in the observed differences in malaria susceptibility and protection. They highlight the importance of considering the genetic diversity of different populations when studying the role of FcγR haplotypes in disease susceptibility or protection. Furthermore, such differences underscore the need for more extensive genetic studies of functional and regulatory variants across diverse populations to gain a comprehensive understanding of the complex interactions between FcγR variants and malaria outcomes.

Conflicting findings concerning coding FCGR polymorphisms and malaria control suggest that other co-regulatory mechanisms may play a role in controlling the balance between activating FcγR and inhibitory FcγRIIB-signaling in response to *P. falciparum* exposure. Coding mutations in the *FCGR2B* gene have been shown to modulate immune activation in response to *Pf* challenge. influencing the capacity to dampen the function of activating Fcγ receptors. However, compared to the *FCGR2A* rs1801274 counterpart, *FCGR2B,* and *FCGR3B* polymorphisms have only been assessed in a limited number of studies [30,40,53,54].

In this context, our current investigation aimed to explore the potential influence of non-coding genetic variants on the regulatory networks shaping the cell-specific FcγR repertoire of immune cells. The main finding of our study is that the rs1771575 non-coding cis-regulatory variant, identified as an expression quantitative trait locus (eQTL) selective for several FCGR gene targets using in silico approaches, results in different capacities to control parasitemia according to infant genotype. Our bioinformatic analysis also reveals that the rs1771575 regulatory variant is associated with ATAC-seq and H3K4me1 modifications in active regulatory regions of monocytes and neutrophils, indicating potential cell-specific enhancer activity in these cell types. Additionally, the rs1771575 mutation affects allele-specific binding of various transcription factors (SOX2-10-15, NCR3C1, POU5F1, and ZNF24) that may influence the cell-specific tuning of host immune responsiveness upon pathogen encounter. We also identify a preferential binding of CTCF to chromatin when the rs177157C allelic variant is present, which may influence enhancer-promoter interactions, favoring coordinated and cell-specific activation of the targeted FCGR gene expression that contributes to the acquisition of an efficient immune response.

In this study, we utilized various tools to conduct a large-scale immune cell-specific analysis of expression quantitative trait loci (eQTL), providing valuable insights into the impact of the rs1771575 variant on the coordinated expression of the FCGR gene repertoire in neutrophils. Our analysis of the ImmuNexUT database revealed that the rs1771575-CC variant is significantly associated with lower gene expression of *FCGR2B* inhibitory receptors and *FCGR3A* activating receptor transcripts in neutrophils. However, the effect on activating *FCGR3B* and *FCGR2A* gene expression in this cell type was less significant.

Previous research suggests that Fcγ receptors on peripheral blood neutrophils play a crucial role as effector phagocytes against *P. falciparum* blood-stage merozoites, contributing to the elimination of malaria-infected cells and protecting against febrile malaria [11]. However, the relationship between neutrophil phagocytosis and protection against malaria is not fully understood. When antibodies bound to malaria-infected cells interact with Fc-receptors on neutrophils, it triggers the release of pro-inflammatory cytokines and chemokines, promoting phagocytosis of the antibody-coated infected cells or activating reactive oxygen species (ROS) granule release of antimicrobial molecules [10,14], contributing to the destruction of malaria parasites and infected cells. *FCGR2A* receptor expression by neutrophils is reported as the dominant activating FcγR mechanism that allows the elimination of merozoites [47]. Additionally, studies have shown significant changes in intermediate/inflammatory monocyte phenotypes and proinflammatory and anti-inflammatory transcriptional profiles associated with the regulation of monocyte subset phagocytic function in response to *P. falciparum* infection in children living in malaria-endemic areas [51]. Proper control of this balance in the immune response is crucial in the context of malaria infection, as dysregulated activation of neutrophils can also contribute to the severity of the disease [10,14], leading to the destruction of malaria parasites and infected cells.

Despite evidence linking coding variants to neutrophil or monocyte immune function, our current understanding of the genetic and epigenetic mechanisms that coregulate the expression levels of FcγRs and their impact on the activatory-to-inhibitory balance of the FcγR repertoire, enabling immune cells to control the parasite, remains limited. Our findings indicate that the variation at the rs1771575 locus may have a lesser impact on *FCGR2A* gene expression in monocytes, dendritic cells, and neutrophils compared to other activating receptors, such as *FCGR3A*, *FCGR3B*, and *FCGR2C*. This suggests that the rs1771575 regulatory variant may not significantly affect the CD32a-mediated activation pathway but rather influences the balance between *FCGR2B*/CD32b inhibitory and *FCGR3A*/CD16a, *FCGR3B*/CD16b activating signaling in neutrophils.

Further research is needed to fully comprehend the complex mechanisms associating the polymorphism of the rs1771575 regulatory variant with its functional impact on immune cells. Therefore, a more in-depth analysis of the rs1771575 eQTL profiles may offer valuable insights into the coordinated fine-tuning of the FcγR-mediated immune cell activation in neutrophils and CD16^+^ monocytes in response to pathogen challenges. Such investigations of regulatory mechanisms are crucial for a better understanding of how immune cells respond to malaria infection and may have implications for the development of targeted therapies or interventions to combat the disease.

It is essential to acknowledge that our study has several limitations. Firstly, we focused solely on single-gene polymorphisms of FcγR regulatory variants, particularly on the commonly assessed *FCGR2A* and *FCGR2B* coding variants. However, we did not consider the additional complexity of haplotypic diversity described for *FCGR3A*, *FCGR3B*, and *FCGR2C*, nor did we investigate gene duplication/deletion mechanisms that affect gene copy number variations (CNVs) in the 1q23 cluster. These factors may also directly impact the expression level and function of these receptors [21,50,54]. In light of the mentioned haplotypic combinations, it is worth noting that a specific FcγRIIA-131R/FcγRIIIA-176F, FcγRIIIb-NA2 haplotype was linked to increased susceptibility to malaria in children from western Kenya [25]. Conversely, studies have shown that the absence of the G3m6 (+)/FcγRIIA-131H/FcγRIICT/FcγRIIIA-176F/FcγRIIIB-NA2 haplotypic combination provides natural protection to young Fulani individuals from northern Benin with decreased susceptibility to malaria infections [55].

Despite these limitations, our study provides initial evidence that the rs1771575 eQTL, located in an intergenic regulatory region, is the sole FCGR polymorphic variant analyzed significantly associated with blood-stage parasitemia in children living in Burkina Faso, regardless of age and the presence of *FCGR2A*/*FCGR2B* coding allelic variants previously associated with protective immunity against malaria. The regulatory variant appears to allow coordinated host-specific regulation of both activating and inhibitory FcγR gene expression in various immune cell types, particularly neutrophils.

Understanding these regulatory mechanisms is crucial to characterizing interindividual variability in host responsiveness and gaining better insights into genetic features that confer disease susceptibility or vaccination efficacy in children. Therefore, further exploration is needed to enhance our understanding of the hidden regulatory functions of FCGR eQTL mapping during dynamic and cell-specific immune processes, which may explain why some children are more susceptible to parasite burden in malaria-endemic regions. Thus, comprehending how non-coding variants regulate the variability in the FcγR-dependent capacity of children to control parasitemia becomes a crucial challenge.

In conclusion, our study sheds light on the potential role of non-coding genetic variants in influencing the coordinated expression of FcγRs in immune cells and their impact on the immune response to *P. falciparum* infection. Further investigations into the genetic and epigenetic mechanisms that regulate FcγR expression and function may open new avenues for understanding the complexity of host-parasite interactions and contribute to the development of more targeted approaches for malaria control and prevention.

## 4. Materials and Methods

### 4.1. Study Population

The study participants reside in Logoforousso, a rural village located to the southwest of Bobo-Dioulasso in Burkina Faso. All participants belong to the Bobo ethnic group, a Mande ethnic group living primarily in Burkina Faso. The local population and the extent of parasite exposure in the area have been thoroughly documented [56]. Volunteer families were randomly selected from a total of 3500 inhabitants. Before the study, informed consent was obtained from all participants individually or from their parents if they were minors. The research protocol was duly approved by the national medical authorities of Burkina Faso.

For the mosquito capture, four specific sites were chosen, and mosquitoes were collected outdoors over four days each month, occurring on two nights every two weeks. The inoculation rate was set at 230 infective bites per person per year. Malaria transmission occurred mainly during the rainy season.

Initially, the study encompassed 234 subjects from 49 pedigrees, forming 50 nuclear families comprising 98 parents and 136 siblings. These individuals were selected for genotyping of variants and retained for genetic association analysis. The characteristics of the genotyped individuals can be found in Table 3.

### 4.2. Phenotyping

Parasitemia was measured as described [57]. During the 18 months of the study, each family in the rural area was visited 28 times. In other words, each family was visited once every three weeks during the dry and rainy seasons. Blood samples were collected from all individuals present, and only measurements of asymptomatic *P. falciparum* parasitemia were included in this study. The median values of asymptomatic *P. falciparum* parasitemia per subject were 18 for parents and 20 for children. Fingerprint peripheral blood samples were obtained from all family members present, and thick and thin blood films were stained with Giemsa for analysis. The determination and quantification of parasites were performed independently by two individuals in a blind manner. Only asexual forms of *P. falciparum* were considered to calculate parasitemia. Parasitemia was defined as the count of parasitized erythrocytes observed per µL in thin blood films.

For each individual, the prevalence of *P. falciparum* infection (Prev*Pf*I) was calculated as the ratio of positive blood samples to the total number of blood samples evaluated. It is important to note that PrevPfI was assessed in 98 adult parents and 136 infants (Table 3). In certain analyses, PrevPfI was adjusted for age effects. Specifically, residuals of Prev*Pf*I were computed for each individual following a linear regression analysis between Prev*Pf*I and age.

### 4.3. Bioinformatic Identification of Non-Coding Variants

The selection of Fc gamma receptor SNPs was conducted using a series of filtering criteria. SNPs with expression quantitative trait locus (eQTL) annotation in whole blood for *FCGR2A*, *FCGR2B*, *FCGR2C*, *FCGR3A*, and *FCGR3B* genes were obtained by querying the GTEX version 8 database [32] (Supplemental Table S1). Subsequently, the RegVar tool [33], which utilizes the GTEX version 7 database, was employed to prioritize the SNPs based on gene-specific and tissue-specific annotations. The top 20 SNPs with the highest scores were selected (Supplemental Table S2). While a RegVar threshold of 0.23, optimized for sensitivity and specificity, was recommended for whole blood, we found that the specificity at this threshold was too low, with a false positive rate of 31% [33]. Therefore, a decision was made to substantially reduce the false positive rate to 0.2%, guided by the specificity study conducted by Lu et al. [33]. Furthermore, the eQTL annotations of these 20 selected SNPs were cross-checked in two independent immune cell eQTL databases, namely ImmuNexUT [45] and the ebi eQTL catalog [46]. SNPs were retained if they had annotations in both of the aforementioned databases. The positions of the eQTL candidates were cross-referenced with ATAC-seq regions in immune cells [47] and regions binding transcription factors in immune cells using the ReMap catalog [54]. The ReMap data were filtered by primary NK, T, and B lymphocytes, monocytes, macrophages, neutrophils, peripheral blood cells, and monocytic and lymphocytic cell lines. The RSAT tool [55] was then employed to assess whether the allele of an SNP influenced the affinity of the transcription factor for the sequence containing the polymorphism. The databases of selected transcription factor motifs in the RSAT parameters were JASPAR and ENCODE. All other parameters were set to their default values. The presence of epigenetic marks (ATAC-seq, H3K4me1, H3K4me3, H3K27ac, and H3K27me3) in monocytes, macrophages, NK cells, and B cells was investigated using the WashU Epigenome Browser and data from the CistromeDB database and ENCODE portal [49]. The threshold allele frequency for the minority allele was set at 10% to ensure sufficient statistical power for the analysis of malaria cohorts. This criterion avoids selecting SNPs with very low representation in the cohorts used. LDlink, a tool including LD information from the 1000 Genome project, was used to identify the SNPs in linkage disequilibrium (LD) [58]. Through this comprehensive selection process, the most relevant and informative Fc gamma receptor SNPs were identified and prioritized for further analysis in the context of malaria infection.

### 4.4. Genotyping of FCGR Variants

Blood samples were collected using venipuncture, and DNA was extracted from mononuclear cells separated by a Ficoll-Hypaque density gradient following the described procedure (27). Before genotyping, the samples underwent whole-genome amplification using the primer extension pre-amplification method. The genotyping of the *FCGR2B*-232I/T (rs1050501) SNPs was performed using a homemade TaqMan discrimination assay. The TaqMan assay was designed using Software Primer Express v3.0 (Applied Biosystems, Inc., Foster City, CA 94404 USA). The TaqMan genotyping assay for *FCGR2B* included a sense primer (TGGGGATCATTGTGGCTGTG), an antisense primer (TACACTGCTCTCCCCAAGAC), and two probes, MGB (ACTGGGACTGCTGTAG for the C allele coding for Thre and ACTGGGATTGCTGTAG for the T allele coding for Iso). The TaqMan genotyping assays were carried out on the QuantStudio 6 Flex (Applied Biosystems Inc.) with the following protocol: [50 °C, 2 min—95 °C, 10 min—[95 °C, 15 s—61 °C, 1 min] *40 cycles]. The specificity and accuracy of the TaqMan assays were validated by confirming a perfect match (100%) with the genotypes obtained by Sanger sequencing in 20 randomly selected human subjects. In each TaqMan assay, positive controls included two homozygous FCG2B alleles (TT and CC) and one heterozygous TC FCG2B allele.

For the genotyping of variations at rs1771575, rs2099684, and rs1801274. TaqMan genotyping assays (Thermo Fisher Scientific, Massachusetts, MA, USA) with IDs C_26275257_10, C_16118535_20, and C_9077561_20, respectively, were used. PCR amplification was performed on the QuantStudio 6 Flex Real-Time PCR Systems (Life Technologies, Waltham, MA, USA) in a final volume of 5 μL, containing 2.1 μL of Master Mix (Life Technologies, Waltham, MA, USA), 0.06 μL of TaqMan probe, 1.84 μL of H_2_O, and 1 μL of DNA (10–15 ng/μL).

### 4.5. Statistical Analyses

Univariate analyses were carried out using Graphpad Prism to assess the association between malaria-related phenotypes and genotypes. Non-parametric Mann-Whitney tests were used to compare median values of Prev*Pf*I across genotypes. A simple linear regression was carried out to calculate the residual for Prev*Pf*I when including age in the model. The residuals were used as phenotypes for some association analyses. As age was shown to influence the prevalence of *P. falciparum* infection, the age effect was further taken into account. Multiple linear regression was carried out to test the effect on the outcome variable Prev*Pf*I, taking into account age and rs1771575. CC:0, CT:1, TT:2, (additive model) or CC:1 vs. CT/TT 0 (recessive model) are the explanatory variables.

Linear mixed models and transmission/disequilibrium (QTDT 2.6.0 program) approaches based on the standard variance components method were used to take into account the family effect [59]. Variance components were used to construct a test that utilizes information from all available offspring. Total association effects can be partitioned into two orthogonal components, which measure between- and within-family effects. Stratification in the population can be assessed by comparing between- and within-family components. Within-family association and association including within- and between-family components can be performed using the orthogonal model and the total association model, respectively. Evidence of association can be evaluated by the likelihood ratio test (null hypothesis likelihood L0 versus alternative hypothesis likelihood L1). Asymptotically, the quantity 2(lnL1-lnL0) is distributed as a chi-square with df equal to the difference in number of parameters estimated. The q values were calculated based on all the statistical tests performed to control for the false discovery rate. A threshold of 0.1 was applied to correct for multiple tests.

## 5. Conclusions

In conclusion, our study presents preliminary evidence of an association between an intergenic variant that selectively regulates *FCGR* gene expression and the prevalence of malaria infection in children residing in Burkina Faso. By highlighting the crucial role of non-coding genetic regulatory variants in controlling *FCGR* expression, this research sheds light on the intricate interactions governing immune responses and disease susceptibility. It also acts as a catalyst for further investigations into the functional implications of these regulatory variants, thereby advancing our understanding of the genetic mechanisms influencing the highly variable capacity of individuals to resist *P. falciparum* infection in African populations.

This work holds significant promise for contributing to a broader understanding of the specific genetic and epigenetic factors that influence immune responses. Consequently, it may guide the development of personalized treatments and vaccine strategies tailored to the diverse host responses to infectious diseases like malaria.

## Figures and Tables

**Table 1:** Structural and functional annotations of the studied SNPs.

				Functional Regulatory Annotation		
SNP	Position ^a^	Annotation ^b^	% Genotyped	ATAC-Seq ^c^	ReMap ^c^	Encode Regulatory Element
rs1771575 (T > C)	161647707	intergenic	100.0	DC, Mo, NK, B	Mo, Ma, N, B	E1391464
rs2099684 (A > G)	161530340	intergenic	96.5	DC, NK	Mo, B	E1391397
rs1801274 (G > A)	161509955	*FCGR2A* exon, missense H-R	97.4	-	-	-
rs1050501 (T > C)	161674008	*FCGR2B* exon, missense I-N	97.5	-	-	-

^a^ Data represent the position on chromosome 1 according to human hg38 coordinates. ^b^ Structural annotation and amino acid change for missense mutations. ^c^ The cell types, in which the SNP is located in ATAC-seq peaks [47] and TF binding regions from the ReMap catalog [48], are stated. B cells (B); Dendritic Cells (DC); Monocytes (Mo); Macrophages (Ma); Natural Killer cells (NK); Neutrophils (N).

**Table 2:** eQTLs in immune cells according to ImmuNexUT database and EBI catalogue.

	eQTLs from ImmuNexUT Database			eQTLs from EBI Catalog		
	Target Gene	Cell Type	*p* Value ^a^	Target Gene	Cell Type	*p* Value ^b^
rs1771575	*FCGR2B*	Neutrophils	3.10 × 10^−38^	*FCGR2B*	Neutrophils	1.15 × 10^−10^
	*FCGR3A*	Neutrophils	6.02 × 10^−26^	*FCGR2B*	Monocyte, naive	9.77 × 10^−7^
	*FCGR2C*	Dendritic cells	2.77 × 10^−17^	*-*	-	-
	*FCGR3B*	NK cells	1.16 × 10^−5^	*-*	-	-
	*FCGR2A*	Dendritic cells	2.87 × 10^−4^	*-*	-	-
rs2099684	*FCGR3B*	NK cells	8.54 × 10^−78^	*FCGR3B*	Monocyte, naive	6.31 × 10^−23^
	*FCGR2C*	NK cells	2.63 × 10^−17^	*FCGR3B*	Monocyte, LPS	1.35 × 10^−18^
	*FCGR2A*	Neutrophils	6.68 × 10^−15^	*FCGR3B*	Monocyte, IAV	3.24 × 10^−18^
	*FCGR2B*	Neutrophils	1.227 × 10^−5^	*FCGR3B*	Monocyte	3.55 × 10^−17^
	-	-	-	*FCGR3B*	NK-cell, naive	4.68 × 10^−16^
	-	-	-	*FCGR3B*	Macrophage, Salmonella	1.78 × 10^−13^
	-	-	-	*FCGR3B*	Macrophage, naive	5.62 × 10^−13^
	-	-	-	*FCGR3B*	Macrophage, Listeria	6.17 × 10^−13^
	-	-	-	*FCGR3B*	CD16 Monocyte, naive	1.07 × 10^−11^

^a^ *p* values were obtained from the ImmuNexUT database. ^b^ *p* values were obtained from the eQTL EBI catalogue.

**Table 3:** Characteristics of Study participants.

	Parents	Children
	(N = 98)	(N = 136)
Gender		
N	98	136
Female	65	71
Male	33	65
Ethnic group: Bobo ^a^ N	98	136
Age		
N	98	136
Median	39	8
(25–75th percentile)	(31–45)	(6–12)
Number of parasitaemia measurements per subject		
N	98	136
Median	18	20
(25th and 75th percentile)	(13–23)	(14–24)
Phenotypes related to infection levels (Prev*PfI*)		
N	98	136
Median	0.14	0.57
(25th and 75th percentile)	(0.07–0.22)	(0.43–0.75)

^a^ The Bobo are a Mande ethnic group living primarily in Burkina Faso. The prevalence of *P. falciparum* infection (Prev*Pf*I) was calculated for each individual as the number of positive blood samples divided by the total number of blood samples evaluated for each individual.

**Table 4:** Univariate and Multivariate Analysis of rs1771575 Association with Prevalence of *P. falciparum* Infection (Prev*Pf*I).

Explanatory Variable: Prev*Pf*I ^a^	Univariate Analysis	Linear Multiple Regression Adjusted to Age		Family Based Linear Mixed Model	
	*p*	b Coefficient (IC)	*p*	b Coefficient (IC)	*p*
^b^ Recessive Model					
Children age	<0.001 *	−0.0225 [−0.0276; −0.0175]	<0.001 *	−0.023 (−0.028; −0.018)	<0.001 *
rs1771575-CC vs. CT/TT	0.012 *	0.0883 [0.0177; 0.159]	0.015 *	0.087 (0.017; 0.158)	0.016 *
^c^ Additive Model					
Children age	<0.001 *	−0.0227 [−0.0277; −0.0177]	<0.001 *	−0.023 (−0.028; −0.018)	<0.001 *
rs1771575- CC vs. -CT vs. -TT	0.0252 *	−0.0525 [−0.0941; −0.0108]	0.014 *	−0.052 (−0.094; −0.010)	0.016 *

^a^ Prevalence of *P. falciparum* infection (Prev*Pf*I) was calculated for each individual as the number of positive blood samples divided by the total number of blood samples evaluated for each individual. It was considered as the quantitative phenotype outcome explanatory variable. Children age was used as a significant adjustment variable in both Linear Multiple Regression and Family Based Linear Mixed Model multivariate analyses. Genotype-based analysis for ^b^ recessive (rs1771575-CC vs. CT/TT) or ^c^ additive (rs1771575 -CC vs. -CT vs. -TT). Significant *p* values < 0.05 are indicated. * *p* remained significant after correcting for multiple tests.

## Data Availability

The datasets used and/or analyzed during the current study are available from the corresponding author on reasonable request.

## Supplementary Materials

The following supporting information can be downloaded at: https://www.mdpi.com/article/10.3390/ijms242115711/s1.

## References

[1] (2022). World Malaria Report. https://www.who.int/teams/global-malaria-programme/reports/world-malaria-report-2022.

[2] Kwiatkowski D.P. (2005). How malaria has affected the human genome and what human genetics can teach us about malaria. Am. J. Hum. Genet..

[3] Esoh K.K., Apinjoh T.O., Amambua-Ngwa A., Nyanjom S.G., Chimusa E.R., Amenga-Etego L., Wonkam A., Achidi E.A. (2023). Genome-wide association study identifies novel candidate malaria resistance genes in Cameroon. Hum. Mol. Genet..

[4] Damena D., Agamah F.E., Kimathi P.O., Kabongo N.E., Girma H., Choga W.T., Golassa L., Chimusa E.R. (2021). Insilico Functional Analysis of Genome-Wide Dataset from 17,000 Individuals Identifies Candidate Malaria Resistance Genes Enriched in Malaria Pathogenic Pathways. Front. Genet..

[5] Moncunill G., Scholzen A., Mpina M., Nhabomba A., Hounkpatin A.B., Osaba L., Valls R., Campo J.J., Sanz H., Jairoce C. (2020). Antigen-stimulated PBMC transcriptional protective signatures for malaria immunization. Sci. Transl. Med..

[6] Tran T.M., Guha R., Portugal S., Skinner J., Ongoiba A., Bhardwaj J., Jones M., Moebius J., Venepally P., Doumbo S. (2019). A Molecular Signature in Blood Reveals a Role for p53 in Regulating Malaria-Induced Inflammation. Immunity.

[7] Antonelli L.R.V., Leoratti F.M.S., Costa P.A.C., Rocha B.C., Diniz S.Q., Tada M.S., Pereira D.B., Teixeira-Carvalho A., Golenbock D.T., Gonçalves R. (2014). The CD14^+^CD16^+^ inflammatory monocyte subset displays increased mitochondrial activity and effector function during acute *Plasmodium* vivax malaria. PLoS Pathog..

[8] Dechavanne C., Nouatin O., Adamou R., Edslev S., Hansen A., Meurisse F., Sadissou I., Gbaguidi E., Milet J., Cottrell G. (2022). Placental Malaria is Associated with Higher LILRB2 Expression in Monocyte Subsets and Lower Anti-Malarial IgG Antibodies During Infancy. Front. Immunol..

[9] Royo J., Rahabi M., Kamaliddin C., Ezinmegnon S., Olagnier D., Authier H., Massougbodji A., Alao J., Ladipo Y., Deloron P. (2019). Changes in monocyte subsets are associated with clinical outcomes in severe malarial anaemia and cerebral malaria. Sci. Rep..

[10] Nziza N., Tran T.M., DeRiso E.A., Dolatshahi S., Herman J.D., Lacerda L.d., Junqueira C., Lieberman J., Ongoiba A., Doumbo S. (2023). Accumulation of Neutrophil Phagocytic Antibody Features Tracks with Naturally Acquired Immunity Against Malaria in Children. J. Infect. Dis..

[11] Garcia-Senosiain A., Kana I.H., Singh S., Das M.K., Dziegiel M.H., Hertegonne S., Adu B., Theisen M. (2021). Neutrophils dominate in opsonic phagocytosis of P. falciparum blood-stage merozoites and protect against febrile malaria. Commun. Biol..

[12] Chimma P., Roussilhon C., Sratongno P., Ruangveerayuth R., Pattanapanyasat K., Pérignon J.-L., Roberts D.J., Druilhe P. (2009). A distinct peripheral blood monocyte phenotype is associated with parasite inhibitory activity in acute uncomplicated Plasmodium falciparum malaria. PLoS Pathog..

[13] Waisberg M., Molina-Cruz A., Mizurini D.M., Gera N., Sousa B.C., Ma D., Leal A.C., Gomes T., Kotsyfakis M., Ribeiro J.M.C. (2014). *Plasmodium falciparum* infection induces expression of a mosquito salivary protein (Agaphelin) that targets neutrophil function and inhibits thrombosis without impairing hemostasis. PLoS Pathog..

[14] Ofori E.A., Garcia-Senosiain A., Naghizadeh M., Kana I.H., Dziegiel M.H., Adu B., Singh S., Theisen M. (2023). Human blood neutrophils generate ROS through FcγR-signaling to mediate protection against febrile P. falciparum malaria. Commun. Biol..

[15] Tangteerawatana P., Krudsood S., Kanchanakhan N., Troye-Blomberg M., Khusmith S. (2014). Low monocyte to neutrophil ratio in peripheral blood associated with disease complication in primary Plasmodium falciparum infection. Southeast Asian J. Trop. Med. Public. Health.

[16] Feng G., Wines B.D., Kurtovic L., Chan J.-A., Boeuf P., Mollard V., Cozijnsen A., Drew D.R., Center R.J., Marshall D.L. (2021). Mechanisms and targets of Fcγ-receptor mediated immunity to malaria sporozoites. Nat. Commun..

[17] Dick J.K., Hart G.T. (2022). Natural Killer Cell Antibody-Dependent Cellular Cytotoxicity (ADCC) Activity Against Plasmodium falciparum-Infected Red Blood Cells. Methods Mol. Biol..

[18] Bouharoun-Tayoun H., Druilhe P. (2015). Antibody-Dependent Cell-Mediated Inhibition (ADCI) of *Plasmodium falciparum*: One- and Two-Step ADCI Assays. Methods Mol. Biol..

[19] Pleass R.J. (2009). Fc-receptors and immunity to malaria: From models to vaccines. Parasite. Immunol..

[20] Tiendrebeogo R.W., Adu B., Singh S.K., Dziegiel M.H., Nébié I., Sirima S.B., Christiansen M., Dodoo D., Theisen M. (2015). Antibody-Dependent Cellular Inhibition Is Associated with Reduced Risk against Febrile Malaria in a Longitudinal Cohort Study Involving Ghanaian Children. Open Forum. Infect. Dis..

[21] Nagelkerke S.Q., Schmidt D.E., Haas M.d., Kuijpers T.W. (2019). Genetic Variation in Low-To-Medium-Affinity Fcγ Receptors: Functional Consequences, Disease Associations, and Opportunities for Personalized Medicine. Front. Immunol..

[22] Anania J.C., Chenoweth A.M., Wines B.D., Hogarth P.M. (2019). The Human FcγRII (CD32) Family of Leukocyte FcR in Health and Disease. Front. Immunol..

[23] Gillis C., Gouel-Chéron A., Jönsson F., Bruhns P. (2014). Contribution of Human FcγRs to Disease with Evidence from Human Polymorphisms and Transgenic Animal Studies. Front. Immunol..

[24] Schuldt K., Esser C., Evans J., May J., Timmann C., Ehmen C., Loag W., Ansong D., Ziegler A., Agbenyega T. (2010). FCGR2A functional genetic variant associated with susceptibility to severe malarial anaemia in Ghanaian children. J. Med. Genet..

[25] Munde E.O., Okeyo W.A., Raballah E., Anyona S.B., Were T., Ong’echa J.M., Perkins D.J., Ouma C. (2017). Association between Fcγ receptor IIA, IIIA and IIIB genetic polymorphisms and susceptibility to severe malaria anemia in children in western Kenya. BMC Infect. Dis..

[26] Ouma C., Keller C.C., Opondo D.A., Were T., Otieno R.O., Otieno M.F., Orago A.S.S., Ong’Echa J.M., Vulule J.M., Ferrell R.E. (2006). Association of FCgamma receptor IIA (CD32) polymorphism with malarial anemia and high-density parasitemia in infants and young children. Am. J. Trop. Med. Hyg..

[27] Sinha S., Mishra S.K., Sharma S., Patibandla P.K., Mallick P.K., Sharma S.K., Mohanty S., Pati S.S., Mishra S.K., Ramteke B.K. (2008). Polymorphisms of TNF-enhancer and gene for FcgammaRIIa correlate with the severity of falciparum malaria in the ethnically diverse Indian population. Malar. J..

[28] Zhao J., Ma L., Chen S., Xie Y., Xie L., Deng Y., He Y., Li T., Wang J., Li S. (2014). Association between Fc-gamma receptor IIa (CD32) gene polymorphism and malaria susceptibility: A meta-analysis based on 6928 subjects. Infect. Genet. Evol..

[29] Amiah M.A., Ouattara A., Okou D.T., N’Guetta S.-P.A., Yavo W. (2020). Polymorphisms in Fc Gamma Receptors and Susceptibility to Malaria in an Endemic Population. Front. Immunol..

[30] Fall A.K.D.J., Courtin D., Adamou R., Edslev S., Hansen A., Domingo N., Christiansen M., Adu B., Milet J., Garcia A. (2022). Fc Gamma Receptor IIIB NA1/NA2/SH Polymorphisms Are Associated with Malaria Susceptibility and Antibody Levels to *P. falciparum* Merozoite Antigens in Beninese Children. Int. J. Mol. Sci..

[31] Smith K.G.C., Clatworthy M.R. (2010). FcgammaRIIB in autoimmunity and infection: Evolutionary and therapeutic implications. Nat. Rev. Immunol..

[32] Fall A.K.D.J., Kana I.H., Dechavanne C., Garcia-Senosiain A., Guitard E., Milet J., Massougbodji A., Garcia A., Dugoujon J.-M., Migot-Nabias F. (2022). Naturally acquired antibodies from Beninese infants promote *Plasmodium falciparum* merozoite-phagocytosis by human blood leukocytes: Implications for control of asymptomatic malaria infections. Malar. J..

[33] Nasr A., Aljada A., Hamid O., Elsheikh H.A., Masuadi E., Al-Bawab A., Alenazi T.H., Abushouk A., Salah A.M. (2021). Significant differences in FcγRIIa, FcγRIIIa and FcγRIIIb genes polymorphism and anti-malarial IgG subclass pattern are associated with severe *Plasmodium falciparum* malaria in Saudi children. Malar. J..

[34] Cherif M.K., Sanou G.S., Maiga B., Israelsson E., Ouédraogo A.L., Bougouma E.C., Diarra A., Ouédraogo A., Ouattara A.S., Troye-Blomberg M. (2012). FcγRIIa polymorphism and anti-malaria-specific IgG and IgG subclass responses in populations differing in susceptibility to malaria in Burkina Faso. Scand. J. Immunol..

[35] Simpson A.P., Roghanian A., Oldham R.J., Chan H.T.C., Penfold C.A., Kim H.J., Inzhelevskaya T., Mockridge C.I., Cox K.L., Bogdanov Y.D. (2022). FcγRIIB controls antibody-mediated target cell depletion by ITIM-independent mechanisms. Cell Rep..

[36] Xu L., Li G., Wang J., Fan Y., Wan Z., Zhang S., Shaheen S., Li J., Wang L., Yue C. (2014). Through an ITIM-independent mechanism the FcγRIIB blocks B cell activation by disrupting the colocalized microclustering of the B cell receptor and CD19. J. Immunol..

[37] Willcocks L.C., Smith K.G.C., Clatworthy M.R. (2009). Low-affinity Fcgamma receptors, autoimmunity and infection. Expert Rev. Mol. Med..

[38] Clatworthy M.R., Willcocks L., Urban B., Langhorne J., Williams T.N., Peshu N., Watkins N.A., Floto R.A., Smith K.G.C. (2007). Systemic lupus erythematosus-associated defects in the inhibitory receptor FcgammaRIIb reduce susceptibility to malaria. Proc. Natl. Acad. Sci. USA.

[39] Gelabert P., Olalde I., de-Dios T., Civit S., Lalueza-Fox C. (2017). Malaria was a weak selective force in ancient Europeans. Sci. Rep..

[40] Willcocks L.C., Carr E.J., Niederer H.A., Rayner T.F., Williams T.N., Yang W., Scott J.A.G., Urban B.C., Peshu N., Vyse T.J. (2010). A defunctioning polymorphism in FCGR2B is associated with protection against malaria but susceptibility to systemic lupus erythematosus. Proc. Natl. Acad. Sci. USA.

[41] Ebonwu J., Lassaunière R., Paximadis M., Strehlau R., Gray G.E., Kuhn L., Tiemessen C.T. (2022). FCGR3A gene duplication, FcγRIIb-232TT and FcγRIIIb-HNA1a associate with an increased risk of vertical acquisition of HIV-1. PLoS ONE.

[42] Hu W., Zhang Y., Sun X., Zhang T., Xu L., Xie H., Li Z., Liu W., Lou J., Chen W. (2019). FcγRIIB-I232T polymorphic change allosterically suppresses ligand binding. Elife.

[43] (2013). GTEx Consortium The Genotype-Tissue Expression (GTEx) project. Nat. Genet..

[44] Lu H., Ma L., Quan C., Li L., Lu Y., Zhou G., Zhang C. (2023). RegVar: Tissue-specific Prioritization of Noncoding Regulatory Variants. Genom. Proteom. Bioinform..

[45] Ota M., Nagafuchi Y., Hatano H., Ishigaki K., Terao C., Takeshima Y., Yanaoka H., Kobayashi S., Okubo M., Shirai H. (2021). Dynamic landscape of immune cell-specific gene regulation in immune-mediated diseases. Cell.

[46] Kerimov N., Hayhurst J.D., Peikova K., Manning J.R., Walter P., Kolberg L., Samoviča M., Sakthivel M.P., Kuzmin I., Trevanion S.J. (2021). A compendium of uniformly processed human gene expression and splicing quantitative trait loci. Nat. Genet..

[47] Calderon D., Nguyen M.L.T., Mezger A., Kathiria A., Müller F., Nguyen V., Lescano N., Wu B., Trombetta J., Ribado J.V. (2019). Landscape of stimulation-responsive chromatin across diverse human immune cells. Nat. Genet..

[48] Hammal F., de Langen P., Bergon A., Lopez F., Ballester B. (2022). ReMap 2022: A database of Human, Mouse, Drosophila and Arabidopsis regulatory regions from an integrative analysis of DNA-binding sequencing experiments. Nucleic Acids Res..

[49] Sloan C.A., Chan E.T., Davidson J.M., Malladi V.S., Strattan J.S., Hitz B.C., Gabdank I., Narayanan A.K., Ho M., Lee B.T. (2016). ENCODE data at the ENCODE portal. Nucleic Acids Res..

[50] Lassaunière R., Tiemessen C.T. (2016). Variability at the FCGR locus: Characterization in Black South Africans and evidence for ethnic variation in and out of Africa. Genes. Immun..

[51] Tai K.Y., Dhaliwal J., Balasubramaniam V. (2022). Leveraging Mann-Whitney U test on large-scale genetic variation data for analysing malaria genetic markers. Malar. J..

[52] Cherif M.K., Sanou G.S., Bougouma E.C., Diarra A., Ouédraogo A., Dolo A., Troye-Blomberg M., Cavanagh D.R., Theisen M., Modiano D. (2015). Is Fc gamma receptor IIA (FcγRIIA) polymorphism associated with clinical malaria and *Plasmodium falciparum* specific antibody levels in children from Burkina Faso?. Acta Trop..

[53] Dwomoh D., Adu B., Dodoo D., Theisen M., Iddi S., Gerds T.A. (2020). Evaluating the predictive performance of malaria antibodies and FCGR3B gene polymorphisms on *Plasmodium falciparum* infection outcome: A prospective cohort study. Malar. J..

[54] Adu B., Jepsen M.P.G., Gerds T.A., Kyei-Baafour E., Christiansen M., Dodoo D., Theisen M. (2014). Fc gamma receptor 3B (FCGR3B-c.233C>A-rs5030738) polymorphism modifies the protective effect of malaria specific antibodies in Ghanaian children. J. Infect. Dis..

[55] Fall A.K.D.J., Dechavanne C., Sabbagh A., Garcia A., Courtin D., Migot-Nabias F. (2023). Combined polymorphisms involving the IgG heavy chain and Fc gamma receptors among Fulani and non-Fulani in Benin: Implications for the natural protection of young Fulani against *Plasmodium falciparum* malaria infections. Infect. Genet. Evol..

[56] Atkinson A., Garnier S., Afridi S., Fumoux F., Rihet P. (2012). Genetic variations in genes involved in heparan sulphate biosynthesis are associated with *Plasmodium falciparum* parasitaemia: A familial study in Burkina Faso. Malar. J..

[57] Rihet P., Abel L., Traoré Y., Traoré-Leroux T., Aucan C., Fumoux F. (1998). Human malaria: Segregation analysis of blood infection levels in a suburban area and a rural area in Burkina Faso. Genet. Epidemiol..

[58] Machiela M.J., Chanock S.J. (2015). LDlink: A web-based application for exploring population-specific haplotype structure and linking correlated alleles of possible functional variants. Bioinformatics.

[59] Abecasis G.R., Cardon L.R., Cookson W.O. (2000). A general test of association for quantitative traits in nuclear families. Am. J. Hum. Genet..

