# Supplementary material for: A Non-Coding Fc Gamma Receptor Cis-Regulatory Variant within the 1q23 Gene Cluster Is Associated with Plasmodium falciparum Infection in Children Residing in Burkina Faso
Source: Int J Mol Sci. 2023 Oct 28;24(21):15711. doi: 10.3390/ijms242115711 (PMC10650193; doi:10.3390/ijms242115711)
Supplement: Supplementary file 1 [file ijms-24-15711-s001.zip › ijms-2572405Revised_supTablesandFigures.pdf]

**Supplemental Figure S1** Epigenetic landscape of the *FCGR* locus in B and Natural Killer (NK) cells

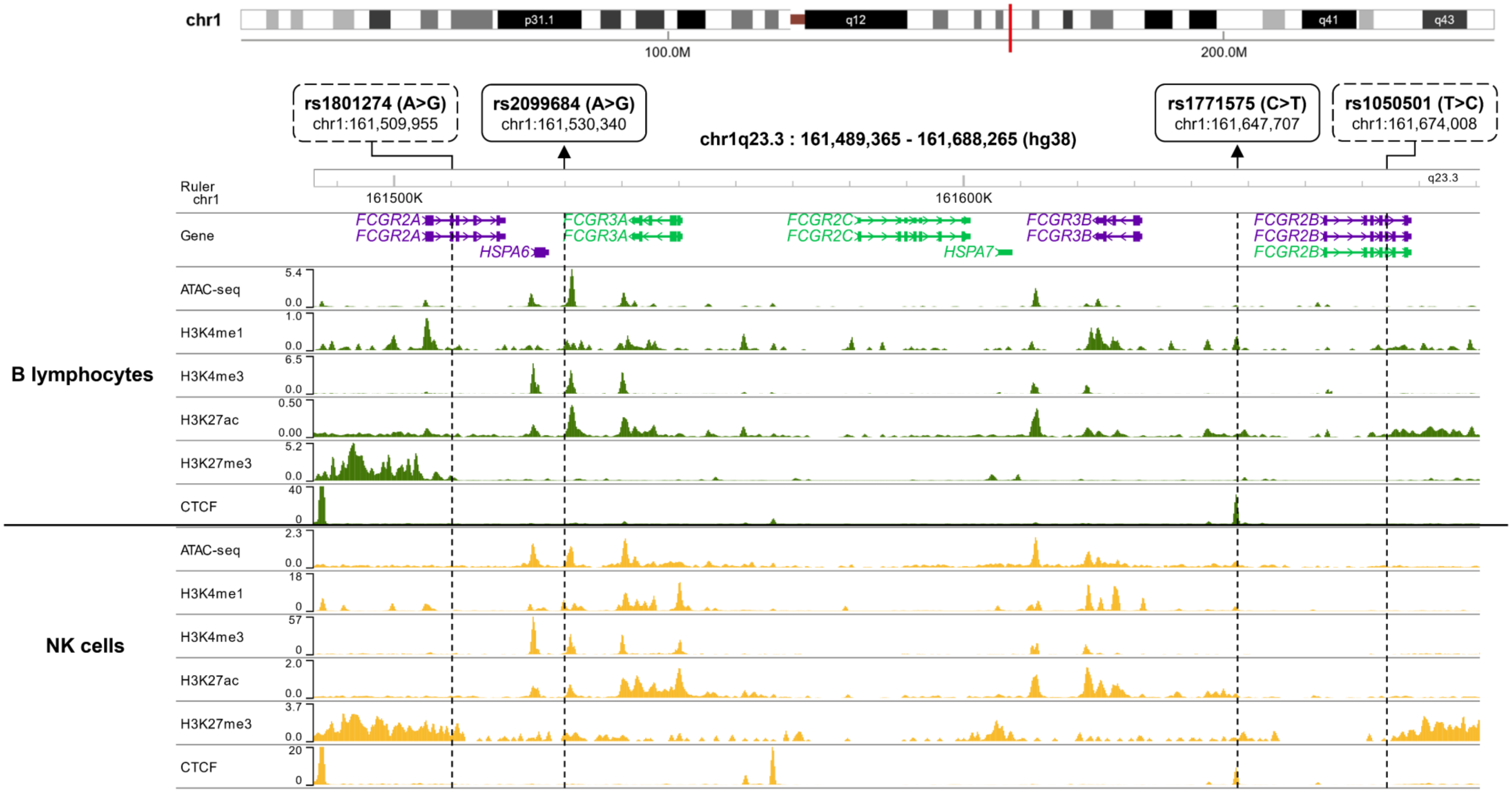


ATAC-seq, CTCF, ChIP-seq peaks and histone modifications associated with active (H3K4me1, H3K4me3, H3K27ac) or repressive (H3K27me3) regulatory regions are represented. Both rs2099684 and rs1771575 are located in enhancer regions active in both cell type. rs1771575 is also found within a CTCF binding site. No regulatory region seems to overlap with coding variants rs1801274 and rs1050501.

**Supplemental Figure S2.**

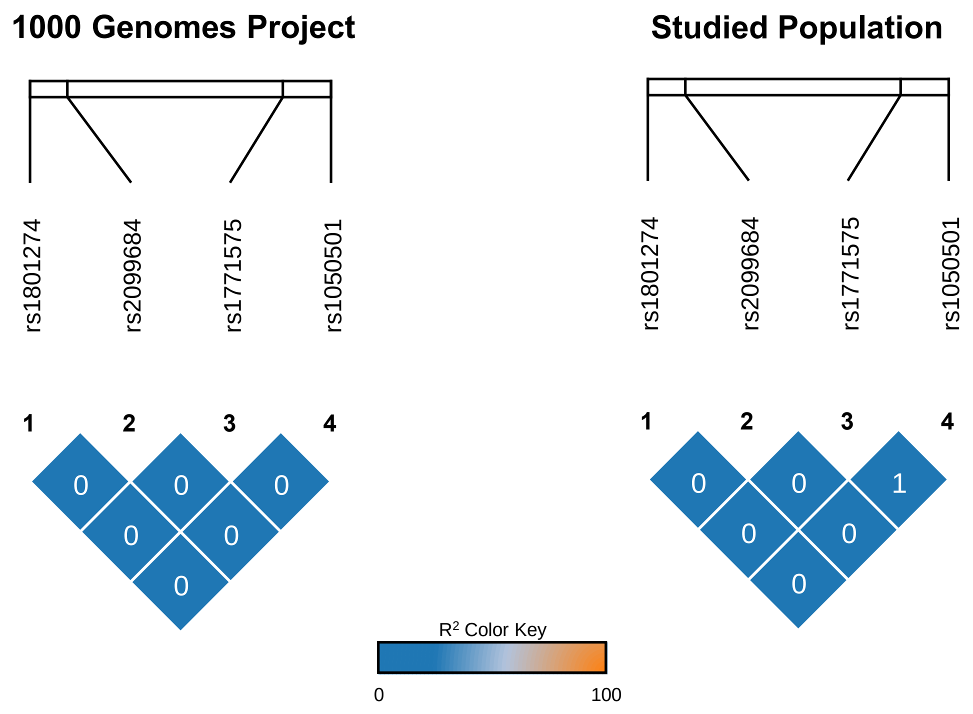


Linkage Disequilibrium (LD) plot generated with Haploview 4.2.
No linkage disequilibrium was observed between rs1801274, rs2099684, rs1771575 and rs1050501 in the African population. Pairwise LD statistics (100*R-squared values) are displayed within each square

**Supplemental Table S1**

**Selection of 773 Whole Blood eQTL for *FCGR2A, FCGR2B, FCGR2C, FCGR3A*, and *FCGR3B* Target Genes using the GTEx Database.** (see attached pdf document **SupTable1.pdf)** The Supplemental Table contains information on 773 whole blood expression quantitative trait loci (eQTL) related to the target genes *FCGR2A*, *FCGR2B*, *FCGR2C*, *FCGR3A*, and *FCGR3B.* These eQTL were selected from the GTEx (Genotype-Tissue Expression) database. Each eQTL is classified based on the RegVar score and has a unique rsID (rs number) to identify the regulatory variant SNP. Additionally. the table provides the position of the regulatory variant SNP and its distance to the respective

**Supplemental Table S2:** The RegVar tool was used to prioritize the eQTL for *FCGR2A*, *FCGR2B*, *FCGR2C*, *FCGR3A*, and *FCGR3B* target genes selected by querying the GTEX (V8). The top 20 SNPs with the best scores RegVar were selected. The eQTL annotations of these 20 selected SNPs were checked in two independent immune cell eQTL databases. ImmuNexUT and ebi eQTL catalog to retain 20 SNPs that had annotations in both databases, Code 1.

| **SNP ID** | **max RegVar score** | ***FCGR2A*** | ***FCGR2B*** | ***FCGR2C*** | ***FCGR3A*** | ***FCGR3B*** | **ImmuNexUT** | **EBI eQTL immune cells** | **ATAC-seq immune cells** | **ReMap immune cells** |
|---|---|---|---|---|---|---|---|---|---|---|
| **rs35740080** | 0.8968 | 0.8968 | 0.73237 | 0.7964 | 0.82888 | 0.6698 | 1 | 0 | 1 | 1 |
| **rs1771575** | 0.88821 | 0.58371 | 0.87185 | 0.88821 | 0.65117 | 0.85067 | 1 | 1 | 1 | 1 |
| **rs426615** | 0.86432 | 0.86432 | 0.75872 | 0.83191 | 0.84602 | 0.6976 | 0 | 1 | 0 | 0 |
| **rs404508** | 0.85288 | 0.85288 | 0.80169 | 0.81282 | 0.75244 | 0.65032 | 1 | 0 | 0 | 0 |
| **rs332398** | 0.84701 | 0.83198 | 0.79963 | 0.84701 | 0.81765 | 0.73929 | 1 | 0 | 1 | 1 |
| **rs35181127** | 0.84625 | 0.74752 | 0.80378 | 0.84625 | 0.66597 | 0.77535 | 1 | 1 | 0 | 0 |
| **rs34416533** | 0.84503 | 0.84158 | 0.82399 | 0.84503 | 0.70214 | 0.77021 | 1 | 1 | 0 | 0 |
| **rs34881159** | 0.84172 | 0.67048 | 0.76736 | 0.84172 | 0.75198 | 0.82406 | 1 | 1 | 0 | 0 |
| **rs35276103** | 0.83382 | 0.7532 | 0.77598 | 0.83382 | 0.75558 | 0.76315 | 1 | 1 | 0 | 0 |
| **rs35369398** | 0.83335 | 0.71495 | 0.82899 | 0.83335 | 0.69597 | 0.82707 | 1 | 0 | 1 | 1 |
| **rs3754053** | 0.82848 | 0.53129 | 0.70669 | 0.82848 | 0.72965 | 0.76918 | 1 | 1 | 1 | 0 |
| **rs1801274** | 0.81588 | 0.81588 | 0.66316 | 0.73976 | 0.64737 | 0.56181 | 0 | 0 | 0 | 0 |
| **rs368433** | 0.79912 | 0.79912 | 0.51332 | 0.60139 | 0.49414 | 0.36326 | 1 | 1 | 0 | 0 |
| **rs35428265** | 0.79284 | 0.6229 | 0.78334 | 0.79284 | 0.62292 | 0.73205 | 1 | 0 | 0 | 0 |
| **rs34182452** | 0.78667 | 0.55497 | 0.67691 | 0.76793 | 0.42453 | 0.78667 | 0 | 1 | 0 | 0 |
| **rs1771588** | 0.78402 | 0.69921 | 0.70211 | 0.78402 | 0.57932 | 0.76763 | 0 | 0 | 0 | 0 |
| **rs36063867** | 0.77923 | 0.64142 | 0.77542 | 0.77923 | 0.65388 | 0.76456 | 1 | 1 | 0 | 0 |
| **rs2099684** | 0.77718 | 0.77718 | 0.58357 | 0.64064 | 0.61372 | 0.48393 | 1 | 1 | 1 | 1 |
| **rs36117182** | 0.77567 | 0.70774 | 0.77567 | 0.75009 | 0.62747 | 0.73007 | 1 | 1 | 0 | 0 |
| **rs35674644** | 0.77337 | 0.49578 | 0.69457 | 0.77337 | 0.59949 | 0.71395 | 1 | 1 | 0 | 0 |

**Supplemental Table S3 Files used for visualization in WashU Epigenome Browser (http://epigenomegateway.wustl.edu/browser/)**

| Factor | Cell Type | GEO/ENCODE | Track Type |
|---|---|---|---|
| ATAC-seq | B Lymphocyte | GSM1832754 | bigwig |
| DNase-seq | B Lymphocyte | GSM1024765 | bigwig |
| H3K4me1 | B Lymphocyte | GSM971340 | bigwig |
| H3K4me3 | B Lymphocyte | GSM945229 | bigwig |
| H3K27ac | B Lymphocyte | GSM1003459 | bigwig |
| H3K27me3 | B Lymphocyte | ENCFF618YNB | bigwig |
| CTCF | B Lymphocyte | ENCFF326SVX | bigwig |
| ATAC-seq | Natural Killer Cell | GSM3320412 | bigwig |
| DNase-seq | Natural Killer Cell | GSM665820 | bigwig |
| H3K4me1 | Natural Killer Cell | ENCFF647IBZ | bigwig |
| H3K4me3 | Natural Killer Cell | ENCFF977TEF | bigwig |
| H3K27ac | Natural Killer Cell | GSM999009 | bigwig |
| H3K27me3 | Natural Killer Cell | ENCFF936TIC | bigwig |
| CTCF | Natural Killer Cell | ENCFF395HSD | bigwig |
| ATAC-seq | Monocyte | GSM2325687 | bigwig |
| DNase-seq | Monocyte | GSM1024791 | bigwig |
| H3K4me1 | Monocyte | GSM1003535 | bigwig |
| H3K4me3 | Monocyte | GSM945225 | bigwig |
| H3K27ac | Monocyte | GSM1003559 | bigwig |
| H3K27me3 | Monocyte | GSM1003564 | bigwig |
| CTCF | Monocyte | GSM1003508 | bigwig |
| ATAC-seq | Neutrophil | GSM2083799 | bigwig |
| DNase-seq | Neutrophil | ENCFF217SFX | bigwig |
| H3K4me1 | Neutrophil | ENCFF490RNA | bigwig |
| H3K4me3 | Neutrophil | GSM3612227 | bigwig |
| H3K27ac | Neutrophil | GSM3612222 | bigwig |
| H3K27me3 | Neutrophil | ENCFF138FFB | bigwig |
| CTCF | Neutrophil | GSM3612219 | bigwig |
| RAD21 | GM12878 | ECNFF821OON | bigwig |
| RAD21 | GM12878 | ENCFF867VSB | bigbed |
| SMC3 | GM12878 | ENCFF054NUV | bigwig |
| SMC3 | GM12878 | ENCFF085RLZ | bigbed |
| MAX | K562 | ENCFF570UXV | bigwig |
| MAX | K562 | ENCFF524IJO | bigbed |
| MYC | K562 | ENCFF140BXD | bigwig |
| MYC | K562 | ENCFF988ZRU | bigbed |

Tracks were imported from Cistrome Data Browser (http://cistrome.org/db/#/) or ENCODE Data Portal (https://www.encodeproject.org/) using the ‘Add Remote Track’ feature in WashU Epigenome Browser. Files details are available in NCBI Gene Expression Omnibus (GEO) or ENCODE Portal.

**Supplemental Table S4: RSAT prediction of transcription factors binding sites altered by rs1771575 and rs2099684**

**RSAT results for rs1771575**

| TF | ac_motif | best_w | worst_w | w_diff | best_pval | worst_pval | pval_ratio | best_variant | worst_variant | best_seq | worst_seq |
|---|---|---|---|---|---|---|---|---|---|---|---|
| SOX2 | SOX2_1 | 11.41 | 6.23 | 5.18 | 8.30E-07 | 2.20E-04 | 265.06 | T | C | CCTTTGTCCTGCAGA | CCTTCGTCCTGCAGA |
| NR3C1 | NR3C1_known5 | 6.03 | -7.85 | 13.89 | 6.40E-04 | 7.50E-02 | 117.19 | T | C | TTTGTCCT | TTCGTCCT |
| SOX15 | MA1152.1 | 6.27 | 1.47 | 4.8 | 4.50E-04 | 8.30E-03 | 18.44 | T | C | CCTTTGTCCT | CCTTCGTCCT |
| SOX10 | SOX10_1 | 6.45 | 5.28 | 1.17 | 8.00E-05 | 1.00E-03 | 12.5 | C | T | CTTCGTC | CTTTGTC |
| POU5F1 | POU5F1_known3 | 7.9 | 4.57 | 3.33 | 4.10E-05 | 5.10E-04 | 12.44 | T | C | CTTTGTCCTGCAGAA | CTTCGTCCTGCAGAA |
| ZNF24 | MA1124.1 | 3.76 | -0.87 | 4.63 | 3.80E-04 | 4.00E-03 | 10.53 | C | T | GATTCCTTCGTCC | GATTCCTTTGTCC |

**RSAT results for rs2099684**

| TF name | ac_motif | best_w | worst_w | w_diff | best_pval | worst_pval | pval_ratio | best_variant | worst_variant | best_seq | worst_seq |
|---|---|---|---|---|---|---|---|---|---|---|---|
| CLOCK | CLOCK_1 | 8.79 | -6.06 | 14.85 | 2.30E-05 | 4.00E-02 | 1739.13 | G | A | AACACATGTT | AACACATATT |
| MYC | MYC_known4 | 6.87 | -8 | 14.87 | 1.60E-04 | 1.40E-01 | 875 | G | A | AAAACACATGTTGT | AAAACACATATTGT |
| MYC | MYC_known21 | 6.11 | -8.76 | 14.87 | 1.30E-04 | 5.30E-02 | 407.69 | G | A | AACACATGTT | AACACATATT |
| ATOH1 | ATOH1_1 | 5.37 | -10.09 | 15.46 | 4.40E-04 | 1.20E-01 | 272.73 | A | G | CACATATTGT | CACATGTTGT |
| MYC | MYC_known22 | 6.33 | -8.54 | 14.88 | 1.70E-04 | 4.20E-02 | 247.06 | G | A | AACACATGTT | AACACATATT |
| BHLHE23 | BHLHE23_1 | 5.31 | -9.56 | 14.87 | 2.50E-04 | 4.50E-02 | 180 | G | A | AACACATGTT | AACACATATT |
| TFAP4 | MA1570.1 | 9.01 | 1.77 | 7.24 | 1.40E-05 | 2.40E-03 | 171.43 | G | A | AACACATGTT | AACACATATT |
| MYC | MYC_known18 | 4.19 | -10.64 | 14.83 | 7.40E-04 | 1.20E-01 | 162.16 | G | A | AACACATGTT | AACACATATT |
| OLIG1 | OLIG1_1 | 5.2 | -9.68 | 14.88 | 1.60E-04 | 2.50E-02 | 156.25 | G | A | AACACATGTT | AACACATATT |
| TFE3 | TFE3_1 | 4.89 | -9.98 | 14.87 | 8.00E-04 | 1.20E-01 | 150 | G | A | AACACATGTT | AACACATATT |
| MYF6 | MYF6_2 | 6.82 | -8.06 | 14.88 | 9.60E-05 | 1.30E-02 | 135.42 | G | A | AACACATGTT | AACACATATT |

| | | | | | | | | | | | |
|---|---|---|---|---|---|---|---|---|---|---|---|
| NEUROD2 | NEUROD2_1 | 6.82 | -8 | 14.81 | 1.80E-04 | 2.30E-02 | 127.78 | G | A | AACACATGTT | AACACATATT |
| ATOH7 | MA1468.1 | 8.2 | 0.57 | 7.63 | 5.00E-05 | 5.90E-03 | 118 | G | A | AACACATGTT | AACACATATT |
| MAX | MA0058.3 | 6.11 | -3.79 | 9.9 | 1.30E-04 | 1.50E-02 | 115.38 | G | A | AACACATGTT | AACACATATT |
| CLOCK | MA0819.1 | 8.78 | 1.66 | 7.13 | 2.30E-05 | 2.60E-03 | 113.04 | G | A | AACACATGTT | AACACATATT |
| MLXIPL | MLXIPL_1 | 5.27 | -9.61 | 14.88 | 1.80E-04 | 2.00E-02 | 111.11 | G | A | AACACATGTT | AACACATATT |
| BHLHE22 | BHLHE22_1 | 4.88 | -10 | 14.88 | 1.40E-04 | 1.50E-02 | 107.14 | G | A | AAACACATGTTG | AAACACATATTG |
| NEUROG2 | NEUROG2_2 | 6.5 | -8.38 | 14.88 | 8.10E-05 | 7.60E-03 | 93.83 | G | A | AACACATGTT | AACACATATT |
| MNT | MNT_1 | 4.81 | -10.03 | 14.85 | 4.80E-04 | 3.70E-02 | 77.08 | G | A | AACACATGTT | AACACATATT |
| OLIG2 | OLIG2_2 | 9.47 | 4.69 | 4.78 | 1.10E-05 | 8.30E-04 | 75.45 | G | A | AACACATGTT | AACACATATT |
| MESP1 | MESP1_1 | 5.65 | -9.08 | 14.73 | 6.20E-04 | 4.00E-02 | 64.52 | G | A | AACACATGTT | AACACATATT |
| NEUROD2 | MA0668.1 | 6.81 | -1.62 | 8.44 | 1.80E-04 | 1.10E-02 | 61.11 | G | A | AACACATGTT | AACACATATT |
| ZBTB18 | ZBTB18_2 | 4.71 | -10.17 | 14.88 | 1.40E-04 | 8.10E-03 | 57.86 | G | A | AAAACACATGTTG | AAAACACATATTG |
| OLIG1 | MA0826.1 | 5.21 | -5.2 | 10.41 | 1.60E-04 | 9.20E-03 | 57.5 | G | A | AACACATGTT | AACACATATT |
| BHLHA15 | BHLHA15_1 | 2.93 | -12.6 | 15.53 | 3.90E-04 | 2.20E-02 | 56.41 | A | G | CACATATTGT | CACATGTTGT |
| TFEC | TFEC_1 | 5.24 | -9.64 | 14.88 | 5.90E-04 | 3.00E-02 | 50.85 | G | A | AACACATGTT | AACACATATT |
| MLXIPL | MA0664.1 | 5.27 | -2.38 | 7.66 | 1.80E-04 | 7.10E-03 | 39.44 | G | A | AACACATGTT | AACACATATT |
| CLOCK | MA0819.1 | 8.36 | 3.34 | 5.02 | 2.90E-05 | 1.10E-03 | 37.93 | G | A | AACATGTGTT | AATATGTGTT |
| CLOCK | CLOCK_1 | 8.36 | 3.22 | 5.14 | 3.00E-05 | 1.10E-03 | 36.67 | G | A | AACATGTGTT | AATATGTGTT |
| NEUROG1 | MA0623.2 | 7.52 | -1.71 | 9.23 | 5.80E-05 | 2.00E-03 | 34.48 | G | A | AACACATGTT | AACACATATT |
| MSGN1 | MA1524.1 | 5.22 | -4.64 | 9.85 | 9.00E-04 | 3.10E-02 | 34.44 | G | A | CAACATGTGTTT | CAATATGTGTTT |
| MYF6 | MA0667.1 | 6.91 | -0.28 | 7.19 | 9.00E-05 | 3.10E-03 | 34.44 | G | A | AACATGTGTT | AATATGTGTT |
| BHLHE23 | MA0817.1 | 4.94 | -4.64 | 9.58 | 1.40E-04 | 4.80E-03 | 34.29 | G | A | AAACACATGTTG | AAACACATATTG |
| OLIG2 | OLIG2_1 | 6.7 | 0.19 | 6.52 | 1.40E-04 | 4.80E-03 | 34.29 | A | G | CACATATTGT | CACATGTTGT |
| NEUROG1 | MA0623.2 | 7.1 | -2.16 | 9.26 | 7.20E-05 | 2.40E-03 | 33.33 | G | A | AACATGTGTT | AATATGTGTT |
| MYF6 | MA0667.1 | 6.83 | -0.33 | 7.16 | 9.70E-05 | 3.20E-03 | 32.99 | G | A | AACACATGTT | AACACATATT |
| NEUROD2 | MA0668.1 | 5.33 | -3.13 | 8.46 | 5.20E-04 | 1.70E-02 | 32.69 | G | A | AACATGTGTT | AATATGTGTT |
| BHLHE22 | MA0818.1 | 5.41 | -1.91 | 7.32 | 2.40E-04 | 7.40E-03 | 30.83 | G | A | AACACATGTT | AACACATATT |
| TFEB | MA0692.1 | 4.44 | -3.22 | 7.66 | 7.20E-04 | 2.20E-02 | 30.56 | G | A | AACATGTGTT | AATATGTGTT |
| TFEB | TFEB_1 | 4.44 | -3.3 | 7.75 | 7.20E-04 | 2.20E-02 | 30.56 | G | A | AACATGTGTT | AATATGTGTT |

| | | | | | | | | | | | |
|---|---|---|---|---|---|---|---|---|---|---|---|
| SCRT1 | SCRT1_1 | 1.93 | -12.98 | 14.91 | 2.50E-04 | 7.60E-03 | 30.4 | G | A | TGACAACATGTG TTT | TGACAATATGTGTTT |
| MYC | MYC_know n3 | 4.71 | -10.17 | 14.88 | 3.20E-04 | 9.70E-03 | 30.31 | G | A | AAAACACATGTT GT | AAAACACATATTGT |
| TFEB | MA0692.1 | 5.39 | -1.16 | 6.55 | 3.70E-04 | 1.10E-02 | 29.73 | G | A | AACACATGTT | AACACATATT |
| TFEB | TFEB_1 | 5.39 | -1.18 | 6.57 | 3.70E-04 | 1.10E-02 | 29.73 | G | A | AACACATGTT | AACACATATT |
| TFAP4 | TFAP4_6 | 2.92 | -11.96 | 14.88 | 6.80E-04 | 2.00E-02 | 29.41 | G | A | AACACATGTT | AACACATATT |
| OLIG3 | OLIG3_1 | 6.2 | 0.33 | 5.87 | 2.40E-04 | 6.80E-03 | 28.33 | G | A | AACACATGTT | AACACATATT |
| OLIG3 | MA0827.1 | 6.21 | 0.4 | 5.81 | 2.40E-04 | 6.60E-03 | 27.5 | G | A | AACACATGTT | AACACATATT |
| MNT | MA0825.1 | 4.82 | -2.77 | 7.59 | 4.80E-04 | 1.30E-02 | 27.08 | G | A | AACACATGTT | AACACATATT |
| NEUROG2 | MA0669.1 | 6.53 | -0.59 | 7.12 | 8.50E-05 | 2.30E-03 | 27.06 | G | A | AACACATGTT | AACACATATT |
| NEUROG2 | NEUROG2 _1 | 6.58 | -0.28 | 6.86 | 1.00E-04 | 2.60E-03 | 26 | G | A | AACACATGTT | AACACATATT |
| OLIG3 | MA0827.1 | 6.55 | 1.11 | 5.44 | 1.90E-04 | 4.80E-03 | 25.26 | A | G | CACATATTGT | CACATGTTGT |
| OLIG3 | OLIG3_1 | 6.55 | 1.09 | 5.46 | 1.90E-04 | 4.80E-03 | 25.26 | A | G | CACATATTGT | CACATGTTGT |
| ZBTB18 | ZBTB18_3 | 4.97 | -3.16 | 8.12 | 2.00E-04 | 5.00E-03 | 25 | G | A | AAAACACATGTT G | AAAACACATATTG |
| MYCN | MYCN_1 | 4.53 | -10.35 | 14.88 | 5.30E-04 | 1.30E-02 | 24.53 | G | A | AAACACATGTTG | AAACACATATTG |
| ZBTB18 | MA0698.1 | 4.97 | -3.07 | 8.04 | 2.00E-04 | 4.90E-03 | 24.5 | G | A | AAAACACATGTT G | AAAACACATATTG |
| ATOH1 | ATOH1_1 | 5.73 | -0.21 | 5.94 | 3.60E-04 | 8.60E-03 | 23.89 | G | A | AACACATGTT | AACACATATT |
| E2F | E2F_disc1 | 4.04 | -10.84 | 14.88 | 2.10E-04 | 4.70E-03 | 22.38 | G | A | CATGTTGTCA | CATATTGTCA |
| MYC | MYC_know n16 | 4.58 | -10.3 | 14.88 | 2.10E-04 | 4.70E-03 | 22.38 | G | A | AAACACATGTT | AAACACATATT |
| TAL1 | TAL1_kno wn5 | 5.13 | -9.73 | 14.86 | 4.60E-04 | 1.00E-02 | 21.74 | G | A | AAAACACATGTT | AAAACACATATT |
| CDX2 | CDX2_1 | 6.55 | -8.99 | 15.53 | 1.60E-04 | 3.40E-03 | 21.25 | A | G | AAACACATATTG TC | AAACACATGTTGTC |
| MYC | MYC_know n7 | 5.12 | -9.76 | 14.88 | 1.00E-03 | 2.10E-02 | 21 | G | A | ACACATGT | ACACATAT |
| OLIG2 | OLIG2_1 | 5.87 | -0.05 | 5.91 | 2.60E-04 | 5.40E-03 | 20.77 | G | A | AACACATGTT | AACACATATT |
| MXI1 | MXI1_kno wn1 | 5.57 | 1.16 | 4.42 | 5.80E-04 | 1.10E-02 | 18.97 | G | A | ACACATGTTG | ACACATATTG |
| CUX1 | CUX1_4 | 1.29 | -14.24 | 15.53 | 8.90E-04 | 1.60E-02 | 17.98 | A | G | GGAAAACACAT ATTG | GGAAAACACATGTTG |
| TFE3 | MA0831.2 | 4.4 | -1.98 | 6.38 | 7.80E-04 | 1.40E-02 | 17.95 | G | A | CACATGTT | CACATATT |

| | | | | | | | | | | | |
|---|---|---|---|---|---|---|---|---|---|---|---|
| TAL1::TCF3 | MA0091.1 | 6.52 | 1.69 | 4.83 | 1.50E-04 | 2.50E-03 | 16.67 | G | A | AAAACACATGTT | AAAACACATATT |
| MYC | MYC_known17 | 4.58 | -10.3 | 14.88 | 5.70E-04 | 9.40E-03 | 16.49 | G | A | CACATGT | CACATAT |
| MYC | MYC_known19 | 4.58 | -0.74 | 5.32 | 4.70E-04 | 7.70E-03 | 16.38 | G | A | AAAACACATGTTGTCA | AAAACACATATTGTCA |
| TFAP4 | MA0691.1 | 2.93 | -6.27 | 9.2 | 6.80E-04 | 1.10E-02 | 16.18 | G | A | AACACATGTT | AACACATATT |
| MSC | MA0665.1 | 5.23 | -0.07 | 5.3 | 3.10E-04 | 4.40E-03 | 14.19 | G | A | AACACATGTT | AACACATATT |
| SREBF2 | MA0828.1 | 1.28 | -5.52 | 6.8 | 6.70E-04 | 9.40E-03 | 14.03 | G | A | AACACATGTT | AACACATATT |
| MA0826 | MA0826.1 | 3.24 | -3.56 | 6.79 | 4.10E-04 | 5.70E-03 | 13.9 | A | G | CACATATTGT | CACATGTTGT |
| NHLH1 | NHLH1_2 | 1.35 | -13.54 | 14.88 | 8.70E-04 | 1.20E-02 | 13.79 | G | A | ACGGAAAACACATGTTGTCAGG | ACGGAAAACACATATTGTCAGG |
| SREBP | SREBP_known5 | 1.27 | -5.62 | 6.88 | 6.50E-04 | 8.90E-03 | 13.69 | G | A | AACACATGTT | AACACATATT |
| TCF21 | MA1568.1 | 4.96 | -0.06 | 5.02 | 5.00E-04 | 6.70E-03 | 13.4 | G | A | AAACACATGTTG | AAACACATATTG |
| BHLHE23 | MA0817.1 | 1.71 | -5.93 | 7.64 | 5.40E-04 | 7.10E-03 | 13.15 | A | G | ACACATATTGTC | ACACATGTTGTC |
| OLIG1 | OLIG1_1 | 3.23 | -3.57 | 6.81 | 4.10E-04 | 5.30E-03 | 12.93 | A | G | CACATATTGT | CACATGTTGT |
| MAX::MYC | MA0059.1 | 4.79 | 0.66 | 4.12 | 4.10E-04 | 5.10E-03 | 12.44 | G | A | AAACACATGTT | AAACACATATT |
| OLIG2 | OLIG2_2 | 4.58 | -0.21 | 4.79 | 8.90E-04 | 1.10E-02 | 12.36 | A | G | CACATATTGT | CACATGTTGT |
| SREBP | SREBP_known6 | 1.85 | -5.3 | 7.15 | 6.90E-04 | 8.50E-03 | 12.32 | G | A | AACACATGTT | AACACATATT |
| SNAI2 | MA0745.2 | 4.07 | -1.16 | 5.23 | 6.20E-04 | 7.40E-03 | 11.94 | G | A | AAACACATGTTGT | AAACACATATTGT |
| OLIG2 | MA0678.1 | 5.9 | 1.12 | 4.78 | 2.10E-04 | 2.50E-03 | 11.9 | A | G | CACATATTGT | CACATGTTGT |
| BHLHE22 | MA0818.1 | 2.91 | -2.83 | 5.75 | 9.30E-04 | 1.10E-02 | 11.83 | A | G | CACATATTGT | CACATGTTGT |
| NEUROG2 | MA1642.1 | 4.56 | 0.08 | 4.48 | 8.50E-04 | 9.90E-03 | 11.65 | G | A | AAAACACATGTTG | AAAACACATATTG |
| TWIST1 | MA1123.2 | 5.56 | 1.64 | 3.92 | 4.60E-04 | 5.20E-03 | 11.3 | G | A | AAAACACATGTTG | AAAACACATATTG |
| BHLHE22 | BHLHE22_1 | 1.67 | -6.05 | 7.72 | 5.20E-04 | 5.80E-03 | 11.15 | A | G | ACACATATTGTC | ACACATGTTGTC |
| BPTF | BPTF_1 | 9.07 | 7.52 | 1.55 | 5.60E-06 | 6.00E-05 | 10.71 | A | G | ACGGAAAACACATA | ACGGAAAACACATG |
| FOXA3 | FOXA_known3 | 6.08 | 2.97 | 3.1 | 3.80E-04 | 3.80E-03 | 10 | A | G | GAAAACACATATT | GAAAACACATGTT |

RSAT predicts 6 and 88 transcription factors binding sites altered by rs1771575 and rs2099684, respectively. Binding profiles from ‘JASPAR core nonredundant vertebrates’ and ‘ENCODE (Human TFs)’ were used to scan variant sequences, ac_motif : transcription factors ID from JASPAR or ENCODE; best_w : Best weight for the putative site; worst_w : Best weight for the putative site; w_diff: Difference between best and worst weight; best_pval: P_value of the best putative site; worst_pval: P_value of the worst putative site; pval_ratio: Ratio between worst and best pval ( pval_ratio = worst_pval/best_pval); best_variant: Variant in the best putative site; worst_variant : Variant in the worst putative site; best_seq: Sequence of the best putative site; worst_seq: Sequence of the worst putative site.
