# Supplementary material for: A Non-Coding Fc Gamma Receptor Cis-Regulatory Variant within the 1q23 Gene Cluster Is Associated with Plasmodium falciparum Infection in Children Residing in Burkina Faso
Source: Int J Mol Sci. 2023 Oct 28;24(21):15711. doi: 10.3390/ijms242115711 (PMC10650193; doi:10.3390/ijms242115711)
Supplement: Supplementary file 1 [file ijms-24-15711-s001.zip › SupTableS1.pdf]

SNP ID	**SNP Position**	**Gene Symbol**
rs35740080	chr1:161494310	FCGR2A
rs1771575	chr1:161617496	FCGR2C
rs1771575	chr1:161617496	FCGR2B
rs426615	chr1:161513376	FCGR2A
rs404508	chr1:161495884	FCGR2A
rs1771575	chr1:161617496	FCGR3B
rs332398	chr1:161536130	FCGR2C
rs35181127	chr1:161590742	FCGR2C
rs426615	chr1:161513376	FCGR3A
rs34416533	chr1:161590692	FCGR2C
rs34881159	chr1:161590502	FCGR2C
rs34416533	chr1:161590692	FCGR2A
rs35276103	chr1:161592212	FCGR2C
rs35369398	chr1:161591547	FCGR2C
rs332398	chr1:161536130	FCGR2A
rs426615	chr1:161513376	FCGR2C
rs35369398	chr1:161591547	FCGR2B
rs35740080	chr1:161494310	FCGR3A
rs3754053	chr1:161575843	FCGR2C
rs35369398	chr1:161591547	FCGR3B
rs34881159	chr1:161590502	FCGR3B
rs34416533	chr1:161590692	FCGR2B
rs332398	chr1:161536130	FCGR3A
rs1801274	chr1:161479744	FCGR2A
rs404508	chr1:161495884	FCGR2C
rs35181127	chr1:161590742	FCGR2B
rs404508	chr1:161495884	FCGR2B
rs332398	chr1:161536130	FCGR2B
rs368433	chr1:161484209	FCGR2A
rs35740080	chr1:161494310	FCGR2C
rs35428265	chr1:161592196	FCGR2C
rs34182452	chr1:161601349	FCGR3B
rs1771588	chr1:161610867	FCGR2C
rs35428265	chr1:161592196	FCGR2B
rs36063867	chr1:161590979	FCGR2C
rs2099684	chr1:161500129	FCGR2A
rs35276103	chr1:161592212	FCGR2B
rs36117182	chr1:161590652	FCGR2B
rs36063867	chr1:161590979	FCGR2B
rs35181127	chr1:161590742	FCGR3B
rs35674644	chr1:161592225	FCGR2C
rs553630	chr1:161582254	FCGR2C
rs34416533	chr1:161590692	FCGR3B
rs3754053	chr1:161575843	FCGR3B
rs34182452	chr1:161601349	FCGR2C
rs1771588	chr1:161610867	FCGR3B
rs34881159	chr1:161590502	FCGR2B
rs2165090	chr1:161457636	FCGR2A
rs36063867	chr1:161590979	FCGR3B

rs35276103	chr1:161592212	FCGR3B
rs553630	chr1:161582254	FCGR2B
rs426615	chr1:161513376	FCGR2B
rs35276103	chr1:161592212	FCGR3A
rs2994672	chr1:161606971	FCGR2C
rs35276103	chr1:161592212	FCGR2A
rs404508	chr1:161495884	FCGR3A
rs34881159	chr1:161590502	FCGR3A
rs438228	chr1:161484123	FCGR2A
rs36117182	chr1:161590652	FCGR2C
rs35181127	chr1:161590742	FCGR2A
rs17397332	chr1:161500894	FCGR2A
rs1801274	chr1:161479744	FCGR2C
rs332398	chr1:161536130	FCGR3B
rs553630	chr1:161582254	FCGR3B
rs1629019	chr1:161607390	FCGR2C
rs35740080	chr1:161494310	FCGR2B
rs35428265	chr1:161592196	FCGR3B
rs36117182	chr1:161590652	FCGR3B
rs60751059	chr1:161612665	FCGR2B
rs3754053	chr1:161575843	FCGR3A
rs1629019	chr1:161607390	FCGR3A
rs2994672	chr1:161606971	FCGR2A
rs1629019	chr1:161607390	FCGR3B
rs35293629	chr1:161601230	FCGR3B
rs35369398	chr1:161591547	FCGR2A
rs1629019	chr1:161607390	FCGR2B
rs35674644	chr1:161592225	FCGR3B
rs2994672	chr1:161606971	FCGR2B
rs36117182	chr1:161590652	FCGR2A
rs3754053	chr1:161575843	FCGR2B
rs430178	chr1:161569418	FCGR2C
rs60751059	chr1:161612665	FCGR3B
rs34416533	chr1:161590692	FCGR3A
rs1771588	chr1:161610867	FCGR2B
rs1771588	chr1:161610867	FCGR2A
rs426615	chr1:161513376	FCGR3B
rs2994672	chr1:161606971	FCGR3B
rs35369398	chr1:161591547	FCGR3A
rs35674644	chr1:161592225	FCGR2B
rs60751059	chr1:161612665	FCGR2C
rs2165090	chr1:161457636	FCGR2C
rs34160304	chr1:161590452	FCGR3B
rs2165090	chr1:161457636	FCGR2B
rs553630	chr1:161582254	FCGR3A
rs9427389	chr1:161443097	FCGR2A
rs34182452	chr1:161601349	FCGR2B
rs3843301	chr1:161614400	FCGR2B
rs34881159	chr1:161590502	FCGR2A
rs35740080	chr1:161494310	FCGR3B

rs34907291	chr1:161601538	FCGR3B
rs35181127	chr1:161590742	FCGR3A
rs79158401	chr1:161581509	FCGR2B
rs72633684	chr1:161582499	FCGR2B
rs1801274	chr1:161479744	FCGR2B
rs1771582	chr1:161614489	FCGR3B
rs2165090	chr1:161457636	FCGR3A
rs430178	chr1:161569418	FCGR2A
rs34160304	chr1:161590452	FCGR2B
rs36063867	chr1:161590979	FCGR3A
rs1771575	chr1:161617496	FCGR3A
rs404508	chr1:161495884	FCGR3B
rs1801274	chr1:161479744	FCGR3A
rs12731669	chr1:161410457	FCGR2A
rs36063867	chr1:161590979	FCGR2A
rs2099684	chr1:161500129	FCGR2C
rs61803005	chr1:161593570	FCGR2A
rs61803001	chr1:161592958	FCGR2A
rs34160304	chr1:161590452	FCGR2C
rs10429882	chr1:161515175	FCGR2A
rs17397332	chr1:161500894	FCGR2C
rs77996283	chr1:161564467	FCGR2A
rs72633684	chr1:161582499	FCGR2C
rs35293629	chr1:161601230	FCGR2C
rs4656310	chr1:161496899	FCGR2A
rs34907291	chr1:161601538	FCGR2C
rs36117182	chr1:161590652	FCGR3A
rs61803001	chr1:161592958	FCGR2B
rs17397332	chr1:161500894	FCGR2B
rs35428265	chr1:161592196	FCGR3A
rs35428265	chr1:161592196	FCGR2A
rs79158401	chr1:161581509	FCGR2C
rs72717048	chr1:161500795	FCGR2A
rs12045955	chr1:161688738	FCGR2B
rs114693598	chr1:161500711	FCGR2A
rs438228	chr1:161484123	FCGR2C
rs35293629	chr1:161601230	FCGR2B
rs2099684	chr1:161500129	FCGR3A
rs61803005	chr1:161593570	FCGR2C
rs34566209	chr1:161590348	FCGR3B
rs61803001	chr1:161592958	FCGR2C
rs430178	chr1:161569418	FCGR2B
rs2994672	chr1:161606971	FCGR3A
rs72702111	chr1:161581265	FCGR2B
rs3843301	chr1:161614400	FCGR2C
rs34907291	chr1:161601538	FCGR2B
rs368433	chr1:161484209	FCGR2C
rs60751059	chr1:161612665	FCGR2A
rs1771577	chr1:161617340	FCGR3B
rs35674644	chr1:161592225	FCGR3A

rs72633684	chr1:161582499	FCGR2A
rs438228	chr1:161484123	FCGR3A
rs74127051	chr1:161582401	FCGR2C
rs34322334	chr1:161601172	FCGR3B
rs60751059	chr1:161612665	FCGR3A
rs61803005	chr1:161593570	FCGR3B
rs7532275	chr1:161477746	FCGR2A
rs34160304	chr1:161590452	FCGR2A
rs72633684	chr1:161582499	FCGR3B
rs3843301	chr1:161614400	FCGR3B
rs1771575	chr1:161617496	FCGR2A
rs2099684	chr1:161500129	FCGR2B
rs17397332	chr1:161500894	FCGR3A
rs1771582	chr1:161614489	FCGR2C
rs2165090	chr1:161457636	FCGR3B
rs74127051	chr1:161582401	FCGR2B
rs77996283	chr1:161564467	FCGR2C
rs3002598	chr1:161620264	FCGR2C
rs1771588	chr1:161610867	FCGR3A
rs72702111	chr1:161581265	FCGR2A
rs74127049	chr1:161582199	FCGR2B
rs34160304	chr1:161590452	FCGR3A
rs16858998	chr1:161700087	FCGR2A
rs61803005	chr1:161593570	FCGR2B
rs61803001	chr1:161592958	FCGR3A
rs12045955	chr1:161688738	FCGR3B
rs1771573	chr1:161618786	FCGR3B
rs12731669	chr1:161410457	FCGR2C
rs9427399	chr1:161476532	FCGR2C
rs1801274	chr1:161479744	FCGR3B
rs72702111	chr1:161581265	FCGR2C
rs430178	chr1:161569418	FCGR3B
rs34566209	chr1:161590348	FCGR2C
rs77996283	chr1:161564467	FCGR3B
rs148948376	chr1:161591107	FCGR2B
rs148685469	chr1:161513831	FCGR2A
rs12045955	chr1:161688738	FCGR2C
rs7515174	chr1:161476948	FCGR2B
rs34182452	chr1:161601349	FCGR2A
rs79158401	chr1:161581509	FCGR3B
rs10737548	chr1:161493544	FCGR2A
rs34322334	chr1:161601172	FCGR2C
rs74127051	chr1:161582401	FCGR2A
rs12045955	chr1:161688738	FCGR2A
rs9427399	chr1:161476532	FCGR2A
rs72702114	chr1:161581802	FCGR2C
rs1771577	chr1:161617340	FCGR2C
rs10429882	chr1:161515175	FCGR2C
rs34085961	chr1:161600994	FCGR2C
rs16858998	chr1:161700087	FCGR2B

rs3002598	chr1:161620264	FCGR2B
rs10429882	chr1:161515175	FCGR3A
rs34566209	chr1:161590348	FCGR2B
rs12731669	chr1:161410457	FCGR2B
rs9427397	chr1:161476203	FCGR2A
rs16858998	chr1:161700087	FCGR2C
rs148948376	chr1:161591107	FCGR2C
rs77996283	chr1:161564467	FCGR2B
rs1771582	chr1:161614489	FCGR2B
rs114693598	chr1:161500711	FCGR2C
rs9427389	chr1:161443097	FCGR3A
rs3754053	chr1:161575843	FCGR2A
rs74127055	chr1:161591323	FCGR2B
rs3002598	chr1:161620264	FCGR3B
rs74127049	chr1:161582199	FCGR2C
rs1771572	chr1:161619639	FCGR3B
rs1771576	chr1:161617365	FCGR3B
rs115588892	chr1:161709147	FCGR3B
rs74127055	chr1:161591323	FCGR2C
rs9427388	chr1:161443085	FCGR2A
rs9427399	chr1:161476532	FCGR2B
rs61803001	chr1:161592958	FCGR3B
rs9427389	chr1:161443097	FCGR2C
rs9427061	chr1:161442859	FCGR2C
rs368433	chr1:161484209	FCGR2B
rs72702114	chr1:161581802	FCGR2B
rs1771572	chr1:161619639	FCGR2B
rs430178	chr1:161569418	FCGR3A
rs74127055	chr1:161591323	FCGR3B
rs144577943	chr1:161592396	FCGR2B
rs149210339	chr1:161515871	FCGR2A
rs4657054	chr1:161493304	FCGR2A
rs1771577	chr1:161617340	FCGR2B
rs9427397	chr1:161476203	FCGR2C
rs112522513	chr1:161735380	FCGR2C
rs61803005	chr1:161593570	FCGR3A
rs115588892	chr1:161709147	FCGR2A
rs17397332	chr1:161500894	FCGR3B
rs61801184	chr1:161703434	FCGR2C
rs35674644	chr1:161592225	FCGR2A
rs1629019	chr1:161607390	FCGR2A
rs74127051	chr1:161582401	FCGR3B
rs61801184	chr1:161703434	FCGR2B
rs368433	chr1:161484209	FCGR3A
rs115588892	chr1:161709147	FCGR2B
rs5030738	chr1:161599653	FCGR2C
rs9427397	chr1:161476203	FCGR2B
rs72702111	chr1:161581265	FCGR3B
rs4656317	chr1:161519118	FCGR2C
rs4657054	chr1:161493304	FCGR2C

rs72633684	chr1:161582499	FCGR3A
rs74127049	chr1:161582199	FCGR3B
rs12045955	chr1:161688738	FCGR3A
rs3843301	chr1:161614400	FCGR3A
rs2085694	chr1:161591978	FCGR2C
rs367724155	chr1:161514301	FCGR2A
rs9427061	chr1:161442859	FCGR2A
rs438228	chr1:161484123	FCGR2B
rs78603229	chr1:161581508	FCGR2B
rs2099684	chr1:161500129	FCGR3B
rs367724155	chr1:161514301	FCGR2C
rs34085961	chr1:161600994	FCGR3B
rs17450862	chr1:161904480	FCGR2B
rs72717048	chr1:161500795	FCGR2B
rs438228	chr1:161484123	FCGR3B
rs1771572	chr1:161619639	FCGR2C
rs114693598	chr1:161500711	FCGR2B
rs72717048	chr1:161500795	FCGR2C
rs9427060	chr1:161442434	FCGR2C
rs9427398	chr1:161476204	FCGR2C
rs111892839	chr1:161736051	FCGR2C
rs4656317	chr1:161519118	FCGR2A
rs34322334	chr1:161601172	FCGR2B
rs9427398	chr1:161476204	FCGR2B
rs111892839	chr1:161736051	FCGR3A
rs79158401	chr1:161581509	FCGR2A
rs148948376	chr1:161591107	FCGR3B
rs553630	chr1:161582254	FCGR2A
rs10917571	chr1:161519410	FCGR2A
rs140970775	chr1:161503012	FCGR2C
rs7532275	chr1:161477746	FCGR3A
rs72706039	chr1:161740155	FCGR2B
rs56369941	chr1:161606970	FCGR2C
rs111256263	chr1:161905434	FCGR2B
rs2085694	chr1:161591978	FCGR2B
rs17415303	chr1:161721266	FCGR2B
rs7515174	chr1:161476948	FCGR2C
rs74127051	chr1:161582401	FCGR3A
rs112522513	chr1:161735380	FCGR2B
rs9427062	chr1:161442860	FCGR2C
rs74127049	chr1:161582199	FCGR2A
rs61802313	chr1:161581158	FCGR2C
rs144265235	chr1:161581513	FCGR2B
rs12756398	chr1:161606925	FCGR2C
rs148948376	chr1:161591107	FCGR2A
rs78603229	chr1:161581508	FCGR2C
rs144577943	chr1:161592396	FCGR2C
rs72704062	chr1:161646624	FCGR2C
rs77996283	chr1:161564467	FCGR3A
rs113058868	chr1:161747043	FCGR2B

rs1771573	chr1:161618786	FCGR2B
rs79158401	chr1:161581509	FCGR3A
rs10737548	chr1:161493544	FCGR3A
rs114693598	chr1:161500711	FCGR3A
rs144577943	chr1:161592396	FCGR3B
rs114693598	chr1:161500711	FCGR3B
rs72702111	chr1:161581265	FCGR3A
rs4656310	chr1:161496899	FCGR2C
rs9427389	chr1:161443097	FCGR2B
rs72702114	chr1:161581802	FCGR2A
rs61801191	chr1:161708499	FCGR3B
rs12731669	chr1:161410457	FCGR3A
rs56199187	chr1:161520930	FCGR2A
rs10494360	chr1:161475749	FCGR2A
rs61802313	chr1:161581158	FCGR2B
rs61801184	chr1:161703434	FCGR3B
rs115588892	chr1:161709147	FCGR2C
rs72702116	chr1:161582451	FCGR2B
rs7515174	chr1:161476948	FCGR3A
rs35293629	chr1:161601230	FCGR3A
rs16858998	chr1:161700087	FCGR3A
rs12731669	chr1:161410457	FCGR3B
rs35284289	chr1:161736208	FCGR2C
rs7532275	chr1:161477746	FCGR2C
rs56369941	chr1:161606970	FCGR3B
rs61802313	chr1:161581158	FCGR3A
rs9427397	chr1:161476203	FCGR3A
rs72702116	chr1:161582451	FCGR2C
rs12756398	chr1:161606925	FCGR2A
rs72704062	chr1:161646624	FCGR2B
rs1771573	chr1:161618786	FCGR2C
rs10737548	chr1:161493544	FCGR2C
rs72717048	chr1:161500795	FCGR3A
rs61802313	chr1:161581158	FCGR3B
rs112522513	chr1:161735380	FCGR3B
rs74127055	chr1:161591323	FCGR2A
rs72706014	chr1:161720348	FCGR2C
rs12142755	chr1:161481742	FCGR2C
rs55971447	chr1:161515325	FCGR2C
rs9427398	chr1:161476204	FCGR2A
rs7539468	chr1:161477065	FCGR2B
rs34182452	chr1:161601349	FCGR3A
rs56199187	chr1:161520930	FCGR2C
rs72706014	chr1:161720348	FCGR3A
rs367724155	chr1:161514301	FCGR3A
rs16858998	chr1:161700087	FCGR3B
rs148685469	chr1:161513831	FCGR2C
rs7515174	chr1:161476948	FCGR2A
rs72702114	chr1:161581802	FCGR3B
rs10429882	chr1:161515175	FCGR2B

rs61801191	chr1:161708499	FCGR2C
rs9427062	chr1:161442860	FCGR2A
rs72702116	chr1:161582451	FCGR3B
rs10737548	chr1:161493544	FCGR2B
rs56315079	chr1:161475874	FCGR3A
rs9427061	chr1:161442859	FCGR2B
rs10917571	chr1:161519410	FCGR3A
rs12142755	chr1:161481742	FCGR3A
rs1771576	chr1:161617365	FCGR2C
rs78603229	chr1:161581508	FCGR3B
rs55971447	chr1:161515325	FCGR3B
rs112522513	chr1:161735380	FCGR3A
rs9427388	chr1:161443085	FCGR2C
rs4656310	chr1:161496899	FCGR3A
rs77258102	chr1:161564511	FCGR3B
rs77258102	chr1:161564511	FCGR2C
rs11810143	chr1:161480648	FCGR2A
rs7532275	chr1:161477746	FCGR2B
rs5030738	chr1:161599653	FCGR2B
rs74127055	chr1:161591323	FCGR3A
rs61802313	chr1:161581158	FCGR2A
rs148685469	chr1:161513831	FCGR3A
rs5030738	chr1:161599653	FCGR3B
rs7539468	chr1:161477065	FCGR2C
rs2085694	chr1:161591978	FCGR3B
rs61801191	chr1:161708499	FCGR3A
rs56315079	chr1:161475874	FCGR2C
rs4656311	chr1:161498849	FCGR2A
rs56369941	chr1:161606970	FCGR2A
rs34907291	chr1:161601538	FCGR3A
rs56315079	chr1:161475874	FCGR2B
rs10429882	chr1:161515175	FCGR3B
rs148948376	chr1:161591107	FCGR3A
rs144577943	chr1:161592396	FCGR2A
rs367724155	chr1:161514301	FCGR2B
rs6427591	chr1:161408368	FCGR2C
rs55971447	chr1:161515325	FCGR2A
rs35284289	chr1:161736208	FCGR3A
rs56315079	chr1:161475874	FCGR2A
rs9427399	chr1:161476532	FCGR3A
rs10494360	chr1:161475749	FCGR2B
rs12756398	chr1:161606925	FCGR2B
rs35284289	chr1:161736208	FCGR3B
rs34566209	chr1:161590348	FCGR3A
rs10494360	chr1:161475749	FCGR2C
rs111892839	chr1:161736051	FCGR3B
rs56369941	chr1:161606970	FCGR2B
rs71632989	chr1:161533054	FCGR2C
rs2446622	chr1:161606972	FCGR2C
rs77258102	chr1:161564511	FCGR3A

rs9427060	chr1:161442434	FCGR2A
rs114817585	chr1:161582478	FCGR2B
rs34085961	chr1:161600994	FCGR2B
rs755339	chr1:161582677	FCGR2C
rs61802998	chr1:161591541	FCGR2B
rs72702114	chr1:161581802	FCGR3A
rs10494360	chr1:161475749	FCGR3A
rs61802998	chr1:161591541	FCGR2C
rs61801180	chr1:161688763	FCGR2B
rs9427398	chr1:161476204	FCGR3A
rs114817585	chr1:161582478	FCGR2C
rs9427397	chr1:161476203	FCGR3B
rs72704062	chr1:161646624	FCGR3B
rs4657054	chr1:161493304	FCGR3B
rs755339	chr1:161582677	FCGR3B
rs74127049	chr1:161582199	FCGR3A
rs61801235	chr1:161722648	FCGR3B
rs140970775	chr1:161503012	FCGR3B
rs55971447	chr1:161515325	FCGR2B
rs77258102	chr1:161564511	FCGR2A
rs2085694	chr1:161591978	FCGR2A
rs12736433	chr1:161410121	FCGR2A
rs4656310	chr1:161496899	FCGR2B
rs9427388	chr1:161443085	FCGR2B
rs9427398	chr1:161476204	FCGR3B
rs61801191	chr1:161708499	FCGR2B
rs9427062	chr1:161442860	FCGR2B
rs9427060	chr1:161442434	FCGR2B
rs72702116	chr1:161582451	FCGR2A
rs17415303	chr1:161721266	FCGR2C
rs7539468	chr1:161477065	FCGR3A
rs114817585	chr1:161582478	FCGR2A
rs111892839	chr1:161736051	FCGR2B
rs1771577	chr1:161617340	FCGR2A
rs7539468	chr1:161477065	FCGR2A
rs144265235	chr1:161581513	FCGR3B
rs144265235	chr1:161581513	FCGR2C
rs4657042	chr1:161482519	FCGR3A
rs4656317	chr1:161519118	FCGR3A
rs755339	chr1:161582677	FCGR2B
rs78603229	chr1:161581508	FCGR3A
rs17416920	chr1:161903651	FCGR2B
rs72717048	chr1:161500795	FCGR3B
rs368433	chr1:161484209	FCGR3B
rs1771576	chr1:161617365	FCGR2B
rs61801823	chr1:161565957	FCGR2C
rs61802996	chr1:161591066	FCGR2B
rs17450862	chr1:161904480	FCGR3B
rs56238128	chr1:161531781	FCGR2A
rs140970775	chr1:161503012	FCGR3A

rs1771573	chr1:161618786	FCGR3A
rs11810143	chr1:161480648	FCGR3A
rs2880055	chr1:161922364	FCGR2B
rs9427389	chr1:161443097	FCGR3B
rs10917571	chr1:161519410	FCGR3B
rs56199187	chr1:161520930	FCGR3B
rs12021510	chr1:161709210	FCGR2C
rs9427061	chr1:161442859	FCGR3A
rs35284289	chr1:161736208	FCGR2B
rs61801235	chr1:161722648	FCGR2B
rs17411858	chr1:161617515	FCGR2B
rs755339	chr1:161582677	FCGR2A
rs17411858	chr1:161617515	FCGR2C
rs2002405	chr1:161583106	FCGR2C
rs7532275	chr1:161477746	FCGR3B
rs114817585	chr1:161582478	FCGR3B
rs61804323	chr1:161525632	FCGR3A
rs61802998	chr1:161591541	FCGR3B
rs72706022	chr1:161724126	FCGR2B
rs12021510	chr1:161709210	FCGR2B
rs115383270	chr1:161531339	FCGR2C
rs72706014	chr1:161720348	FCGR2B
rs10917571	chr1:161519410	FCGR2C
rs9427399	chr1:161476532	FCGR3B
rs17416633	chr1:161888268	FCGR2B
rs72706023	chr1:161724248	FCGR2B
rs56199187	chr1:161520930	FCGR3A
rs56199187	chr1:161520930	FCGR2B
rs149210339	chr1:161515871	FCGR2C
rs10917571	chr1:161519410	FCGR2B
rs4657039	chr1:161462727	FCGR2C
rs34566209	chr1:161590348	FCGR2A
rs12756398	chr1:161606925	FCGR3B
rs17415303	chr1:161721266	FCGR3B
rs146617619	chr1:161592477	FCGR2C
rs4657054	chr1:161493304	FCGR3A
rs111281737	chr1:161738637	FCGR2B
rs552366171	chr1:161410648	FCGR2A
rs2085694	chr1:161591978	FCGR3A
rs180854462	chr1:161410177	FCGR2A
rs17450862	chr1:161904480	FCGR3A
rs12021510	chr1:161709210	FCGR3B
rs11810143	chr1:161480648	FCGR3B
rs61802998	chr1:161591541	FCGR2A
rs7552317	chr1:161479437	FCGR3A
rs76652463	chr1:161741910	FCGR2B
rs140970775	chr1:161503012	FCGR2B
rs12044611	chr1:161697546	FCGR3B
rs4657042	chr1:161482519	FCGR2C
rs17411858	chr1:161617515	FCGR3B

rs140970775	chr1:161503012	FCGR2A
rs61802335	chr1:161590402	FCGR2C
rs4656317	chr1:161519118	FCGR3B
rs61802996	chr1:161591066	FCGR3B
rs71632960	chr1:161516359	FCGR2C
rs114591250	chr1:161905423	FCGR2B
rs72706023	chr1:161724248	FCGR3B
rs113682966	chr1:161734724	FCGR2C
rs72706014	chr1:161720348	FCGR3B
rs61802335	chr1:161590402	FCGR3B
rs2446622	chr1:161606972	FCGR3B
rs61802996	chr1:161591066	FCGR2C
rs78876532	chr1:161574627	FCGR2C
rs1771582	chr1:161614489	FCGR3A
rs12044611	chr1:161697546	FCGR2C
rs56238128	chr1:161531781	FCGR3A
rs4657054	chr1:161493304	FCGR2B
rs148685469	chr1:161513831	FCGR2B
rs180854462	chr1:161410177	FCGR2C
rs111281737	chr1:161738637	FCGR3B
rs9427400	chr1:161477203	FCGR2B
rs146617619	chr1:161592477	FCGR3B
rs76652463	chr1:161741910	FCGR2A
rs4657039	chr1:161462727	FCGR3A
rs9427400	chr1:161477203	FCGR3A
rs5030738	chr1:161599653	FCGR3A
rs61804323	chr1:161525632	FCGR2C
rs180854462	chr1:161410177	FCGR3A
rs2002405	chr1:161583106	FCGR2B
rs76652463	chr1:161741910	FCGR2C
rs76652463	chr1:161741910	FCGR3B
rs6671753	chr1:161478707	FCGR2C
rs12142755	chr1:161481742	FCGR2B
rs56238128	chr1:161531781	FCGR2C
rs12140361	chr1:161321997	FCGR2C
rs9427062	chr1:161442860	FCGR3A
rs17450862	chr1:161904480	FCGR2A
rs367724155	chr1:161514301	FCGR3B
rs115383270	chr1:161531339	FCGR2A
rs115383270	chr1:161531339	FCGR3B
rs144126567	chr1:161510515	FCGR2B
rs115383270	chr1:161531339	FCGR3A
rs148685469	chr1:161513831	FCGR3B
rs72706039	chr1:161740155	FCGR2C
rs71632960	chr1:161516359	FCGR2A
rs144577943	chr1:161592396	FCGR3A
rs61801238	chr1:161724302	FCGR2B
rs71632989	chr1:161533054	FCGR2A
rs9427061	chr1:161442859	FCGR3B
rs79654278	chr1:161700647	FCGR2B

rs67411091	chr1:161593961	FCGR3B
rs144265235	chr1:161581513	FCGR3A
rs4656311	chr1:161498849	FCGR2C
rs2002405	chr1:161583106	FCGR3A
rs113058868	chr1:161747043	FCGR2C
rs144265235	chr1:161581513	FCGR2A
rs56315079	chr1:161475874	FCGR3B
rs77258102	chr1:161564511	FCGR2B
rs111281737	chr1:161738637	FCGR2C
rs61804323	chr1:161525632	FCGR2A
rs12140361	chr1:161321997	FCGR2B
rs9427400	chr1:161477203	FCGR2C
rs17450862	chr1:161904480	FCGR2C
rs1954172	chr1:161677734	FCGR2B
rs113682966	chr1:161734724	FCGR2B
rs114817585	chr1:161582478	FCGR3A
rs180854462	chr1:161410177	FCGR2B
rs34085961	chr1:161600994	FCGR3A
rs905595	chr1:161684889	FCGR2B
rs2002405	chr1:161583106	FCGR3B
rs72717013	chr1:161443371	FCGR2C
rs61803006	chr1:161593717	FCGR3B
rs111256263	chr1:161905434	FCGR2C
rs1954172	chr1:161677734	FCGR2C
rs56369941	chr1:161606970	FCGR3A
rs61801182	chr1:161699245	FCGR2B
rs755339	chr1:161582677	FCGR3A
rs9427059	chr1:161442334	FCGR2C
rs113060703	chr1:161736271	FCGR3B
rs71632960	chr1:161516359	FCGR3B
rs56124956	chr1:161725791	FCGR2C
rs61802335	chr1:161590402	FCGR2B
rs17415553	chr1:161734303	FCGR2C
rs3843301	chr1:161614400	FCGR2A
rs3002598	chr1:161620264	FCGR3A
rs12752280	chr1:161607376	FCGR3B
rs12736433	chr1:161410121	FCGR2C
rs113682966	chr1:161734724	FCGR2A
rs72706023	chr1:161724248	FCGR2C
rs61804323	chr1:161525632	FCGR3B
rs4657039	chr1:161462727	FCGR2B
rs112896977	chr1:161924114	FCGR2B
rs61802996	chr1:161591066	FCGR2A
rs4656310	chr1:161496899	FCGR3B
rs115264949	chr1:161501931	FCGR2C
rs113682966	chr1:161734724	FCGR3B
rs78603229	chr1:161581508	FCGR2A
rs67411091	chr1:161593961	FCGR2C
rs61802998	chr1:161591541	FCGR3A
rs72717013	chr1:161443371	FCGR2B

rs72717013	chr1:161443371	FCGR2A
rs2290832	chr1:161596703	FCGR3B
rs12736433	chr1:161410121	FCGR3B
rs117827527	chr1:161605888	FCGR2C
rs2446622	chr1:161606972	FCGR2B
rs72702116	chr1:161582451	FCGR3A
rs72706022	chr1:161724126	FCGR3B
rs55971447	chr1:161515325	FCGR3A
rs1771577	chr1:161617340	FCGR3A
rs144126567	chr1:161510515	FCGR2A
rs12756398	chr1:161606925	FCGR3A
rs9427388	chr1:161443085	FCGR3B
rs10737548	chr1:161493544	FCGR3B
rs905595	chr1:161684889	FCGR3B
rs144126567	chr1:161510515	FCGR2C
rs1771572	chr1:161619639	FCGR3A
rs115588892	chr1:161709147	FCGR3A
rs6671753	chr1:161478707	FCGR2B
rs67411091	chr1:161593961	FCGR2B
rs7518087	chr1:161477815	FCGR3A
rs4657039	chr1:161462727	FCGR2A
rs61801180	chr1:161688763	FCGR2C
rs146617619	chr1:161592477	FCGR2B
rs115264949	chr1:161501931	FCGR2B
rs113058868	chr1:161747043	FCGR3B
rs9427060	chr1:161442434	FCGR3A
rs4656311	chr1:161498849	FCGR3A
rs138010330	chr1:161526186	FCGR2A
rs143493683	chr1:161607280	FCGR2C
rs12752280	chr1:161607376	FCGR2C
rs9427387	chr1:161443040	FCGR2A
rs12736433	chr1:161410121	FCGR2B
rs12142755	chr1:161481742	FCGR2A
rs905595	chr1:161684889	FCGR2C
rs67167367	chr1:161593957	FCGR2B
rs72704062	chr1:161646624	FCGR3A
rs113682966	chr1:161734724	FCGR3A
rs78876532	chr1:161574627	FCGR2B
rs17415553	chr1:161734303	FCGR3B
rs4656317	chr1:161519118	FCGR2B
rs149210339	chr1:161515871	FCGR3B
rs56238128	chr1:161531781	FCGR3B
rs7529225	chr1:161479351	FCGR2A
rs61802996	chr1:161591066	FCGR3A
rs71632989	chr1:161533054	FCGR2B
rs6427591	chr1:161408368	FCGR2B
rs2446622	chr1:161606972	FCGR2A
rs61801180	chr1:161688763	FCGR3B
rs552366171	chr1:161410648	FCGR2C
rs9427388	chr1:161443085	FCGR3A

rs149210339	chr1:161515871	FCGR2B
rs7515174	chr1:161476948	FCGR3B
rs12736433	chr1:161410121	FCGR3A
rs61802324	chr1:161588079	FCGR2C
rs2002405	chr1:161583106	FCGR2A
rs6671753	chr1:161478707	FCGR3A
rs1674766	chr1:161607145	FCGR3B
rs34754216	chr1:161575537	FCGR2C
rs12044611	chr1:161697546	FCGR2B
rs9427387	chr1:161443040	FCGR2C
rs4657039	chr1:161462727	FCGR3B
rs9427059	chr1:161442334	FCGR2B
rs34754216	chr1:161575537	FCGR3B
rs111612785	chr1:161890330	FCGR2B
rs111256263	chr1:161905434	FCGR3B
rs79654278	chr1:161700647	FCGR3B
rs143493683	chr1:161607280	FCGR3B
rs61803006	chr1:161593717	FCGR2C
rs11810143	chr1:161480648	FCGR2C
rs7518087	chr1:161477815	FCGR2C
rs74127076	chr1:161600743	FCGR3B
rs77066692	chr1:161564347	FCGR2C
rs12021510	chr1:161709210	FCGR2A
rs115383270	chr1:161531339	FCGR2B
rs17415553	chr1:161734303	FCGR2B
rs6671753	chr1:161478707	FCGR2A
rs78876532	chr1:161574627	FCGR3B
rs144126567	chr1:161510515	FCGR3B
rs61801832	chr1:161570802	FCGR2C
rs67167367	chr1:161593957	FCGR3B
rs138010330	chr1:161526186	FCGR3B
rs4656311	chr1:161498849	FCGR2B
rs71632979	chr1:161526396	FCGR3B
rs61802335	chr1:161590402	FCGR2A
rs61801180	chr1:161688763	FCGR3A
rs144126567	chr1:161510515	FCGR3A
rs9427400	chr1:161477203	FCGR3B
rs9427062	chr1:161442860	FCGR3B
rs74127053	chr1:161582548	FCGR2B
rs61801180	chr1:161688763	FCGR2A
rs35520307	chr1:161199430	FCGR2C
rs12752280	chr1:161607376	FCGR2B
rs60574804	chr1:161478168	FCGR2C
rs71632989	chr1:161533054	FCGR3B
rs117827527	chr1:161605888	FCGR3A
rs113060703	chr1:161736271	FCGR2C
rs113060703	chr1:161736271	FCGR2B
rs511278	chr1:161490895	FCGR2C
rs145335641	chr1:161592674	FCGR2C
rs1954172	chr1:161677734	FCGR3B

rs34754216	chr1:161575537	FCGR2B
rs10494360	chr1:161475749	FCGR3B
rs12136414	chr1:161678421	FCGR2B
rs1771576	chr1:161617365	FCGR3A
rs72706039	chr1:161740155	FCGR3B
rs61803006	chr1:161593717	FCGR3A
rs145335641	chr1:161592674	FCGR3B
rs9427387	chr1:161443040	FCGR2B
rs115264949	chr1:161501931	FCGR2A
rs4656311	chr1:161498849	FCGR3B
rs61801833	chr1:161571302	FCGR2C
rs9427400	chr1:161477203	FCGR2A
rs16832790	chr1:161275942	FCGR3A
rs74127053	chr1:161582548	FCGR2C
rs17411858	chr1:161617515	FCGR2A
rs138010330	chr1:161526186	FCGR3A
rs145335641	chr1:161592674	FCGR2B
rs180854462	chr1:161410177	FCGR3B
rs115827707	chr1:161530106	FCGR2C
rs61801182	chr1:161699245	FCGR2C
rs9427402	chr1:161492666	FCGR2C
rs17415553	chr1:161734303	FCGR3A
rs67167367	chr1:161593957	FCGR2A
rs9427059	chr1:161442334	FCGR3A
rs143493683	chr1:161607280	FCGR2B
rs71632979	chr1:161526396	FCGR2C
rs9427060	chr1:161442434	FCGR3B
rs12136414	chr1:161678421	FCGR2C
rs12752280	chr1:161607376	FCGR3A
rs4656309	chr1:161478789	FCGR3A
rs17411858	chr1:161617515	FCGR3A
rs12136414	chr1:161678421	FCGR3B
rs34322334	chr1:161601172	FCGR3A
rs2333748	chr1:161615545	FCGR2C
rs12044611	chr1:161697546	FCGR3A
rs61802299	chr1:161574745	FCGR2C
rs35520307	chr1:161199430	FCGR2B
rs139529340	chr1:161563553	FCGR2C
rs34527977	chr1:161596996	FCGR3B
rs149210339	chr1:161515871	FCGR3A
rs113060703	chr1:161736271	FCGR3A
rs35293629	chr1:161601230	FCGR2A
rs74127076	chr1:161600743	FCGR2C
rs145335641	chr1:161592674	FCGR3A
rs11810143	chr1:161480648	FCGR2B
rs12021510	chr1:161709210	FCGR3A
rs117827527	chr1:161605888	FCGR3B
rs61804323	chr1:161525632	FCGR2B
rs7529225	chr1:161479351	FCGR2B
rs422670	chr1:161569147	FCGR2C

rs115264949	chr1:161501931	FCGR3A
rs61802335	chr1:161590402	FCGR3A
rs17448307	chr1:161791339	FCGR2B
rs145335641	chr1:161592674	FCGR2A
rs148885671	chr1:161588133	FCGR2C
rs67167367	chr1:161593957	FCGR2C
rs35046931	chr1:161314112	FCGR2C
rs61801184	chr1:161703434	FCGR3A
rs1771573	chr1:161618786	FCGR2A
rs61803006	chr1:161593717	FCGR2B
rs76652463	chr1:161741910	FCGR3A
rs12142755	chr1:161481742	FCGR3B
rs6427592	chr1:161408396	FCGR2C
rs74127053	chr1:161582548	FCGR3A
rs111256263	chr1:161905434	FCGR3A
rs112045543	chr1:161587760	FCGR2C
rs71632960	chr1:161516359	FCGR3A
rs34540988	chr1:161575760	FCGR2B
rs9427396	chr1:161451892	FCGR2C
rs2290832	chr1:161596703	FCGR2B
rs61801823	chr1:161565957	FCGR3A
rs17406901	chr1:161726018	FCGR2C
rs3002598	chr1:161620264	FCGR2A
rs552366171	chr1:161410648	FCGR3A
rs552366171	chr1:161410648	FCGR2B
rs79654278	chr1:161700647	FCGR2C
rs5030738	chr1:161599653	FCGR2A
rs143493683	chr1:161607280	FCGR3A
rs12140361	chr1:161321997	FCGR3A
rs9427387	chr1:161443040	FCGR3B
rs16857520	chr1:161691747	FCGR3B
rs60574804	chr1:161478168	FCGR2B
rs2290832	chr1:161596703	FCGR2C
rs6427592	chr1:161408396	FCGR2B
rs72717013	chr1:161443371	FCGR3A
rs115827707	chr1:161530106	FCGR3B
rs7518087	chr1:161477815	FCGR3B
rs115827707	chr1:161530106	FCGR3A
rs71632989	chr1:161533054	FCGR3A
rs55864322	chr1:161306464	FCGR2C
rs56124956	chr1:161725791	FCGR3B
rs77825069	chr1:161517684	FCGR2C
rs138010330	chr1:161526186	FCGR2C
rs2290832	chr1:161596703	FCGR3A
rs16832790	chr1:161275942	FCGR2A
rs61802299	chr1:161574745	FCGR2B
rs7518087	chr1:161477815	FCGR2B
rs4656309	chr1:161478789	FCGR2C
rs34540988	chr1:161575760	FCGR2C
rs61802299	chr1:161574745	FCGR3B

rs146617619	chr1:161592477	FCGR3A
rs9427402	chr1:161492666	FCGR2A
rs9427059	chr1:161442334	FCGR2A
rs111744179	chr1:161776942	FCGR2B
rs77858450	chr1:161700648	FCGR3B
rs7529225	chr1:161479351	FCGR3A
rs12756382	chr1:161607517	FCGR3B
rs60574804	chr1:161478168	FCGR3B
rs117827527	chr1:161605888	FCGR2B
rs67167367	chr1:161593957	FCGR3A
rs4657042	chr1:161482519	FCGR2B
rs138728964	chr1:161607118	FCGR2B
rs17415303	chr1:161721266	FCGR3A
rs146653557	chr1:161593121	FCGR3B
rs2446622	chr1:161606972	FCGR3A
rs61804162	chr1:161622834	FCGR2C
rs77858450	chr1:161700648	FCGR2B
rs1771582	chr1:161614489	FCGR2A
rs60574804	chr1:161478168	FCGR3A
rs61801235	chr1:161722648	FCGR2C
rs7552317	chr1:161479437	FCGR3B
rs16832790	chr1:161275942	FCGR2C
rs10917740	chr1:161680011	FCGR2B
rs111281737	chr1:161738637	FCGR3A
rs72717013	chr1:161443371	FCGR3B
rs10919544	chr1:161508762	FCGR2C
rs4657042	chr1:161482519	FCGR2A
rs543438691	chr1:161597123	FCGR2C
rs17406901	chr1:161726018	FCGR3B
rs138728964	chr1:161607118	FCGR2C
rs138728964	chr1:161607118	FCGR3B
rs61801182	chr1:161699245	FCGR3B
rs9427065	chr1:161474960	FCGR2C
rs1674766	chr1:161607145	FCGR2B
rs2333748	chr1:161615545	FCGR3B
rs9427065	chr1:161474960	FCGR3A
rs1674757	chr1:161644386	FCGR2C
rs16857520	chr1:161691747	FCGR2B
rs71632979	chr1:161526396	FCGR2A
rs71632979	chr1:161526396	FCGR3A
rs17415553	chr1:161734303	FCGR2A
rs67411091	chr1:161593961	FCGR3A
rs17416920	chr1:161903651	FCGR3B
rs7552317	chr1:161479437	FCGR2A
rs1674766	chr1:161607145	FCGR2C
rs149686267	chr1:161600108	FCGR2C
rs41271949	chr1:161771811	FCGR2C
rs111971126	chr1:161563695	FCGR2C
rs61801191	chr1:161708499	FCGR2A
rs72706022	chr1:161724126	FCGR2C

rs183786059	chr1:161800435	FCGR2B
rs34322334	chr1:161601172	FCGR2A
rs146423333	chr1:161722607	FCGR3B
rs511278	chr1:161490895	FCGR2A
rs75131185	chr1:161488942	FCGR2C
rs7529225	chr1:161479351	FCGR3B
rs61803026	chr1:161601117	FCGR2C
rs61801823	chr1:161565957	FCGR3B
rs61802324	chr1:161588079	FCGR3B
rs61802324	chr1:161588079	FCGR2B
rs41299300	chr1:161579686	FCGR2C
rs72717016	chr1:161450340	FCGR2C
rs143493683	chr1:161607280	FCGR2A
rs17406901	chr1:161726018	FCGR2B
rs116422836	chr1:161588312	FCGR2C
rs34527977	chr1:161596996	FCGR2C
rs7518642	chr1:161607354	FCGR3B
rs7529225	chr1:161479351	FCGR2C
rs34527977	chr1:161596996	FCGR2B
rs75417510	chr1:161528063	FCGR2C
rs61801829	chr1:161570106	FCGR2C
rs1674766	chr1:161607145	FCGR3A
rs2290832	chr1:161596703	FCGR2A
rs7539468	chr1:161477065	FCGR3B
rs10919543	chr1:161508616	FCGR2C
rs552366171	chr1:161410648	FCGR3B
rs148885671	chr1:161588133	FCGR3B
rs35520307	chr1:161199430	FCGR2A
rs111281737	chr1:161738637	FCGR2A
rs2045573	chr1:161618714	FCGR3B
rs61801830	chr1:161570202	FCGR2C
rs72700095	chr1:161567188	FCGR2C
rs72706039	chr1:161740155	FCGR2A
rs12756382	chr1:161607517	FCGR2C
rs139529340	chr1:161563553	FCGR2B
rs139529340	chr1:161563553	FCGR3A
rs79654278	chr1:161700647	FCGR3A
rs66888136	chr1:161315277	FCGR2C
rs61801834	chr1:161571748	FCGR2C
rs61801823	chr1:161565957	FCGR2B
rs61802301	chr1:161575148	FCGR3B
rs112522513	chr1:161735380	FCGR2A
rs867624	chr1:161467836	FCGR2C
rs1674757	chr1:161644386	FCGR3B
rs4656309	chr1:161478789	FCGR2B
rs34527977	chr1:161596996	FCGR3A
rs117827527	chr1:161605888	FCGR2A
rs60574804	chr1:161478168	FCGR2A
rs9427059	chr1:161442334	FCGR3B
rs2045572	chr1:161643642	FCGR2C

rs9427387	chr1:161443040	FCGR3A
rs1674766	chr1:161607145	FCGR2A
rs1771572	chr1:161619639	FCGR2A
rs78876532	chr1:161574627	FCGR3A
rs116422836	chr1:161588312	FCGR3B
rs34540988	chr1:161575760	FCGR3B
rs61803006	chr1:161593717	FCGR2A
rs2880055	chr1:161922364	FCGR3B
rs56124956	chr1:161725791	FCGR2B
rs61801831	chr1:161570662	FCGR2C
rs61804161	chr1:161622756	FCGR3B
rs77066692	chr1:161564347	FCGR3B
rs12043181	chr1:161680790	FCGR2B
rs114591250	chr1:161905423	FCGR2C
rs74127053	chr1:161582548	FCGR2A
rs113058868	chr1:161747043	FCGR2A
rs146423333	chr1:161722607	FCGR2C
rs61803029	chr1:161601520	FCGR2C
rs74127053	chr1:161582548	FCGR3B
rs149686267	chr1:161600108	FCGR3B
rs61804161	chr1:161622756	FCGR2C
rs77169596	chr1:161534616	FCGR2C
rs518377	chr1:161573294	FCGR2C
rs17416633	chr1:161888268	FCGR2C
rs61801832	chr1:161570802	FCGR2B
rs146777125	chr1:161571941	FCGR2C
rs4656309	chr1:161478789	FCGR3B
rs113263422	chr1:161791735	FCGR3B
rs2045572	chr1:161643642	FCGR2B
rs71632960	chr1:161516359	FCGR2B
rs111724172	chr1:161767856	FCGR2C
rs2333748	chr1:161615545	FCGR2B
rs61801189	chr1:161706626	FCGR2C
rs2045573	chr1:161618714	FCGR2C
rs4657042	chr1:161482519	FCGR3B
rs61801831	chr1:161570662	FCGR2B
rs17416920	chr1:161903651	FCGR3A
rs115032752	chr1:161451117	FCGR2C
rs12752280	chr1:161607376	FCGR2A
rs111971126	chr1:161563695	FCGR3B
rs66888136	chr1:161315277	FCGR2B
rs34754216	chr1:161575537	FCGR3A
rs146653557	chr1:161593121	FCGR2C
rs61801833	chr1:161571302	FCGR2B
rs41271949	chr1:161771811	FCGR2B
rs34540988	chr1:161575760	FCGR3A
rs72708099	chr1:161908876	FCGR2B
rs61802325	chr1:161588096	FCGR3B
rs17415408	chr1:161722804	FCGR2B
rs61804162	chr1:161622834	FCGR2B

rs115264949	chr1:161501931	FCGR3B
rs2880055	chr1:161922364	FCGR2C
rs112335631	chr1:161771048	FCGR2B
rs61801836	chr1:161572759	FCGR2C
rs17416633	chr1:161888268	FCGR3B
rs9427374	chr1:161406055	FCGR2C
rs61802324	chr1:161588079	FCGR2A
rs543438691	chr1:161597123	FCGR3B
rs184018038	chr1:161608312	FCGR2C
rs78154415	chr1:161600648	FCGR2C
rs72706014	chr1:161720348	FCGR2A
rs543438691	chr1:161597123	FCGR2B
rs12040409	chr1:161678181	FCGR2C
rs138010330	chr1:161526186	FCGR2B
rs61802298	chr1:161574739	FCGR2C
rs61803026	chr1:161601117	FCGR2B
rs422670	chr1:161569147	FCGR2B
rs61801238	chr1:161724302	FCGR3B
rs2045572	chr1:161643642	FCGR3B
rs56238128	chr1:161531781	FCGR2B
rs61803026	chr1:161601117	FCGR3B
rs422670	chr1:161569147	FCGR3B
rs72717018	chr1:161450596	FCGR2C
rs61802301	chr1:161575148	FCGR2C
rs142579257	chr1:161643071	FCGR2C
rs74127076	chr1:161600743	FCGR2B
rs10917740	chr1:161680011	FCGR2C
rs34527977	chr1:161596996	FCGR2A
rs77858450	chr1:161700648	FCGR2C
rs111340912	chr1:161580264	FCGR3B
rs61804162	chr1:161622834	FCGR3B
rs77066692	chr1:161564347	FCGR2B
rs111724172	chr1:161767856	FCGR2B
rs2045573	chr1:161618714	FCGR2B
rs1417579	chr1:161661052	FCGR2C
rs77858450	chr1:161700648	FCGR3A
rs12140361	chr1:161321997	FCGR2A
rs61802325	chr1:161588096	FCGR2C
rs66535289	chr1:161289440	FCGR2B
rs61801182	chr1:161699245	FCGR3A
rs111759390	chr1:161922527	FCGR2B
rs7518087	chr1:161477815	FCGR2A
rs17450980	chr1:161908095	FCGR3B
rs7518642	chr1:161607354	FCGR2C
rs112298225	chr1:161907448	FCGR3B
rs114591250	chr1:161905423	FCGR3A
rs17405152	chr1:161680431	FCGR2B
rs17448307	chr1:161791339	FCGR3B
rs112578212	chr1:161915089	FCGR2B
rs7552317	chr1:161479437	FCGR2C

rs408849	chr1:161612339	FCGR2C
rs146617619	chr1:161592477	FCGR2A
rs72706043	chr1:161751864	FCGR2B
rs146423333	chr1:161722607	FCGR2B
rs35046931	chr1:161314112	FCGR2B
rs12749237	chr1:161505961	FCGR2C
rs139529340	chr1:161563553	FCGR3B
rs3845548	chr1:161507447	FCGR2C
rs7518642	chr1:161607354	FCGR2B
rs9427065	chr1:161474960	FCGR2A
rs34907291	chr1:161601538	FCGR2A
rs10919544	chr1:161508762	FCGR2A
rs72700095	chr1:161567188	FCGR2B
rs9427064	chr1:161451638	FCGR2C
rs79654278	chr1:161700647	FCGR2A
rs7526944	chr1:161515951	FCGR3B
rs16832790	chr1:161275942	FCGR3B
rs475807	chr1:161589347	FCGR2C
rs723177	chr1:161651063	FCGR2C
rs905595	chr1:161684889	FCGR3A
rs844	chr1:161647532	FCGR3B
rs10917740	chr1:161680011	FCGR3B
rs35654618	chr1:161573521	FCGR2C
rs111724172	chr1:161767856	FCGR3B
rs6427592	chr1:161408396	FCGR2A
rs867624	chr1:161467836	FCGR3A
rs77066692	chr1:161564347	FCGR3A
rs61801834	chr1:161571748	FCGR2B
rs12402741	chr1:161404253	FCGR2C
rs114591250	chr1:161905423	FCGR3B
rs61803025	chr1:161600590	FCGR2C
rs6427591	chr1:161408368	FCGR3B
rs17417647	chr1:161928613	FCGR2B
rs61801831	chr1:161570662	FCGR3A
rs9427396	chr1:161451892	FCGR2B
rs115827707	chr1:161530106	FCGR2A
rs112045543	chr1:161587760	FCGR2B
rs9427063	chr1:161448240	FCGR2C
rs12043181	chr1:161680790	FCGR2C
rs61802298	chr1:161574739	FCGR3B
rs112298225	chr1:161907448	FCGR2B
rs146653557	chr1:161593121	FCGR2B
rs12044611	chr1:161697546	FCGR2A
rs10919543	chr1:161508616	FCGR2B
rs112896977	chr1:161924114	FCGR2C
rs7526944	chr1:161515951	FCGR2C
rs518377	chr1:161573294	FCGR3B
rs74917046	chr1:161580192	FCGR3B
rs560093485	chr1:161771181	FCGR3B
rs116422836	chr1:161588312	FCGR2B

rs61801829	chr1:161570106	FCGR3B
rs138728964	chr1:161607118	FCGR2A
rs4092450	chr1:161623340	FCGR2C
rs111796652	chr1:161831575	FCGR2B
rs149779887	chr1:161869243	FCGR2B
rs408849	chr1:161612339	FCGR2B
rs61803029	chr1:161601520	FCGR3B
rs113293012	chr1:161919977	FCGR2B
rs75131185	chr1:161488942	FCGR2A
rs146777125	chr1:161571941	FCGR3A
rs12040409	chr1:161678181	FCGR2B
rs1417579	chr1:161661052	FCGR2B
rs12043181	chr1:161680790	FCGR3B
rs7526944	chr1:161515951	FCGR3A
rs61803012	chr1:161594560	FCGR3B
rs61801830	chr1:161570202	FCGR3B
rs12756382	chr1:161607517	FCGR2A
rs112298225	chr1:161907448	FCGR2C
rs72702109	chr1:161575092	FCGR2C
rs9427065	chr1:161474960	FCGR3B
rs7529425	chr1:161479598	FCGR3A
rs61801238	chr1:161724302	FCGR2C
rs61802324	chr1:161588079	FCGR3A
rs111744179	chr1:161776942	FCGR2C
rs74917046	chr1:161580192	FCGR2C
rs72717040	chr1:161487451	FCGR2C
rs55864322	chr1:161306464	FCGR2B
rs422670	chr1:161569147	FCGR2A
rs511278	chr1:161490895	FCGR2B
rs112184925	chr1:161865887	FCGR2B
rs113263422	chr1:161791735	FCGR2B
rs146653557	chr1:161593121	FCGR2A
rs17415505	chr1:161727926	FCGR2C
rs61802299	chr1:161574745	FCGR3A
rs66535289	chr1:161289440	FCGR2C
rs61801822	chr1:161565323	FCGR2C
rs35544618	chr1:161585455	FCGR2C
rs72706039	chr1:161740155	FCGR3A
rs183786059	chr1:161800435	FCGR3B
rs6671753	chr1:161478707	FCGR3B
rs148885671	chr1:161588133	FCGR2B
rs184018038	chr1:161608312	FCGR3B
rs112045543	chr1:161587760	FCGR3A
rs17415505	chr1:161727926	FCGR2B
rs5014880	chr1:161891604	FCGR2B
rs61801832	chr1:161570802	FCGR3B
rs61801235	chr1:161722648	FCGR2A
rs17406901	chr1:161726018	FCGR3A
rs61803025	chr1:161600590	FCGR2B
rs1771576	chr1:161617365	FCGR2A

rs146653557	chr1:161593121	FCGR3A
rs41299300	chr1:161579686	FCGR3B
rs61801822	chr1:161565323	FCGR3B
rs61801187	chr1:161703991	FCGR2C
rs76277413	chr1:161565380	FCGR2B
rs61802316	chr1:161585395	FCGR2C
rs17415408	chr1:161722804	FCGR2C
rs61804161	chr1:161622756	FCGR2B
rs72717009	chr1:161405052	FCGR2C
rs61801244	chr1:161732118	FCGR2C
rs61803012	chr1:161594560	FCGR2C
rs77858450	chr1:161700648	FCGR2A
rs12043179	chr1:161680785	FCGR2B
rs61801242	chr1:161729699	FCGR2C
rs16857520	chr1:161691747	FCGR3A
rs111256263	chr1:161905434	FCGR2A
rs1674757	chr1:161644386	FCGR2B
rs61801182	chr1:161699245	FCGR2A
rs34772855	chr1:161585296	FCGR2C
rs61802298	chr1:161574739	FCGR2B
rs17415505	chr1:161727926	FCGR3B
rs113946660	chr1:161913402	FCGR2B
rs148483621	chr1:161396301	FCGR2C
rs35046931	chr1:161314112	FCGR3A
rs35284289	chr1:161736208	FCGR2A
rs78876532	chr1:161574627	FCGR2A
rs149686267	chr1:161600108	FCGR2B
rs75417510	chr1:161528063	FCGR3B
rs17405152	chr1:161680431	FCGR2C
rs12140361	chr1:161321997	FCGR3B
rs112335631	chr1:161771048	FCGR3B
rs111340912	chr1:161580264	FCGR2C
rs9427402	chr1:161492666	FCGR2B
rs111305724	chr1:161796421	FCGR2B
rs181761989	chr1:161801555	FCGR2B
rs61801829	chr1:161570106	FCGR3A
rs143812595	chr1:161602334	FCGR2C
rs9427047	chr1:161402523	FCGR2C
rs142579257	chr1:161643071	FCGR2B
rs61801832	chr1:161570802	FCGR3A
rs560093485	chr1:161771181	FCGR2B
rs112045543	chr1:161587760	FCGR3B
rs111892839	chr1:161736051	FCGR2A
rs61801831	chr1:161570662	FCGR3B
rs16857520	chr1:161691747	FCGR2C
rs7552317	chr1:161479437	FCGR2B
rs75131185	chr1:161488942	FCGR3A
rs180978155	chr1:161587015	FCGR2C
rs72717018	chr1:161450596	FCGR2B
rs9427064	chr1:161451638	FCGR2B

rs72700090	chr1:161564369	FCGR2C
rs17448307	chr1:161791339	FCGR2C
rs16832790	chr1:161275942	FCGR2B
rs35046931	chr1:161314112	FCGR3B
rs61801832	chr1:161570802	FCGR2A
rs74127076	chr1:161600743	FCGR3A
rs61802301	chr1:161575148	FCGR3A
rs17415408	chr1:161722804	FCGR3B
rs2333748	chr1:161615545	FCGR3A
rs61801829	chr1:161570106	FCGR2B
rs61801834	chr1:161571748	FCGR3A
rs77169596	chr1:161534616	FCGR3A
rs72704062	chr1:161646624	FCGR2A
rs475807	chr1:161589347	FCGR3B
rs17416920	chr1:161903651	FCGR2C
rs77825069	chr1:161517684	FCGR2A
rs61803025	chr1:161600590	FCGR3B
rs4092450	chr1:161623340	FCGR3B
rs60397531	chr1:161704472	FCGR2C
rs78154415	chr1:161600648	FCGR3B
rs61801833	chr1:161571302	FCGR3B
rs150121834	chr1:161589952	FCGR2C
rs61801239	chr1:161725233	FCGR2C
rs9427402	chr1:161492666	FCGR3A
rs72633697	chr1:161659087	FCGR2C
rs61801239	chr1:161725233	FCGR3B
rs61803026	chr1:161601117	FCGR2A
rs150591267	chr1:161666700	FCGR2B
rs9427373	chr1:161404552	FCGR2C
rs112369311	chr1:161580800	FCGR3B
rs72700095	chr1:161567188	FCGR3B
rs146777125	chr1:161571941	FCGR2B
rs61801242	chr1:161729699	FCGR2B
rs61803040	chr1:161612409	FCGR2C
rs61802316	chr1:161585395	FCGR3B
rs146777125	chr1:161571941	FCGR3B
rs72700096	chr1:161567193	FCGR2C
rs72717016	chr1:161450340	FCGR2B
rs61801187	chr1:161703991	FCGR2B
rs7518642	chr1:161607354	FCGR3A
rs12756382	chr1:161607517	FCGR3A
rs61801836	chr1:161572759	FCGR3A
rs12043179	chr1:161680785	FCGR2C
rs35835689	chr1:161575446	FCGR3B
rs9427065	chr1:161474960	FCGR2B
rs867624	chr1:161467836	FCGR2B
rs111384507	chr1:161588872	FCGR2C
rs138728964	chr1:161607118	FCGR3A
rs422670	chr1:161569147	FCGR3A
rs1417579	chr1:161661052	FCGR3B

rs6427591	chr1:161408368	FCGR2A
rs41271949	chr1:161771811	FCGR3B
rs7539053	chr1:161659844	FCGR2C
rs111570995	chr1:161783660	FCGR3B
rs72706043	chr1:161751864	FCGR2C
rs74917046	chr1:161580192	FCGR2B
rs78154415	chr1:161600648	FCGR2B
rs34772855	chr1:161585296	FCGR3B
rs181761989	chr1:161801555	FCGR3B
rs112163369	chr1:161763025	FCGR2C
rs143874519	chr1:161537597	FCGR2C
rs67411091	chr1:161593961	FCGR2A
rs35654618	chr1:161573521	FCGR2B
rs142579257	chr1:161643071	FCGR3B
rs61801833	chr1:161571302	FCGR2A
rs139294219	chr1:161607760	FCGR2C
rs35544618	chr1:161585455	FCGR3B
rs560093485	chr1:161771181	FCGR2C
rs511278	chr1:161490895	FCGR3A
rs17418130	chr1:161936617	FCGR2B
rs17450980	chr1:161908095	FCGR2B
rs150591267	chr1:161666700	FCGR2C
rs41299300	chr1:161579686	FCGR2B
rs61801833	chr1:161571302	FCGR3A
rs59916521	chr1:161661659	FCGR2B
rs12134087	chr1:161678310	FCGR3B
rs518377	chr1:161573294	FCGR3A
rs113726452	chr1:161861509	FCGR2B
rs35835689	chr1:161575446	FCGR2C
rs61801236	chr1:161723387	FCGR2C
rs12040409	chr1:161678181	FCGR3B
rs16859391	chr1:161702383	FCGR2C
rs61801834	chr1:161571748	FCGR3B
rs71632979	chr1:161526396	FCGR2B
rs72717040	chr1:161487451	FCGR2A
rs75417510	chr1:161528063	FCGR2A
rs72700090	chr1:161564369	FCGR2B
rs61801836	chr1:161572759	FCGR2B
rs72708088	chr1:161882188	FCGR2B
rs77825069	chr1:161517684	FCGR3A
rs72702109	chr1:161575092	FCGR3B
rs61801244	chr1:161732118	FCGR2B
rs10919543	chr1:161508616	FCGR2A
rs35835689	chr1:161575446	FCGR2B
rs60397531	chr1:161704472	FCGR2B
rs61802316	chr1:161585395	FCGR2B
rs61801189	chr1:161706626	FCGR2B
rs61801189	chr1:161706626	FCGR3A
rs183786059	chr1:161800435	FCGR2C
rs112572824	chr1:161481594	FCGR3A

rs10917815	chr1:161705870	FCGR2B
rs151061115	chr1:161507448	FCGR2C
rs61803025	chr1:161600590	FCGR3A
rs475807	chr1:161589347	FCGR2B
rs12756382	chr1:161607517	FCGR2B
rs61801236	chr1:161723387	FCGR2B
rs844	chr1:161647532	FCGR2B
rs61803015	chr1:161597986	FCGR2C
rs59916521	chr1:161661659	FCGR3B
rs151061115	chr1:161507448	FCGR2A
rs111744179	chr1:161776942	FCGR3B
rs180978155	chr1:161587015	FCGR3B
rs76277413	chr1:161565380	FCGR2C
rs149779887	chr1:161869243	FCGR2C
rs61801830	chr1:161570202	FCGR2B
rs55871495	chr1:161730973	FCGR2C
rs112289794	chr1:161887647	FCGR2B
rs76583185	chr1:161660394	FCGR2C
rs150494819	chr1:161555169	FCGR2C
rs150591267	chr1:161666700	FCGR3B
rs511278	chr1:161490895	FCGR3B
rs114066064	chr1:161280634	FCGR2C
rs116422836	chr1:161588312	FCGR3A
rs61803029	chr1:161601520	FCGR2B
rs59916521	chr1:161661659	FCGR2C
rs867624	chr1:161467836	FCGR3B
rs72700095	chr1:161567188	FCGR2A
rs61803012	chr1:161594560	FCGR3A
rs12121292	chr1:161650507	FCGR2B
rs61801836	chr1:161572759	FCGR3B
rs143874519	chr1:161537597	FCGR3A
rs112578212	chr1:161915089	FCGR2C
rs61803012	chr1:161594560	FCGR2A
rs34107302	chr1:161585405	FCGR2C
rs723177	chr1:161651063	FCGR2B
rs7539053	chr1:161659844	FCGR2B
rs9427396	chr1:161451892	FCGR3A
rs149686267	chr1:161600108	FCGR3A
rs180978155	chr1:161587015	FCGR2B
rs3949343	chr1:161664764	FCGR3B
rs72706023	chr1:161724248	FCGR3A
rs61801237	chr1:161723391	FCGR3B
rs34772855	chr1:161585296	FCGR2B
rs61803012	chr1:161594560	FCGR2B
rs61801189	chr1:161706626	FCGR3B
rs116835268	chr1:161663334	FCGR2C
rs112896977	chr1:161924114	FCGR3B
rs77825069	chr1:161517684	FCGR2B
rs61801184	chr1:161703434	FCGR2A
rs723177	chr1:161651063	FCGR3B

rs10919544	chr1:161508762	FCGR3B
rs17416920	chr1:161903651	FCGR2A
rs116835268	chr1:161663334	FCGR3B
rs4656309	chr1:161478789	FCGR2A
rs35046931	chr1:161314112	FCGR2A
rs10800573	chr1:161507923	FCGR2A
rs111759390	chr1:161922527	FCGR3B
rs113505621	chr1:161798708	FCGR2B
rs16859391	chr1:161702383	FCGR2B
rs76157571	chr1:161850924	FCGR2B
rs114461997	chr1:161901823	FCGR3B
rs12121292	chr1:161650507	FCGR2C
rs6686673	chr1:161654947	FCGR2B
rs12121021	chr1:161655515	FCGR2B
rs111971126	chr1:161563695	FCGR2B
rs74538320	chr1:161573506	FCGR3B
rs111768204	chr1:161785705	FCGR2C
rs116835268	chr1:161663334	FCGR2B
rs377720202	chr1:161620431	FCGR2C
rs844	chr1:161647532	FCGR2C
rs75131185	chr1:161488942	FCGR3B
rs113263422	chr1:161791735	FCGR2C
rs10919544	chr1:161508762	FCGR2B
rs150121834	chr1:161589952	FCGR3B
rs35544618	chr1:161585455	FCGR2B
rs72706022	chr1:161724126	FCGR3A
rs10917740	chr1:161680011	FCGR3A
rs112335631	chr1:161771048	FCGR2C
rs34754216	chr1:161575537	FCGR2A
rs12746613	chr1:161467041	FCGR2C
rs72700096	chr1:161567193	FCGR2B
rs55864322	chr1:161306464	FCGR3B
rs61802325	chr1:161588096	FCGR2B
rs35983472	chr1:161575767	FCGR2C
rs150121834	chr1:161589952	FCGR2B
rs72702109	chr1:161575092	FCGR2B
rs7552498	chr1:161643662	FCGR2C
rs3845548	chr1:161507447	FCGR2A
rs61802301	chr1:161575148	FCGR2B
rs113062223	chr1:161753505	FCGR2B
rs67418890	chr1:161540856	FCGR2C
rs72717018	chr1:161450596	FCGR3A
rs408849	chr1:161612339	FCGR3B
rs74538320	chr1:161573506	FCGR2C
rs75417510	chr1:161528063	FCGR3A
rs77410877	chr1:161563074	FCGR2C
rs9427374	chr1:161406055	FCGR2B
rs16859391	chr1:161702383	FCGR3B
rs111992131	chr1:161929936	FCGR2B
rs115827707	chr1:161530106	FCGR2B

rs187814751	chr1:161587891	FCGR2C
rs75417510	chr1:161528063	FCGR2B
rs112045543	chr1:161587760	FCGR2A
rs2878011	chr1:161612475	FCGR2C
rs3845548	chr1:161507447	FCGR3A
rs10919544	chr1:161508762	FCGR3A
rs149686267	chr1:161600108	FCGR2A
rs35983472	chr1:161575767	FCGR3B
rs35654618	chr1:161573521	FCGR3B
rs6427591	chr1:161408368	FCGR3A
rs12741391	chr1:161306941	FCGR3B
rs17416424	chr1:161783290	FCGR3B
rs61801823	chr1:161565957	FCGR2A
rs61801236	chr1:161723387	FCGR3B
rs12121021	chr1:161655515	FCGR2C
rs867624	chr1:161467836	FCGR2A
rs186982892	chr1:161304124	FCGR2C
rs72717018	chr1:161450596	FCGR2A
rs377720202	chr1:161620431	FCGR2B
rs113058868	chr1:161747043	FCGR3A
rs10917815	chr1:161705870	FCGR2C
rs66535289	chr1:161289440	FCGR3B
rs5014880	chr1:161891604	FCGR2C
rs55970405	chr1:161617819	FCGR2C
rs61802333	chr1:161589981	FCGR2C
rs3949343	chr1:161664764	FCGR2C
rs61801163	chr1:161686725	FCGR2B
rs10919543	chr1:161508616	FCGR3A
rs112811497	chr1:161802420	FCGR2B
rs61802326	chr1:161588256	FCGR2C
rs61801235	chr1:161722648	FCGR3A
rs2878011	chr1:161612475	FCGR3B
rs111340912	chr1:161580264	FCGR2B
rs184018038	chr1:161608312	FCGR2B
rs61801836	chr1:161572759	FCGR2A
rs150494819	chr1:161555169	FCGR3A
rs61801239	chr1:161725233	FCGR3A
rs5014880	chr1:161891604	FCGR3B
rs12741391	chr1:161306941	FCGR2C
rs12136414	chr1:161678421	FCGR3A
rs61802298	chr1:161574739	FCGR3A
rs12749237	chr1:161505961	FCGR2B
rs111998161	chr1:161871791	FCGR2B
rs111612785	chr1:161890330	FCGR3B
rs76583185	chr1:161660394	FCGR3B
rs72717016	chr1:161450340	FCGR3A
rs72714988	chr1:161334022	FCGR2C
rs2185551	chr1:161662004	FCGR3B
rs138731942	chr1:161569568	FCGR2C
rs34540988	chr1:161575760	FCGR2A

rs34380170	chr1:161575607	FCGR3B
rs9427064	chr1:161451638	FCGR3A
rs61801830	chr1:161570202	FCGR3A
rs905590	chr1:161703398	FCGR2C
rs61801841	chr1:161574653	FCGR3B
rs6686673	chr1:161654947	FCGR2C
rs12136407	chr1:161678376	FCGR3B
rs7526944	chr1:161515951	FCGR2B
rs34107302	chr1:161585405	FCGR2B
rs76277413	chr1:161565380	FCGR3A
rs17450980	chr1:161908095	FCGR2C
rs3949343	chr1:161664764	FCGR2B
rs72704050	chr1:161600591	FCGR2C
rs112896977	chr1:161924114	FCGR3A
rs72714978	chr1:161302353	FCGR2C
rs111340912	chr1:161580264	FCGR3A
rs72633697	chr1:161659087	FCGR3B
rs9427063	chr1:161448240	FCGR2B
rs111305724	chr1:161796421	FCGR2C
rs113726452	chr1:161861509	FCGR2C
rs139294219	chr1:161607760	FCGR3B
rs181761989	chr1:161801555	FCGR2C
rs2878011	chr1:161612475	FCGR2B
rs34472144	chr1:161575743	FCGR3B
rs6694403	chr1:161460210	FCGR2C
rs475807	chr1:161589347	FCGR3A
rs151061115	chr1:161507448	FCGR3A
rs61801237	chr1:161723391	FCGR2B
rs368475173	chr1:161560172	FCGR2C
rs905590	chr1:161703398	FCGR2B
rs76583185	chr1:161660394	FCGR2B
rs184018038	chr1:161608312	FCGR3A
rs112163369	chr1:161763025	FCGR2B
rs518377	chr1:161573294	FCGR2B
rs475807	chr1:161589347	FCGR2A
rs61801242	chr1:161729699	FCGR3B
rs72702109	chr1:161575092	FCGR3A
rs12402741	chr1:161404253	FCGR2B
rs7529425	chr1:161479598	FCGR2A
rs72700095	chr1:161567188	FCGR3A
rs139294219	chr1:161607760	FCGR3A
rs61801822	chr1:161565323	FCGR3A
rs2185551	chr1:161662004	FCGR2B
rs368475173	chr1:161560172	FCGR3A
rs35654618	chr1:161573521	FCGR3A
rs61801163	chr1:161686725	FCGR2C
rs113062223	chr1:161753505	FCGR2C
rs143812595	chr1:161602334	FCGR2B
rs61801822	chr1:161565323	FCGR2B
rs10800573	chr1:161507923	FCGR2C

rs113062223	chr1:161753505	FCGR3B
rs7552498	chr1:161643662	FCGR3B
rs61801831	chr1:161570662	FCGR2A
rs55871495	chr1:161730973	FCGR3A
rs7526944	chr1:161515951	FCGR2A
rs111612785	chr1:161890330	FCGR2C
rs115032752	chr1:161451117	FCGR2B
rs61802326	chr1:161588256	FCGR3B
rs61801239	chr1:161725233	FCGR2B
rs77066692	chr1:161564347	FCGR2A
rs61803026	chr1:161601117	FCGR3A
rs72700090	chr1:161564369	FCGR3B
rs61801841	chr1:161574653	FCGR2C
rs111322113	chr1:161589767	FCGR2C
rs148885671	chr1:161588133	FCGR3A
rs9427373	chr1:161404552	FCGR2B
rs112626262	chr1:161782274	FCGR2C
rs551272312	chr1:161800986	FCGR2B
rs78154415	chr1:161600648	FCGR3A
rs146777125	chr1:161571941	FCGR2A
rs55871495	chr1:161730973	FCGR2B
rs12134087	chr1:161678310	FCGR2C
rs7529425	chr1:161479598	FCGR3B
rs35949692	chr1:161302331	FCGR2C
rs72633697	chr1:161659087	FCGR2B
rs61803040	chr1:161612409	FCGR2B
rs143029017	chr1:161380700	FCGR2C
rs12040409	chr1:161678181	FCGR3A
rs111384507	chr1:161588872	FCGR2B
rs543438691	chr1:161597123	FCGR3A
rs61803015	chr1:161597986	FCGR2B
rs79016857	chr1:161835626	FCGR2B
rs74538320	chr1:161573506	FCGR2B
rs9427396	chr1:161451892	FCGR2A
rs56124956	chr1:161725791	FCGR3A
rs61804184	chr1:161643124	FCGR3B
rs12117530	chr1:161643983	FCGR3B
rs61801834	chr1:161571748	FCGR2A
rs34107302	chr1:161585405	FCGR3B
rs61803040	chr1:161612409	FCGR3B
rs77169596	chr1:161534616	FCGR2B
rs368475173	chr1:161560172	FCGR3B
rs61801244	chr1:161732118	FCGR3B
rs2045572	chr1:161643642	FCGR3A
rs149822268	chr1:161917828	FCGR2B
rs115032752	chr1:161451117	FCGR3B
rs7539053	chr1:161659844	FCGR3B
rs1954172	chr1:161677734	FCGR3A
rs61802325	chr1:161588096	FCGR3A
rs35544618	chr1:161585455	FCGR2A

rs72700096	chr1:161567193	FCGR3B
rs111384507	chr1:161588872	FCGR3B
rs35520307	chr1:161199430	FCGR3B
rs7518642	chr1:161607354	FCGR2A
rs143029017	chr1:161380700	FCGR3A
rs61801829	chr1:161570106	FCGR2A
rs61803015	chr1:161597986	FCGR3B
rs112289794	chr1:161887647	FCGR3B
rs3856196	chr1:161620027	FCGR2C
rs9427402	chr1:161492666	FCGR3B
rs9427390	chr1:161445513	FCGR2C
rs35983472	chr1:161575767	FCGR3A
rs7511868	chr1:161463330	FCGR2C
rs143812595	chr1:161602334	FCGR3A
rs1674757	chr1:161644386	FCGR3A
rs61801237	chr1:161723391	FCGR2C
rs112626262	chr1:161782274	FCGR3B
rs12134087	chr1:161678310	FCGR2B
rs74917046	chr1:161580192	FCGR3A
rs41299300	chr1:161579686	FCGR3A
rs112369311	chr1:161580800	FCGR2C
rs61803015	chr1:161597986	FCGR3A
rs67307769	chr1:161319168	FCGR3B
rs192123799	chr1:161608300	FCGR2B
rs61801822	chr1:161565323	FCGR2A
rs112732063	chr1:161259215	FCGR2C
rs12749237	chr1:161505961	FCGR3B
rs17408026	chr1:161789991	FCGR3B
rs3856196	chr1:161620027	FCGR2B
rs187814751	chr1:161587891	FCGR3B
rs113505621	chr1:161798708	FCGR3B
rs55970405	chr1:161617819	FCGR3B
rs75131185	chr1:161488942	FCGR2B
rs17405152	chr1:161680431	FCGR3A
rs35949692	chr1:161302331	FCGR3B
rs72714978	chr1:161302353	FCGR3B
rs6427592	chr1:161408396	FCGR3B
rs34472144	chr1:161575743	FCGR2C
rs61802326	chr1:161588256	FCGR2B
rs377720202	chr1:161620431	FCGR3B
rs2305972	chr1:161574547	FCGR2B
rs2185551	chr1:161662004	FCGR2C
rs374469827	chr1:161528989	FCGR2C
rs12121021	chr1:161655515	FCGR3B
rs6694403	chr1:161460210	FCGR3B
rs77410877	chr1:161563074	FCGR2B
rs61803040	chr1:161612409	FCGR3A
rs111828362	chr1:161557527	FCGR3B
rs112296661	chr1:161863965	FCGR2B
rs150494819	chr1:161555169	FCGR3B

rs138441127	chr1:161508714	FCGR2C
rs72706043	chr1:161751864	FCGR3B
rs142917083	chr1:161780700	FCGR2C
rs75476984	chr1:161978244	FCGR2B
rs61804161	chr1:161622756	FCGR3A
rs368475173	chr1:161560172	FCGR2B
rs12749237	chr1:161505961	FCGR2A
rs111971126	chr1:161563695	FCGR3A
rs67307769	chr1:161319168	FCGR2B
rs72714996	chr1:161374603	FCGR2C
rs34472144	chr1:161575743	FCGR2B
rs55970405	chr1:161617819	FCGR2B
rs4657090	chr1:161662483	FCGR2B
rs4092450	chr1:161623340	FCGR2B
rs116422836	chr1:161588312	FCGR2A
rs35544618	chr1:161585455	FCGR3A
rs112975798	chr1:161807939	FCGR2B
rs72700090	chr1:161564369	FCGR3A
rs112975798	chr1:161807939	FCGR2C
rs140797462	chr1:161450097	FCGR2C
rs112896977	chr1:161924114	FCGR2A
rs72717016	chr1:161450340	FCGR3B
rs34380170	chr1:161575607	FCGR2C
rs61801841	chr1:161574653	FCGR2B
rs150494819	chr1:161555169	FCGR2B
rs111768204	chr1:161785705	FCGR2B
rs408849	chr1:161612339	FCGR3A
rs10919543	chr1:161508616	FCGR3B
rs72714988	chr1:161334022	FCGR3B
rs17448571	chr1:161807951	FCGR3B
rs77169596	chr1:161534616	FCGR3B
rs139294219	chr1:161607760	FCGR2B
rs2333748	chr1:161615545	FCGR2A
rs12046383	chr1:161707501	FCGR2C
rs17405152	chr1:161680431	FCGR3B
rs186982892	chr1:161304124	FCGR3B
rs71632995	chr1:161542345	FCGR2C
rs61802326	chr1:161588256	FCGR3A
rs12741391	chr1:161306941	FCGR2B
rs77801348	chr1:161563054	FCGR2C
rs111570995	chr1:161783660	FCGR2C
rs9427039	chr1:161390515	FCGR2C
rs61802299	chr1:161574745	FCGR2A
rs67418890	chr1:161540856	FCGR3A
rs72704050	chr1:161600591	FCGR2B
rs17408026	chr1:161789991	FCGR2B
rs114265063	chr1:161662649	FCGR2B
rs143812595	chr1:161602334	FCGR3B
rs369381518	chr1:161542913	FCGR2C
rs111340912	chr1:161580264	FCGR2A

rs2446624	chr1:161619360	FCGR2C
rs9427064	chr1:161451638	FCGR2A
rs111322113	chr1:161589767	FCGR3B
rs151171702	chr1:161566043	FCGR2C
rs10800573	chr1:161507923	FCGR3A
rs115802971	chr1:161601260	FCGR2C
rs61804184	chr1:161643124	FCGR2C
rs111796652	chr1:161831575	FCGR2C
rs144840741	chr1:161523323	FCGR2C
rs61802333	chr1:161589981	FCGR2B
rs17417647	chr1:161928613	FCGR2C
rs35835689	chr1:161575446	FCGR3A
rs111768204	chr1:161785705	FCGR3B
rs112369311	chr1:161580800	FCGR3A
rs6666989	chr1:161288593	FCGR2C
rs142917083	chr1:161780700	FCGR3B
rs17394957	chr1:161313436	FCGR3A
rs115032752	chr1:161451117	FCGR3A
rs905590	chr1:161703398	FCGR3B
rs35983472	chr1:161575767	FCGR2B
rs112811497	chr1:161802420	FCGR3B
rs180978155	chr1:161587015	FCGR2A
rs6427592	chr1:161408396	FCGR3A
rs12136407	chr1:161678376	FCGR2B
rs55871495	chr1:161730973	FCGR3B
rs148483621	chr1:161396301	FCGR2B
rs3845548	chr1:161507447	FCGR2B
rs190884597	chr1:161758757	FCGR3B
rs111384507	chr1:161588872	FCGR3A
rs2880055	chr1:161922364	FCGR3A
rs74127074	chr1:161597263	FCGR2C
rs3856196	chr1:161620027	FCGR3B
rs72700096	chr1:161567193	FCGR3A
rs61804160	chr1:161620088	FCGR3B
rs6686673	chr1:161654947	FCGR3B
rs112369311	chr1:161580800	FCGR2B
rs372149781	chr1:161535231	FCGR2C
rs143029017	chr1:161380700	FCGR3B
rs66535289	chr1:161289440	FCGR2A
rs142917083	chr1:161780700	FCGR2B
rs34085961	chr1:161600994	FCGR2A
rs67775399	chr1:161572352	FCGR3B
rs113062223	chr1:161753505	FCGR3A
rs61801187	chr1:161703991	FCGR3B
rs143596860	chr1:161595729	FCGR3B
rs111440656	chr1:161921760	FCGR2B
rs76157571	chr1:161850924	FCGR2C
rs59916521	chr1:161661659	FCGR3A
rs115802971	chr1:161601260	FCGR3B
rs148465811	chr1:161573300	FCGR2C

rs138731942	chr1:161569568	FCGR3B
rs180978155	chr1:161587015	FCGR3A
rs10800573	chr1:161507923	FCGR3B
rs112298225	chr1:161907448	FCGR3A
rs17452514	chr1:161951076	FCGR2B
rs61802333	chr1:161589981	FCGR3B
rs17418028	chr1:161934951	FCGR2B
rs77825069	chr1:161517684	FCGR3B
rs146423333	chr1:161722607	FCGR3A
rs138731942	chr1:161569568	FCGR2B
rs112805729	chr1:161798658	FCGR2B
rs72706028	chr1:161729439	FCGR3B
rs67307769	chr1:161319168	FCGR2C
rs36059328	chr1:161301817	FCGR3B
rs79515951	chr1:161825446	FCGR3B
rs72700091	chr1:161565775	FCGR3B
rs72708099	chr1:161908876	FCGR2C
rs76277413	chr1:161565380	FCGR2A
rs6686673	chr1:161654947	FCGR3A
rs143989790	chr1:161586980	FCGR3B
rs112626262	chr1:161782274	FCGR2B
rs61809495	chr1:161936486	FCGR3B
rs111322113	chr1:161589767	FCGR3A
rs113428915	chr1:161875881	FCGR2B
rs72717016	chr1:161450340	FCGR2A
rs9427396	chr1:161451892	FCGR3B
rs7511868	chr1:161463330	FCGR2B
rs111305724	chr1:161796421	FCGR3B
rs150494819	chr1:161555169	FCGR2A
rs369381518	chr1:161542913	FCGR3B
rs137903827	chr1:161407141	FCGR2B
rs55864322	chr1:161306464	FCGR3A
rs79631525	chr1:161449152	FCGR2C
rs61803029	chr1:161601520	FCGR3A
rs16859935	chr1:161705072	FCGR2C
rs61809501	chr1:161941818	FCGR2B
rs2305972	chr1:161574547	FCGR2C
rs34380170	chr1:161575607	FCGR2B
rs148465811	chr1:161573300	FCGR3B
rs143596860	chr1:161595729	FCGR2C
rs10753610	chr1:161663822	FCGR2B
rs17408026	chr1:161789991	FCGR2C
rs60397531	chr1:161704472	FCGR3B
rs112163369	chr1:161763025	FCGR3B
rs10753610	chr1:161663822	FCGR2C
rs12136407	chr1:161678376	FCGR2C
rs12117530	chr1:161643983	FCGR2B
rs4092450	chr1:161623340	FCGR3A
rs17416633	chr1:161888268	FCGR2A
rs61809495	chr1:161936486	FCGR2B

rs4657090	chr1:161662483	FCGR3B
rs77109226	chr1:161600631	FCGR3B
rs143989790	chr1:161586980	FCGR2C
rs12749237	chr1:161505961	FCGR3A
rs114384494	chr1:161653553	FCGR3B
rs34472144	chr1:161575743	FCGR3A
rs112975798	chr1:161807939	FCGR3B
rs146808754	chr1:161667659	FCGR3B
rs72714978	chr1:161302353	FCGR2B
rs111612785	chr1:161890330	FCGR3A
rs9427374	chr1:161406055	FCGR3B
rs72633697	chr1:161659087	FCGR3A
rs112572824	chr1:161481594	FCGR3B
rs72704050	chr1:161600591	FCGR3B
rs6694403	chr1:161460210	FCGR2B
rs34107302	chr1:161585405	FCGR3A
rs72717040	chr1:161487451	FCGR2B
rs7539053	chr1:161659844	FCGR3A
rs61802846	chr1:161473872	FCGR3B
rs72717009	chr1:161405052	FCGR2B
rs9427064	chr1:161451638	FCGR3B
rs2446624	chr1:161619360	FCGR3A
rs9427063	chr1:161448240	FCGR2A
rs114265063	chr1:161662649	FCGR2C
rs74127078	chr1:161601322	FCGR2C
rs72717040	chr1:161487451	FCGR3A
rs35519204	chr1:161573326	FCGR2B
rs408849	chr1:161612339	FCGR2A
rs111570995	chr1:161783660	FCGR2B
rs77109226	chr1:161600631	FCGR2C
rs12117530	chr1:161643983	FCGR2C
rs115802971	chr1:161601260	FCGR2B
rs72704072	chr1:161675889	FCGR3B
rs35949692	chr1:161302331	FCGR2B
rs72706028	chr1:161729439	FCGR2B
rs192123799	chr1:161608300	FCGR2C
rs34772855	chr1:161585296	FCGR2A
rs111828362	chr1:161557527	FCGR2B
rs61804162	chr1:161622834	FCGR3A
rs112184925	chr1:161865887	FCGR2C
rs12121292	chr1:161650507	FCGR3B
rs35519204	chr1:161573326	FCGR2C
rs7540959	chr1:161612228	FCGR2C
rs17416424	chr1:161783290	FCGR2B
rs7512140	chr1:161463600	FCGR2C
rs113946660	chr1:161913402	FCGR2C
rs77410877	chr1:161563074	FCGR3B
rs77801348	chr1:161563054	FCGR3B
rs74538320	chr1:161573506	FCGR2A
rs61804209	chr1:161666632	FCGR2B

rs61801841	chr1:161574653	FCGR3A
rs67295569	chr1:161597820	FCGR2C
rs67775399	chr1:161572352	FCGR2C
rs17418130	chr1:161936617	FCGR2C
rs61801237	chr1:161723391	FCGR3A
rs66888136	chr1:161315277	FCGR3B
rs9427047	chr1:161402523	FCGR2B
rs74127076	chr1:161600743	FCGR2A
rs61802326	chr1:161588256	FCGR2A
rs2446624	chr1:161619360	FCGR2B
rs114461997	chr1:161901823	FCGR2B
rs34772855	chr1:161585296	FCGR3A
rs17416633	chr1:161888268	FCGR3A
rs114066064	chr1:161280634	FCGR2B
rs1674785	chr1:161570356	FCGR2C
rs190884597	chr1:161758757	FCGR2B
rs61804209	chr1:161666632	FCGR3B
rs16859391	chr1:161702383	FCGR3A
rs113248291	chr1:161845532	FCGR2B
rs12746613	chr1:161467041	FCGR2B
rs79601960	chr1:161666121	FCGR2B
rs113505621	chr1:161798708	FCGR2C
rs187814751	chr1:161587891	FCGR2B
rs35835689	chr1:161575446	FCGR2A
rs36059328	chr1:161301817	FCGR2C
rs17394957	chr1:161313436	FCGR3B
rs10917717	chr1:161672051	FCGR2B
rs61801234	chr1:161716941	FCGR3B
rs1674785	chr1:161570356	FCGR3B
rs114265063	chr1:161662649	FCGR3B
rs7552498	chr1:161643662	FCGR2B
rs77801348	chr1:161563054	FCGR2B
rs72717018	chr1:161450596	FCGR3B
rs111828362	chr1:161557527	FCGR2C
rs67418890	chr1:161540856	FCGR3B
rs2446624	chr1:161619360	FCGR3B
rs143029017	chr1:161380700	FCGR2A
rs143874519	chr1:161537597	FCGR2B
rs111322113	chr1:161589767	FCGR2B
rs374469827	chr1:161528989	FCGR3B
rs2085696	chr1:161569029	FCGR2C
rs146423333	chr1:161722607	FCGR2A
rs34508679	chr1:161574285	FCGR3B
rs4657090	chr1:161662483	FCGR2C
rs114771574	chr1:161443692	FCGR2B
rs187814751	chr1:161587891	FCGR3A
rs112689091	chr1:161791601	FCGR3B
rs7554873	chr1:161612232	FCGR2C
rs72704072	chr1:161675889	FCGR2B
rs55725332	chr1:161611023	FCGR2C

rs1340976	chr1:161661488	FCGR2B
rs138441127	chr1:161508714	FCGR3A
rs77109226	chr1:161600631	FCGR2B
rs12046383	chr1:161707501	FCGR3B
rs41299300	chr1:161579686	FCGR2A
rs7512140	chr1:161463600	FCGR2B
rs77169596	chr1:161534616	FCGR2A
rs112401004	chr1:161912728	FCGR2B
rs72700091	chr1:161565775	FCGR2C
rs67418890	chr1:161540856	FCGR2B
rs151061115	chr1:161507448	FCGR2B
rs551272312	chr1:161800986	FCGR2C
rs113726452	chr1:161861509	FCGR3B
rs1674785	chr1:161570356	FCGR3A
rs6666989	chr1:161288593	FCGR3B
rs16857520	chr1:161691747	FCGR2A
rs67295569	chr1:161597820	FCGR3B
rs72706034	chr1:161732311	FCGR2B
rs115531571	chr1:161790546	FCGR3B
rs61801830	chr1:161570202	FCGR2A
rs180719335	chr1:161589658	FCGR2C
rs35519204	chr1:161573326	FCGR3B
rs61804208	chr1:161661410	FCGR2B
rs72714988	chr1:161334022	FCGR2B
rs143989790	chr1:161586980	FCGR2B
rs2878011	chr1:161612475	FCGR3A
rs369381518	chr1:161542913	FCGR3A
rs111828362	chr1:161557527	FCGR3A
rs74127074	chr1:161597263	FCGR3B
rs12046383	chr1:161707501	FCGR2B
rs72700091	chr1:161565775	FCGR2B
rs138441127	chr1:161508714	FCGR2B
rs146808754	chr1:161667659	FCGR2B
rs112369311	chr1:161580800	FCGR2A
rs12135523	chr1:161678253	FCGR2C
rs17415303	chr1:161721266	FCGR2A
rs2085696	chr1:161569029	FCGR3B
rs56091027	chr1:161615394	FCGR3B
rs74127078	chr1:161601322	FCGR3B
rs150121834	chr1:161589952	FCGR3A
rs79515951	chr1:161825446	FCGR2B
rs97349	chr1:161615705	FCGR2C
rs137903827	chr1:161407141	FCGR2C
rs12131017	chr1:161615369	FCGR3B
rs17416424	chr1:161783290	FCGR2C
rs146140071	chr1:161649698	FCGR3B
rs111971126	chr1:161563695	FCGR2A
rs4657086	chr1:161639284	FCGR2B
rs61804187	chr1:161649988	FCGR3B
rs56124956	chr1:161725791	FCGR2A

rs111796652	chr1:161831575	FCGR3B
rs151061115	chr1:161507448	FCGR3B
rs76025404	chr1:161407518	FCGR2C
rs72702109	chr1:161575092	FCGR2A
rs111504845	chr1:161565478	FCGR2C
rs5014880	chr1:161891604	FCGR3A
rs16859935	chr1:161705072	FCGR3B
rs35139848	chr1:161597176	FCGR2C
rs76025404	chr1:161407518	FCGR2B
rs115531571	chr1:161790546	FCGR2B
rs112175965	chr1:161779939	FCGR2B
rs146808754	chr1:161667659	FCGR2C
rs10753610	chr1:161663822	FCGR3B
rs9427048	chr1:161404550	FCGR2C
rs113428915	chr1:161875881	FCGR2C
rs7511868	chr1:161463330	FCGR2A
rs150019709	chr1:161589586	FCGR2B
rs199688803	chr1:161603447	FCGR3B
rs7511868	chr1:161463330	FCGR3A
rs61809496	chr1:161938877	FCGR2B
rs78154415	chr1:161600648	FCGR2A
rs114384494	chr1:161653553	FCGR2C
rs143029017	chr1:161380700	FCGR2B
rs61802333	chr1:161589981	FCGR3A
rs116835268	chr1:161663334	FCGR3A
rs76025404	chr1:161407518	FCGR3B
rs3845548	chr1:161507447	FCGR3B
rs113293012	chr1:161919977	FCGR3B
rs2305972	chr1:161574547	FCGR3B
rs551272312	chr1:161800986	FCGR3B
rs532090372	chr1:161600275	FCGR2B
rs71519213	chr1:161572329	FCGR2C
rs139529340	chr1:161563553	FCGR2A
rs74538320	chr1:161573506	FCGR3A
rs181761989	chr1:161801555	FCGR3A
rs72714988	chr1:161334022	FCGR2A
rs77801348	chr1:161563054	FCGR2A
rs112572824	chr1:161481594	FCGR2A
rs151171702	chr1:161566043	FCGR2B
rs79827863	chr1:161836559	FCGR3B
rs61801163	chr1:161686725	FCGR3B
rs61802316	chr1:161585395	FCGR3A
rs61804184	chr1:161643124	FCGR2B
rs72708088	chr1:161882188	FCGR2C
rs10800573	chr1:161507923	FCGR2B
rs143596860	chr1:161595729	FCGR3A
rs3883934	chr1:161600831	FCGR2C
rs145143330	chr1:161778882	FCGR2B
rs61804187	chr1:161649988	FCGR2B
rs143874519	chr1:161537597	FCGR2A

rs74127078	chr1:161601322	FCGR2B
rs6694403	chr1:161460210	FCGR3A
rs112811497	chr1:161802420	FCGR2C
rs9427047	chr1:161402523	FCGR3A
rs149822268	chr1:161917828	FCGR3B
rs61804205	chr1:161653736	FCGR2B
rs199688803	chr1:161603447	FCGR2C
rs11578979	chr1:161658657	FCGR2B
rs76277413	chr1:161565380	FCGR3B
rs56159502	chr1:161618544	FCGR3B
rs138731942	chr1:161569568	FCGR3A
rs112578212	chr1:161915089	FCGR3B
rs61801242	chr1:161729699	FCGR3A
rs2045573	chr1:161618714	FCGR2A
rs34380170	chr1:161575607	FCGR3A
rs61802846	chr1:161473872	FCGR2C
rs61804208	chr1:161661410	FCGR3B
rs9427039	chr1:161390515	FCGR2B
rs111570995	chr1:161783660	FCGR3A
rs114384494	chr1:161653553	FCGR2B
rs139474963	chr1:161619757	FCGR2C
rs180719335	chr1:161589658	FCGR2B
rs71632995	chr1:161542345	FCGR3B
rs551272312	chr1:161800986	FCGR3A
rs12135523	chr1:161678253	FCGR3B
rs137903827	chr1:161407141	FCGR3B
rs72717009	chr1:161405052	FCGR3A
rs112674212	chr1:161913466	FCGR2B
rs61809501	chr1:161941818	FCGR2C
rs72706028	chr1:161729439	FCGR2C
rs113979070	chr1:161935328	FCGR2B
rs80300109	chr1:160977607	FCGR2C
rs34508679	chr1:161574285	FCGR2B
rs7529425	chr1:161479598	FCGR2B
rs97349	chr1:161615705	FCGR3B
rs61804205	chr1:161653736	FCGR3B
rs61348372	chr1:161617731	FCGR2C
rs150019709	chr1:161589586	FCGR2C
rs111998161	chr1:161871791	FCGR3B
rs12746613	chr1:161467041	FCGR3A
rs1256288	chr1:161654842	FCGR2B
rs35520307	chr1:161199430	FCGR3A
rs9427390	chr1:161445513	FCGR2B
rs3883934	chr1:161600831	FCGR3B
rs61348372	chr1:161617731	FCGR3B
rs148483621	chr1:161396301	FCGR3A
rs61803025	chr1:161600590	FCGR2A
rs2045573	chr1:161618714	FCGR3A
rs72714996	chr1:161374603	FCGR2B
rs148241977	chr1:161674069	FCGR3B

rs143874519	chr1:161537597	FCGR3B
rs17409485	chr1:161853119	FCGR2B
rs372149781	chr1:161535231	FCGR3A
rs34508679	chr1:161574285	FCGR2C
rs112572824	chr1:161481594	FCGR2C
rs374469827	chr1:161528989	FCGR3A
rs142579257	chr1:161643071	FCGR3A
rs12746613	chr1:161467041	FCGR2A
rs112699037	chr1:161941039	FCGR2B
rs183786059	chr1:161800435	FCGR3A
rs61801244	chr1:161732118	FCGR3A
rs186982892	chr1:161304124	FCGR2B
rs17417198	chr1:161910746	FCGR2B
rs148465811	chr1:161573300	FCGR2B
rs35139848	chr1:161597176	FCGR3B
rs72700096	chr1:161567193	FCGR2A
rs61801234	chr1:161716941	FCGR2C
rs149822268	chr1:161917828	FCGR2C
rs199688803	chr1:161603447	FCGR2B
rs112732063	chr1:161259215	FCGR2B
rs532090372	chr1:161600275	FCGR2C
rs111384507	chr1:161588872	FCGR2A
rs4656329	chr1:161667657	FCGR2B
rs112296661	chr1:161863965	FCGR2C
rs2880055	chr1:161922364	FCGR2A
rs532090372	chr1:161600275	FCGR3B
rs61802846	chr1:161473872	FCGR3A
rs57978522	chr1:161608159	FCGR3B
rs41271949	chr1:161771811	FCGR3A
rs17418028	chr1:161934951	FCGR3B
rs61804206	chr1:161658820	FCGR2B
rs12741391	chr1:161306941	FCGR2A
rs146140071	chr1:161649698	FCGR2B
rs61804207	chr1:161660717	FCGR3B
rs67775399	chr1:161572352	FCGR2B
rs77109226	chr1:161600631	FCGR3A
rs112859576	chr1:161900802	FCGR2B
rs148241977	chr1:161674069	FCGR2B
rs116205652	chr1:161659458	FCGR3B
rs76025404	chr1:161407518	FCGR3A
rs12043181	chr1:161680790	FCGR3A
rs75476984	chr1:161978244	FCGR2C
rs77410877	chr1:161563074	FCGR3A
rs72702132	chr1:161597205	FCGR2C
rs79016857	chr1:161835626	FCGR2C
rs9427048	chr1:161404550	FCGR2B
rs114461997	chr1:161901823	FCGR2C
rs147640330	chr1:161536757	FCGR2C
rs192123799	chr1:161608300	FCGR3B
rs111440656	chr1:161921760	FCGR2C

rs111440656	chr1:161921760	FCGR3B
rs115032752	chr1:161451117	FCGR2A
rs55725332	chr1:161611023	FCGR3A
rs12135523	chr1:161678253	FCGR2B
rs77032824	chr1:161567441	FCGR2C
rs190884597	chr1:161758757	FCGR2C
rs532984690	chr1:161597122	FCGR3B
rs148885671	chr1:161588133	FCGR2A
rs36059328	chr1:161301817	FCGR2B
rs111504845	chr1:161565478	FCGR2B
rs57978522	chr1:161608159	FCGR2B
rs144840741	chr1:161523323	FCGR3B
rs4656329	chr1:161667657	FCGR3B
rs55725332	chr1:161611023	FCGR3B
rs79016857	chr1:161835626	FCGR3B
rs7552498	chr1:161643662	FCGR3A
rs142966355	chr1:161622469	FCGR2C
rs61801187	chr1:161703991	FCGR3A
rs12136407	chr1:161678376	FCGR3A
rs10917717	chr1:161672051	FCGR2C
rs61804209	chr1:161666632	FCGR2C
rs113060703	chr1:161736271	FCGR2A
rs12402741	chr1:161404253	FCGR2A
rs844	chr1:161647532	FCGR3A
rs116205652	chr1:161659458	FCGR2B
rs140797462	chr1:161450097	FCGR2B
rs9427374	chr1:161406055	FCGR3A
rs10917815	chr1:161705870	FCGR3B
rs55970405	chr1:161617819	FCGR3A
rs144605559	chr1:160925140	FCGR2C
rs12134087	chr1:161678310	FCGR3A
rs61801002	chr1:161537845	FCGR2C
rs61801234	chr1:161716941	FCGR2B
rs77801348	chr1:161563054	FCGR3A
rs17414989	chr1:161706148	FCGR2B
rs61809495	chr1:161936486	FCGR2C
rs61804187	chr1:161649988	FCGR2C
rs72704072	chr1:161675889	FCGR2C
rs61802306	chr1:161577656	FCGR2B
rs12141690	chr1:161367646	FCGR2C
rs72706023	chr1:161724248	FCGR2A
rs181468352	chr1:161567035	FCGR2B
rs55725332	chr1:161611023	FCGR2B
rs143596860	chr1:161595729	FCGR2B
rs72704050	chr1:161600591	FCGR3A
rs17415408	chr1:161722804	FCGR3A
rs111998161	chr1:161871791	FCGR2C
rs12043179	chr1:161680785	FCGR3B
rs150591267	chr1:161666700	FCGR3A
rs113248291	chr1:161845532	FCGR2C

rs56159502	chr1:161618544	FCGR2C
rs2878011	chr1:161612475	FCGR2A
rs150019709	chr1:161589586	FCGR3B
rs150392443	chr1:161598897	FCGR2B
rs34820280	chr1:161574289	FCGR2B
rs78897192	chr1:161595079	FCGR3B
rs3883934	chr1:161600831	FCGR2B
rs56159502	chr1:161618544	FCGR2B
rs6666989	chr1:161288593	FCGR2B
rs7554873	chr1:161612232	FCGR3B
rs140797462	chr1:161450097	FCGR3A
rs181468352	chr1:161567035	FCGR3B
rs9427374	chr1:161406055	FCGR2A
rs17400517	chr1:161485258	FCGR3A
rs16859935	chr1:161705072	FCGR2B
rs181468352	chr1:161567035	FCGR2C
rs74127074	chr1:161597263	FCGR2B
rs115531571	chr1:161790546	FCGR2C
rs111918621	chr1:161927277	FCGR2B
rs7554873	chr1:161612232	FCGR2B
rs17394957	chr1:161313436	FCGR2C
rs532725665	chr1:161608311	FCGR2B
rs147640330	chr1:161536757	FCGR3B
rs55864322	chr1:161306464	FCGR2A
rs144605559	chr1:160925140	FCGR2B
rs1954172	chr1:161677734	FCGR2A
rs72717040	chr1:161487451	FCGR3B
rs72708088	chr1:161882188	FCGR3B
rs554414140	chr1:161584472	FCGR2B
rs72706034	chr1:161732311	FCGR3B
rs3856196	chr1:161620027	FCGR3A
rs79601960	chr1:161666121	FCGR3B
rs12746613	chr1:161467041	FCGR3B
rs144840741	chr1:161523323	FCGR3A
rs112674212	chr1:161913466	FCGR3B
rs3949343	chr1:161664764	FCGR3A
rs112572824	chr1:161481594	FCGR2B
rs142137670	chr1:161561233	FCGR3B
rs17418389	chr1:161941185	FCGR2B
rs144840741	chr1:161523323	FCGR2B
rs6683306	chr1:161660205	FCGR2B
rs12131017	chr1:161615369	FCGR2B
rs113293012	chr1:161919977	FCGR2C
rs4657086	chr1:161639284	FCGR2C
rs61804162	chr1:161622834	FCGR2A
rs1674785	chr1:161570356	FCGR2B
rs35200803	chr1:161395025	FCGR2C
rs112626262	chr1:161782274	FCGR3A
rs374469827	chr1:161528989	FCGR2B
rs61804160	chr1:161620088	FCGR2B

rs180719335	chr1:161589658	FCGR3B
rs79631525	chr1:161449152	FCGR2B
rs148241977	chr1:161674069	FCGR2C
rs111724172	chr1:161767856	FCGR2A
rs151171702	chr1:161566043	FCGR3A
rs7512140	chr1:161463600	FCGR2A
rs74651366	chr1:161649482	FCGR3B
rs532984690	chr1:161597122	FCGR2B
rs4657086	chr1:161639284	FCGR3B
rs74127079	chr1:161601419	FCGR2C
rs17418130	chr1:161936617	FCGR3B
rs2169052	chr1:161557153	FCGR3B
rs79631525	chr1:161449152	FCGR3A
rs147640330	chr1:161536757	FCGR3A
rs12131017	chr1:161615369	FCGR2C
rs115130696	chr1:161601259	FCGR2B
rs532984690	chr1:161597122	FCGR2C
rs142137670	chr1:161561233	FCGR2C
rs723177	chr1:161651063	FCGR3A
rs72706043	chr1:161751864	FCGR3A
rs12402741	chr1:161404253	FCGR3A
rs9427390	chr1:161445513	FCGR2A
rs112699037	chr1:161941039	FCGR2C
rs7540959	chr1:161612228	FCGR3B
rs34612244	chr1:161598277	FCGR2C
rs76052159	chr1:161567985	FCGR2C
rs35654618	chr1:161573521	FCGR2A
rs72706022	chr1:161724126	FCGR2A
rs61801238	chr1:161724302	FCGR3A
rs60397531	chr1:161704472	FCGR3A
rs61801163	chr1:161686725	FCGR3A
rs9427063	chr1:161448240	FCGR3A
rs2085696	chr1:161569029	FCGR2B
rs61804208	chr1:161661410	FCGR2C
rs76583185	chr1:161660394	FCGR3A
rs35139848	chr1:161597176	FCGR2B
rs2185551	chr1:161662004	FCGR3A
rs114771574	chr1:161443692	FCGR2C
rs1417579	chr1:161661052	FCGR3A
rs77032824	chr1:161567441	FCGR3B
rs7529425	chr1:161479598	FCGR2C
rs2169052	chr1:161557153	FCGR2C
rs72714988	chr1:161334022	FCGR3A
rs150392443	chr1:161598897	FCGR2C
rs12741391	chr1:161306941	FCGR3A
rs61804160	chr1:161620088	FCGR2C
rs61801161	chr1:161680517	FCGR3B
rs78897192	chr1:161595079	FCGR2C
rs112363639	chr1:161588852	FCGR3B
rs532090372	chr1:161600275	FCGR3A

rs554414140	chr1:161584472	FCGR3B
rs12046383	chr1:161707501	FCGR3A
rs17415505	chr1:161727926	FCGR3A
rs188672904	chr1:161541122	FCGR2C
rs180719335	chr1:161589658	FCGR3A
rs17399583	chr1:161339575	FCGR2C
rs79631525	chr1:161449152	FCGR2A
rs61802298	chr1:161574739	FCGR2A
rs71632995	chr1:161542345	FCGR3A
rs74651366	chr1:161649482	FCGR2C
rs1340976	chr1:161661488	FCGR3B
rs150392443	chr1:161598897	FCGR3B
rs518377	chr1:161573294	FCGR2A
rs61802329	chr1:161589625	FCGR2C
rs74560530	chr1:161941387	FCGR2B
rs115802971	chr1:161601260	FCGR3A
rs61803028	chr1:161601294	FCGR3B
rs112184925	chr1:161865887	FCGR3B
rs36059328	chr1:161301817	FCGR3A
rs72714978	chr1:161302353	FCGR3A
rs149779887	chr1:161869243	FCGR3B
rs111744179	chr1:161776942	FCGR3A
rs112363639	chr1:161588852	FCGR2C
rs72706034	chr1:161732311	FCGR2C
rs377720202	chr1:161620431	FCGR3A
rs12043179	chr1:161680785	FCGR3A
rs532725665	chr1:161608311	FCGR3B
rs79601960	chr1:161666121	FCGR2C
rs56091027	chr1:161615394	FCGR2B
rs6679543	chr1:161288597	FCGR3B
rs61802334	chr1:161590276	FCGR2B
rs72700090	chr1:161564369	FCGR2A
rs72704075	chr1:161675993	FCGR2B
rs11586976	chr1:161673931	FCGR2B
rs4656329	chr1:161667657	FCGR2C
rs9427390	chr1:161445513	FCGR3B
rs61801161	chr1:161680517	FCGR2C
rs61801837	chr1:161573126	FCGR2C
rs112578212	chr1:161915089	FCGR3A
rs12121021	chr1:161655515	FCGR3A
rs112298225	chr1:161907448	FCGR2A
rs61804184	chr1:161643124	FCGR3A
rs7540959	chr1:161612228	FCGR2B
rs78861213	chr1:161603102	FCGR3B
rs199705513	chr1:161602517	FCGR2B
rs74127079	chr1:161601419	FCGR3B
rs76052159	chr1:161567985	FCGR2B
rs6683306	chr1:161660205	FCGR3B
rs57924693	chr1:161977606	FCGR2B
rs76157571	chr1:161850924	FCGR3B

rs72702132	chr1:161597205	FCGR2B
rs114771574	chr1:161443692	FCGR3A
rs112674212	chr1:161913466	FCGR2C
rs12136414	chr1:161678421	FCGR2A
rs61801837	chr1:161573126	FCGR3B
rs111759390	chr1:161922527	FCGR2C
rs111759390	chr1:161922527	FCGR3A
rs4656327	chr1:161664582	FCGR2B
rs61802846	chr1:161473872	FCGR2B
rs372149781	chr1:161535231	FCGR2A
rs374469827	chr1:161528989	FCGR2A
rs114461997	chr1:161901823	FCGR3A
rs139474963	chr1:161619757	FCGR3B
rs61801161	chr1:161680517	FCGR2B
rs61804205	chr1:161653736	FCGR2C
rs560093485	chr1:161771181	FCGR2A
rs74127078	chr1:161601322	FCGR3A
rs72633696	chr1:161657895	FCGR2B
rs137903827	chr1:161407141	FCGR3A
rs188672904	chr1:161541122	FCGR3B
rs9427401	chr1:161492174	FCGR2C
rs4657090	chr1:161662483	FCGR3A
rs72708099	chr1:161908876	FCGR3B
rs72702132	chr1:161597205	FCGR3B
rs61803028	chr1:161601294	FCGR2B
rs9427392	chr1:161448690	FCGR2C
rs2045574	chr1:161618741	FCGR3B
rs113910081	chr1:161865409	FCGR2B
rs61809496	chr1:161938877	FCGR2C
rs66888136	chr1:161315277	FCGR3A
rs35519204	chr1:161573326	FCGR3A
rs112175965	chr1:161779939	FCGR3B
rs139474963	chr1:161619757	FCGR2B
rs111918621	chr1:161927277	FCGR3B
rs61801826	chr1:161569197	FCGR2C
rs12121292	chr1:161650507	FCGR3A
rs17452514	chr1:161951076	FCGR2C
rs61804207	chr1:161660717	FCGR2B
rs11578979	chr1:161658657	FCGR2C
rs143989790	chr1:161586980	FCGR3A
rs17400517	chr1:161485258	FCGR2C
rs560093485	chr1:161771181	FCGR3A
rs2305972	chr1:161574547	FCGR3A
rs112363639	chr1:161588852	FCGR3A
rs61803040	chr1:161612409	FCGR2A
rs9427390	chr1:161445513	FCGR3A
rs78861213	chr1:161603102	FCGR2C
rs192123799	chr1:161608300	FCGR3A
rs11578979	chr1:161658657	FCGR3B
rs189314279	chr1:161948452	FCGR2B

rs112859576	chr1:161900802	FCGR3B
rs112142198	chr1:161568861	FCGR2C
rs61801002	chr1:161537845	FCGR2B
rs9427401	chr1:161492174	FCGR3A
rs56091027	chr1:161615394	FCGR2C
rs142964320	chr1:161597756	FCGR3B
rs67295569	chr1:161597820	FCGR2B
rs142964320	chr1:161597756	FCGR2C
rs78861213	chr1:161603102	FCGR2B
rs61802329	chr1:161589625	FCGR2B
rs72717009	chr1:161405052	FCGR2A
rs57978522	chr1:161608159	FCGR2C
rs61802334	chr1:161590276	FCGR3B
rs67307769	chr1:161319168	FCGR3A
rs78897192	chr1:161595079	FCGR2B
rs113910081	chr1:161865409	FCGR3B
rs6694403	chr1:161460210	FCGR2A
rs554414140	chr1:161584472	FCGR2C
rs61804206	chr1:161658820	FCGR2C
rs142966355	chr1:161622469	FCGR2B
rs77032824	chr1:161567441	FCGR3A
rs97349	chr1:161615705	FCGR2B
rs139294219	chr1:161607760	FCGR2A
rs112363639	chr1:161588852	FCGR2B
rs71632995	chr1:161542345	FCGR2B
rs112689091	chr1:161791601	FCGR2B
rs113428915	chr1:161875881	FCGR3B
rs10753610	chr1:161663822	FCGR3A
rs61802316	chr1:161585395	FCGR2A
rs76052159	chr1:161567985	FCGR3B
rs12117530	chr1:161643983	FCGR3A
rs61801245	chr1:161732305	FCGR2B
rs72717009	chr1:161405052	FCGR3B
rs72714978	chr1:161302353	FCGR2A
rs146140071	chr1:161649698	FCGR2C
rs111504845	chr1:161565478	FCGR3B
rs112805729	chr1:161798658	FCGR3B
rs6671332	chr1:161672450	FCGR2B
rs35200803	chr1:161395025	FCGR2B
rs67295569	chr1:161597820	FCGR3A
rs17417647	chr1:161928613	FCGR3B
rs35989200	chr1:161574304	FCGR3B
rs543438691	chr1:161597123	FCGR2A
rs186982892	chr1:161304124	FCGR3A
rs71519213	chr1:161572329	FCGR3B
rs79515951	chr1:161825446	FCGR2C
rs61802334	chr1:161590276	FCGR2C
rs6671332	chr1:161672450	FCGR3B
rs74127079	chr1:161601419	FCGR2B
rs34472144	chr1:161575743	FCGR2A

rs77032824	chr1:161567441	FCGR2B
rs145831350	chr1:161600344	FCGR2C
rs17414989	chr1:161706148	FCGR2C
rs9427063	chr1:161448240	FCGR3B
rs138441127	chr1:161508714	FCGR3B
rs36021445	chr1:161585646	FCGR2C
rs17450980	chr1:161908095	FCGR3A
rs145143330	chr1:161778882	FCGR3B
rs111992131	chr1:161929936	FCGR3B
rs111612785	chr1:161890330	FCGR2A
rs114771574	chr1:161443692	FCGR3B
rs67418890	chr1:161540856	FCGR2A
rs34612244	chr1:161598277	FCGR3B
rs113946660	chr1:161913402	FCGR3B
rs148483621	chr1:161396301	FCGR3B
rs115782472	chr1:161838205	FCGR3B
rs199705513	chr1:161602517	FCGR2C
rs12121292	chr1:161650507	FCGR2A
rs372149781	chr1:161535231	FCGR2B
rs17448307	chr1:161791339	FCGR2A
rs112335631	chr1:161771048	FCGR3A
rs61809501	chr1:161941818	FCGR3B
rs146808754	chr1:161667659	FCGR3A
rs35989200	chr1:161574304	FCGR2B
rs17448307	chr1:161791339	FCGR3A
rs34612244	chr1:161598277	FCGR3A
rs34508679	chr1:161574285	FCGR3A
rs80300109	chr1:160977607	FCGR2B
rs2045574	chr1:161618741	FCGR2B
rs114265063	chr1:161662649	FCGR3A
rs72704075	chr1:161675993	FCGR2C
rs61801236	chr1:161723387	FCGR3A
rs34612244	chr1:161598277	FCGR2B
rs72700091	chr1:161565775	FCGR3A
rs34107302	chr1:161585405	FCGR2A
rs116205652	chr1:161659458	FCGR2C
rs61801160	chr1:161673858	FCGR2C
rs113910081	chr1:161865409	FCGR2C
rs10917717	chr1:161672051	FCGR3B
rs905590	chr1:161703398	FCGR3A
rs79631525	chr1:161449152	FCGR3B
rs2446624	chr1:161619360	FCGR2A
rs61802306	chr1:161577656	FCGR2C
rs150019709	chr1:161589586	FCGR3A
rs184018038	chr1:161608312	FCGR2A
rs2169052	chr1:161557153	FCGR2B
rs71519213	chr1:161572329	FCGR2B
rs61802329	chr1:161589625	FCGR3B
rs12402741	chr1:161404253	FCGR3B
rs35949692	chr1:161302331	FCGR2A

rs111724172	chr1:161767856	FCGR3A
rs199705513	chr1:161602517	FCGR3B
rs372149781	chr1:161535231	FCGR3B
rs112805729	chr1:161798658	FCGR2C
rs6679543	chr1:161288597	FCGR2C
rs61804206	chr1:161658820	FCGR3B
rs146797688	chr1:161664448	FCGR3B
rs368475173	chr1:161560172	FCGR2A
rs371355868	chr1:161569342	FCGR3B
rs36021445	chr1:161585646	FCGR2B
rs9427373	chr1:161404552	FCGR2A
rs9427047	chr1:161402523	FCGR2A
rs7540959	chr1:161612228	FCGR3A
rs115782472	chr1:161838205	FCGR2B
rs72714996	chr1:161374603	FCGR3B
rs12141690	chr1:161367646	FCGR3A
rs6683306	chr1:161660205	FCGR2C
rs142964320	chr1:161597756	FCGR2B
rs16859935	chr1:161705072	FCGR3A
rs61801841	chr1:161574653	FCGR2A
rs114771574	chr1:161443692	FCGR2A
rs74124990	chr1:161466494	FCGR2C
rs74127074	chr1:161597263	FCGR3A
rs138441127	chr1:161508714	FCGR2A
rs371355868	chr1:161569342	FCGR2C
rs142966355	chr1:161622469	FCGR3B
rs35139848	chr1:161597176	FCGR3A
rs7554873	chr1:161612232	FCGR3A
rs12141690	chr1:161367646	FCGR2B
rs61801002	chr1:161537845	FCGR3A
rs17418028	chr1:161934951	FCGR2C
rs147640330	chr1:161536757	FCGR2B
rs112689091	chr1:161791601	FCGR2C
rs72704075	chr1:161675993	FCGR3B
rs4628483	chr1:161958189	FCGR2B
rs113979070	chr1:161935328	FCGR3B
rs6671332	chr1:161672450	FCGR2C
rs146797688	chr1:161664448	FCGR2B
rs113263422	chr1:161791735	FCGR3A
rs12405558	chr1:161526978	FCGR3A
rs187814751	chr1:161587891	FCGR2A
rs142137670	chr1:161561233	FCGR3A
rs532725665	chr1:161608311	FCGR2C
rs7512140	chr1:161463600	FCGR3A
rs9427394	chr1:161450175	FCGR2C
rs56779916	chr1:161222047	FCGR2C
rs71519213	chr1:161572329	FCGR3A
rs12405558	chr1:161526978	FCGR2C
rs1256288	chr1:161654842	FCGR2C
rs4092450	chr1:161623340	FCGR2A

rs112142198	chr1:161568861	FCGR3B
rs145831350	chr1:161600344	FCGR2B
rs61348372	chr1:161617731	FCGR2B
rs17414989	chr1:161706148	FCGR3B
rs111828362	chr1:161557527	FCGR2A
rs9427401	chr1:161492174	FCGR2A
rs145831350	chr1:161600344	FCGR3B
rs151171702	chr1:161566043	FCGR3B
rs111504845	chr1:161565478	FCGR3A
rs17394957	chr1:161313436	FCGR2B
rs56383975	chr1:161403306	FCGR2B
rs61802325	chr1:161588096	FCGR2A
rs137903827	chr1:161407141	FCGR2A
rs61802333	chr1:161589981	FCGR2A
rs112296661	chr1:161863965	FCGR3B
rs111918621	chr1:161927277	FCGR2C
rs17406901	chr1:161726018	FCGR2A
rs10494359	chr1:161463875	FCGR2B
rs61804160	chr1:161620088	FCGR3A
rs6679543	chr1:161288597	FCGR2B
rs149822268	chr1:161917828	FCGR3A
rs34820280	chr1:161574289	FCGR2C
rs10917717	chr1:161672051	FCGR3A
rs56383975	chr1:161403306	FCGR2C
rs61803029	chr1:161601520	FCGR2A
rs61804210	chr1:161670826	FCGR3B
rs142579257	chr1:161643071	FCGR2A
rs61801234	chr1:161716941	FCGR3A
rs369381518	chr1:161542913	FCGR2B
rs113726452	chr1:161861509	FCGR2A
rs6665610	chr1:161641383	FCGR2B
rs4656330	chr1:161668463	FCGR3B
rs35989200	chr1:161574304	FCGR2C
rs17415505	chr1:161727926	FCGR2A
rs10917815	chr1:161705870	FCGR3A
rs114591250	chr1:161905423	FCGR2A
rs79827863	chr1:161836559	FCGR2C
rs150591267	chr1:161666700	FCGR2A
rs150937010	chr1:161844725	FCGR2B
rs7511868	chr1:161463330	FCGR3B
rs112975798	chr1:161807939	FCGR3A
rs35949692	chr1:161302331	FCGR3A
rs1340976	chr1:161661488	FCGR2C
rs7525363	chr1:161660208	FCGR2B
rs1256288	chr1:161654842	FCGR3A
rs112142198	chr1:161568861	FCGR2B
rs61803028	chr1:161601294	FCGR2C
rs4656327	chr1:161664582	FCGR3B
rs844	chr1:161647532	FCGR2A
rs111440656	chr1:161921760	FCGR3A

rs79827863	chr1:161836559	FCGR2B
rs9427047	chr1:161402523	FCGR3B
rs72633696	chr1:161657895	FCGR2C
rs17405152	chr1:161680431	FCGR2A
rs61802306	chr1:161577656	FCGR3B
rs77931302	chr1:161943946	FCGR2B
rs114384494	chr1:161653553	FCGR3A
rs17409485	chr1:161853119	FCGR2C
rs61801186	chr1:161703690	FCGR3B
rs4657092	chr1:161665413	FCGR3B
rs61801189	chr1:161706626	FCGR2A
rs4656330	chr1:161668463	FCGR2B
rs115130696	chr1:161601259	FCGR3B
rs4657092	chr1:161665413	FCGR2B
rs17448571	chr1:161807951	FCGR2B
rs9427392	chr1:161448690	FCGR2B
rs56077197	chr1:161731254	FCGR2B
rs36021445	chr1:161585646	FCGR3B
rs142137670	chr1:161561233	FCGR2B
rs2045572	chr1:161643642	FCGR2A
rs199688803	chr1:161603447	FCGR3A
rs9427394	chr1:161450175	FCGR2B
rs56779916	chr1:161222047	FCGR2B
rs12722986	chr1:161465419	FCGR2C
rs111768204	chr1:161785705	FCGR3A
rs17448571	chr1:161807951	FCGR2C
rs60397531	chr1:161704472	FCGR2A
rs74651366	chr1:161649482	FCGR2B
rs61802846	chr1:161473872	FCGR2A
rs112175965	chr1:161779939	FCGR2C
rs143524589	chr1:161484069	FCGR2C
rs61804161	chr1:161622756	FCGR2A
rs74917046	chr1:161580192	FCGR2A
rs188672904	chr1:161541122	FCGR2B
rs56077197	chr1:161731254	FCGR3B
rs61801002	chr1:161537845	FCGR3B
rs145143330	chr1:161778882	FCGR2C
rs2045574	chr1:161618741	FCGR2C
rs111992131	chr1:161929936	FCGR2C
rs150392443	chr1:161598897	FCGR3A
rs61801185	chr1:161703659	FCGR2B
rs61801826	chr1:161569197	FCGR2B
rs61801160	chr1:161673858	FCGR2B
rs17417198	chr1:161910746	FCGR2C
rs1256288	chr1:161654842	FCGR3B
rs61801238	chr1:161724302	FCGR2A
rs61801186	chr1:161703690	FCGR2B
rs148465811	chr1:161573300	FCGR3A
rs34820280	chr1:161574289	FCGR3B
rs112401004	chr1:161912728	FCGR2C

rs189314279	chr1:161948452	FCGR3B
rs61801186	chr1:161703690	FCGR2C
rs61804207	chr1:161660717	FCGR2C
rs150138577	chr1:161850153	FCGR2B
rs61801837	chr1:161573126	FCGR2B
rs74127079	chr1:161601419	FCGR3A
rs72633696	chr1:161657895	FCGR3B
rs61801825	chr1:161568885	FCGR2C
rs74124990	chr1:161466494	FCGR3B
rs148241977	chr1:161674069	FCGR3A
rs61802329	chr1:161589625	FCGR3A
rs11586976	chr1:161673931	FCGR2C
rs112184925	chr1:161865887	FCGR3A
rs143812595	chr1:161602334	FCGR2A
rs67775399	chr1:161572352	FCGR3A
rs17417198	chr1:161910746	FCGR3B
rs72700091	chr1:161565775	FCGR2A
rs554414140	chr1:161584472	FCGR3A
rs61804187	chr1:161649988	FCGR3A
rs142966355	chr1:161622469	FCGR3A
rs9427392	chr1:161448690	FCGR3B
rs17452514	chr1:161951076	FCGR3B
rs17414989	chr1:161706148	FCGR3A
rs61809500	chr1:161941091	FCGR2B
rs150121834	chr1:161589952	FCGR2A
rs111992131	chr1:161929936	FCGR3A
rs9427401	chr1:161492174	FCGR2B
rs12722986	chr1:161465419	FCGR2B
rs2085696	chr1:161569029	FCGR3A
rs112699037	chr1:161941039	FCGR3B
rs61801825	chr1:161568885	FCGR2B
rs17400517	chr1:161485258	FCGR2A
rs17394957	chr1:161313436	FCGR2A
rs61801825	chr1:161568885	FCGR3B
rs61801826	chr1:161569197	FCGR3B
rs188672904	chr1:161541122	FCGR3A
rs9427401	chr1:161492174	FCGR3B
rs11578979	chr1:161658657	FCGR3A
rs61801160	chr1:161673858	FCGR3B
rs6665610	chr1:161641383	FCGR3B
rs34820280	chr1:161574289	FCGR3A
rs72706028	chr1:161729439	FCGR3A
rs9427048	chr1:161404550	FCGR3A
rs9427046	chr1:161402258	FCGR2B
rs61802306	chr1:161577656	FCGR3A
rs140391508	chr1:160913403	FCGR2B
rs4657086	chr1:161639284	FCGR3A
rs9427394	chr1:161450175	FCGR3A
rs139474963	chr1:161619757	FCGR3A
rs140797462	chr1:161450097	FCGR3B

rs72706035	chr1:161732627	FCGR2B
rs6679543	chr1:161288597	FCGR3A
rs72714996	chr1:161374603	FCGR3A
rs112401004	chr1:161912728	FCGR3B
rs10917740	chr1:161680011	FCGR2A
rs6666989	chr1:161288593	FCGR3A
rs61809495	chr1:161936486	FCGR3A
rs17400517	chr1:161485258	FCGR2B
rs72706035	chr1:161732627	FCGR3B
rs148483621	chr1:161396301	FCGR2A
rs113248291	chr1:161845532	FCGR3B
rs371355868	chr1:161569342	FCGR3A
rs371355868	chr1:161569342	FCGR2B
rs66535289	chr1:161289440	FCGR3A
rs17416424	chr1:161783290	FCGR3A
rs113263422	chr1:161791735	FCGR2A
rs55970405	chr1:161617819	FCGR2A
rs150248941	chr1:161447535	FCGR2B
rs17418389	chr1:161941185	FCGR2C
rs74124990	chr1:161466494	FCGR2B
rs140578039	chr1:161358226	FCGR2C
rs10494359	chr1:161463875	FCGR2C
rs199705513	chr1:161602517	FCGR3A
rs17399583	chr1:161339575	FCGR2B
rs199688803	chr1:161603447	FCGR2A
rs36021445	chr1:161585646	FCGR3A
rs6666989	chr1:161288593	FCGR2A
rs146188788	chr1:161445364	FCGR2B
rs56077197	chr1:161731254	FCGR2C
rs112289794	chr1:161887647	FCGR2C
rs7525363	chr1:161660208	FCGR3B
rs79515951	chr1:161825446	FCGR3A
rs7552498	chr1:161643662	FCGR2A
rs140797462	chr1:161450097	FCGR2A
rs74127054	chr1:161589245	FCGR2C
rs9427046	chr1:161402258	FCGR2C
rs61804206	chr1:161658820	FCGR3A
rs10737488	chr1:161619362	FCGR2B
rs56091027	chr1:161615394	FCGR3A
rs57924693	chr1:161977606	FCGR2C
rs74124990	chr1:161466494	FCGR3A
rs147936938	chr1:161668736	FCGR3B
rs76052159	chr1:161567985	FCGR3A
rs61809498	chr1:161940670	FCGR2B
rs74127054	chr1:161589245	FCGR2B
rs7512140	chr1:161463600	FCGR3B
rs181468352	chr1:161567035	FCGR3A
rs190884597	chr1:161758757	FCGR3A
rs115130696	chr1:161601259	FCGR2C
rs532725665	chr1:161608311	FCGR3A

rs72708099	chr1:161908876	FCGR3A
rs369381518	chr1:161542913	FCGR2A
rs61801236	chr1:161723387	FCGR2A
rs532984690	chr1:161597122	FCGR3A
rs57978522	chr1:161608159	FCGR3A
rs7525363	chr1:161660208	FCGR2C
rs112732063	chr1:161259215	FCGR3B
rs9427039	chr1:161390515	FCGR3A
rs61802328	chr1:161589326	FCGR2C
rs74127054	chr1:161589245	FCGR3A
rs10494359	chr1:161463875	FCGR3B
rs150937010	chr1:161844725	FCGR2C
rs61802328	chr1:161589326	FCGR2B
rs74127054	chr1:161589245	FCGR3B
rs112859576	chr1:161900802	FCGR2C
rs61802301	chr1:161575148	FCGR2A
rs113293012	chr1:161919977	FCGR3A
rs180719335	chr1:161589658	FCGR2A
rs4656330	chr1:161668463	FCGR2C
rs142964320	chr1:161597756	FCGR3A
rs78897192	chr1:161595079	FCGR3A
rs12135523	chr1:161678253	FCGR3A
rs61801185	chr1:161703659	FCGR3B
rs114066064	chr1:161280634	FCGR3B
rs72633697	chr1:161659087	FCGR2A
rs143524589	chr1:161484069	FCGR2B
rs111796652	chr1:161831575	FCGR3A
rs3883934	chr1:161600831	FCGR3A
rs61809500	chr1:161941091	FCGR3B
rs140578039	chr1:161358226	FCGR2B
rs11586976	chr1:161673931	FCGR3B
rs147936938	chr1:161668736	FCGR2B
rs114066064	chr1:161280634	FCGR3A
rs113910081	chr1:161865409	FCGR3A
rs143524589	chr1:161484069	FCGR3B
rs74651366	chr1:161649482	FCGR3A
rs74127078	chr1:161601322	FCGR2A
rs56976800	chr1:161665374	FCGR2B
rs72702132	chr1:161597205	FCGR3A
rs10494359	chr1:161463875	FCGR3A
rs12131017	chr1:161615369	FCGR3A
rs145831350	chr1:161600344	FCGR3A
rs76025404	chr1:161407518	FCGR2A
rs41271949	chr1:161771811	FCGR2A
rs12405558	chr1:161526978	FCGR2B
rs35200803	chr1:161395025	FCGR3B
rs112289794	chr1:161887647	FCGR3A
rs17418389	chr1:161941185	FCGR3B
rs61804209	chr1:161666632	FCGR3A
rs72706035	chr1:161732627	FCGR2C

rs1340976	chr1:161661488	FCGR3A
rs140391508	chr1:160913403	FCGR2C
rs2169052	chr1:161557153	FCGR3A
rs112142198	chr1:161568861	FCGR3A
rs149779887	chr1:161869243	FCGR3A
rs61804210	chr1:161670826	FCGR2B
rs72704072	chr1:161675889	FCGR3A
rs111322113	chr1:161589767	FCGR2A
rs77410877	chr1:161563074	FCGR2A
rs61801837	chr1:161573126	FCGR3A
rs9427373	chr1:161404552	FCGR3B
rs143524589	chr1:161484069	FCGR2A
rs113979070	chr1:161935328	FCGR2C
rs189314279	chr1:161948452	FCGR2C
rs12043179	chr1:161680785	FCGR2A
rs147640330	chr1:161536757	FCGR2A
rs61801826	chr1:161569197	FCGR3A
rs78861213	chr1:161603102	FCGR3A
rs6671332	chr1:161672450	FCGR3A
rs61801160	chr1:161673858	FCGR3A
rs146140071	chr1:161649698	FCGR3A
rs17448571	chr1:161807951	FCGR3A
rs9427394	chr1:161450175	FCGR3B
rs61804207	chr1:161660717	FCGR3A
rs112163369	chr1:161763025	FCGR3A
rs7535475	chr1:161463586	FCGR2C
rs17400517	chr1:161485258	FCGR3B
rs72704075	chr1:161675993	FCGR3A
rs186982892	chr1:161304124	FCGR2A
rs146188788	chr1:161445364	FCGR2C
rs61801185	chr1:161703659	FCGR2C
rs12117530	chr1:161643983	FCGR2A
rs146797688	chr1:161664448	FCGR2C
rs61802328	chr1:161589326	FCGR3B
rs17409485	chr1:161853119	FCGR3B
rs115782472	chr1:161838205	FCGR3A
rs56976800	chr1:161665374	FCGR3B
rs150248941	chr1:161447535	FCGR3A
rs12405558	chr1:161526978	FCGR3B
rs66888136	chr1:161315277	FCGR2A
rs17450980	chr1:161908095	FCGR2A
rs112335631	chr1:161771048	FCGR2A
rs114066064	chr1:161280634	FCGR2A
rs74567013	chr1:161977236	FCGR2B
rs113979070	chr1:161935328	FCGR3A
rs6665610	chr1:161641383	FCGR2C
rs150937010	chr1:161844725	FCGR3B
rs199705513	chr1:161602517	FCGR2A
rs10737488	chr1:161619362	FCGR2C
rs61804211	chr1:161671305	FCGR3B

rs61801161	chr1:161680517	FCGR3A
rs111402007	chr1:161600188	FCGR2B
rs7535475	chr1:161463586	FCGR2B
rs5014880	chr1:161891604	FCGR2A
rs79601960	chr1:161666121	FCGR3A
rs114931935	chr1:161659800	FCGR3B
rs75476984	chr1:161978244	FCGR3B
rs111787169	chr1:161900150	FCGR2B
rs111402007	chr1:161600188	FCGR2C
rs61803028	chr1:161601294	FCGR3A
rs116205652	chr1:161659458	FCGR3A
rs97349	chr1:161615705	FCGR3A
rs183849087	chr1:161843083	FCGR2B
rs61348372	chr1:161617731	FCGR3A
rs12722986	chr1:161465419	FCGR3B
rs112805729	chr1:161798658	FCGR3A
rs112578212	chr1:161915089	FCGR2A
rs147936938	chr1:161668736	FCGR2C
rs74560530	chr1:161941387	FCGR3B
rs9427048	chr1:161404550	FCGR3B
rs113248291	chr1:161845532	FCGR3A
rs74567013	chr1:161977236	FCGR3B
rs2045574	chr1:161618741	FCGR3A
rs61804208	chr1:161661410	FCGR3A
rs61802328	chr1:161589326	FCGR3A
rs61803015	chr1:161597986	FCGR2A
rs56383975	chr1:161403306	FCGR3B
rs12405558	chr1:161526978	FCGR2A
rs61809496	chr1:161938877	FCGR3B
rs10494359	chr1:161463875	FCGR2A
rs61801245	chr1:161732305	FCGR3B
rs6683306	chr1:161660205	FCGR3A
rs138731942	chr1:161569568	FCGR2A
rs113505621	chr1:161798708	FCGR3A
rs61804210	chr1:161670826	FCGR2C
rs113428915	chr1:161875881	FCGR3A
rs2185551	chr1:161662004	FCGR2A
rs4628483	chr1:161958189	FCGR2C
rs67307769	chr1:161319168	FCGR2A
rs200837054	chr1:161577203	FCGR2B
rs115802971	chr1:161601260	FCGR2A
rs112811497	chr1:161802420	FCGR3A
rs67295569	chr1:161597820	FCGR2A
rs17415408	chr1:161722804	FCGR2A
rs76157571	chr1:161850924	FCGR3A
rs61804210	chr1:161670826	FCGR3A
rs4657092	chr1:161665413	FCGR2C
rs61801185	chr1:161703659	FCGR3A
rs115130696	chr1:161601259	FCGR3A
rs6665610	chr1:161641383	FCGR3A

rs36059328	chr1:161301817	FCGR2A
rs143524589	chr1:161484069	FCGR3A
rs56077197	chr1:161731254	FCGR3A
rs72704050	chr1:161600591	FCGR2A
rs4628483	chr1:161958189	FCGR3B
rs74124990	chr1:161466494	FCGR2A
rs112296661	chr1:161863965	FCGR3A
rs144840741	chr1:161523323	FCGR2A
rs72714996	chr1:161374603	FCGR2A
rs72633696	chr1:161657895	FCGR3A
rs114931935	chr1:161659800	FCGR2B
rs17450717	chr1:161899635	FCGR2B
rs74560530	chr1:161941387	FCGR2C
rs61801825	chr1:161568885	FCGR3A
rs4656327	chr1:161664582	FCGR2C
rs11586976	chr1:161673931	FCGR3A
rs12141690	chr1:161367646	FCGR3B
rs142917083	chr1:161780700	FCGR3A
rs61804205	chr1:161653736	FCGR3A
rs57924693	chr1:161977606	FCGR3B
rs80300109	chr1:160977607	FCGR3B
rs61809498	chr1:161940670	FCGR3B
rs61801239	chr1:161725233	FCGR2A
rs181761989	chr1:161801555	FCGR2A
rs111305724	chr1:161796421	FCGR3A
rs150138577	chr1:161850153	FCGR2C
rs9427373	chr1:161404552	FCGR3A
rs9427048	chr1:161404550	FCGR2A
rs146188788	chr1:161445364	FCGR3B
rs56159502	chr1:161618544	FCGR3A
rs1417579	chr1:161661052	FCGR2A
rs184915469	chr1:161879915	FCGR2B
rs1674785	chr1:161570356	FCGR2A
rs75476984	chr1:161978244	FCGR3A
rs111787169	chr1:161900150	FCGR2C
rs112699037	chr1:161941039	FCGR3A
rs17452514	chr1:161951076	FCGR3A
rs112674212	chr1:161913466	FCGR3A
rs200837054	chr1:161577203	FCGR3A
rs9427372	chr1:161399919	FCGR2C
rs56383975	chr1:161403306	FCGR3A
rs74127074	chr1:161597263	FCGR2A
rs76157571	chr1:161850924	FCGR2A
rs183849087	chr1:161843083	FCGR2C
rs4656330	chr1:161668463	FCGR3A
rs112401004	chr1:161912728	FCGR3A
rs145143330	chr1:161778882	FCGR3A
rs112975798	chr1:161807939	FCGR2A
rs115782472	chr1:161838205	FCGR2C
rs184915469	chr1:161879915	FCGR3B

rs9427392	chr1:161448690	FCGR3A
rs34380170	chr1:161575607	FCGR2A
rs61809501	chr1:161941818	FCGR3A
rs112732063	chr1:161259215	FCGR3A
rs17417198	chr1:161910746	FCGR3A
rs17450717	chr1:161899635	FCGR3B
rs10737488	chr1:161619362	FCGR3B
rs61804211	chr1:161671305	FCGR2B
rs12134087	chr1:161678310	FCGR2A
rs112163369	chr1:161763025	FCGR2A
rs150248941	chr1:161447535	FCGR2C
rs12043181	chr1:161680790	FCGR2A
rs74567013	chr1:161977236	FCGR2C
rs377720202	chr1:161620431	FCGR2A
rs72706035	chr1:161732627	FCGR3A
rs140809349	chr1:161849495	FCGR2B
rs9427372	chr1:161399919	FCGR2B
rs74328646	chr1:161851909	FCGR2B
rs72706043	chr1:161751864	FCGR2A
rs12136407	chr1:161678376	FCGR2A
rs7535475	chr1:161463586	FCGR3A
rs61804184	chr1:161643124	FCGR2A
rs112104785	chr1:161851785	FCGR2B
rs61801244	chr1:161732118	FCGR2A
rs17418028	chr1:161934951	FCGR3A
rs150138577	chr1:161850153	FCGR3B
rs114461997	chr1:161901823	FCGR2A
rs1674757	chr1:161644386	FCGR2A
rs59916521	chr1:161661659	FCGR2A
rs113062223	chr1:161753505	FCGR2A
rs17418389	chr1:161941185	FCGR3A
rs35989200	chr1:161574304	FCGR3A
rs10737488	chr1:161619362	FCGR3A
rs111918621	chr1:161927277	FCGR3A
rs61802334	chr1:161590276	FCGR3A
rs74328646	chr1:161851909	FCGR3B
rs6686673	chr1:161654947	FCGR2A
rs9427394	chr1:161450175	FCGR2A
rs61801002	chr1:161537845	FCGR2A
rs17408026	chr1:161789991	FCGR3A
rs77109226	chr1:161600631	FCGR2A
rs112175965	chr1:161779939	FCGR3A
rs4656327	chr1:161664582	FCGR3A
rs4628483	chr1:161958189	FCGR3A
rs12040409	chr1:161678181	FCGR2A
rs34612244	chr1:161598277	FCGR2A
rs61801237	chr1:161723391	FCGR2A
rs111787169	chr1:161900150	FCGR3B
rs72708088	chr1:161882188	FCGR3A
rs115531571	chr1:161790546	FCGR3A

rs56779916	chr1:161222047	FCGR3B
rs200837054	chr1:161577203	FCGR2C
rs112689091	chr1:161791601	FCGR3A
rs111402007	chr1:161600188	FCGR3B
rs112859576	chr1:161900802	FCGR3A
rs905595	chr1:161684889	FCGR2A
rs71632995	chr1:161542345	FCGR2A
rs77931302	chr1:161943946	FCGR2C
rs142964320	chr1:161597756	FCGR2A
rs142137670	chr1:161561233	FCGR2A
rs147936938	chr1:161668736	FCGR3A
rs113726452	chr1:161861509	FCGR3A
rs9427039	chr1:161390515	FCGR3B
rs111787169	chr1:161900150	FCGR3A
rs183849087	chr1:161843083	FCGR3B
rs4656329	chr1:161667657	FCGR3A
rs9427392	chr1:161448690	FCGR2A
rs2305972	chr1:161574547	FCGR2A
rs111744179	chr1:161776942	FCGR2A
rs67775399	chr1:161572352	FCGR2A
rs61801837	chr1:161573126	FCGR2A
rs17399583	chr1:161339575	FCGR3B
rs7525363	chr1:161660208	FCGR3A
rs191320140	chr1:160868347	FCGR2B
rs61801245	chr1:161732305	FCGR2C
rs61802307	chr1:161577668	FCGR2B
rs34508679	chr1:161574285	FCGR2A
rs35200803	chr1:161395025	FCGR3A
rs77931302	chr1:161943946	FCGR3B
rs150248941	chr1:161447535	FCGR3B
rs35519204	chr1:161573326	FCGR2A
rs554414140	chr1:161584472	FCGR2A
rs72706034	chr1:161732311	FCGR3A
rs200837054	chr1:161577203	FCGR3B
rs77032824	chr1:161567441	FCGR2A
rs12722986	chr1:161465419	FCGR3A
rs150019709	chr1:161589586	FCGR2A
rs17408026	chr1:161789991	FCGR2A
rs3949343	chr1:161664764	FCGR2A
rs61809500	chr1:161941091	FCGR2C
rs140809349	chr1:161849495	FCGR3B
rs61809498	chr1:161940670	FCGR2C
rs9427046	chr1:161402258	FCGR3A
rs61802329	chr1:161589625	FCGR2A
rs111759390	chr1:161922527	FCGR2A
rs36021445	chr1:161585646	FCGR2A
rs61809498	chr1:161940670	FCGR3A
rs61801186	chr1:161703690	FCGR3A
rs61804211	chr1:161671305	FCGR2C
rs61801826	chr1:161569197	FCGR2A

rs55871495	chr1:161730973	FCGR2A
rs188672904	chr1:161541122	FCGR2A
rs74560530	chr1:161941387	FCGR3A
rs146188788	chr1:161445364	FCGR3A
rs7535475	chr1:161463586	FCGR3B
rs112732063	chr1:161259215	FCGR2A
rs12141690	chr1:161367646	FCGR2A
rs148465811	chr1:161573300	FCGR2A
rs10917815	chr1:161705870	FCGR2A
rs140809349	chr1:161849495	FCGR2C
rs371355868	chr1:161569342	FCGR2A
rs150248941	chr1:161447535	FCGR2A
rs12722986	chr1:161465419	FCGR2A
rs17450717	chr1:161899635	FCGR2C
rs9427046	chr1:161402258	FCGR3B
rs35983472	chr1:161575767	FCGR2A
rs6679543	chr1:161288597	FCGR2A
rs79827863	chr1:161836559	FCGR3A
rs112184925	chr1:161865887	FCGR2A
rs17399583	chr1:161339575	FCGR3A
rs71519213	chr1:161572329	FCGR2A
rs113946660	chr1:161913402	FCGR3A
rs111402007	chr1:161600188	FCGR3A
rs7539053	chr1:161659844	FCGR2A
rs532725665	chr1:161608311	FCGR2A
rs143989790	chr1:161586980	FCGR2A
rs181468352	chr1:161567035	FCGR2A
rs114931935	chr1:161659800	FCGR2C
rs79016857	chr1:161835626	FCGR3A
rs74328646	chr1:161851909	FCGR2C
rs61809496	chr1:161938877	FCGR3A
rs9427372	chr1:161399919	FCGR3B
rs111504845	chr1:161565478	FCGR2A
rs146797688	chr1:161664448	FCGR3A
rs192123799	chr1:161608300	FCGR2A
rs111998161	chr1:161871791	FCGR3A
rs56976800	chr1:161665374	FCGR2C
rs151171702	chr1:161566043	FCGR2A
rs150937010	chr1:161844725	FCGR3A
rs17418130	chr1:161936617	FCGR3A
rs143596860	chr1:161595729	FCGR2A
rs61801245	chr1:161732305	FCGR3A
rs9427372	chr1:161399919	FCGR3A
rs76052159	chr1:161567985	FCGR2A
rs61801163	chr1:161686725	FCGR2A
rs7535475	chr1:161463586	FCGR2A
rs12121021	chr1:161655515	FCGR2A
rs61804206	chr1:161658820	FCGR2A
rs61802307	chr1:161577668	FCGR2C
rs61802306	chr1:161577656	FCGR2A

rs4657092	chr1:161665413	FCGR3A
rs34820280	chr1:161574289	FCGR2A
rs184915469	chr1:161879915	FCGR2C
rs149779887	chr1:161869243	FCGR2A
rs56383975	chr1:161403306	FCGR2A
rs184915469	chr1:161879915	FCGR3A
rs61801242	chr1:161729699	FCGR2A
rs55725332	chr1:161611023	FCGR2A
rs16859935	chr1:161705072	FCGR2A
rs72714967	chr1:161291227	FCGR3B
rs17418130	chr1:161936617	FCGR2A
rs12046383	chr1:161707501	FCGR2A
rs140578039	chr1:161358226	FCGR3B
rs183849087	chr1:161843083	FCGR3A
rs113248291	chr1:161845532	FCGR2A
rs114265063	chr1:161662649	FCGR2A
rs146808754	chr1:161667659	FCGR2A
rs35200803	chr1:161395025	FCGR2A
rs76583185	chr1:161660394	FCGR2A
rs7554873	chr1:161612232	FCGR2A
rs146188788	chr1:161445364	FCGR2A
rs111305724	chr1:161796421	FCGR2A
rs9427046	chr1:161402258	FCGR2A
rs191320140	chr1:160868347	FCGR2C
rs6665610	chr1:161641383	FCGR2A
rs189314279	chr1:161948452	FCGR3A
rs116835268	chr1:161663334	FCGR2A
rs78897192	chr1:161595079	FCGR2A
rs4301614	chr1:161681847	FCGR3B
rs183786059	chr1:161800435	FCGR2A
rs115531571	chr1:161790546	FCGR2A
rs61802307	chr1:161577668	FCGR3A
rs140578039	chr1:161358226	FCGR3A
rs72714967	chr1:161291227	FCGR2A
rs61804187	chr1:161649988	FCGR2A
rs61809500	chr1:161941091	FCGR3A
rs145831350	chr1:161600344	FCGR2A
rs140809349	chr1:161849495	FCGR3A
rs72714967	chr1:161291227	FCGR2B
rs2045574	chr1:161618741	FCGR2A
rs61804211	chr1:161671305	FCGR3A
rs2085696	chr1:161569029	FCGR2A
rs17414989	chr1:161706148	FCGR2A
rs114931935	chr1:161659800	FCGR3A
rs7540959	chr1:161612228	FCGR2A
rs72708099	chr1:161908876	FCGR2A
rs61801187	chr1:161703991	FCGR2A
rs139474963	chr1:161619757	FCGR2A
rs97349	chr1:161615705	FCGR2A
rs114384494	chr1:161653553	FCGR2A

rs723177	chr1:161651063	FCGR2A
rs905590	chr1:161703398	FCGR2A
rs4301614	chr1:161681847	FCGR2B
rs61801825	chr1:161568885	FCGR2A
rs17409485	chr1:161853119	FCGR3A
rs111570995	chr1:161783660	FCGR2A
rs111768204	chr1:161785705	FCGR2A
rs56779916	chr1:161222047	FCGR3A
rs76731254	chr1:161876466	FCGR3B
rs150138577	chr1:161850153	FCGR3A
rs16859391	chr1:161702383	FCGR2A
rs77931302	chr1:161943946	FCGR3A
rs11586976	chr1:161673931	FCGR2A
rs74328646	chr1:161851909	FCGR3A
rs3856196	chr1:161620027	FCGR2A
rs148241977	chr1:161674069	FCGR2A
rs2169052	chr1:161557153	FCGR2A
rs144605559	chr1:160925140	FCGR3B
rs56976800	chr1:161665374	FCGR3A
rs1340976	chr1:161661488	FCGR2A
rs532984690	chr1:161597122	FCGR2A
rs112104785	chr1:161851785	FCGR3A
rs112689091	chr1:161791601	FCGR2A
rs17417647	chr1:161928613	FCGR2A
rs72708088	chr1:161882188	FCGR2A
rs200837054	chr1:161577203	FCGR2A
rs142966355	chr1:161622469	FCGR2A
rs74651366	chr1:161649482	FCGR2A
rs112104785	chr1:161851785	FCGR3B
rs112626262	chr1:161782274	FCGR2A
rs56091027	chr1:161615394	FCGR2A
rs61802307	chr1:161577668	FCGR3B
rs61801161	chr1:161680517	FCGR2A
rs72714967	chr1:161291227	FCGR3A
rs12135523	chr1:161678253	FCGR2A
rs61804160	chr1:161620088	FCGR2A
rs35139848	chr1:161597176	FCGR2A
rs9427039	chr1:161390515	FCGR2A
rs112142198	chr1:161568861	FCGR2A
rs10917717	chr1:161672051	FCGR2A
rs61802334	chr1:161590276	FCGR2A
rs112296661	chr1:161863965	FCGR2A
rs61802328	chr1:161589326	FCGR2A
rs532090372	chr1:161600275	FCGR2A
rs3883934	chr1:161600831	FCGR2A
rs113293012	chr1:161919977	FCGR2A
rs72633696	chr1:161657895	FCGR2A
rs9427372	chr1:161399919	FCGR2A
rs75476984	chr1:161978244	FCGR2A
rs76731254	chr1:161876466	FCGR2B

rs112104785	chr1:161851785	FCGR2C
rs4657090	chr1:161662483	FCGR2A
rs111918621	chr1:161927277	FCGR2A
rs72714967	chr1:161291227	FCGR2C
rs4301614	chr1:161681847	FCGR2C
rs78861213	chr1:161603102	FCGR2A
rs79601960	chr1:161666121	FCGR2A
rs4301614	chr1:161681847	FCGR3A
rs12131017	chr1:161615369	FCGR2A
rs146140071	chr1:161649698	FCGR2A
rs74567013	chr1:161977236	FCGR3A
rs113505621	chr1:161798708	FCGR2A
rs61803028	chr1:161601294	FCGR2A
rs116205652	chr1:161659458	FCGR2A
rs56159502	chr1:161618544	FCGR2A
rs17416424	chr1:161783290	FCGR2A
rs79827863	chr1:161836559	FCGR2A
rs140391508	chr1:160913403	FCGR3B
rs72704072	chr1:161675889	FCGR2A
rs17417647	chr1:161928613	FCGR3A
rs112363639	chr1:161588852	FCGR2A
rs61804208	chr1:161661410	FCGR2A
rs57978522	chr1:161608159	FCGR2A
rs190884597	chr1:161758757	FCGR2A
rs35989200	chr1:161574304	FCGR2A
rs80300109	chr1:160977607	FCGR3A
rs61804209	chr1:161666632	FCGR2A
rs111796652	chr1:161831575	FCGR2A
rs74127054	chr1:161589245	FCGR2A
rs61348372	chr1:161617731	FCGR2A
rs61804207	chr1:161660717	FCGR2A
rs112699037	chr1:161941039	FCGR2A
rs1256288	chr1:161654842	FCGR2A
rs72702132	chr1:161597205	FCGR2A
rs72706028	chr1:161729439	FCGR2A
rs56077197	chr1:161731254	FCGR2A
rs74127079	chr1:161601419	FCGR2A
rs7525363	chr1:161660208	FCGR2A
rs4656327	chr1:161664582	FCGR2A
rs57924693	chr1:161977606	FCGR3A
rs112289794	chr1:161887647	FCGR2A
rs11578979	chr1:161658657	FCGR2A
rs551272312	chr1:161800986	FCGR2A
rs17450717	chr1:161899635	FCGR3A
rs61802307	chr1:161577668	FCGR2A
rs140578039	chr1:161358226	FCGR2A
rs17452514	chr1:161951076	FCGR2A
rs61801234	chr1:161716941	FCGR2A
rs112805729	chr1:161798658	FCGR2A
rs10737488	chr1:161619362	FCGR2A

rs4657086	chr1:161639284	FCGR2A
rs113946660	chr1:161913402	FCGR2A
rs61804205	chr1:161653736	FCGR2A
rs61809495	chr1:161936486	FCGR2A
rs149822268	chr1:161917828	FCGR2A
rs111402007	chr1:161600188	FCGR2A
rs76731254	chr1:161876466	FCGR2C
rs61801160	chr1:161673858	FCGR2A
rs17448571	chr1:161807951	FCGR2A
rs72706034	chr1:161732311	FCGR2A
rs111998161	chr1:161871791	FCGR2A
rs111992131	chr1:161929936	FCGR2A
rs142917083	chr1:161780700	FCGR2A
rs112674212	chr1:161913466	FCGR2A
rs17418028	chr1:161934951	FCGR2A
rs150392443	chr1:161598897	FCGR2A
rs76731254	chr1:161876466	FCGR3A
rs113910081	chr1:161865409	FCGR2A
rs80300109	chr1:160977607	FCGR2A
rs147936938	chr1:161668736	FCGR2A
rs115130696	chr1:161601259	FCGR2A
rs4656329	chr1:161667657	FCGR2A
rs72706035	chr1:161732627	FCGR2A
rs10753610	chr1:161663822	FCGR2A
rs17417198	chr1:161910746	FCGR2A
rs61809501	chr1:161941818	FCGR2A
rs61804210	chr1:161670826	FCGR2A
rs112811497	chr1:161802420	FCGR2A
rs113428915	chr1:161875881	FCGR2A
rs6683306	chr1:161660205	FCGR2A
rs144605559	chr1:160925140	FCGR3A
rs79016857	chr1:161835626	FCGR2A
rs17409485	chr1:161853119	FCGR2A
rs61801186	chr1:161703690	FCGR2A
rs150937010	chr1:161844725	FCGR2A
rs112859576	chr1:161900802	FCGR2A
rs72704075	chr1:161675993	FCGR2A
rs4656330	chr1:161668463	FCGR2A
rs191320140	chr1:160868347	FCGR3A
rs191320140	chr1:160868347	FCGR3B
rs112175965	chr1:161779939	FCGR2A
rs79515951	chr1:161825446	FCGR2A
rs61809496	chr1:161938877	FCGR2A
rs111787169	chr1:161900150	FCGR2A
rs17399583	chr1:161339575	FCGR2A
rs61801185	chr1:161703659	FCGR2A
rs115782472	chr1:161838205	FCGR2A
rs140809349	chr1:161849495	FCGR2A
rs111440656	chr1:161921760	FCGR2A
rs4628483	chr1:161958189	FCGR2A

rs6671332	chr1:161672450	FCGR2A
rs146797688	chr1:161664448	FCGR2A
rs183849087	chr1:161843083	FCGR2A
rs61804211	chr1:161671305	FCGR2A
rs56779916	chr1:161222047	FCGR2A
rs4657092	chr1:161665413	FCGR2A
rs144605559	chr1:160925140	FCGR2A
rs140391508	chr1:160913403	FCGR3A
rs113979070	chr1:161935328	FCGR2A
rs4301614	chr1:161681847	FCGR2A
rs184915469	chr1:161879915	FCGR2A
rs61801245	chr1:161732305	FCGR2A
rs77931302	chr1:161943946	FCGR2A
rs112401004	chr1:161912728	FCGR2A
rs74567013	chr1:161977236	FCGR2A
rs145143330	chr1:161778882	FCGR2A
rs61809498	chr1:161940670	FCGR2A
rs150138577	chr1:161850153	FCGR2A
rs114931935	chr1:161659800	FCGR2A
rs189314279	chr1:161948452	FCGR2A
rs56976800	chr1:161665374	FCGR2A
rs17418389	chr1:161941185	FCGR2A
rs61809500	chr1:161941091	FCGR2A
rs17450717	chr1:161899635	FCGR2A
rs57924693	chr1:161977606	FCGR2A
rs74328646	chr1:161851909	FCGR2A
rs191320140	chr1:160868347	FCGR2A
rs112104785	chr1:161851785	FCGR2A
rs74560530	chr1:161941387	FCGR2A
rs76731254	chr1:161876466	FCGR2A
rs140391508	chr1:160913403	FCGR2A

Gene Position	Distance	Tissue
chr1:161475219	19091	Whole Blood
chr1:161552879	64617	Whole Blood
chr1:161551100	66396	Whole Blood
chr1:161475219	38157	Whole Blood
chr1:161475219	20665	Whole Blood
chr1:161601752	15744	Whole Blood
chr1:161552879	16749	Whole Blood
chr1:161552879	37863	Whole Blood
chr1:161520526	7150	Whole Blood
chr1:161552879	37813	Whole Blood
chr1:161552879	37623	Whole Blood
chr1:161475219	115473	Whole Blood
chr1:161552879	39333	Whole Blood
chr1:161552879	38668	Whole Blood
chr1:161475219	60911	Whole Blood
chr1:161552879	39503	Whole Blood
chr1:161551100	40447	Whole Blood
chr1:161520526	26216	Whole Blood
chr1:161552879	22964	Whole Blood
chr1:161601752	10205	Whole Blood
chr1:161601752	11250	Whole Blood
chr1:161551100	39592	Whole Blood
chr1:161520526	15604	Whole Blood
chr1:161475219	4525	Whole Blood
chr1:161552879	56995	Whole Blood
chr1:161551100	39642	Whole Blood
chr1:161551100	55216	Whole Blood
chr1:161551100	14970	Whole Blood
chr1:161475219	8990	Whole Blood
chr1:161552879	58569	Whole Blood
chr1:161552879	39317	Whole Blood
chr1:161601752	403	Whole Blood
chr1:161552879	57988	Whole Blood
chr1:161551100	41096	Whole Blood
chr1:161552879	38100	Whole Blood
chr1:161475219	24910	Whole Blood
chr1:161551100	41112	Whole Blood
chr1:161551100	39552	Whole Blood
chr1:161551100	39879	Whole Blood
chr1:161601752	11010	Whole Blood
chr1:161552879	39346	Whole Blood
chr1:161552879	29375	Whole Blood
chr1:161601752	11060	Whole Blood
chr1:161601752	25909	Whole Blood
chr1:161552879	48470	Whole Blood
chr1:161601752	9115	Whole Blood
chr1:161551100	39402	Whole Blood
chr1:161475219	17583	Whole Blood
chr1:161601752	10773	Whole Blood

chr1:161601752	9540	Whole Blood
chr1:161551100	31154	Whole Blood
chr1:161551100	37724	Whole Blood
chr1:161520526	71686	Whole Blood
chr1:161552879	54092	Whole Blood
chr1:161475219	116993	Whole Blood
chr1:161520526	24642	Whole Blood
chr1:161520526	69976	Whole Blood
chr1:161475219	8904	Whole Blood
chr1:161552879	37773	Whole Blood
chr1:161475219	115523	Whole Blood
chr1:161475219	25675	Whole Blood
chr1:161552879	73135	Whole Blood
chr1:161601752	65622	Whole Blood
chr1:161601752	19498	Whole Blood
chr1:161552879	54511	Whole Blood
chr1:161551100	56790	Whole Blood
chr1:161601752	9556	Whole Blood
chr1:161601752	11100	Whole Blood
chr1:161551100	61565	Whole Blood
chr1:161520526	55317	Whole Blood
chr1:161520526	86864	Whole Blood
chr1:161475219	131752	Whole Blood
chr1:161601752	5638	Whole Blood
chr1:161601752	522	Whole Blood
chr1:161475219	116328	Whole Blood
chr1:161551100	56290	Whole Blood
chr1:161601752	9527	Whole Blood
chr1:161551100	55871	Whole Blood
chr1:161475219	115433	Whole Blood
chr1:161551100	24743	Whole Blood
chr1:161552879	16539	Whole Blood
chr1:161601752	10913	Whole Blood
chr1:161520526	70166	Whole Blood
chr1:161551100	59767	Whole Blood
chr1:161475219	135648	Whole Blood
chr1:161601752	88376	Whole Blood
chr1:161601752	5219	Whole Blood
chr1:161520526	71021	Whole Blood
chr1:161551100	41125	Whole Blood
chr1:161552879	59786	Whole Blood
chr1:161552879	95243	Whole Blood
chr1:161601752	11300	Whole Blood
chr1:161551100	93464	Whole Blood
chr1:161520526	61728	Whole Blood
chr1:161475219	32122	Whole Blood
chr1:161551100	50249	Whole Blood
chr1:161551100	63300	Whole Blood
chr1:161475219	115283	Whole Blood
chr1:161601752	107442	Whole Blood

chr1:161601752	214	Whole Blood
chr1:161520526	70216	Whole Blood
chr1:161551100	30409	Whole Blood
chr1:161551100	31399	Whole Blood
chr1:161551100	71356	Whole Blood
chr1:161601752	12737	Whole Blood
chr1:161520526	62890	Whole Blood
chr1:161475219	94199	Whole Blood
chr1:161551100	39352	Whole Blood
chr1:161520526	70453	Whole Blood
chr1:161520526	96970	Whole Blood
chr1:161601752	105868	Whole Blood
chr1:161520526	40782	Whole Blood
chr1:161475219	64762	Whole Blood
chr1:161475219	115760	Whole Blood
chr1:161552879	52750	Whole Blood
chr1:161475219	118351	Whole Blood
chr1:161475219	117739	Whole Blood
chr1:161552879	37573	Whole Blood
chr1:161475219	39956	Whole Blood
chr1:161552879	51985	Whole Blood
chr1:161475219	89248	Whole Blood
chr1:161552879	29620	Whole Blood
chr1:161552879	48351	Whole Blood
chr1:161475219	21680	Whole Blood
chr1:161552879	48659	Whole Blood
chr1:161520526	70126	Whole Blood
chr1:161551100	41858	Whole Blood
chr1:161551100	50206	Whole Blood
chr1:161520526	71670	Whole Blood
chr1:161475219	116977	Whole Blood
chr1:161552879	28630	Whole Blood
chr1:161475219	25576	Whole Blood
chr1:161551100	137638	Whole Blood
chr1:161475219	25492	Whole Blood
chr1:161552879	68756	Whole Blood
chr1:161551100	50130	Whole Blood
chr1:161520526	20397	Whole Blood
chr1:161552879	40691	Whole Blood
chr1:161601752	11404	Whole Blood
chr1:161552879	40079	Whole Blood
chr1:161551100	18318	Whole Blood
chr1:161520526	86445	Whole Blood
chr1:161551100	30165	Whole Blood
chr1:161552879	61521	Whole Blood
chr1:161551100	50438	Whole Blood
chr1:161552879	68670	Whole Blood
chr1:161475219	137446	Whole Blood
chr1:161601752	15588	Whole Blood
chr1:161520526	71699	Whole Blood

chr1:161475219	107280	Whole Blood
chr1:161520526	36403	Whole Blood
chr1:161552879	29522	Whole Blood
chr1:161601752	580	Whole Blood
chr1:161520526	92139	Whole Blood
chr1:161601752	8182	Whole Blood
chr1:161475219	2527	Whole Blood
chr1:161475219	115233	Whole Blood
chr1:161601752	19253	Whole Blood
chr1:161601752	12648	Whole Blood
chr1:161475219	142277	Whole Blood
chr1:161551100	50971	Whole Blood
chr1:161520526	19632	Whole Blood
chr1:161552879	61610	Whole Blood
chr1:161601752	144116	Whole Blood
chr1:161551100	31301	Whole Blood
chr1:161552879	11588	Whole Blood
chr1:161552879	67385	Whole Blood
chr1:161520526	90341	Whole Blood
chr1:161475219	106046	Whole Blood
chr1:161551100	31099	Whole Blood
chr1:161520526	69926	Whole Blood
chr1:161475219	224868	Whole Blood
chr1:161551100	42470	Whole Blood
chr1:161520526	72432	Whole Blood
chr1:161601752	86986	Whole Blood
chr1:161601752	17034	Whole Blood
chr1:161552879	142422	Whole Blood
chr1:161552879	76347	Whole Blood
chr1:161601752	122008	Whole Blood
chr1:161552879	28386	Whole Blood
chr1:161601752	32334	Whole Blood
chr1:161552879	37469	Whole Blood
chr1:161601752	37285	Whole Blood
chr1:161551100	40007	Whole Blood
chr1:161475219	38612	Whole Blood
chr1:161552879	135859	Whole Blood
chr1:161551100	74152	Whole Blood
chr1:161475219	126130	Whole Blood
chr1:161601752	20243	Whole Blood
chr1:161475219	18325	Whole Blood
chr1:161552879	48293	Whole Blood
chr1:161475219	107182	Whole Blood
chr1:161475219	213519	Whole Blood
chr1:161475219	1313	Whole Blood
chr1:161552879	28923	Whole Blood
chr1:161552879	64461	Whole Blood
chr1:161552879	37704	Whole Blood
chr1:161552879	48115	Whole Blood
chr1:161551100	148987	Whole Blood

chr1:161551100	69164	Whole Blood
chr1:161520526	5351	Whole Blood
chr1:161551100	39248	Whole Blood
chr1:161551100	140643	Whole Blood
chr1:161475219	984	Whole Blood
chr1:161552879	147208	Whole Blood
chr1:161552879	38228	Whole Blood
chr1:161551100	13367	Whole Blood
chr1:161551100	63389	Whole Blood
chr1:161552879	52168	Whole Blood
chr1:161520526	77429	Whole Blood
chr1:161475219	100624	Whole Blood
chr1:161551100	40223	Whole Blood
chr1:161601752	18512	Whole Blood
chr1:161552879	29320	Whole Blood
chr1:161601752	17887	Whole Blood
chr1:161601752	15613	Whole Blood
chr1:161601752	107395	Whole Blood
chr1:161552879	38444	Whole Blood
chr1:161475219	32134	Whole Blood
chr1:161551100	74568	Whole Blood
chr1:161601752	8794	Whole Blood
chr1:161552879	109782	Whole Blood
chr1:161552879	110020	Whole Blood
chr1:161551100	66891	Whole Blood
chr1:161551100	30702	Whole Blood
chr1:161551100	68539	Whole Blood
chr1:161520526	48892	Whole Blood
chr1:161601752	10429	Whole Blood
chr1:161551100	41296	Whole Blood
chr1:161475219	40652	Whole Blood
chr1:161475219	18085	Whole Blood
chr1:161551100	66240	Whole Blood
chr1:161552879	76676	Whole Blood
chr1:161552879	182501	Whole Blood
chr1:161520526	73044	Whole Blood
chr1:161475219	233928	Whole Blood
chr1:161601752	100858	Whole Blood
chr1:161552879	150555	Whole Blood
chr1:161475219	117006	Whole Blood
chr1:161475219	132171	Whole Blood
chr1:161601752	19351	Whole Blood
chr1:161551100	152334	Whole Blood
chr1:161520526	36317	Whole Blood
chr1:161551100	158047	Whole Blood
chr1:161552879	46774	Whole Blood
chr1:161551100	74897	Whole Blood
chr1:161601752	20487	Whole Blood
chr1:161552879	33761	Whole Blood
chr1:161552879	59575	Whole Blood

chr1:161520526	61973	Whole Blood
chr1:161601752	19553	Whole Blood
chr1:161520526	168212	Whole Blood
chr1:161520526	93874	Whole Blood
chr1:161552879	39099	Whole Blood
chr1:161475219	39082	Whole Blood
chr1:161475219	32360	Whole Blood
chr1:161551100	66977	Whole Blood
chr1:161551100	30408	Whole Blood
chr1:161601752	101623	Whole Blood
chr1:161552879	38578	Whole Blood
chr1:161601752	758	Whole Blood
chr1:161551100	353380	Whole Blood
chr1:161551100	50305	Whole Blood
chr1:161601752	117629	Whole Blood
chr1:161552879	66760	Whole Blood
chr1:161551100	50389	Whole Blood
chr1:161552879	52084	Whole Blood
chr1:161552879	110445	Whole Blood
chr1:161552879	76675	Whole Blood
chr1:161552879	183172	Whole Blood
chr1:161475219	43899	Whole Blood
chr1:161551100	50072	Whole Blood
chr1:161551100	74896	Whole Blood
chr1:161520526	215525	Whole Blood
chr1:161475219	106290	Whole Blood
chr1:161601752	10645	Whole Blood
chr1:161475219	107035	Whole Blood
chr1:161475219	44191	Whole Blood
chr1:161552879	49867	Whole Blood
chr1:161520526	42780	Whole Blood
chr1:161551100	189055	Whole Blood
chr1:161552879	54091	Whole Blood
chr1:161551100	354334	Whole Blood
chr1:161551100	40878	Whole Blood
chr1:161551100	170166	Whole Blood
chr1:161552879	75931	Whole Blood
chr1:161520526	61875	Whole Blood
chr1:161551100	184280	Whole Blood
chr1:161552879	110019	Whole Blood
chr1:161475219	106980	Whole Blood
chr1:161552879	28279	Whole Blood
chr1:161551100	30413	Whole Blood
chr1:161552879	54046	Whole Blood
chr1:161475219	115888	Whole Blood
chr1:161552879	28629	Whole Blood
chr1:161552879	39517	Whole Blood
chr1:161552879	93745	Whole Blood
chr1:161520526	43941	Whole Blood
chr1:161551100	195943	Whole Blood

chr1:161551100	67686	Whole Blood
chr1:161520526	60983	Whole Blood
chr1:161520526	26982	Whole Blood
chr1:161520526	19815	Whole Blood
chr1:161601752	9356	Whole Blood
chr1:161601752	101041	Whole Blood
chr1:161520526	60739	Whole Blood
chr1:161552879	55980	Whole Blood
chr1:161551100	108003	Whole Blood
chr1:161475219	106583	Whole Blood
chr1:161601752	106747	Whole Blood
chr1:161520526	110069	Whole Blood
chr1:161475219	45711	Whole Blood
chr1:161475219	530	Whole Blood
chr1:161551100	30058	Whole Blood
chr1:161601752	101682	Whole Blood
chr1:161552879	156268	Whole Blood
chr1:161551100	31351	Whole Blood
chr1:161520526	43578	Whole Blood
chr1:161520526	80704	Whole Blood
chr1:161520526	179561	Whole Blood
chr1:161601752	191295	Whole Blood
chr1:161552879	183329	Whole Blood
chr1:161552879	75133	Whole Blood
chr1:161601752	5218	Whole Blood
chr1:161520526	60632	Whole Blood
chr1:161520526	44323	Whole Blood
chr1:161552879	29572	Whole Blood
chr1:161475219	131706	Whole Blood
chr1:161551100	95524	Whole Blood
chr1:161552879	65907	Whole Blood
chr1:161552879	59335	Whole Blood
chr1:161520526	19731	Whole Blood
chr1:161601752	20594	Whole Blood
chr1:161601752	133628	Whole Blood
chr1:161475219	116104	Whole Blood
chr1:161552879	167469	Whole Blood
chr1:161552879	71137	Whole Blood
chr1:161552879	37554	Whole Blood
chr1:161475219	985	Whole Blood
chr1:161551100	74035	Whole Blood
chr1:161520526	80823	Whole Blood
chr1:161552879	31949	Whole Blood
chr1:161520526	199822	Whole Blood
chr1:161520526	6225	Whole Blood
chr1:161601752	98335	Whole Blood
chr1:161552879	39048	Whole Blood
chr1:161475219	1729	Whole Blood
chr1:161601752	19950	Whole Blood
chr1:161551100	35925	Whole Blood

chr1:161552879	155620	Whole Blood
chr1:161475219	32359	Whole Blood
chr1:161601752	19301	Whole Blood
chr1:161551100	57556	Whole Blood
chr1:161520526	44652	Whole Blood
chr1:161551100	108241	Whole Blood
chr1:161520526	1116	Whole Blood
chr1:161520526	38784	Whole Blood
chr1:161552879	64486	Whole Blood
chr1:161601752	20244	Whole Blood
chr1:161601752	86427	Whole Blood
chr1:161520526	214854	Whole Blood
chr1:161552879	109794	Whole Blood
chr1:161520526	23627	Whole Blood
chr1:161601752	37241	Whole Blood
chr1:161552879	11632	Whole Blood
chr1:161475219	5429	Whole Blood
chr1:161551100	73354	Whole Blood
chr1:161551100	48553	Whole Blood
chr1:161520526	70797	Whole Blood
chr1:161475219	105939	Whole Blood
chr1:161520526	6695	Whole Blood
chr1:161601752	2099	Whole Blood
chr1:161552879	75814	Whole Blood
chr1:161601752	9774	Whole Blood
chr1:161520526	187973	Whole Blood
chr1:161552879	77005	Whole Blood
chr1:161475219	23630	Whole Blood
chr1:161475219	131751	Whole Blood
chr1:161520526	81012	Whole Blood
chr1:161551100	75226	Whole Blood
chr1:161601752	86577	Whole Blood
chr1:161520526	70581	Whole Blood
chr1:161475219	117177	Whole Blood
chr1:161551100	36799	Whole Blood
chr1:161552879	144511	Whole Blood
chr1:161475219	40106	Whole Blood
chr1:161520526	215682	Whole Blood
chr1:161475219	655	Whole Blood
chr1:161520526	43994	Whole Blood
chr1:161551100	75351	Whole Blood
chr1:161551100	55825	Whole Blood
chr1:161601752	134456	Whole Blood
chr1:161520526	69822	Whole Blood
chr1:161552879	77130	Whole Blood
chr1:161601752	134299	Whole Blood
chr1:161551100	55870	Whole Blood
chr1:161552879	19825	Whole Blood
chr1:161552879	54093	Whole Blood
chr1:161520526	43985	Whole Blood

chr1:161475219	32785	Whole Blood
chr1:161551100	31378	Whole Blood
chr1:161551100	49894	Whole Blood
chr1:161552879	29798	Whole Blood
chr1:161551100	40441	Whole Blood
chr1:161520526	61276	Whole Blood
chr1:161520526	44777	Whole Blood
chr1:161552879	38662	Whole Blood
chr1:161551100	137663	Whole Blood
chr1:161520526	44322	Whole Blood
chr1:161552879	29599	Whole Blood
chr1:161601752	125549	Whole Blood
chr1:161601752	44872	Whole Blood
chr1:161601752	108448	Whole Blood
chr1:161601752	19075	Whole Blood
chr1:161520526	61673	Whole Blood
chr1:161601752	120896	Whole Blood
chr1:161601752	98740	Whole Blood
chr1:161551100	35775	Whole Blood
chr1:161475219	89292	Whole Blood
chr1:161475219	116759	Whole Blood
chr1:161475219	65098	Whole Blood
chr1:161551100	54201	Whole Blood
chr1:161551100	108015	Whole Blood
chr1:161601752	125548	Whole Blood
chr1:161551100	157399	Whole Blood
chr1:161551100	108240	Whole Blood
chr1:161551100	108666	Whole Blood
chr1:161475219	107232	Whole Blood
chr1:161552879	168387	Whole Blood
chr1:161520526	43461	Whole Blood
chr1:161475219	107259	Whole Blood
chr1:161551100	184951	Whole Blood
chr1:161475219	142121	Whole Blood
chr1:161475219	1846	Whole Blood
chr1:161601752	20239	Whole Blood
chr1:161552879	28634	Whole Blood
chr1:161520526	38007	Whole Blood
chr1:161520526	1408	Whole Blood
chr1:161551100	31577	Whole Blood
chr1:161520526	60982	Whole Blood
chr1:161551100	352551	Whole Blood
chr1:161601752	100957	Whole Blood
chr1:161601752	117543	Whole Blood
chr1:161551100	66265	Whole Blood
chr1:161552879	13078	Whole Blood
chr1:161551100	39966	Whole Blood
chr1:161601752	302728	Whole Blood
chr1:161475219	56562	Whole Blood
chr1:161520526	17514	Whole Blood

chr1:161520526	98260	Whole Blood
chr1:161520526	39878	Whole Blood
chr1:161551100	371264	Whole Blood
chr1:161601752	158655	Whole Blood
chr1:161601752	82342	Whole Blood
chr1:161601752	80822	Whole Blood
chr1:161552879	156331	Whole Blood
chr1:161520526	77667	Whole Blood
chr1:161551100	185108	Whole Blood
chr1:161551100	171548	Whole Blood
chr1:161551100	66415	Whole Blood
chr1:161475219	107458	Whole Blood
chr1:161552879	64636	Whole Blood
chr1:161552879	30227	Whole Blood
chr1:161601752	124006	Whole Blood
chr1:161601752	19274	Whole Blood
chr1:161520526	5106	Whole Blood
chr1:161601752	10211	Whole Blood
chr1:161551100	173026	Whole Blood
chr1:161551100	158110	Whole Blood
chr1:161552879	21540	Whole Blood
chr1:161551100	169248	Whole Blood
chr1:161552879	33469	Whole Blood
chr1:161601752	125220	Whole Blood
chr1:161551100	337168	Whole Blood
chr1:161551100	173148	Whole Blood
chr1:161520526	404	Whole Blood
chr1:161551100	30170	Whole Blood
chr1:161552879	37008	Whole Blood
chr1:161551100	31690	Whole Blood
chr1:161552879	90152	Whole Blood
chr1:161475219	115129	Whole Blood
chr1:161601752	5173	Whole Blood
chr1:161601752	119514	Whole Blood
chr1:161552879	39598	Whole Blood
chr1:161520526	27222	Whole Blood
chr1:161551100	187537	Whole Blood
chr1:161475219	64571	Whole Blood
chr1:161520526	71452	Whole Blood
chr1:161475219	65042	Whole Blood
chr1:161520526	383954	Whole Blood
chr1:161601752	107458	Whole Blood
chr1:161601752	121104	Whole Blood
chr1:161475219	116322	Whole Blood
chr1:161520526	41089	Whole Blood
chr1:161551100	190810	Whole Blood
chr1:161551100	48088	Whole Blood
chr1:161601752	95794	Whole Blood
chr1:161552879	70360	Whole Blood
chr1:161601752	15763	Whole Blood

chr1:161475219	27793	Whole Blood
chr1:161552879	37523	Whole Blood
chr1:161601752	82634	Whole Blood
chr1:161601752	10686	Whole Blood
chr1:161552879	36520	Whole Blood
chr1:161551100	354323	Whole Blood
chr1:161601752	122496	Whole Blood
chr1:161552879	181845	Whole Blood
chr1:161601752	118596	Whole Blood
chr1:161601752	11350	Whole Blood
chr1:161601752	5220	Whole Blood
chr1:161552879	38187	Whole Blood
chr1:161552879	21748	Whole Blood
chr1:161520526	93963	Whole Blood
chr1:161552879	144667	Whole Blood
chr1:161520526	11255	Whole Blood
chr1:161551100	57796	Whole Blood
chr1:161551100	37269	Whole Blood
chr1:161552879	142702	Whole Blood
chr1:161601752	136885	Whole Blood
chr1:161551100	73897	Whole Blood
chr1:161601752	9275	Whole Blood
chr1:161475219	266691	Whole Blood
chr1:161520526	57799	Whole Blood
chr1:161520526	43323	Whole Blood
chr1:161520526	79127	Whole Blood
chr1:161552879	27247	Whole Blood
chr1:161520526	110349	Whole Blood
chr1:161551100	32006	Whole Blood
chr1:161552879	189031	Whole Blood
chr1:161601752	140158	Whole Blood
chr1:161552879	74172	Whole Blood
chr1:161551100	69358	Whole Blood
chr1:161552879	21098	Whole Blood
chr1:161552879	230882	Whole Blood
chr1:161520526	77666	Whole Blood
chr1:161475219	429261	Whole Blood
chr1:161601752	87451	Whole Blood
chr1:161475219	56120	Whole Blood
chr1:161601752	70413	Whole Blood
chr1:161551100	40585	Whole Blood
chr1:161520526	10813	Whole Blood
chr1:161601752	87921	Whole Blood
chr1:161552879	187276	Whole Blood
chr1:161475219	41140	Whole Blood
chr1:161520526	71870	Whole Blood
chr1:161551100	173202	Whole Blood
chr1:161475219	57835	Whole Blood
chr1:161601752	158893	Whole Blood
chr1:161551100	149547	Whole Blood

chr1:161601752	7791	Whole Blood
chr1:161520526	60987	Whole Blood
chr1:161552879	54030	Whole Blood
chr1:161520526	62580	Whole Blood
chr1:161552879	194164	Whole Blood
chr1:161475219	106294	Whole Blood
chr1:161601752	125878	Whole Blood
chr1:161551100	13411	Whole Blood
chr1:161552879	185758	Whole Blood
chr1:161475219	50413	Whole Blood
chr1:161551100	229103	Whole Blood
chr1:161552879	75676	Whole Blood
chr1:161552879	351601	Whole Blood
chr1:161551100	126634	Whole Blood
chr1:161551100	183624	Whole Blood
chr1:161520526	61952	Whole Blood
chr1:161551100	140923	Whole Blood
chr1:161520526	80468	Whole Blood
chr1:161551100	133789	Whole Blood
chr1:161601752	18646	Whole Blood
chr1:161552879	109508	Whole Blood
chr1:161601752	8035	Whole Blood
chr1:161552879	352555	Whole Blood
chr1:161552879	124855	Whole Blood
chr1:161520526	86444	Whole Blood
chr1:161551100	148145	Whole Blood
chr1:161520526	62151	Whole Blood
chr1:161552879	110545	Whole Blood
chr1:161601752	134519	Whole Blood
chr1:161601752	85393	Whole Blood
chr1:161552879	172912	Whole Blood
chr1:161551100	39302	Whole Blood
chr1:161552879	181424	Whole Blood
chr1:161475219	139181	Whole Blood
chr1:161520526	99738	Whole Blood
chr1:161601752	5624	Whole Blood
chr1:161552879	142758	Whole Blood
chr1:161475219	259505	Whole Blood
chr1:161552879	171369	Whole Blood
chr1:161601752	76120	Whole Blood
chr1:161551100	88373	Whole Blood
chr1:161551100	373014	Whole Blood
chr1:161475219	115847	Whole Blood
chr1:161601752	104853	Whole Blood
chr1:161552879	50948	Whole Blood
chr1:161601752	132972	Whole Blood
chr1:161475219	106289	Whole Blood
chr1:161552879	41082	Whole Blood
chr1:161520526	71015	Whole Blood
chr1:161551100	107729	Whole Blood

chr1:161475219	31848	Whole Blood
chr1:161601752	5049	Whole Blood
chr1:161601752	191631	Whole Blood
chr1:161552879	53009	Whole Blood
chr1:161551100	55872	Whole Blood
chr1:161520526	61925	Whole Blood
chr1:161601752	122374	Whole Blood
chr1:161520526	5201	Whole Blood
chr1:161520526	96814	Whole Blood
chr1:161475219	35296	Whole Blood
chr1:161520526	86399	Whole Blood
chr1:161601752	158667	Whole Blood
chr1:161601752	108208	Whole Blood
chr1:161601752	83137	Whole Blood
chr1:161552879	42364	Whole Blood
chr1:161520526	99113	Whole Blood
chr1:161520526	188621	Whole Blood
chr1:161551100	72393	Whole Blood
chr1:161551100	42861	Whole Blood
chr1:161520526	42711	Whole Blood
chr1:161475219	12492	Whole Blood
chr1:161552879	135884	Whole Blood
chr1:161551100	41377	Whole Blood
chr1:161551100	49169	Whole Blood
chr1:161601752	145291	Whole Blood
chr1:161520526	78092	Whole Blood
chr1:161520526	21677	Whole Blood
chr1:161475219	50967	Whole Blood
chr1:161552879	54401	Whole Blood
chr1:161552879	54497	Whole Blood
chr1:161475219	32179	Whole Blood
chr1:161551100	140979	Whole Blood
chr1:161475219	6523	Whole Blood
chr1:161552879	132010	Whole Blood
chr1:161551100	42857	Whole Blood
chr1:161520526	126098	Whole Blood
chr1:161520526	214198	Whole Blood
chr1:161551100	23527	Whole Blood
chr1:161601752	132551	Whole Blood
chr1:161551100	31982	Whole Blood
chr1:161601752	85881	Whole Blood
chr1:161601752	69971	Whole Blood
chr1:161475219	4132	Whole Blood
chr1:161520526	70540	Whole Blood
chr1:161551100	18046	Whole Blood
chr1:161551100	142732	Whole Blood
chr1:161475219	131753	Whole Blood
chr1:161601752	87011	Whole Blood
chr1:161552879	142231	Whole Blood
chr1:161520526	77441	Whole Blood

chr1:161551100	35229	Whole Blood
chr1:161601752	124804	Whole Blood
chr1:161520526	110405	Whole Blood
chr1:161552879	35200	Whole Blood
chr1:161475219	107887	Whole Blood
chr1:161520526	41819	Whole Blood
chr1:161601752	5393	Whole Blood
chr1:161552879	22658	Whole Blood
chr1:161551100	146446	Whole Blood
chr1:161552879	109839	Whole Blood
chr1:161601752	139025	Whole Blood
chr1:161551100	108766	Whole Blood
chr1:161601752	26215	Whole Blood
chr1:161551100	339230	Whole Blood
chr1:161601752	303682	Whole Blood
chr1:161601752	98895	Whole Blood
chr1:161601752	5528	Whole Blood
chr1:161552879	40838	Whole Blood
chr1:161552879	72231	Whole Blood
chr1:161552879	75064	Whole Blood
chr1:161601752	1009	Whole Blood
chr1:161552879	11468	Whole Blood
chr1:161475219	233991	Whole Blood
chr1:161551100	19761	Whole Blood
chr1:161551100	183203	Whole Blood
chr1:161475219	3488	Whole Blood
chr1:161601752	27125	Whole Blood
chr1:161601752	91237	Whole Blood
chr1:161552879	17923	Whole Blood
chr1:161601752	7795	Whole Blood
chr1:161601752	75566	Whole Blood
chr1:161551100	52251	Whole Blood
chr1:161601752	75356	Whole Blood
chr1:161475219	115183	Whole Blood
chr1:161520526	168237	Whole Blood
chr1:161520526	10011	Whole Blood
chr1:161601752	124549	Whole Blood
chr1:161601752	158892	Whole Blood
chr1:161551100	31448	Whole Blood
chr1:161475219	213544	Whole Blood
chr1:161552879	353449	Whole Blood
chr1:161551100	56276	Whole Blood
chr1:161552879	74711	Whole Blood
chr1:161601752	68698	Whole Blood
chr1:161520526	85362	Whole Blood
chr1:161552879	183392	Whole Blood
chr1:161551100	185171	Whole Blood
chr1:161552879	61984	Whole Blood
chr1:161552879	39795	Whole Blood
chr1:161601752	75982	Whole Blood

chr1:161551100	24437	Whole Blood
chr1:161601752	126003	Whole Blood
chr1:161551100	127321	Whole Blood
chr1:161520526	96839	Whole Blood
chr1:161601752	138403	Whole Blood
chr1:161520526	73191	Whole Blood
chr1:161601752	9078	Whole Blood
chr1:161551100	108060	Whole Blood
chr1:161475219	26712	Whole Blood
chr1:161601752	102903	Whole Blood
chr1:161552879	18423	Whole Blood
chr1:161475219	1984	Whole Blood
chr1:161520526	244584	Whole Blood
chr1:161552879	29669	Whole Blood
chr1:161475219	142296	Whole Blood
chr1:161520526	5660	Whole Blood
chr1:161551100	41574	Whole Blood
chr1:161601752	191575	Whole Blood
chr1:161552879	22773	Whole Blood
chr1:161552879	146366	Whole Blood
chr1:161552879	60213	Whole Blood
chr1:161520526	213777	Whole Blood
chr1:161475219	118738	Whole Blood
chr1:161520526	78192	Whole Blood
chr1:161551100	56180	Whole Blood
chr1:161552879	26483	Whole Blood
chr1:161601752	159318	Whole Blood
chr1:161552879	125542	Whole Blood
chr1:161520526	86850	Whole Blood
chr1:161520526	41737	Whole Blood
chr1:161520526	96989	Whole Blood
chr1:161601752	76669	Whole Blood
chr1:161520526	80646	Whole Blood
chr1:161552879	62666	Whole Blood
chr1:161520526	177020	Whole Blood
chr1:161552879	21866	Whole Blood
chr1:161551100	351670	Whole Blood
chr1:161552879	10674	Whole Blood
chr1:161601752	4756	Whole Blood
chr1:161520526	4655	Whole Blood
chr1:161520526	215745	Whole Blood
chr1:161475219	126011	Whole Blood
chr1:161552879	47864	Whole Blood
chr1:161520526	72148	Whole Blood
chr1:161551100	70452	Whole Blood
chr1:161520526	188684	Whole Blood
chr1:161601752	4136	Whole Blood
chr1:161551100	25468	Whole Blood
chr1:161551100	71749	Whole Blood
chr1:161552879	16268	Whole Blood

chr1:161520526	18595	Whole Blood
chr1:161520526	69876	Whole Blood
chr1:161551100	240239	Whole Blood
chr1:161475219	117455	Whole Blood
chr1:161552879	35254	Whole Blood
chr1:161552879	41078	Whole Blood
chr1:161552879	238767	Whole Blood
chr1:161520526	182908	Whole Blood
chr1:161475219	143567	Whole Blood
chr1:161551100	42617	Whole Blood
chr1:161520526	221384	Whole Blood
chr1:161601752	120010	Whole Blood
chr1:161552879	144483	Whole Blood
chr1:161520526	62022	Whole Blood
chr1:161520526	384908	Whole Blood
chr1:161552879	34881	Whole Blood
chr1:161520526	4167	Whole Blood
chr1:161551100	24660	Whole Blood
chr1:161552879	100987	Whole Blood
chr1:161551100	45603	Whole Blood
chr1:161520526	45431	Whole Blood
chr1:161552879	173139	Whole Blood
chr1:161475219	145045	Whole Blood
chr1:161520526	109878	Whole Blood
chr1:161551100	140452	Whole Blood
chr1:161552879	147768	Whole Blood
chr1:161475219	124434	Whole Blood
chr1:161520526	86754	Whole Blood
chr1:161520526	198529	Whole Blood
chr1:161601752	158712	Whole Blood
chr1:161601752	89995	Whole Blood
chr1:161551100	72932	Whole Blood
chr1:161552879	43824	Whole Blood
chr1:161551100	142704	Whole Blood
chr1:161520526	77155	Whole Blood
chr1:161601752	71646	Whole Blood
chr1:161601752	123937	Whole Blood
chr1:161520526	9580	Whole Blood
chr1:161520526	12528	Whole Blood
chr1:161552879	246415	Whole Blood
chr1:161601752	124039	Whole Blood
chr1:161552879	35195	Whole Blood
chr1:161552879	26693	Whole Blood
chr1:161520526	76177	Whole Blood
chr1:161475219	199277	Whole Blood
chr1:161551100	23645	Whole Blood
chr1:161551100	73285	Whole Blood
chr1:161552879	74090	Whole Blood
chr1:161552879	22881	Whole Blood
chr1:161601752	27007	Whole Blood

chr1:161520526	71951	Whole Blood
chr1:161475219	17447	Whole Blood
chr1:161475219	32885	Whole Blood
chr1:161551100	225842	Whole Blood
chr1:161601752	98896	Whole Blood
chr1:161520526	41175	Whole Blood
chr1:161601752	5765	Whole Blood
chr1:161601752	123584	Whole Blood
chr1:161551100	54788	Whole Blood
chr1:161520526	73431	Whole Blood
chr1:161551100	68581	Whole Blood
chr1:161551100	56018	Whole Blood
chr1:161520526	200740	Whole Blood
chr1:161601752	8631	Whole Blood
chr1:161520526	86446	Whole Blood
chr1:161552879	69955	Whole Blood
chr1:161551100	149548	Whole Blood
chr1:161475219	139270	Whole Blood
chr1:161520526	42358	Whole Blood
chr1:161552879	169769	Whole Blood
chr1:161601752	122315	Whole Blood
chr1:161552879	276937	Whole Blood
chr1:161551100	128911	Whole Blood
chr1:161520526	218111	Whole Blood
chr1:161601752	158381	Whole Blood
chr1:161552879	44117	Whole Blood
chr1:161475219	7300	Whole Blood
chr1:161552879	44244	Whole Blood
chr1:161601752	124266	Whole Blood
chr1:161552879	54239	Whole Blood
chr1:161601752	5366	Whole Blood
chr1:161601752	97493	Whole Blood
chr1:161552879	77919	Whole Blood
chr1:161551100	56045	Whole Blood
chr1:161601752	13793	Whole Blood
chr1:161520526	45566	Whole Blood
chr1:161552879	91507	Whole Blood
chr1:161551100	140647	Whole Blood
chr1:161475219	51177	Whole Blood
chr1:161520526	5870	Whole Blood
chr1:161475219	259084	Whole Blood
chr1:161520526	73435	Whole Blood
chr1:161601752	301899	Whole Blood
chr1:161475219	4218	Whole Blood
chr1:161552879	54266	Whole Blood
chr1:161552879	47229	Whole Blood
chr1:161552879	218932	Whole Blood
chr1:161552879	10816	Whole Blood
chr1:161475219	233280	Whole Blood
chr1:161552879	171247	Whole Blood

chr1:161551100	249335	Whole Blood
chr1:161475219	125953	Whole Blood
chr1:161601752	120855	Whole Blood
chr1:161475219	15676	Whole Blood
chr1:161552879	63937	Whole Blood
chr1:161601752	122401	Whole Blood
chr1:161552879	48238	Whole Blood
chr1:161601752	35795	Whole Blood
chr1:161601752	13673	Whole Blood
chr1:161551100	36979	Whole Blood
chr1:161552879	26807	Whole Blood
chr1:161552879	102539	Whole Blood
chr1:161475219	132061	Whole Blood
chr1:161551100	174918	Whole Blood
chr1:161552879	35433	Whole Blood
chr1:161552879	44117	Whole Blood
chr1:161601752	5602	Whole Blood
chr1:161552879	73528	Whole Blood
chr1:161551100	45896	Whole Blood
chr1:161552879	24816	Whole Blood
chr1:161552879	17227	Whole Blood
chr1:161520526	86619	Whole Blood
chr1:161475219	121484	Whole Blood
chr1:161601752	124687	Whole Blood
chr1:161552879	44263	Whole Blood
chr1:161601752	191104	Whole Blood
chr1:161601752	13619	Whole Blood
chr1:161475219	275789	Whole Blood
chr1:161475219	263418	Whole Blood
chr1:161601752	16962	Whole Blood
chr1:161552879	17323	Whole Blood
chr1:161552879	14309	Whole Blood
chr1:161475219	264936	Whole Blood
chr1:161552879	54638	Whole Blood
chr1:161551100	12453	Whole Blood
chr1:161520526	43027	Whole Blood
chr1:161520526	180121	Whole Blood
chr1:161552879	237602	Whole Blood
chr1:161552879	18869	Whole Blood
chr1:161551100	14857	Whole Blood
chr1:161601752	26604	Whole Blood
chr1:161475219	260161	Whole Blood
chr1:161552879	85043	Whole Blood
chr1:161601752	42634	Whole Blood
chr1:161551100	72311	Whole Blood
chr1:161520526	76470	Whole Blood
chr1:161475219	130669	Whole Blood
chr1:161475219	2949	Whole Blood
chr1:161601752	159418	Whole Blood
chr1:161552879	90763	Whole Blood

chr1:161520526	77486	Whole Blood
chr1:161475219	131926	Whole Blood
chr1:161475219	144420	Whole Blood
chr1:161520526	54101	Whole Blood
chr1:161601752	13440	Whole Blood
chr1:161601752	25992	Whole Blood
chr1:161475219	118498	Whole Blood
chr1:161601752	320612	Whole Blood
chr1:161551100	174691	Whole Blood
chr1:161552879	17783	Whole Blood
chr1:161601752	21004	Whole Blood
chr1:161601752	37405	Whole Blood
chr1:161551100	129690	Whole Blood
chr1:161552879	352544	Whole Blood
chr1:161475219	107329	Whole Blood
chr1:161475219	271824	Whole Blood
chr1:161552879	169728	Whole Blood
chr1:161552879	48641	Whole Blood
chr1:161601752	19204	Whole Blood
chr1:161601752	1644	Whole Blood
chr1:161552879	69877	Whole Blood
chr1:161552879	18263	Whole Blood
chr1:161552879	20415	Whole Blood
chr1:161552879	335389	Whole Blood
chr1:161551100	19702	Whole Blood
chr1:161552879	19062	Whole Blood
chr1:161601752	122963	Whole Blood
chr1:161601752	189983	Whole Blood
chr1:161551100	92542	Whole Blood
chr1:161551100	34741	Whole Blood
chr1:161552879	214977	Whole Blood
chr1:161551100	64445	Whole Blood
chr1:161552879	153747	Whole Blood
chr1:161552879	65835	Whole Blood
chr1:161601752	119233	Whole Blood
chr1:161551100	19562	Whole Blood
chr1:161520526	383125	Whole Blood
chr1:161552879	101762	Whole Blood
chr1:161475219	132157	Whole Blood
chr1:161601752	38057	Whole Blood
chr1:161551100	235823	Whole Blood
chr1:161520526	55011	Whole Blood
chr1:161552879	40242	Whole Blood
chr1:161551100	20202	Whole Blood
chr1:161551100	220711	Whole Blood
chr1:161520526	55234	Whole Blood
chr1:161551100	357776	Whole Blood
chr1:161601752	13656	Whole Blood
chr1:161551100	171704	Whole Blood
chr1:161551100	71734	Whole Blood

chr1:161601752	99821	Whole Blood
chr1:161552879	369485	Whole Blood
chr1:161551100	219948	Whole Blood
chr1:161552879	19880	Whole Blood
chr1:161601752	286516	Whole Blood
chr1:161552879	146824	Whole Blood
chr1:161475219	112860	Whole Blood
chr1:161601752	4629	Whole Blood
chr1:161552879	55433	Whole Blood
chr1:161552879	47769	Whole Blood
chr1:161475219	245129	Whole Blood
chr1:161551100	46023	Whole Blood
chr1:161552879	125302	Whole Blood
chr1:161551100	24914	Whole Blood
chr1:161552879	21860	Whole Blood
chr1:161551100	50017	Whole Blood
chr1:161551100	18047	Whole Blood
chr1:161601752	122550	Whole Blood
chr1:161601752	41890	Whole Blood
chr1:161551100	19319	Whole Blood
chr1:161601752	635	Whole Blood
chr1:161601752	32605	Whole Blood
chr1:161552879	102283	Whole Blood
chr1:161552879	22269	Whole Blood
chr1:161552879	90192	Whole Blood
chr1:161551100	49643	Whole Blood
chr1:161552879	127132	Whole Blood
chr1:161475219	121777	Whole Blood
chr1:161552879	147769	Whole Blood
chr1:161601752	21488	Whole Blood
chr1:161601752	21082	Whole Blood
chr1:161551100	13247	Whole Blood
chr1:161551100	216756	Whole Blood
chr1:161551100	67614	Whole Blood
chr1:161552879	108173	Whole Blood
chr1:161520526	180122	Whole Blood
chr1:161475219	153222	Whole Blood
chr1:161552879	35217	Whole Blood
chr1:161551100	261660	Whole Blood
chr1:161520526	178719	Whole Blood
chr1:161551100	371427	Whole Blood
chr1:161475219	2596	Whole Blood
chr1:161601752	306343	Whole Blood
chr1:161552879	54475	Whole Blood
chr1:161601752	305696	Whole Blood
chr1:161520526	384897	Whole Blood
chr1:161551100	129331	Whole Blood
chr1:161601752	189587	Whole Blood
chr1:161551100	363989	Whole Blood
chr1:161552879	73442	Whole Blood

chr1:161552879	59460	Whole Blood
chr1:161475219	117258	Whole Blood
chr1:161551100	200764	Whole Blood
chr1:161551100	171507	Whole Blood
chr1:161551100	236988	Whole Blood
chr1:161552879	46918	Whole Blood
chr1:161601752	38199	Whole Blood
chr1:161552879	45432	Whole Blood
chr1:161551100	56254	Whole Blood
chr1:161475219	259	Whole Blood
chr1:161475219	126319	Whole Blood
chr1:161475219	33543	Whole Blood
chr1:161551100	16088	Whole Blood
chr1:161552879	101241	Whole Blood
chr1:161475219	225428	Whole Blood
chr1:161601752	85801	Whole Blood
chr1:161601752	325810	Whole Blood
chr1:161552879	36468	Whole Blood
chr1:161552879	98184	Whole Blood
chr1:161520526	164363	Whole Blood
chr1:161601752	45780	Whole Blood
chr1:161601752	78259	Whole Blood
chr1:161552879	20642	Whole Blood
chr1:161601752	166104	Whole Blood
chr1:161475219	66823	Whole Blood
chr1:161520526	52690	Whole Blood
chr1:161520526	43821	Whole Blood
chr1:161551100	20648	Whole Blood
chr1:161552879	148626	Whole Blood
chr1:161601752	303671	Whole Blood
chr1:161552879	47711	Whole Blood
chr1:161601752	193384	Whole Blood
chr1:161551100	377513	Whole Blood
chr1:161520526	50136	Whole Blood
chr1:161551100	99208	Whole Blood
chr1:161475219	54887	Whole Blood
chr1:161551100	36660	Whole Blood
chr1:161552879	104639	Whole Blood
chr1:161552879	127911	Whole Blood
chr1:161601752	27013	Whole Blood
chr1:161551100	356348	Whole Blood
chr1:161551100	42021	Whole Blood
chr1:161475219	222327	Whole Blood
chr1:161551100	42484	Whole Blood
chr1:161552879	371235	Whole Blood
chr1:161552879	36928	Whole Blood
chr1:161601752	28458	Whole Blood
chr1:161601752	21560	Whole Blood
chr1:161601752	169429	Whole Blood
chr1:161551100	37212	Whole Blood

chr1:161601752	31646	Whole Blood
chr1:161475219	131899	Whole Blood
chr1:161552879	70461	Whole Blood
chr1:161551100	280475	Whole Blood
chr1:161551100	318143	Whole Blood
chr1:161551100	61239	Whole Blood
chr1:161601752	232	Whole Blood
chr1:161551100	368877	Whole Blood
chr1:161475219	13723	Whole Blood
chr1:161520526	51415	Whole Blood
chr1:161551100	127081	Whole Blood
chr1:161551100	109952	Whole Blood
chr1:161601752	79038	Whole Blood
chr1:161520526	4575	Whole Blood
chr1:161601752	7192	Whole Blood
chr1:161601752	31550	Whole Blood
chr1:161475219	132298	Whole Blood
chr1:161552879	354569	Whole Blood
chr1:161552879	22213	Whole Blood
chr1:161601752	126792	Whole Blood
chr1:161520526	40928	Whole Blood
chr1:161552879	171423	Whole Blood
chr1:161520526	67553	Whole Blood
chr1:161552879	224063	Whole Blood
chr1:161552879	27313	Whole Blood
chr1:161552879	65428	Whole Blood
chr1:161551100	244636	Whole Blood
chr1:161475219	93928	Whole Blood
chr1:161551100	60205	Whole Blood
chr1:161551100	314787	Whole Blood
chr1:161551100	240635	Whole Blood
chr1:161475219	117902	Whole Blood
chr1:161552879	175047	Whole Blood
chr1:161520526	54219	Whole Blood
chr1:161552879	263439	Whole Blood
chr1:161552879	12444	Whole Blood
chr1:161552879	32576	Whole Blood
chr1:161520526	219629	Whole Blood
chr1:161601752	198683	Whole Blood
chr1:161601752	123045	Whole Blood
chr1:161551100	37033	Whole Blood
chr1:161601752	6560	Whole Blood
chr1:161520526	67234	Whole Blood
chr1:161551100	176826	Whole Blood
chr1:161551100	340504	Whole Blood
chr1:161601752	30950	Whole Blood
chr1:161475219	247429	Whole Blood
chr1:161520526	205492	Whole Blood
chr1:161551100	49490	Whole Blood
chr1:161475219	142146	Whole Blood

chr1:161520526	72595	Whole Blood
chr1:161601752	22066	Whole Blood
chr1:161601752	36429	Whole Blood
chr1:161552879	151112	Whole Blood
chr1:161551100	14280	Whole Blood
chr1:161552879	32516	Whole Blood
chr1:161552879	169925	Whole Blood
chr1:161551100	71656	Whole Blood
chr1:161552879	147827	Whole Blood
chr1:161552879	179239	Whole Blood
chr1:161552879	41681	Whole Blood
chr1:161475219	225429	Whole Blood
chr1:161551100	129685	Whole Blood
chr1:161552879	176820	Whole Blood
chr1:161520526	171221	Whole Blood
chr1:161475219	430215	Whole Blood
chr1:161551100	93286	Whole Blood
chr1:161475219	224026	Whole Blood
chr1:161552879	32417	Whole Blood
chr1:161551100	23639	Whole Blood
chr1:161601752	126174	Whole Blood
chr1:161551100	362302	Whole Blood
chr1:161552879	156578	Whole Blood
chr1:161520526	206414	Whole Blood
chr1:161475219	260989	Whole Blood
chr1:161475219	99408	Whole Blood
chr1:161551100	49008	Whole Blood
chr1:161601752	73689	Whole Blood
chr1:161552879	127552	Whole Blood
chr1:161601752	279755	Whole Blood
chr1:161601752	169296	Whole Blood
chr1:161552879	27385	Whole Blood
chr1:161551100	58434	Whole Blood
chr1:161551100	245321	Whole Blood
chr1:161551100	250455	Whole Blood
chr1:161520526	49580	Whole Blood
chr1:161552879	49455	Whole Blood
chr1:161552879	150356	Whole Blood
chr1:161551100	91971	Whole Blood
chr1:161520526	50276	Whole Blood
chr1:161551100	220081	Whole Blood
chr1:161601752	13992	Whole Blood
chr1:161475219	260832	Whole Blood
chr1:161601752	31090	Whole Blood
chr1:161552879	138868	Whole Blood
chr1:161551100	71663	Whole Blood
chr1:161520526	31584	Whole Blood
chr1:161552879	34136	Whole Blood
chr1:161551100	100504	Whole Blood
chr1:161551100	99462	Whole Blood

chr1:161552879	11490	Whole Blood
chr1:161552879	238460	Whole Blood
chr1:161551100	275158	Whole Blood
chr1:161601752	287640	Whole Blood
chr1:161475219	95583	Whole Blood
chr1:161520526	80217	Whole Blood
chr1:161520526	54622	Whole Blood
chr1:161601752	121052	Whole Blood
chr1:161520526	95019	Whole Blood
chr1:161551100	19006	Whole Blood
chr1:161520526	51222	Whole Blood
chr1:161520526	14090	Whole Blood
chr1:161475219	171405	Whole Blood
chr1:161601752	12405	Whole Blood
chr1:161552879	350772	Whole Blood
chr1:161475219	42465	Whole Blood
chr1:161601752	1162	Whole Blood
chr1:161601752	21588	Whole Blood
chr1:161552879	151593	Whole Blood
chr1:161601752	1104	Whole Blood
chr1:161601752	30450	Whole Blood
chr1:161552879	37073	Whole Blood
chr1:161552879	172354	Whole Blood
chr1:161520526	27860	Whole Blood
chr1:161552879	106208	Whole Blood
chr1:161601752	123481	Whole Blood
chr1:161475219	125898	Whole Blood
chr1:161551100	115600	Whole Blood
chr1:161552879	148327	Whole Blood
chr1:161601752	20952	Whole Blood
chr1:161601752	34564	Whole Blood
chr1:161551100	20841	Whole Blood
chr1:161551100	178599	Whole Blood
chr1:161552879	59530	Whole Blood
chr1:161601752	16357	Whole Blood
chr1:161601752	29811	Whole Blood
chr1:161552879	14314	Whole Blood
chr1:161551100	100760	Whole Blood
chr1:161551100	152891	Whole Blood
chr1:161520526	86828	Whole Blood
chr1:161520526	86991	Whole Blood
chr1:161520526	52233	Whole Blood
chr1:161552879	127906	Whole Blood
chr1:161601752	26306	Whole Blood
chr1:161551100	76140	Whole Blood
chr1:161551100	83264	Whole Blood
chr1:161552879	35993	Whole Blood
chr1:161520526	86592	Whole Blood
chr1:161520526	48621	Whole Blood
chr1:161601752	59300	Whole Blood

chr1:161475219	66851	Whole Blood
chr1:161601752	170059	Whole Blood
chr1:161552879	106965	Whole Blood
chr1:161601752	181908	Whole Blood
chr1:161552879	198985	Whole Blood
chr1:161551100	29092	Whole Blood
chr1:161551100	49548	Whole Blood
chr1:161601752	16456	Whole Blood
chr1:161601752	199803	Whole Blood
chr1:161552879	210146	Whole Blood
chr1:161552879	15282	Whole Blood
chr1:161475219	118742	Whole Blood
chr1:161551100	22421	Whole Blood
chr1:161601752	41319	Whole Blood
chr1:161475219	96083	Whole Blood
chr1:161552879	54881	Whole Blood
chr1:161601752	16297	Whole Blood
chr1:161552879	218302	Whole Blood
chr1:161520526	29631	Whole Blood
chr1:161551100	385517	Whole Blood
chr1:161551100	356995	Whole Blood
chr1:161552879	113821	Whole Blood
chr1:161551100	28586	Whole Blood
chr1:161520526	50776	Whole Blood
chr1:161551100	110559	Whole Blood
chr1:161601752	76558	Whole Blood
chr1:161520526	52768	Whole Blood
chr1:161551100	310409	Whole Blood
chr1:161552879	22567	Whole Blood
chr1:161552879	170508	Whole Blood
chr1:161601752	76429	Whole Blood
chr1:161552879	149504	Whole Blood
chr1:161601752	30004	Whole Blood
chr1:161551100	24704	Whole Blood
chr1:161475219	12232	Whole Blood
chr1:161475219	52844	Whole Blood
chr1:161551100	13269	Whole Blood
chr1:161551100	21659	Whole Blood
chr1:161551100	331088	Whole Blood
chr1:161520526	2842	Whole Blood
chr1:161601752	26660	Whole Blood
chr1:161551100	181018	Whole Blood
chr1:161475219	33397	Whole Blood
chr1:161551100	24346	Whole Blood
chr1:161551100	153372	Whole Blood
chr1:161551100	34295	Whole Blood
chr1:161551100	155526	Whole Blood
chr1:161520526	186100	Whole Blood
chr1:161552879	247556	Whole Blood
chr1:161520526	38932	Whole Blood

chr1:161551100	154770	Whole Blood
chr1:161552879	45431	Whole Blood
chr1:161520526	80064	Whole Blood
chr1:161551100	38247	Whole Blood
chr1:161551100	56417	Whole Blood
chr1:161551100	172287	Whole Blood
chr1:161551100	96432	Whole Blood
chr1:161552879	45107	Whole Blood
chr1:161601752	59907	Whole Blood
chr1:161475219	32229	Whole Blood
chr1:161601752	175190	Whole Blood
chr1:161601752	14737	Whole Blood
chr1:161552879	12501	Whole Blood
chr1:161552879	316364	Whole Blood
chr1:161551100	19102	Whole Blood
chr1:161552879	178094	Whole Blood
chr1:161551100	336547	Whole Blood
chr1:161552879	107515	Whole Blood
chr1:161552879	2290	Whole Blood
chr1:161601752	64948	Whole Blood
chr1:161601752	110857	Whole Blood
chr1:161552879	272245	Whole Blood
chr1:161520526	67786	Whole Blood
chr1:161551100	50420	Whole Blood
chr1:161552879	108780	Whole Blood
chr1:161601752	133916	Whole Blood
chr1:161475219	91969	Whole Blood
chr1:161520526	74034	Whole Blood
chr1:161551100	99407	Whole Blood
chr1:161601752	28993	Whole Blood
chr1:161520526	17071	Whole Blood
chr1:161552879	362210	Whole Blood
chr1:161475219	119341	Whole Blood
chr1:161552879	32526	Whole Blood
chr1:161551100	99963	Whole Blood
chr1:161551100	108744	Whole Blood
chr1:161520526	68634	Whole Blood
chr1:161520526	79582	Whole Blood
chr1:161551100	35915	Whole Blood
chr1:161601752	63012	Whole Blood
chr1:161520526	203722	Whole Blood
chr1:161601752	121639	Whole Blood
chr1:161551100	34196	Whole Blood
chr1:161551100	43460	Whole Blood
chr1:161601752	104874	Whole Blood
chr1:161552879	110455	Whole Blood
chr1:161601752	322362	Whole Blood
chr1:161551100	33416	Whole Blood
chr1:161475219	228215	Whole Blood
chr1:161601752	49311	Whole Blood

chr1:161601752	92990	Whole Blood
chr1:161475219	428432	Whole Blood
chr1:161601752	61582	Whole Blood
chr1:161475219	3570	Whole Blood
chr1:161475219	161107	Whole Blood
chr1:161475219	32704	Whole Blood
chr1:161601752	320775	Whole Blood
chr1:161551100	247608	Whole Blood
chr1:161551100	151283	Whole Blood
chr1:161551100	299824	Whole Blood
chr1:161601752	300071	Whole Blood
chr1:161552879	97628	Whole Blood
chr1:161551100	103847	Whole Blood
chr1:161551100	104415	Whole Blood
chr1:161551100	12595	Whole Blood
chr1:161601752	28246	Whole Blood
chr1:161552879	232826	Whole Blood
chr1:161551100	112234	Whole Blood
chr1:161552879	67552	Whole Blood
chr1:161552879	94653	Whole Blood
chr1:161601752	112810	Whole Blood
chr1:161552879	238856	Whole Blood
chr1:161551100	42338	Whole Blood
chr1:161601752	11800	Whole Blood
chr1:161551100	34355	Whole Blood
chr1:161520526	203600	Whole Blood
chr1:161520526	159485	Whole Blood
chr1:161552879	218169	Whole Blood
chr1:161475219	100318	Whole Blood
chr1:161552879	85838	Whole Blood
chr1:161551100	16093	Whole Blood
chr1:161601752	295288	Whole Blood
chr1:161551100	36996	Whole Blood
chr1:161552879	22888	Whole Blood
chr1:161551100	38852	Whole Blood
chr1:161551100	23992	Whole Blood
chr1:161552879	90783	Whole Blood
chr1:161475219	32228	Whole Blood
chr1:161551100	24048	Whole Blood
chr1:161551100	202405	Whole Blood
chr1:161552879	12023	Whole Blood
chr1:161520526	69930	Whole Blood
chr1:161601752	10587	Whole Blood
chr1:161552879	20627	Whole Blood
chr1:161520526	7537	Whole Blood
chr1:161552879	10195	Whole Blood
chr1:161551100	145045	Whole Blood
chr1:161601752	100631	Whole Blood
chr1:161551100	378836	Whole Blood
chr1:161551100	20994	Whole Blood

chr1:161552879	35012	Whole Blood
chr1:161551100	23037	Whole Blood
chr1:161475219	112541	Whole Blood
chr1:161552879	59596	Whole Blood
chr1:161520526	13079	Whole Blood
chr1:161520526	11764	Whole Blood
chr1:161475219	124889	Whole Blood
chr1:161601752	25985	Whole Blood
chr1:161601752	28231	Whole Blood
chr1:161520526	112158	Whole Blood
chr1:161601752	294811	Whole Blood
chr1:161601752	181538	Whole Blood
chr1:161475219	90738	Whole Blood
chr1:161601752	121635	Whole Blood
chr1:161552879	102636	Whole Blood
chr1:161475219	7383	Whole Blood
chr1:161552879	248755	Whole Blood
chr1:161475219	24623	Whole Blood
chr1:161551100	69331	Whole Blood
chr1:161520526	226517	Whole Blood
chr1:161552879	152991	Whole Blood
chr1:161601752	312312	Whole Blood
chr1:161552879	338725	Whole Blood
chr1:161552879	64940	Whole Blood
chr1:161552879	37102	Whole Blood
chr1:161552879	111885	Whole Blood
chr1:161551100	135625	Whole Blood
chr1:161520526	11910	Whole Blood
chr1:161551100	251320	Whole Blood
chr1:161552879	35377	Whole Blood
chr1:161520526	202122	Whole Blood
chr1:161601752	10723	Whole Blood
chr1:161551100	29164	Whole Blood
chr1:161551100	57212	Whole Blood
chr1:161475219	97540	Whole Blood
chr1:161520526	34643	Whole Blood
chr1:161520526	204707	Whole Blood
chr1:161601752	289852	Whole Blood
chr1:161552879	245938	Whole Blood
chr1:161520526	157895	Whole Blood
chr1:161520526	54213	Whole Blood
chr1:161551100	45139	Whole Blood
chr1:161551100	320691	Whole Blood
chr1:161601752	288578	Whole Blood
chr1:161601752	58642	Whole Blood
chr1:161520526	70186	Whole Blood
chr1:161552879	218857	Whole Blood
chr1:161601752	60252	Whole Blood
chr1:161552879	16689	Whole Blood
chr1:161475219	100541	Whole Blood

chr1:161601752	26145	Whole Blood
chr1:161520526	68888	Whole Blood
chr1:161520526	49676	Whole Blood
chr1:161552879	150519	Whole Blood
chr1:161601752	27099	Whole Blood
chr1:161552879	102068	Whole Blood
chr1:161601752	76624	Whole Blood
chr1:161551100	35149	Whole Blood
chr1:161551100	34305	Whole Blood
chr1:161520526	44854	Whole Blood
chr1:161552879	355216	Whole Blood
chr1:161551100	113664	Whole Blood
chr1:161552879	47712	Whole Blood
chr1:161520526	403588	Whole Blood
chr1:161552879	250526	Whole Blood
chr1:161520526	59738	Whole Blood
chr1:161601752	57335	Whole Blood
chr1:161551100	102860	Whole Blood
chr1:161552879	243542	Whole Blood
chr1:161552879	308630	Whole Blood
chr1:161601752	6008	Whole Blood
chr1:161552879	248676	Whole Blood
chr1:161551100	61375	Whole Blood
chr1:161601752	26009	Whole Blood
chr1:161552879	92669	Whole Blood
chr1:161520526	68821	Whole Blood
chr1:161520526	13078	Whole Blood
chr1:161551100	172291	Whole Blood
chr1:161552879	7293	Whole Blood
chr1:161551100	152298	Whole Blood
chr1:161551100	109294	Whole Blood
chr1:161520526	87786	Whole Blood
chr1:161551100	211925	Whole Blood
chr1:161551100	22194	Whole Blood
chr1:161475219	114128	Whole Blood
chr1:161601752	127947	Whole Blood
chr1:161520526	54566	Whole Blood
chr1:161551100	146847	Whole Blood
chr1:161475219	4379	Whole Blood
chr1:161520526	46662	Whole Blood
chr1:161520526	87234	Whole Blood
chr1:161520526	44797	Whole Blood
chr1:161551100	110904	Whole Blood
chr1:161520526	39646	Whole Blood
chr1:161520526	52995	Whole Blood
chr1:161552879	133846	Whole Blood
chr1:161552879	200626	Whole Blood
chr1:161551100	51234	Whole Blood
chr1:161551100	14223	Whole Blood
chr1:161552879	44956	Whole Blood

chr1:161601752	151753	Whole Blood
chr1:161601752	41910	Whole Blood
chr1:161475219	95443	Whole Blood
chr1:161520526	210447	Whole Blood
chr1:161475219	40732	Whole Blood
chr1:161552879	337451	Whole Blood
chr1:161551100	99983	Whole Blood
chr1:161601752	13496	Whole Blood
chr1:161551100	174133	Whole Blood
chr1:161475219	89128	Whole Blood
chr1:161520526	80591	Whole Blood
chr1:161601752	37383	Whole Blood
chr1:161552879	21774	Whole Blood
chr1:161552879	36888	Whole Blood
chr1:161520526	67607	Whole Blood
chr1:161551100	146548	Whole Blood
chr1:161552879	229395	Whole Blood
chr1:161551100	249886	Whole Blood
chr1:161520526	80122	Whole Blood
chr1:161475219	96722	Whole Blood
chr1:161551100	179873	Whole Blood
chr1:161552879	125431	Whole Blood
chr1:161601752	122154	Whole Blood
chr1:161552879	250548	Whole Blood
chr1:161551100	107987	Whole Blood
chr1:161551100	61309	Whole Blood
chr1:161552879	172179	Whole Blood
chr1:161520526	157655	Whole Blood
chr1:161551100	37772	Whole Blood
chr1:161520526	76597	Whole Blood
chr1:161551100	46886	Whole Blood
chr1:161551100	284526	Whole Blood
chr1:161551100	22406	Whole Blood
chr1:161475219	23327	Whole Blood
chr1:161520526	205265	Whole Blood
chr1:161601752	41372	Whole Blood
chr1:161601752	42231	Whole Blood
chr1:161475219	96529	Whole Blood
chr1:161601752	16347	Whole Blood
chr1:161601752	10657	Whole Blood
chr1:161551100	16484	Whole Blood
chr1:161601752	41580	Whole Blood
chr1:161601752	130366	Whole Blood
chr1:161520526	123116	Whole Blood
chr1:161551100	366728	Whole Blood
chr1:161601752	150635	Whole Blood
chr1:161601752	58092	Whole Blood
chr1:161520526	157208	Whole Blood
chr1:161520526	67570	Whole Blood
chr1:161475219	110236	Whole Blood

chr1:161601752	34559	Whole Blood
chr1:161601752	12880	Whole Blood
chr1:161601752	402322	Whole Blood
chr1:161475219	132135	Whole Blood
chr1:161520526	139826	Whole Blood
chr1:161475219	94887	Whole Blood
chr1:161601752	3766	Whole Blood
chr1:161601752	285895	Whole Blood
chr1:161552879	67148	Whole Blood
chr1:161601752	109086	Whole Blood
chr1:161552879	107366	Whole Blood
chr1:161520526	55241	Whole Blood
chr1:161552879	89549	Whole Blood
chr1:161520526	81808	Whole Blood
chr1:161520526	123860	Whole Blood
chr1:161552879	170512	Whole Blood
chr1:161601752	180522	Whole Blood
chr1:161551100	127210	Whole Blood
chr1:161520526	59666	Whole Blood
chr1:161520526	59160	Whole Blood
chr1:161552879	27921	Whole Blood
chr1:161520526	77460	Whole Blood
chr1:161601752	282584	Whole Blood
chr1:161551100	57200	Whole Blood
chr1:161475219	90104	Whole Blood
chr1:161552879	293664	Whole Blood
chr1:161601752	95791	Whole Blood
chr1:161601752	188239	Whole Blood
chr1:161551100	68927	Whole Blood
chr1:161601752	13861	Whole Blood
chr1:161601752	196956	Whole Blood
chr1:161601752	16067	Whole Blood
chr1:161551100	62158	Whole Blood
chr1:161520526	159905	Whole Blood
chr1:161601752	299421	Whole Blood
chr1:161601752	299399	Whole Blood
chr1:161601752	193356	Whole Blood
chr1:161552879	22864	Whole Blood
chr1:161551100	37156	Whole Blood
chr1:161601752	18679	Whole Blood
chr1:161551100	23447	Whole Blood
chr1:161552879	109125	Whole Blood
chr1:161552879	23890	Whole Blood
chr1:161601752	53763	Whole Blood
chr1:161601752	141542	Whole Blood
chr1:161551100	11974	Whole Blood
chr1:161520526	91883	Whole Blood
chr1:161601752	44225	Whole Blood
chr1:161551100	312865	Whole Blood
chr1:161601752	46583	Whole Blood

chr1:161552879	44165	Whole Blood
chr1:161601752	150112	Whole Blood
chr1:161552879	227821	Whole Blood
chr1:161551100	427144	Whole Blood
chr1:161520526	102230	Whole Blood
chr1:161551100	9072	Whole Blood
chr1:161475219	30742	Whole Blood
chr1:161520526	43169	Whole Blood
chr1:161551100	231932	Whole Blood
chr1:161552879	178276	Whole Blood
chr1:161551100	24643	Whole Blood
chr1:161551100	66719	Whole Blood
chr1:161551100	111383	Whole Blood
chr1:161551100	72240	Whole Blood
chr1:161475219	113093	Whole Blood
chr1:161520526	64929	Whole Blood
chr1:161551100	256839	Whole Blood
chr1:161520526	43843	Whole Blood
chr1:161552879	255060	Whole Blood
chr1:161552879	102782	Whole Blood
chr1:161475219	448895	Whole Blood
chr1:161601752	151412	Whole Blood
chr1:161552879	22728	Whole Blood
chr1:161551100	23553	Whole Blood
chr1:161551100	4069	Whole Blood
chr1:161551100	234605	Whole Blood
chr1:161520526	91813	Whole Blood
chr1:161601752	93136	Whole Blood
chr1:161601752	267730	Whole Blood
chr1:161601752	206199	Whole Blood
chr1:161601752	67136	Whole Blood
chr1:161551100	56660	Whole Blood
chr1:161475219	140326	Whole Blood
chr1:161552879	154622	Whole Blood
chr1:161601752	78679	Whole Blood
chr1:161601752	297628	Whole Blood
chr1:161552879	10534	Whole Blood
chr1:161520526	67730	Whole Blood
chr1:161551100	244159	Whole Blood
chr1:161552879	10175	Whole Blood
chr1:161552879	230781	Whole Blood
chr1:161552879	162364	Whole Blood
chr1:161475219	99526	Whole Blood
chr1:161520526	20330	Whole Blood
chr1:161551100	49491	Whole Blood
chr1:161551100	238891	Whole Blood
chr1:161551100	111549	Whole Blood
chr1:161601752	582	Whole Blood
chr1:161552879	9966	Whole Blood
chr1:161475219	105045	Whole Blood

chr1:161552879	66481	Whole Blood
chr1:161475219	23581	Whole Blood
chr1:161601752	11985	Whole Blood
chr1:161552879	13164	Whole Blood
chr1:161520526	12603	Whole Blood
chr1:161552879	48381	Whole Blood
chr1:161552879	90245	Whole Blood
chr1:161552879	278696	Whole Blood
chr1:161552879	29556	Whole Blood
chr1:161551100	38881	Whole Blood
chr1:161552879	375734	Whole Blood
chr1:161520526	54920	Whole Blood
chr1:161601752	183953	Whole Blood
chr1:161520526	60274	Whole Blood
chr1:161552879	264286	Whole Blood
chr1:161601752	178948	Whole Blood
chr1:161520526	207090	Whole Blood
chr1:161520526	69409	Whole Blood
chr1:161601752	101646	Whole Blood
chr1:161551100	24667	Whole Blood
chr1:161601752	200668	Whole Blood
chr1:161475219	111796	Whole Blood
chr1:161520526	112130	Whole Blood
chr1:161551100	127276	Whole Blood
chr1:161601752	129221	Whole Blood
chr1:161551100	154799	Whole Blood
chr1:161551100	43653	Whole Blood
chr1:161601752	157005	Whole Blood
chr1:161520526	68346	Whole Blood
chr1:161520526	401838	Whole Blood
chr1:161552879	44384	Whole Blood
chr1:161601752	18275	Whole Blood
chr1:161520526	46667	Whole Blood
chr1:161601752	18336	Whole Blood
chr1:161601752	53195	Whole Blood
chr1:161551100	29700	Whole Blood
chr1:161552879	17648	Whole Blood
chr1:161601752	221052	Whole Blood
chr1:161475219	185779	Whole Blood
chr1:161551100	229600	Whole Blood
chr1:161475219	125775	Whole Blood
chr1:161601752	29400	Whole Blood
chr1:161520526	232979	Whole Blood
chr1:161601752	102239	Whole Blood
chr1:161601752	6023	Whole Blood
chr1:161551100	370660	Whole Blood
chr1:161552879	298045	Whole Blood
chr1:161520526	141133	Whole Blood
chr1:161601752	492	Whole Blood
chr1:161552879	20421	Whole Blood

chr1:161601752	32184	Whole Blood
chr1:161520526	66489	Whole Blood
chr1:161601752	93829	Whole Blood
chr1:161520526	386922	Whole Blood
chr1:161551100	399976	Whole Blood
chr1:161601752	11771	Whole Blood
chr1:161551100	383851	Whole Blood
chr1:161601752	84068	Whole Blood
chr1:161520526	202081	Whole Blood
chr1:161551100	18468	Whole Blood
chr1:161551100	247558	Whole Blood
chr1:161601752	127687	Whole Blood
chr1:161552879	233711	Whole Blood
chr1:161601752	299935	Whole Blood
chr1:161601752	223694	Whole Blood
chr1:161601752	35977	Whole Blood
chr1:161552879	355997	Whole Blood
chr1:161475219	90161	Whole Blood
chr1:161520526	134421	Whole Blood
chr1:161601752	14772	Whole Blood
chr1:161551100	231174	Whole Blood
chr1:161601752	334734	Whole Blood
chr1:161520526	69241	Whole Blood
chr1:161551100	324781	Whole Blood
chr1:161475219	24879	Whole Blood
chr1:161601752	149860	Whole Blood
chr1:161551100	87770	Whole Blood
chr1:161601752	194669	Whole Blood
chr1:161475219	79950	Whole Blood
chr1:161601752	58839	Whole Blood
chr1:161551100	143959	Whole Blood
chr1:161520526	214062	Whole Blood
chr1:161552879	103727	Whole Blood
chr1:161520526	80994	Whole Blood
chr1:161552879	152193	Whole Blood
chr1:161551100	390718	Whole Blood
chr1:161552879	21668	Whole Blood
chr1:161551100	24507	Whole Blood
chr1:161601752	28452	Whole Blood
chr1:161552879	42850	Whole Blood
chr1:161551100	112722	Whole Blood
chr1:161552879	237112	Whole Blood
chr1:161601752	102720	Whole Blood
chr1:161601752	161273	Whole Blood
chr1:161552879	110943	Whole Blood
chr1:161552879	125497	Whole Blood
chr1:161551100	92883	Whole Blood
chr1:161520526	102814	Whole Blood
chr1:161475219	413049	Whole Blood
chr1:161551100	385386	Whole Blood

chr1:161601752	60731	Whole Blood
chr1:161601752	1121	Whole Blood
chr1:161552879	34101	Whole Blood
chr1:161520526	14565	Whole Blood
chr1:161601752	51801	Whole Blood
chr1:161520526	55217	Whole Blood
chr1:161601752	206187	Whole Blood
chr1:161601752	65907	Whole Blood
chr1:161551100	248747	Whole Blood
chr1:161520526	369804	Whole Blood
chr1:161601752	195697	Whole Blood
chr1:161520526	138561	Whole Blood
chr1:161601752	120158	Whole Blood
chr1:161601752	1161	Whole Blood
chr1:161551100	90890	Whole Blood
chr1:161520526	64879	Whole Blood
chr1:161551100	63649	Whole Blood
chr1:161520526	139318	Whole Blood
chr1:161601752	127880	Whole Blood
chr1:161551100	146048	Whole Blood
chr1:161601752	150114	Whole Blood
chr1:161520526	98834	Whole Blood
chr1:161475219	26979	Whole Blood
chr1:161552879	109770	Whole Blood
chr1:161552879	48443	Whole Blood
chr1:161520526	33075	Whole Blood
chr1:161551100	22226	Whole Blood
chr1:161475219	137120	Whole Blood
chr1:161551100	232560	Whole Blood
chr1:161552879	47752	Whole Blood
chr1:161552879	91104	Whole Blood
chr1:161551100	50160	Whole Blood
chr1:161601752	74137	Whole Blood
chr1:161551100	248769	Whole Blood
chr1:161551100	178339	Whole Blood
chr1:161552879	55421	Whole Blood
chr1:161475219	110077	Whole Blood
chr1:161551100	6427	Whole Blood
chr1:161520526	102308	Whole Blood
chr1:161552879	313008	Whole Blood
chr1:161601752	48755	Whole Blood
chr1:161552879	20447	Whole Blood
chr1:161552879	59349	Whole Blood
chr1:161551100	232190	Whole Blood
chr1:161552879	89279	Whole Blood
chr1:161552879	360523	Whole Blood
chr1:161601752	38678	Whole Blood
chr1:161601752	38698	Whole Blood
chr1:161475219	98287	Whole Blood
chr1:161551100	115532	Whole Blood

chr1:161520526	54127	Whole Blood
chr1:161552879	44941	Whole Blood
chr1:161552879	19473	Whole Blood
chr1:161552879	383738	Whole Blood
chr1:161520526	202865	Whole Blood
chr1:161601752	286475	Whole Blood
chr1:161551100	148577	Whole Blood
chr1:161475219	125524	Whole Blood
chr1:161475219	113037	Whole Blood
chr1:161551100	68260	Whole Blood
chr1:161551100	350723	Whole Blood
chr1:161520526	64770	Whole Blood
chr1:161520526	367742	Whole Blood
chr1:161551100	270466	Whole Blood
chr1:161552879	17477	Whole Blood
chr1:161551100	207657	Whole Blood
chr1:161601752	64880	Whole Blood
chr1:161520526	181857	Whole Blood
chr1:161551100	294432	Whole Blood
chr1:161551100	84059	Whole Blood
chr1:161551100	115021	Whole Blood
chr1:161552879	245829	Whole Blood
chr1:161551100	36791	Whole Blood
chr1:161475219	100227	Whole Blood
chr1:161552879	251062	Whole Blood
chr1:161601752	288316	Whole Blood
chr1:161551100	120951	Whole Blood
chr1:161601752	115189	Whole Blood
chr1:161601752	31396	Whole Blood
chr1:161601752	60897	Whole Blood
chr1:161551100	92562	Whole Blood
chr1:161551100	11954	Whole Blood
chr1:161601752	151156	Whole Blood
chr1:161552879	4648	Whole Blood
chr1:161601752	60896	Whole Blood
chr1:161601752	17608	Whole Blood
chr1:161475219	94519	Whole Blood
chr1:161551100	13503	Whole Blood
chr1:161551100	38667	Whole Blood
chr1:161601752	72763	Whole Blood
chr1:161552879	16150	Whole Blood
chr1:161475219	247388	Whole Blood
chr1:161601752	27467	Whole Blood
chr1:161552879	109604	Whole Blood
chr1:161551100	107408	Whole Blood
chr1:161520526	67365	Whole Blood
chr1:161601752	189849	Whole Blood
chr1:161552879	59353	Whole Blood
chr1:161551100	124789	Whole Blood
chr1:161552879	58144	Whole Blood

chr1:161551100	110388	Whole Blood
chr1:161520526	11812	Whole Blood
chr1:161551100	49531	Whole Blood
chr1:161601752	105749	Whole Blood
chr1:161475219	104467	Whole Blood
chr1:161551100	87500	Whole Blood
chr1:161475219	59397	Whole Blood
chr1:161551100	361628	Whole Blood
chr1:161552879	12896	Whole Blood
chr1:161551100	10244	Whole Blood
chr1:161551100	43652	Whole Blood
chr1:161552879	248107	Whole Blood
chr1:161601752	259757	Whole Blood
chr1:161520526	49830	Whole Blood
chr1:161601752	313159	Whole Blood
chr1:161475219	216528	Whole Blood
chr1:161601752	3932	Whole Blood
chr1:161551100	181211	Whole Blood
chr1:161601752	188794	Whole Blood
chr1:161475219	94983	Whole Blood
chr1:161552879	36779	Whole Blood
chr1:161601752	28426	Whole Blood
chr1:161551100	110310	Whole Blood
chr1:161551100	217078	Whole Blood
chr1:161551100	35880	Whole Blood
chr1:161520526	91949	Whole Blood
chr1:161520526	22387	Whole Blood
chr1:161520526	37001	Whole Blood
chr1:161601752	4489	Whole Blood
chr1:161551100	156401	Whole Blood
chr1:161551100	14675	Whole Blood
chr1:161551100	42386	Whole Blood
chr1:161551100	116559	Whole Blood
chr1:161475219	105581	Whole Blood
chr1:161552879	125374	Whole Blood
chr1:161475219	246047	Whole Blood
chr1:161601752	32723	Whole Blood
chr1:161601752	13642	Whole Blood
chr1:161601752	430	Whole Blood
chr1:161520526	69426	Whole Blood
chr1:161551100	274346	Whole Blood
chr1:161552879	62826	Whole Blood
chr1:161552879	145738	Whole Blood
chr1:161601752	13617	Whole Blood
chr1:161552879	230411	Whole Blood
chr1:161601752	47946	Whole Blood
chr1:161475219	88476	Whole Blood
chr1:161551100	88184	Whole Blood
chr1:161601752	48236	Whole Blood
chr1:161475219	250572	Whole Blood

chr1:161601752	229823	Whole Blood
chr1:161601752	94304	Whole Blood
chr1:161552879	145361	Whole Blood
chr1:161475219	99873	Whole Blood
chr1:161552879	12599	Whole Blood
chr1:161520526	371078	Whole Blood
chr1:161601752	103320	Whole Blood
chr1:161552879	44297	Whole Blood
chr1:161551100	143582	Whole Blood
chr1:161551100	239446	Whole Blood
chr1:161551100	228839	Whole Blood
chr1:161552879	114780	Whole Blood
chr1:161601752	62070	Whole Blood
chr1:161552879	148329	Whole Blood
chr1:161552879	323002	Whole Blood
chr1:161475219	11889	Whole Blood
chr1:161551100	38486	Whole Blood
chr1:161601752	1695	Whole Blood
chr1:161520526	57196	Whole Blood
chr1:161551100	387777	Whole Blood
chr1:161475219	125429	Whole Blood
chr1:161552879	100674	Whole Blood
chr1:161551100	170400	Whole Blood
chr1:161520526	69455	Whole Blood
chr1:161520526	142808	Whole Blood
chr1:161601752	194234	Whole Blood
chr1:161601752	94305	Whole Blood
chr1:161601752	318225	Whole Blood
chr1:161601752	27205	Whole Blood
chr1:161601752	199234	Whole Blood
chr1:161551100	49175	Whole Blood
chr1:161552879	19450	Whole Blood
chr1:161475219	88334	Whole Blood
chr1:161520526	52980	Whole Blood
chr1:161520526	281029	Whole Blood
chr1:161475219	141197	Whole Blood
chr1:161475219	87835	Whole Blood
chr1:161475219	6375	Whole Blood
chr1:161551100	14943	Whole Blood
chr1:161601752	234807	Whole Blood
chr1:161601752	84973	Whole Blood
chr1:161520526	64869	Whole Blood
chr1:161551100	92024	Whole Blood
chr1:161552879	329309	Whole Blood
chr1:161551100	43177	Whole Blood
chr1:161520526	75203	Whole Blood
chr1:161552879	47952	Whole Blood
chr1:161551100	227782	Whole Blood
chr1:161551100	98888	Whole Blood
chr1:161475219	62378	Whole Blood

chr1:161551100	50222	Whole Blood
chr1:161520526	60316	Whole Blood
chr1:161552879	249541	Whole Blood
chr1:161520526	118003	Whole Blood
chr1:161601752	316076	Whole Blood
chr1:161551100	102636	Whole Blood
chr1:161552879	50568	Whole Blood
chr1:161551100	107557	Whole Blood
chr1:161601752	36372	Whole Blood
chr1:161601752	16792	Whole Blood
chr1:161520526	49042	Whole Blood
chr1:161601752	313337	Whole Blood
chr1:161520526	209173	Whole Blood
chr1:161475219	143495	Whole Blood
chr1:161520526	55081	Whole Blood
chr1:161552879	79007	Whole Blood
chr1:161601752	59658	Whole Blood
chr1:161551100	160585	Whole Blood
chr1:161520526	263134	Whole Blood
chr1:161551100	102453	Whole Blood
chr1:161552879	66878	Whole Blood
chr1:161551100	38558	Whole Blood
chr1:161601752	59407	Whole Blood
chr1:161520526	280460	Whole Blood
chr1:161601752	76501	Whole Blood
chr1:161601752	194611	Whole Blood
chr1:161520526	115474	Whole Blood
chr1:161551100	362366	Whole Blood
chr1:161552879	388939	Whole Blood
chr1:161552879	176560	Whole Blood
chr1:161551100	384228	Whole Blood
chr1:161552879	575272	Whole Blood
chr1:161551100	23185	Whole Blood
chr1:161551100	71502	Whole Blood
chr1:161601752	13953	Whole Blood
chr1:161601752	51984	Whole Blood
chr1:161552879	64852	Whole Blood
chr1:161552879	36707	Whole Blood
chr1:161601752	270039	Whole Blood
chr1:161520526	53485	Whole Blood
chr1:161551100	103742	Whole Blood
chr1:161520526	321096	Whole Blood
chr1:161551100	105587	Whole Blood
chr1:161601752	921	Whole Blood
chr1:161601752	15979	Whole Blood
chr1:161520526	124225	Whole Blood
chr1:161475219	125371	Whole Blood
chr1:161520526	98188	Whole Blood
chr1:161551100	176497	Whole Blood
chr1:161601752	72317	Whole Blood

chr1:161601752	64155	Whole Blood
chr1:161551100	302019	Whole Blood
chr1:161520526	14705	Whole Blood
chr1:161552879	21406	Whole Blood
chr1:161552879	71285	Whole Blood
chr1:161520526	8463	Whole Blood
chr1:161520526	122545	Whole Blood
chr1:161475219	8178	Whole Blood
chr1:161551100	389939	Whole Blood
chr1:161520526	279909	Whole Blood
chr1:161520526	211592	Whole Blood
chr1:161551100	246976	Whole Blood
chr1:161551100	359646	Whole Blood
chr1:161551100	22200	Whole Blood
chr1:161601752	4576	Whole Blood
chr1:161475219	91974	Whole Blood
chr1:161552879	164062	Whole Blood
chr1:161552879	364949	Whole Blood
chr1:161551100	52347	Whole Blood
chr1:161551100	291885	Whole Blood
chr1:161552879	47396	Whole Blood
chr1:161475219	113653	Whole Blood
chr1:161551100	116557	Whole Blood
chr1:161552879	311086	Whole Blood
chr1:161475219	447145	Whole Blood
chr1:161601752	1477	Whole Blood
chr1:161520526	46654	Whole Blood
chr1:161601752	6407	Whole Blood
chr1:161520526	251285	Whole Blood
chr1:161601752	333199	Whole Blood
chr1:161551100	107720	Whole Blood
chr1:161475219	168278	Whole Blood
chr1:161551100	98598	Whole Blood
chr1:161601752	58965	Whole Blood
chr1:161551100	21252	Whole Blood
chr1:161520526	80105	Whole Blood
chr1:161551100	349702	Whole Blood
chr1:161551100	122969	Whole Blood
chr1:161601752	57706	Whole Blood
chr1:161520526	113008	Whole Blood
chr1:161520526	160264	Whole Blood
chr1:161552879	425365	Whole Blood
chr1:161520526	42548	Whole Blood
chr1:161552879	44326	Whole Blood
chr1:161552879	282747	Whole Blood
chr1:161551100	146550	Whole Blood
chr1:161552879	348944	Whole Blood
chr1:161552879	16122	Whole Blood
chr1:161601752	6548	Whole Blood
chr1:161552879	368881	Whole Blood

chr1:161601752	320008	Whole Blood
chr1:161475219	24102	Whole Blood
chr1:161520526	90497	Whole Blood
chr1:161551100	127153	Whole Blood
chr1:161552879	14562	Whole Blood
chr1:161552879	205878	Whole Blood
chr1:161601752	4630	Whole Blood
chr1:161475219	112914	Whole Blood
chr1:161551100	249283	Whole Blood
chr1:161551100	14378	Whole Blood
chr1:161551100	57059	Whole Blood
chr1:161601752	78429	Whole Blood
chr1:161601752	65905	Whole Blood
chr1:161601752	9271	Whole Blood
chr1:161601752	233874	Whole Blood
chr1:161520526	123136	Whole Blood
chr1:161552879	69590	Whole Blood
chr1:161520526	183465	Whole Blood
chr1:161520526	157850	Whole Blood
chr1:161552879	119172	Whole Blood
chr1:161552879	113753	Whole Blood
chr1:161475219	261052	Whole Blood
chr1:161475219	70966	Whole Blood
chr1:161520526	127006	Whole Blood
chr1:161551100	108358	Whole Blood
chr1:161551100	101003	Whole Blood
chr1:161520526	114471	Whole Blood
chr1:161601752	104118	Whole Blood
chr1:161520526	97293	Whole Blood
chr1:161552879	627739	Whole Blood
chr1:161520526	157784	Whole Blood
chr1:161552879	15034	Whole Blood
chr1:161551100	165841	Whole Blood
chr1:161520526	42528	Whole Blood
chr1:161551100	155048	Whole Blood
chr1:161552879	383607	Whole Blood
chr1:161552879	97109	Whole Blood
chr1:161552879	123010	Whole Blood
chr1:161551100	26556	Whole Blood
chr1:161552879	185233	Whole Blood
chr1:161475219	249029	Whole Blood
chr1:161551100	15935	Whole Blood
chr1:161551100	59923	Whole Blood
chr1:161551100	44629	Whole Blood
chr1:161520526	80065	Whole Blood
chr1:161520526	202278	Whole Blood
chr1:161552879	318912	Whole Blood
chr1:161601752	79033	Whole Blood
chr1:161520526	146174	Whole Blood
chr1:161552879	292653	Whole Blood

chr1:161552879	65665	Whole Blood
chr1:161475219	137256	Whole Blood
chr1:161601752	12166	Whole Blood
chr1:161551100	47797	Whole Blood
chr1:161551100	23189	Whole Blood
chr1:161601752	6673	Whole Blood
chr1:161551100	49731	Whole Blood
chr1:161551100	67444	Whole Blood
chr1:161551100	262507	Whole Blood
chr1:161601752	10480	Whole Blood
chr1:161520526	70429	Whole Blood
chr1:161601752	34717	Whole Blood
chr1:161475219	69164	Whole Blood
chr1:161520526	35268	Whole Blood
chr1:161551100	153972	Whole Blood
chr1:161552879	14156	Whole Blood
chr1:161551100	46163	Whole Blood
chr1:161552879	237667	Whole Blood
chr1:161551100	376177	Whole Blood
chr1:161551100	61132	Whole Blood
chr1:161552879	239443	Whole Blood
chr1:161551100	57211	Whole Blood
chr1:161601752	64995	Whole Blood
chr1:161475219	168755	Whole Blood
chr1:161551100	625960	Whole Blood
chr1:161475219	202515	Whole Blood
chr1:161601752	114301	Whole Blood
chr1:161601752	280436	Whole Blood
chr1:161551100	33372	Whole Blood
chr1:161601752	130559	Whole Blood
chr1:161520526	99501	Whole Blood
chr1:161601752	64369	Whole Blood
chr1:161601752	134711	Whole Blood
chr1:161520526	2797	Whole Blood
chr1:161601752	311714	Whole Blood
chr1:161520526	144238	Whole Blood
chr1:161551100	69506	Whole Blood
chr1:161601752	40519	Whole Blood
chr1:161551100	390085	Whole Blood
chr1:161551100	27777	Whole Blood
chr1:161551100	109105	Whole Blood
chr1:161551100	64269	Whole Blood
chr1:161552879	367098	Whole Blood
chr1:161552879	86405	Whole Blood
chr1:161475219	147615	Whole Blood
chr1:161551100	19256	Whole Blood
chr1:161552879	157854	Whole Blood
chr1:161520526	261748	Whole Blood
chr1:161551100	22111	Whole Blood
chr1:161551100	68988	Whole Blood

chr1:161601752	12094	Whole Blood
chr1:161551100	101948	Whole Blood
chr1:161552879	121190	Whole Blood
chr1:161475219	292637	Whole Blood
chr1:161520526	45517	Whole Blood
chr1:161475219	11619	Whole Blood
chr1:161601752	47730	Whole Blood
chr1:161551100	46022	Whole Blood
chr1:161601752	37532	Whole Blood
chr1:161552879	48540	Whole Blood
chr1:161601752	334865	Whole Blood
chr1:161601752	44599	Whole Blood
chr1:161520526	71374	Whole Blood
chr1:161520526	16231	Whole Blood
chr1:161552879	62490	Whole Blood
chr1:161551100	50159	Whole Blood
chr1:161552879	44243	Whole Blood
chr1:161552879	8354	Whole Blood
chr1:161520526	130537	Whole Blood
chr1:161520526	231338	Whole Blood
chr1:161520526	116273	Whole Blood
chr1:161475219	29706	Whole Blood
chr1:161552879	388160	Whole Blood
chr1:161601752	10476	Whole Blood
chr1:161552879	45398	Whole Blood
chr1:161552879	15106	Whole Blood
chr1:161475219	98302	Whole Blood
chr1:161475219	248907	Whole Blood
chr1:161520526	203776	Whole Blood
chr1:161520526	183946	Whole Blood
chr1:161520526	166199	Whole Blood
chr1:161520526	72286	Whole Blood
chr1:161551100	17929	Whole Blood
chr1:161552879	108531	Whole Blood
chr1:161520526	139868	Whole Blood
chr1:161551100	46076	Whole Blood
chr1:161520526	141478	Whole Blood
chr1:161552879	109187	Whole Blood
chr1:161520526	140526	Whole Blood
chr1:161601752	34311	Whole Blood
chr1:161552879	73281	Whole Blood
chr1:161552879	4274	Whole Blood
chr1:161520526	186504	Whole Blood
chr1:161552879	46018	Whole Blood
chr1:161520526	213585	Whole Blood
chr1:161552879	67209	Whole Blood
chr1:161601752	78765	Whole Blood
chr1:161552879	42200	Whole Blood
chr1:161601752	12900	Whole Blood
chr1:161520526	79749	Whole Blood

chr1:161601752	17280	Whole Blood
chr1:161520526	186975	Whole Blood
chr1:161520526	207400	Whole Blood
chr1:161552879	11757	Whole Blood
chr1:161520526	69132	Whole Blood
chr1:161552879	213304	Whole Blood
chr1:161475219	26067	Whole Blood
chr1:161475219	99520	Whole Blood
chr1:161520526	21819	Whole Blood
chr1:161552879	96603	Whole Blood
chr1:161601752	59736	Whole Blood
chr1:161601752	2855	Whole Blood
chr1:161475219	98075	Whole Blood
chr1:161552879	36746	Whole Blood
chr1:161551100	390287	Whole Blood
chr1:161520526	80734	Whole Blood
chr1:161601752	458	Whole Blood
chr1:161601752	264135	Whole Blood
chr1:161520526	218709	Whole Blood
chr1:161520526	218173	Whole Blood
chr1:161601752	267491	Whole Blood
chr1:161520526	256416	Whole Blood
chr1:161552879	35973	Whole Blood
chr1:161552879	179432	Whole Blood
chr1:161520526	99905	Whole Blood
chr1:161520526	160259	Whole Blood
chr1:161601752	6559	Whole Blood
chr1:161552879	113242	Whole Blood
chr1:161551100	64294	Whole Blood
chr1:161601752	313155	Whole Blood
chr1:161551100	39176	Whole Blood
chr1:161475219	89150	Whole Blood
chr1:161551100	124893	Whole Blood
chr1:161551100	122831	Whole Blood
chr1:161552879	114778	Whole Blood
chr1:161601752	156239	Whole Blood
chr1:161552879	127638	Whole Blood
chr1:161552879	20247	Whole Blood
chr1:161520526	394563	Whole Blood
chr1:161520526	134989	Whole Blood
chr1:161475219	432229	Whole Blood
chr1:161520526	122598	Whole Blood
chr1:161551100	61128	Whole Blood
chr1:161601752	1350	Whole Blood
chr1:161551100	51417	Whole Blood
chr1:161601752	333	Whole Blood
chr1:161551100	16885	Whole Blood
chr1:161601752	58453	Whole Blood
chr1:161551100	426506	Whole Blood
chr1:161601752	249172	Whole Blood

chr1:161551100	46105	Whole Blood
chr1:161520526	76834	Whole Blood
chr1:161552879	360587	Whole Blood
chr1:161475219	203202	Whole Blood
chr1:161601752	28626	Whole Blood
chr1:161552879	369648	Whole Blood
chr1:161520526	402001	Whole Blood
chr1:161551100	113482	Whole Blood
chr1:161551100	77228	Whole Blood
chr1:161475219	60012	Whole Blood
chr1:161475219	53770	Whole Blood
chr1:161520526	381297	Whole Blood
chr1:161601752	18005	Whole Blood
chr1:161551100	129417	Whole Blood
chr1:161552879	100857	Whole Blood
chr1:161475219	295962	Whole Blood
chr1:161520526	80796	Whole Blood
chr1:161551100	106795	Whole Blood
chr1:161520526	113385	Whole Blood
chr1:161601752	60630	Whole Blood
chr1:161552879	60705	Whole Blood
chr1:161520526	141957	Whole Blood
chr1:161601752	307124	Whole Blood
chr1:161601752	4547	Whole Blood
chr1:161551100	50194	Whole Blood
chr1:161552879	104189	Whole Blood
chr1:161601752	16989	Whole Blood
chr1:161551100	314309	Whole Blood
chr1:161552879	385998	Whole Blood
chr1:161520526	205249	Whole Blood
chr1:161520526	52800	Whole Blood
chr1:161601752	178187	Whole Blood
chr1:161551100	68657	Whole Blood
chr1:161601752	325525	Whole Blood
chr1:161552879	16318	Whole Blood
chr1:161520526	129981	Whole Blood
chr1:161552879	398197	Whole Blood
chr1:161551100	109617	Whole Blood
chr1:161552879	105778	Whole Blood
chr1:161520526	66454	Whole Blood
chr1:161552879	67621	Whole Blood
chr1:161520526	250655	Whole Blood
chr1:161520526	54021	Whole Blood
chr1:161520526	68326	Whole Blood
chr1:161475219	137190	Whole Blood
chr1:161520526	75013	Whole Blood
chr1:161552879	50223	Whole Blood
chr1:161520526	87774	Whole Blood
chr1:161601752	56905	Whole Blood
chr1:161551100	397352	Whole Blood

chr1:161601752	299050	Whole Blood
chr1:161552879	15982	Whole Blood
chr1:161551100	13255	Whole Blood
chr1:161520526	28352	Whole Blood
chr1:161552879	62515	Whole Blood
chr1:161601752	3996	Whole Blood
chr1:161551100	46720	Whole Blood
chr1:161552879	44877	Whole Blood
chr1:161551100	52002	Whole Blood
chr1:161551100	38525	Whole Blood
chr1:161475219	70167	Whole Blood
chr1:161552879	55280	Whole Blood
chr1:161601752	11476	Whole Blood
chr1:161520526	201358	Whole Blood
chr1:161551100	43979	Whole Blood
chr1:161601752	263657	Whole Blood
chr1:161475219	15009	Whole Blood
chr1:161552879	31593	Whole Blood
chr1:161552879	105941	Whole Blood
chr1:161551100	71369	Whole Blood
chr1:161520526	46915	Whole Blood
chr1:161551100	64605	Whole Blood
chr1:161475219	132541	Whole Blood
chr1:161551100	37752	Whole Blood
chr1:161551100	8755	Whole Blood
chr1:161551100	240501	Whole Blood
chr1:161601752	274129	Whole Blood
chr1:161520526	143296	Whole Blood
chr1:161475219	110176	Whole Blood
chr1:161601752	33767	Whole Blood
chr1:161520526	123457	Whole Blood
chr1:161551100	181205	Whole Blood
chr1:161601752	196700	Whole Blood
chr1:161475219	172866	Whole Blood
chr1:161552879	96819	Whole Blood
chr1:161601752	36274	Whole Blood
chr1:161601752	196906	Whole Blood
chr1:161551100	121350	Whole Blood
chr1:161551100	156075	Whole Blood
chr1:161520526	77294	Whole Blood
chr1:161601752	326861	Whole Blood
chr1:161601752	27448	Whole Blood
chr1:161475219	121904	Whole Blood
chr1:161520526	216402	Whole Blood
chr1:161601752	29423	Whole Blood
chr1:161552879	272567	Whole Blood
chr1:161552879	37397	Whole Blood
chr1:161601752	70698	Whole Blood
chr1:161551100	50319	Whole Blood
chr1:161475219	100524	Whole Blood

chr1:161551100	16341	Whole Blood
chr1:161552879	47465	Whole Blood
chr1:161552879	153269	Whole Blood
chr1:161601752	153512	Whole Blood
chr1:161601752	93038	Whole Blood
chr1:161552879	32767	Whole Blood
chr1:161520526	387569	Whole Blood
chr1:161601752	177130	Whole Blood
chr1:161601752	328184	Whole Blood
chr1:161475219	415111	Whole Blood
chr1:161601752	158060	Whole Blood
chr1:161475219	65637	Whole Blood
chr1:161601752	3475	Whole Blood
chr1:161601752	311650	Whole Blood
chr1:161601752	205451	Whole Blood
chr1:161601752	236453	Whole Blood
chr1:161552879	49638	Whole Blood
chr1:161475219	175288	Whole Blood
chr1:161551100	15869	Whole Blood
chr1:161475219	316120	Whole Blood
chr1:161520526	250522	Whole Blood
chr1:161601752	340066	Whole Blood
chr1:161520526	147133	Whole Blood
chr1:161551100	23204	Whole Blood
chr1:161520526	270813	Whole Blood
chr1:161520526	77751	Whole Blood
chr1:161520526	53759	Whole Blood
chr1:161551100	573493	Whole Blood
chr1:161551100	67641	Whole Blood
chr1:161520526	142123	Whole Blood
chr1:161552879	123114	Whole Blood
chr1:161520526	202861	Whole Blood
chr1:161551100	47177	Whole Blood
chr1:161520526	45249	Whole Blood
chr1:161475219	110186	Whole Blood
chr1:161552879	106579	Whole Blood
chr1:161552879	120979	Whole Blood
chr1:161552879	312530	Whole Blood
chr1:161601752	70299	Whole Blood
chr1:161520526	182872	Whole Blood
chr1:161601752	152600	Whole Blood
chr1:161475219	144141	Whole Blood
chr1:161552879	24777	Whole Blood
chr1:161520526	69060	Whole Blood
chr1:161475219	133093	Whole Blood
chr1:161551100	6053	Whole Blood
chr1:161551100	21229	Whole Blood
chr1:161601752	12127	Whole Blood
chr1:161601752	197499	Whole Blood
chr1:161475219	172888	Whole Blood

chr1:161520526	247330	Whole Blood
chr1:161601752	765	Whole Blood
chr1:161601752	66521	Whole Blood
chr1:161552879	245779	Whole Blood
chr1:161552879	264282	Whole Blood
chr1:161601752	57068	Whole Blood
chr1:161601752	62696	Whole Blood
chr1:161475219	84953	Whole Blood
chr1:161601752	32410	Whole Blood
chr1:161551100	34546	Whole Blood
chr1:161475219	70667	Whole Blood
chr1:161475219	72696	Whole Blood
chr1:161520526	91702	Whole Blood
chr1:161551100	287105	Whole Blood
chr1:161601752	227149	Whole Blood
chr1:161520526	152880	Whole Blood
chr1:161552879	107326	Whole Blood
chr1:161551100	46656	Whole Blood
chr1:161520526	184546	Whole Blood
chr1:161475219	99434	Whole Blood
chr1:161475219	31527	Whole Blood
chr1:161552879	86385	Whole Blood
chr1:161520526	76737	Whole Blood
chr1:161475219	33495	Whole Blood
chr1:161552879	16463	Whole Blood
chr1:161601752	20717	Whole Blood
chr1:161520526	76650	Whole Blood
chr1:161520526	91706	Whole Blood
chr1:161551100	183454	Whole Blood
chr1:161520526	17319	Whole Blood
chr1:161552879	382072	Whole Blood
chr1:161551100	14343	Whole Blood
chr1:161552879	238722	Whole Blood
chr1:161601752	74241	Whole Blood
chr1:161551100	407089	Whole Blood
chr1:161601752	333576	Whole Blood
chr1:161552879	119571	Whole Blood
chr1:161551100	113348	Whole Blood
chr1:161520526	271209	Whole Blood
chr1:161520526	6452	Whole Blood
chr1:161475219	112672	Whole Blood
chr1:161520526	40707	Whole Blood
chr1:161552879	55432	Whole Blood
chr1:161520526	56926	Whole Blood
chr1:161552879	102704	Whole Blood
chr1:161552879	330832	Whole Blood
chr1:161520526	51803	Whole Blood
chr1:161552879	25901	Whole Blood
chr1:161552879	101963	Whole Blood
chr1:161475219	148121	Whole Blood

chr1:161601752	32891	Whole Blood
chr1:161551100	49244	Whole Blood
chr1:161551100	66631	Whole Blood
chr1:161601752	104396	Whole Blood
chr1:161475219	82308	Whole Blood
chr1:161475219	16955	Whole Blood
chr1:161601752	1408	Whole Blood
chr1:161601752	35709	Whole Blood
chr1:161520526	44952	Whole Blood
chr1:161551100	237664	Whole Blood
chr1:161551100	147794	Whole Blood
chr1:161475219	112877	Whole Blood
chr1:161475219	68078	Whole Blood
chr1:161475219	114762	Whole Blood
chr1:161601752	262213	Whole Blood
chr1:161552879	374398	Whole Blood
chr1:161475219	250799	Whole Blood
chr1:161551100	87225	Whole Blood
chr1:161520526	99562	Whole Blood
chr1:161551100	262503	Whole Blood
chr1:161520526	397302	Whole Blood
chr1:161552879	21410	Whole Blood
chr1:161520526	151525	Whole Blood
chr1:161552879	149573	Whole Blood
chr1:161475219	126301	Whole Blood
chr1:161601752	69074	Whole Blood
chr1:161475219	167852	Whole Blood
chr1:161520526	196415	Whole Blood
chr1:161551100	8187	Whole Blood
chr1:161475219	386290	Whole Blood
chr1:161551100	90283	Whole Blood
chr1:161601752	66711	Whole Blood
chr1:161552879	21425	Whole Blood
chr1:161475219	252707	Whole Blood
chr1:161520526	185344	Whole Blood
chr1:161475219	430204	Whole Blood
chr1:161552879	283680	Whole Blood
chr1:161475219	191481	Whole Blood
chr1:161551100	293625	Whole Blood
chr1:161601752	138422	Whole Blood
chr1:161520526	287413	Whole Blood
chr1:161520526	218195	Whole Blood
chr1:161552879	108609	Whole Blood
chr1:161551100	109108	Whole Blood
chr1:161520526	134316	Whole Blood
chr1:161551100	17761	Whole Blood
chr1:161552879	48415	Whole Blood
chr1:161601752	62830	Whole Blood
chr1:161475219	172313	Whole Blood
chr1:161520526	401234	Whole Blood

chr1:161551100	285459	Whole Blood
chr1:161601752	199229	Whole Blood
chr1:161552879	105016	Whole Blood
chr1:161475219	205212	Whole Blood
chr1:161601752	24096	Whole Blood
chr1:161551100	392846	Whole Blood
chr1:161520526	133027	Whole Blood
chr1:161552879	300240	Whole Blood
chr1:161601752	101938	Whole Blood
chr1:161601752	63661	Whole Blood
chr1:161475219	231407	Whole Blood
chr1:161551100	117363	Whole Blood
chr1:161601752	493	Whole Blood
chr1:161551100	114313	Whole Blood
chr1:161551100	256851	Whole Blood
chr1:161551100	102410	Whole Blood
chr1:161551100	180154	Whole Blood
chr1:161601752	16106	Whole Blood
chr1:161551100	10133	Whole Blood
chr1:161475219	168423	Whole Blood
chr1:161520526	82921	Whole Blood
chr1:161551100	100925	Whole Blood
chr1:161551100	329053	Whole Blood
chr1:161552879	87460	Whole Blood
chr1:161520526	265179	Whole Blood
chr1:161552879	255072	Whole Blood
chr1:161475219	229253	Whole Blood
chr1:161551100	98382	Whole Blood
chr1:161475219	1347	Whole Blood
chr1:161552879	227060	Whole Blood
chr1:161552879	68810	Whole Blood
chr1:161475219	147537	Whole Blood
chr1:161475219	104973	Whole Blood
chr1:161551100	9978	Whole Blood
chr1:161601752	129502	Whole Blood
chr1:161601752	63907	Whole Blood
chr1:161552879	226003	Whole Blood
chr1:161552879	65862	Whole Blood
chr1:161552879	377057	Whole Blood
chr1:161520526	78371	Whole Blood
chr1:161551100	152559	Whole Blood
chr1:161551100	18097	Whole Blood
chr1:161551100	122758	Whole Blood
chr1:161552879	357867	Whole Blood
chr1:161601752	53090	Whole Blood
chr1:161475219	249083	Whole Blood
chr1:161551100	152590	Whole Blood
chr1:161520526	52774	Whole Blood
chr1:161601752	27463	Whole Blood
chr1:161552879	359849	Whole Blood

chr1:161601752	346700	Whole Blood
chr1:161552879	150811	Whole Blood
chr1:161552879	107838	Whole Blood
chr1:161551100	299053	Whole Blood
chr1:161551100	22026	Whole Blood
chr1:161520526	80893	Whole Blood
chr1:161601752	56143	Whole Blood
chr1:161552879	16006	Whole Blood
chr1:161601752	135258	Whole Blood
chr1:161520526	153543	Whole Blood
chr1:161520526	69099	Whole Blood
chr1:161552879	121052	Whole Blood
chr1:161520526	345361	Whole Blood
chr1:161475219	127115	Whole Blood
chr1:161520526	51826	Whole Blood
chr1:161601752	308994	Whole Blood
chr1:161475219	90556	Whole Blood
chr1:161520526	63946	Whole Blood
chr1:161520526	129462	Whole Blood
chr1:161520526	101943	Whole Blood
chr1:161601752	153062	Whole Blood
chr1:161601752	349324	Whole Blood
chr1:161520526	185622	Whole Blood
chr1:161551100	389991	Whole Blood
chr1:161475219	114733	Whole Blood
chr1:161520526	409410	Whole Blood
chr1:161551100	58926	Whole Blood
chr1:161551100	85681	Whole Blood
chr1:161520526	48503	Whole Blood
chr1:161601752	339287	Whole Blood
chr1:161551100	17785	Whole Blood
chr1:161475219	10039	Whole Blood
chr1:161475219	161783	Whole Blood
chr1:161601752	32867	Whole Blood
chr1:161601752	32555	Whole Blood
chr1:161520526	20596	Whole Blood
chr1:161601752	109578	Whole Blood
chr1:161520526	138131	Whole Blood
chr1:161601752	72106	Whole Blood
chr1:161601752	39631	Whole Blood
chr1:161520526	53763	Whole Blood
chr1:161520526	208913	Whole Blood
chr1:161520526	115976	Whole Blood
chr1:161551100	148842	Whole Blood
chr1:161520526	57130	Whole Blood
chr1:161551100	637697	Whole Blood
chr1:161520526	118758	Whole Blood
chr1:161520526	70351	Whole Blood
chr1:161520526	99231	Whole Blood
chr1:161601752	151655	Whole Blood

chr1:161551100	181527	Whole Blood
chr1:161520526	231929	Whole Blood
chr1:161520526	145923	Whole Blood
chr1:161601752	310976	Whole Blood
chr1:161475219	204792	Whole Blood
chr1:161520526	231933	Whole Blood
chr1:161520526	415960	Whole Blood
chr1:161551100	65842	Whole Blood
chr1:161601752	130875	Whole Blood
chr1:161475219	78918	Whole Blood
chr1:161601752	243780	Whole Blood
chr1:161520526	48816	Whole Blood
chr1:161551100	18242	Whole Blood
chr1:161520526	231086	Whole Blood
chr1:161520526	262764	Whole Blood
chr1:161475219	316516	Whole Blood
chr1:161475219	142600	Whole Blood
chr1:161551100	103565	Whole Blood
chr1:161552879	388306	Whole Blood
chr1:161551100	84606	Whole Blood
chr1:161552879	194653	Whole Blood
chr1:161552879	89004	Whole Blood
chr1:161520526	81991	Whole Blood
chr1:161551100	211525	Whole Blood
chr1:161475219	128228	Whole Blood
chr1:161520526	65120	Whole Blood
chr1:161475219	186626	Whole Blood
chr1:161551100	105736	Whole Blood
chr1:161552879	178375	Whole Blood
chr1:161552879	334768	Whole Blood
chr1:161601752	58456	Whole Blood
chr1:161520526	304920	Whole Blood
chr1:161475219	168443	Whole Blood
chr1:161475219	25122	Whole Blood
chr1:161552879	36366	Whole Blood
chr1:161552879	150621	Whole Blood
chr1:161520526	138294	Whole Blood
chr1:161551100	68262	Whole Blood
chr1:161520526	94868	Whole Blood
chr1:161552879	424727	Whole Blood
chr1:161520526	54032	Whole Blood
chr1:161601752	66984	Whole Blood
chr1:161520526	47459	Whole Blood
chr1:161551100	389570	Whole Blood
chr1:161551100	38145	Whole Blood
chr1:161601752	138152	Whole Blood
chr1:161520526	46509	Whole Blood
chr1:161520526	238231	Whole Blood
chr1:161552879	48380	Whole Blood
chr1:161520526	87785	Whole Blood

chr1:161520526	388350	Whole Blood
chr1:161475219	67694	Whole Blood
chr1:161475219	248168	Whole Blood
chr1:161520526	76596	Whole Blood
chr1:161520526	87633	Whole Blood
chr1:161552879	107329	Whole Blood
chr1:161601752	342537	Whole Blood
chr1:161520526	130011	Whole Blood
chr1:161552879	36447	Whole Blood
chr1:161520526	68719	Whole Blood
chr1:161601752	137877	Whole Blood
chr1:161552879	291846	Whole Blood
chr1:161551100	38226	Whole Blood
chr1:161601752	12507	Whole Blood
chr1:161552879	347923	Whole Blood
chr1:161475219	99929	Whole Blood
chr1:161520526	399451	Whole Blood
chr1:161475219	114439	Whole Blood
chr1:161552879	115584	Whole Blood
chr1:161520526	77230	Whole Blood
chr1:161520526	74553	Whole Blood
chr1:161520526	157727	Whole Blood
chr1:161601752	101907	Whole Blood
chr1:161601752	321118	Whole Blood
chr1:161475219	183868	Whole Blood
chr1:161551100	67031	Whole Blood
chr1:161520526	311049	Whole Blood
chr1:161520526	80305	Whole Blood
chr1:161601752	339339	Whole Blood
chr1:161551100	192874	Whole Blood
chr1:161601752	72179	Whole Blood
chr1:161551100	117636	Whole Blood
chr1:161520526	239892	Whole Blood
chr1:161520526	344883	Whole Blood
chr1:161601752	117683	Whole Blood
chr1:161520526	128956	Whole Blood
chr1:161475219	126103	Whole Blood
chr1:161551100	114274	Whole Blood
chr1:161520526	76679	Whole Blood
chr1:161520526	56651	Whole Blood
chr1:161520526	94843	Whole Blood
chr1:161520526	79818	Whole Blood
chr1:161475219	67701	Whole Blood
chr1:161475219	296592	Whole Blood
chr1:161551100	24122	Whole Blood
chr1:161601752	206727	Whole Blood
chr1:161520526	367121	Whole Blood
chr1:161601752	339433	Whole Blood
chr1:161520526	146106	Whole Blood
chr1:161552879	179748	Whole Blood

chr1:161520526	140962	Whole Blood
chr1:161552879	639476	Whole Blood
chr1:161520526	36627	Whole Blood
chr1:161520526	48335	Whole Blood
chr1:161520526	348717	Whole Blood
chr1:161551100	119726	Whole Blood
chr1:161520526	155363	Whole Blood
chr1:161475219	114548	Whole Blood
chr1:161475219	87855	Whole Blood
chr1:161520526	52600	Whole Blood
chr1:161601752	197200	Whole Blood
chr1:161475219	8850	Whole Blood
chr1:161552879	382449	Whole Blood
chr1:161552879	395573	Whole Blood
chr1:161475219	205566	Whole Blood
chr1:161475219	61538	Whole Blood
chr1:161520526	48671	Whole Blood
chr1:161520526	82576	Whole Blood
chr1:161520526	151924	Whole Blood
chr1:161520526	153332	Whole Blood
chr1:161520526	129172	Whole Blood
chr1:161520526	287425	Whole Blood
chr1:161601752	151577	Whole Blood
chr1:161520526	140191	Whole Blood
chr1:161520526	242499	Whole Blood
chr1:161552879	89293	Whole Blood
chr1:161601752	116494	Whole Blood
chr1:161520526	155467	Whole Blood
chr1:161475219	171095	Whole Blood
chr1:161552879	107515	Whole Blood
chr1:161552879	150780	Whole Blood
chr1:161475219	168764	Whole Blood
chr1:161552879	111569	Whole Blood
chr1:161601752	12426	Whole Blood
chr1:161601752	251367	Whole Blood
chr1:161520526	317679	Whole Blood
chr1:161601752	63622	Whole Blood
chr1:161520526	72991	Whole Blood
chr1:161601752	74774	Whole Blood
chr1:161475219	159942	Whole Blood
chr1:161475219	432876	Whole Blood
chr1:161475219	295829	Whole Blood
chr1:161475219	194585	Whole Blood
chr1:161551100	426136	Whole Blood
chr1:161520526	414802	Whole Blood
chr1:161552879	88504	Whole Blood
chr1:161601752	242973	Whole Blood
chr1:161475219	127298	Whole Blood
chr1:161552879	66483	Whole Blood
chr1:161601752	69553	Whole Blood

chr1:161520526	159991	Whole Blood
chr1:161551100	49088	Whole Blood
chr1:161551100	87514	Whole Blood
chr1:161475219	416385	Whole Blood
chr1:161520526	145595	Whole Blood
chr1:161601752	58048	Whole Blood
chr1:161601752	376492	Whole Blood
chr1:161551100	349050	Whole Blood
chr1:161552879	47309	Whole Blood
chr1:161520526	80768	Whole Blood
chr1:161520526	138932	Whole Blood
chr1:161520526	95179	Whole Blood
chr1:161551100	291983	Whole Blood
chr1:161520526	97205	Whole Blood
chr1:161601752	136333	Whole Blood
chr1:161520526	278132	Whole Blood
chr1:161475219	439870	Whole Blood
chr1:161552879	115857	Whole Blood
chr1:161601752	339635	Whole Blood
chr1:161601752	197202	Whole Blood
chr1:161520526	325006	Whole Blood
chr1:161601752	375484	Whole Blood
chr1:161520526	98215	Whole Blood
chr1:161520526	140884	Whole Blood
chr1:161520526	68800	Whole Blood
chr1:161475219	122767	Whole Blood
chr1:161601752	198446	Whole Blood
chr1:161475219	51759	Whole Blood
chr1:161601752	337125	Whole Blood
chr1:161475219	11344	Whole Blood
chr1:161601752	130553	Whole Blood
chr1:161520526	139679	Whole Blood
chr1:161475219	94349	Whole Blood
chr1:161520526	278182	Whole Blood
chr1:161552879	117947	Whole Blood
chr1:161520526	355355	Whole Blood
chr1:161475219	186785	Whole Blood
chr1:161552879	405310	Whole Blood
chr1:161475219	156051	Whole Blood
chr1:161551100	26103	Whole Blood
chr1:161475219	126041	Whole Blood
chr1:161520526	281894	Whole Blood
chr1:161475219	122601	Whole Blood
chr1:161475219	247585	Whole Blood
chr1:161520526	330398	Whole Blood
chr1:161520526	150300	Whole Blood
chr1:161552879	112534	Whole Blood
chr1:161520526	183133	Whole Blood
chr1:161520526	80733	Whole Blood
chr1:161520526	120857	Whole Blood

chr1:161475219	173402	Whole Blood
chr1:161520526	36457	Whole Blood
chr1:161520526	210728	Whole Blood
chr1:161475219	125372	Whole Blood
chr1:161601752	356437	Whole Blood
chr1:161475219	8725	Whole Blood
chr1:161520526	343439	Whole Blood
chr1:161475219	48104	Whole Blood
chr1:161475219	100616	Whole Blood
chr1:161520526	137369	Whole Blood
chr1:161551100	108700	Whole Blood
chr1:161551100	348535	Whole Blood
chr1:161552879	388508	Whole Blood
chr1:161520526	48359	Whole Blood
chr1:161552879	111703	Whole Blood
chr1:161520526	153405	Whole Blood
chr1:161601752	234106	Whole Blood
chr1:161520526	260174	Whole Blood
chr1:161520526	133210	Whole Blood
chr1:161601752	375854	Whole Blood
chr1:161601752	624145	Whole Blood
chr1:161601752	338918	Whole Blood
chr1:161475219	250014	Whole Blood
chr1:161475219	326336	Whole Blood
chr1:161520526	275895	Whole Blood
chr1:161552879	297274	Whole Blood
chr1:161520526	115974	Whole Blood
chr1:161475219	70669	Whole Blood
chr1:161601752	156388	Whole Blood
chr1:161520526	98018	Whole Blood
chr1:161475219	185833	Whole Blood
chr1:161551100	328815	Whole Blood
chr1:161475219	95137	Whole Blood
chr1:161520526	457718	Whole Blood
chr1:161552879	347271	Whole Blood
chr1:161520526	420513	Whole Blood
chr1:161520526	430550	Whole Blood
chr1:161520526	392940	Whole Blood
chr1:161520526	56677	Whole Blood
chr1:161552879	152960	Whole Blood
chr1:161520526	117220	Whole Blood
chr1:161475219	122044	Whole Blood
chr1:161475219	375705	Whole Blood
chr1:161552879	290204	Whole Blood
chr1:161520526	147937	Whole Blood
chr1:161520526	392202	Whole Blood
chr1:161520526	258356	Whole Blood
chr1:161475219	332720	Whole Blood
chr1:161552879	285326	Whole Blood
chr1:161601752	278163	Whole Blood

chr1:161520526	71836	Whole Blood
chr1:161475219	100388	Whole Blood
chr1:161520526	421292	Whole Blood
chr1:161520526	261311	Whole Blood
chr1:161520526	390220	Whole Blood
chr1:161601752	297883	Whole Blood
chr1:161601752	17610	Whole Blood
chr1:161551100	120205	Whole Blood
chr1:161475219	203091	Whole Blood
chr1:161475219	287806	Whole Blood
chr1:161552879	105344	Whole Blood
chr1:161475219	205571	Whole Blood
chr1:161552879	424357	Whole Blood
chr1:161475219	145212	Whole Blood
chr1:161520526	212101	Whole Blood
chr1:161551100	298395	Whole Blood
chr1:161551100	151181	Whole Blood
chr1:161551100	300809	Whole Blood
chr1:161475219	276645	Whole Blood
chr1:161475219	203157	Whole Blood
chr1:161520526	56940	Whole Blood
chr1:161475219	167905	Whole Blood
chr1:161551100	300685	Whole Blood
chr1:161475219	256899	Whole Blood
chr1:161520526	414425	Whole Blood
chr1:161601752	248401	Whole Blood
chr1:161475219	426604	Whole Blood
chr1:161475219	169167	Whole Blood
chr1:161475219	186440	Whole Blood
chr1:161475219	278286	Whole Blood
chr1:161520526	420659	Whole Blood
chr1:161520526	53778	Whole Blood
chr1:161520526	98836	Whole Blood
chr1:161520526	406751	Whole Blood
chr1:161520526	69750	Whole Blood
chr1:161601752	250157	Whole Blood
chr1:161475219	179728	Whole Blood
chr1:161475219	25044	Whole Blood
chr1:161475219	62626	Whole Blood
chr1:161520526	269465	Whole Blood
chr1:161475219	125412	Whole Blood
chr1:161520526	259413	Whole Blood
chr1:161520526	144056	Whole Blood
chr1:161520526	437663	Whole Blood
chr1:161475219	202962	Whole Blood
chr1:161475219	123058	Whole Blood
chr1:161475219	248172	Whole Blood
chr1:161601752	298398	Whole Blood
chr1:161520526	361662	Whole Blood
chr1:161520526	270020	Whole Blood

chr1:161601752	379705	Whole Blood
chr1:161552879	24324	Whole Blood
chr1:161520526	271075	Whole Blood
chr1:161601752	1564	Whole Blood
chr1:161520526	380276	Whole Blood
chr1:161475219	209670	Whole Blood
chr1:161475219	67126	Whole Blood
chr1:161552879	391067	Whole Blood
chr1:161475219	122537	Whole Blood
chr1:161475219	86014	Whole Blood
chr1:161520526	148210	Whole Blood
chr1:161520526	340983	Whole Blood
chr1:161601752	211237	Whole Blood
chr1:161520526	379624	Whole Blood
chr1:161601752	241331	Whole Blood
chr1:161520526	147131	Whole Blood
chr1:161475219	26529	Whole Blood
chr1:161475219	99328	Whole Blood
chr1:161475219	301723	Whole Blood
chr1:161475219	97133	Whole Blood
chr1:161475219	97907	Whole Blood
chr1:161601752	262177	Whole Blood
chr1:161520526	139682	Whole Blood
chr1:161551100	682753	Whole Blood
chr1:161552879	179426	Whole Blood
chr1:161551100	26568	Whole Blood
chr1:161475219	99066	Whole Blood
chr1:161520526	125501	Whole Blood
chr1:161601752	342194	Whole Blood
chr1:161601752	154217	Whole Blood
chr1:161475219	98107	Whole Blood
chr1:161475219	109253	Whole Blood
chr1:161520526	211785	Whole Blood
chr1:161601752	24549	Whole Blood
chr1:161475219	92222	Whole Blood
chr1:161520526	55107	Whole Blood
chr1:161475219	114367	Whole Blood
chr1:161475219	314772	Whole Blood
chr1:161475219	189545	Whole Blood
chr1:161552879	388212	Whole Blood
chr1:161601752	247743	Whole Blood
chr1:161552879	387791	Whole Blood
chr1:161520526	118268	Whole Blood
chr1:161475219	114406	Whole Blood
chr1:161475219	447308	Whole Blood
chr1:161475219	110427	Whole Blood
chr1:161520526	420144	Whole Blood
chr1:161520526	183164	Whole Blood
chr1:161552879	118426	Whole Blood
chr1:161475219	93978	Whole Blood

chr1:161475219	255754	Whole Blood
chr1:161475219	65903	Whole Blood
chr1:161520526	420861	Whole Blood
chr1:161520526	75162	Whole Blood
chr1:161601752	138166	Whole Blood
chr1:161475219	216004	Whole Blood
chr1:161475219	107573	Whole Blood
chr1:161475219	98081	Whole Blood
chr1:161475219	230651	Whole Blood
chr1:161552879	296616	Whole Blood
chr1:161475219	94123	Whole Blood
chr1:161475219	27684	Whole Blood
chr1:161475219	9800	Whole Blood
chr1:161552879	346756	Whole Blood
chr1:161601752	199494	Whole Blood
chr1:161475219	100548	Whole Blood
chr1:161475219	186622	Whole Blood
chr1:161520526	316033	Whole Blood
chr1:161475219	390668	Whole Blood
chr1:161520526	180951	Whole Blood
chr1:161475219	97110	Whole Blood
chr1:161520526	392876	Whole Blood
chr1:161520526	79662	Whole Blood
chr1:161475219	184625	Whole Blood
chr1:161475219	133092	Whole Blood
chr1:161475219	111761	Whole Blood
chr1:161475219	91816	Whole Blood
chr1:161552879	106921	Whole Blood
chr1:161520526	315100	Whole Blood
chr1:161552879	299030	Whole Blood
chr1:161520526	418351	Whole Blood
chr1:161601752	201833	Whole Blood
chr1:161475219	90259	Whole Blood
chr1:161520526	143922	Whole Blood
chr1:161475219	133081	Whole Blood
chr1:161520526	351265	Whole Blood
chr1:161552879	112495	Whole Blood
chr1:161475219	90824	Whole Blood
chr1:161520526	324199	Whole Blood
chr1:161520526	416091	Whole Blood
chr1:161475219	120510	Whole Blood
chr1:161520526	211779	Whole Blood
chr1:161520526	120607	Whole Blood
chr1:161475219	92766	Whole Blood
chr1:161475219	211506	Whole Blood
chr1:161475219	11633	Whole Blood
chr1:161475219	180296	Whole Blood
chr1:161475219	183601	Whole Blood
chr1:161552879	24789	Whole Blood
chr1:161475219	102437	Whole Blood

chr1:161520526	144887	Whole Blood
chr1:161475219	99070	Whole Blood
chr1:161552879	327036	Whole Blood
chr1:161475219	394024	Whole Blood
chr1:161475219	71913	Whole Blood
chr1:161520526	359389	Whole Blood
chr1:161475219	254480	Whole Blood
chr1:161475219	135804	Whole Blood
chr1:161475219	229853	Whole Blood
chr1:161601752	310525	Whole Blood
chr1:161475219	461398	Whole Blood
chr1:161475219	232282	Whole Blood
chr1:161601752	243526	Whole Blood
chr1:161520526	322557	Whole Blood
chr1:161475219	370313	Whole Blood
chr1:161475219	187430	Whole Blood
chr1:161475219	192440	Whole Blood
chr1:161475219	80194	Whole Blood
chr1:161475219	185175	Whole Blood
chr1:161475219	137013	Whole Blood
chr1:161475219	29855	Whole Blood
chr1:161475219	321202	Whole Blood
chr1:161475219	72961	Whole Blood
chr1:161552879	684532	Whole Blood
chr1:161475219	166164	Whole Blood
chr1:161520526	427926	Whole Blood
chr1:161475219	188115	Whole Blood
chr1:161475219	119860	Whole Blood
chr1:161601752	80095	Whole Blood
chr1:161475219	325216	Whole Blood
chr1:161475219	315327	Whole Blood
chr1:161520526	57142	Whole Blood
chr1:161520526	162300	Whole Blood
chr1:161475219	183992	Whole Blood
chr1:161475219	174769	Whole Blood
chr1:161520526	420565	Whole Blood
chr1:161475219	125125	Whole Blood
chr1:161520526	328969	Whole Blood
chr1:161551100	259873	Whole Blood
chr1:161475219	143522	Whole Blood
chr1:161520526	150779	Whole Blood
chr1:161475219	93810	Whole Blood
chr1:161475219	230929	Whole Blood
chr1:161520526	139274	Whole Blood
chr1:161475219	137009	Whole Blood
chr1:161475219	433657	Whole Blood
chr1:161475219	228772	Whole Blood
chr1:161475219	144538	Whole Blood
chr1:161475219	140486	Whole Blood
chr1:161475219	178334	Whole Blood

chr1:161475219	175844	Whole Blood
chr1:161475219	228179	Whole Blood
chr1:161551100	130747	Whole Blood
chr1:161475219	93666	Whole Blood
chr1:161520526	332593	Whole Blood
chr1:161475219	308441	Whole Blood
chr1:161475219	310486	Whole Blood
chr1:161520526	298479	Whole Blood
chr1:161601752	274714	Whole Blood
chr1:161520526	329627	Whole Blood
chr1:161475219	227164	Whole Blood
chr1:161520526	423420	Whole Blood
chr1:161475219	198712	Whole Blood
chr1:161520526	331383	Whole Blood
chr1:161475219	144808	Whole Blood
chr1:161475219	198850	Whole Blood
chr1:161475219	81934	Whole Blood
chr1:161601752	676612	Whole Blood
chr1:161520526	144848	Whole Blood
chr1:161475219	186269	Whole Blood
chr1:161475219	121903	Whole Blood
chr1:161520526	331259	Whole Blood
chr1:161475219	316382	Whole Blood
chr1:161475219	453394	Whole Blood
chr1:161475219	406969	Whole Blood
chr1:161475219	101984	Whole Blood
chr1:161475219	147250	Whole Blood
chr1:161475219	174263	Whole Blood
chr1:161601752	250033	Whole Blood
chr1:161475219	307055	Whole Blood
chr1:161475219	140175	Whole Blood
chr1:161601752	24084	Whole Blood
chr1:161475219	205298	Whole Blood
chr1:161520526	229299	Whole Blood
chr1:161475219	203034	Whole Blood
chr1:161475219	144869	Whole Blood
chr1:161475219	121957	Whole Blood
chr1:161475219	84704	Whole Blood
chr1:161475219	93642	Whole Blood
chr1:161475219	196832	Whole Blood
chr1:161475219	115057	Whole Blood
chr1:161475219	388746	Whole Blood
chr1:161475219	114107	Whole Blood
chr1:161475219	125056	Whole Blood
chr1:161475219	125612	Whole Blood
chr1:161475219	444758	Whole Blood
chr1:161475219	182676	Whole Blood
chr1:161475219	75300	Whole Blood
chr1:161475219	503025	Whole Blood
chr1:161551100	325366	Whole Blood

chr1:161552879	298906	Whole Blood
chr1:161475219	187264	Whole Blood
chr1:161475219	452058	Whole Blood
chr1:161552879	261652	Whole Blood
chr1:161552879	128968	Whole Blood
chr1:161475219	127883	Whole Blood
chr1:161475219	190902	Whole Blood
chr1:161520526	161321	Whole Blood
chr1:161475219	140150	Whole Blood
chr1:161475219	174479	Whole Blood
chr1:161520526	456710	Whole Blood
chr1:161475219	323489	Whole Blood
chr1:161475219	126075	Whole Blood
chr1:161475219	184239	Whole Blood
chr1:161475219	143325	Whole Blood
chr1:161475219	308071	Whole Blood
chr1:161475219	361340	Whole Blood
chr1:161601752	688349	Whole Blood
chr1:161475219	200670	Whole Blood
chr1:161520526	408087	Whole Blood
chr1:161475219	113633	Whole Blood
chr1:161475219	186191	Whole Blood
chr1:161475219	132940	Whole Blood
chr1:161475219	283538	Whole Blood
chr1:161475219	99085	Whole Blood
chr1:161520526	542919	Whole Blood
chr1:161475219	191413	Whole Blood
chr1:161475219	356356	Whole Blood
chr1:161475219	114026	Whole Blood
chr1:161475219	142512	Whole Blood
chr1:161475219	185498	Whole Blood
chr1:161475219	465820	Whole Blood
chr1:161475219	179623	Whole Blood
chr1:161475219	121986	Whole Blood
chr1:161475219	254220	Whole Blood
chr1:161475219	256035	Whole Blood
chr1:161475219	126200	Whole Blood
chr1:161475219	184989	Whole Blood
chr1:161475219	189363	Whole Blood
chr1:161520526	457080	Whole Blood
chr1:161475219	412428	Whole Blood
chr1:161475219	183438	Whole Blood
chr1:161475219	325767	Whole Blood
chr1:161520526	379109	Whole Blood
chr1:161475219	102449	Whole Blood
chr1:161475219	116993	Whole Blood
chr1:161475219	475857	Whole Blood
chr1:161475219	241722	Whole Blood
chr1:161475219	323439	Whole Blood
chr1:161475219	144143	Whole Blood

chr1:161475219	164065	Whole Blood
chr1:161475219	438183	Whole Blood
chr1:161475219	178517	Whole Blood
chr1:161475219	461267	Whole Blood
chr1:161475219	442609	Whole Blood
chr1:161475219	124969	Whole Blood
chr1:161552879	323587	Whole Blood
chr1:161475219	198639	Whole Blood
chr1:161475219	332732	Whole Blood
chr1:161475219	257092	Whole Blood
chr1:161475219	396572	Whole Blood
chr1:161475219	454717	Whole Blood
chr1:161475219	305481	Whole Blood
chr1:161475219	438247	Whole Blood
chr1:161475219	459732	Whole Blood
chr1:161475219	123678	Whole Blood
chr1:161520526	355940	Whole Blood
chr1:161475219	390190	Whole Blood
chr1:161475219	497612	Whole Blood
chr1:161475219	193517	Whole Blood
chr1:161475219	126040	Whole Blood
chr1:161475219	192438	Whole Blood
chr1:161475219	257408	Whole Blood
chr1:161475219	188603	Whole Blood
chr1:161475219	435527	Whole Blood
chr1:161475219	466599	Whole Blood
chr1:161475219	195607	Whole Blood
chr1:161475219	327201	Whole Blood
chr1:161475219	400662	Whole Blood
chr1:161475219	184986	Whole Blood
chr1:161520526	595386	Whole Blood
chr1:161475219	360407	Whole Blood
chr1:161475219	377900	Whole Blood
chr1:161475219	228471	Whole Blood
chr1:161475219	369506	Whole Blood
chr1:161475219	425583	Whole Blood
chr1:161475219	200774	Whole Blood
chr1:161475219	193244	Whole Blood
chr1:161520526	652179	Whole Blood
chr1:161601752	733405	Whole Blood
chr1:161475219	304720	Whole Blood
chr1:161475219	350227	Whole Blood
chr1:161475219	463658	Whole Blood
chr1:161475219	424931	Whole Blood
chr1:161475219	135644	Whole Blood
chr1:161475219	228440	Whole Blood
chr1:161475219	362986	Whole Blood
chr1:161475219	374276	Whole Blood
chr1:161475219	446541	Whole Blood
chr1:161475219	482970	Whole Blood

chr1:161475219	197231	Whole Blood
chr1:161475219	189229	Whole Blood
chr1:161475219	367864	Whole Blood
chr1:161475219	196086	Whole Blood
chr1:161475219	253172	Whole Blood
chr1:161475219	190194	Whole Blood
chr1:161475219	550079	Whole Blood
chr1:161520526	607123	Whole Blood
chr1:161475219	460109	Whole Blood
chr1:161475219	206628	Whole Blood
chr1:161475219	404696	Whole Blood
chr1:161475219	257086	Whole Blood
chr1:161475219	468727	Whole Blood
chr1:161475219	437509	Whole Blood
chr1:161475219	502017	Whole Blood
chr1:161475219	303663	Whole Blood
chr1:161475219	465451	Whole Blood
chr1:161475219	374934	Whole Blood
chr1:161475219	184581	Whole Blood
chr1:161475219	473233	Whole Blood
chr1:161475219	190155	Whole Blood
chr1:161475219	465966	Whole Blood
chr1:161475219	465872	Whole Blood
chr1:161475219	424416	Whole Blood
chr1:161475219	502387	Whole Blood
chr1:161475219	376690	Whole Blood
chr1:161475219	606872	Whole Blood
chr1:161475219	376566	Whole Blood
chr1:161475219	466168	Whole Blood
chr1:161475219	401247	Whole Blood
chr1:161475219	561816	Whole Blood

Score
0,8968
0,88821
0,87185
0,86432
0,85288
0,85067
0,84701
0,84625
0,84602
0,84503
0,84172
0,84158
0,83382
0,83335
0,83198
0,83191
0,82899
0,82888
0,82848
0,82707
0,82406
0,82399
0,81765
0,81588
0,81282
0,80378
0,80169
0,79963
0,79912
0,7964
0,79284
0,78667
0,78402
0,78334
0,77923
0,77718
0,77598
0,77567
0,77542
0,77535
0,77337
0,77187
0,77021
0,76918
0,76793
0,76763
0,76736
0,76554
0,76456

0,76315
0,76171
0,75872
0,75558
0,75402
0,7532
0,75244
0,75198
0,75079
0,75009
0,74752
0,74154
0,73976
0,73929
0,73916
0,73334
0,73237
0,73205
0,73007
0,72989
0,72965
0,72868
0,72723
0,72687
0,72392
0,71495
0,71412
0,71395
0,70999
0,70774
0,70669
0,70541
0,70479
0,70214
0,70211
0,69921
0,6976
0,69731
0,69597
0,69457
0,69296
0,69029
0,68994
0,68261
0,68134
0,68028
0,67691
0,67121
0,67048
0,6698

0,66734
0,66597
0,66465
0,6643
0,66316
0,65842
0,65718
0,65418
0,65388
0,65388
0,65117
0,65032
0,64737
0,64334
0,64142
0,64064
0,63847
0,63644
0,6357
0,63481
0,6341
0,63267
0,63243
0,63124
0,63072
0,63035
0,62747
0,62648
0,62467
0,62292
0,6229
0,62205
0,6212
0,6209
0,61557
0,61524
0,61434
0,61372
0,61166
0,61037
0,60929
0,60568
0,60442
0,60426
0,60347
0,60263
0,60139
0,60118
0,60019
0,59949

0,59824
0,59764
0,59731
0,59646
0,594
0,59355
0,59349
0,5927
0,58831
0,58803
0,58371
0,58357
0,58253
0,5814
0,58137
0,58114
0,58003
0,57996
0,57932
0,57771
0,57636
0,57394
0,57367
0,57028
0,56967
0,56673
0,5657
0,56547
0,56206
0,56181
0,56139
0,56036
0,55886
0,55822
0,55782
0,55728
0,55594
0,55532
0,55497
0,55479
0,55187
0,55137
0,55117
0,54912
0,54688
0,54477
0,54467
0,54363
0,54148
0,54063

0,54055
0,53886
0,53725
0,5366
0,53649
0,53641
0,53639
0,53535
0,53415
0,5339
0,53377
0,53129
0,52589
0,52551
0,52298
0,52263
0,52251
0,52151
0,52132
0,51834
0,51552
0,51539
0,51508
0,51445
0,51332
0,51211
0,50637
0,50507
0,50449
0,50186
0,50132
0,50104
0,50009
0,49972
0,49943
0,49886
0,49864
0,49748
0,49714
0,49578
0,49518
0,49473
0,49464
0,49414
0,49352
0,49293
0,49222
0,49209
0,49199
0,49052

0,4891
0,48761
0,48748
0,48724
0,48592
0,48585
0,48574
0,48514
0,48437
0,48393
0,48275
0,48148
0,48122
0,48108
0,47701
0,4759
0,47536
0,47519
0,47515
0,47479
0,47424
0,47387
0,47369
0,47345
0,47247
0,47146
0,46979
0,46677
0,46552
0,46543
0,46502
0,46486
0,45905
0,45901
0,45845
0,4583
0,45827
0,45805
0,45717
0,45688
0,45616
0,45468
0,45417
0,45406
0,45382
0,45368
0,453
0,45296
0,45258
0,45207

0,45128
0,4494
0,44841
0,44669
0,44648
0,44596
0,44546
0,44479
0,44438
0,44399
0,44383
0,44381
0,44268
0,4426
0,44075
0,44037
0,4402
0,43949
0,43893
0,43797
0,43697
0,4362
0,43618
0,4357
0,43557
0,43551
0,43374
0,43365
0,43273
0,43139
0,42903
0,42902
0,42783
0,42721
0,42699
0,42696
0,42671
0,42666
0,42604
0,42545
0,4253
0,42453
0,42251
0,42229
0,42218
0,42125
0,42124
0,41855
0,4176
0,41587

0,41576
0,4153
0,41475
0,4136
0,4128
0,4128
0,41258
0,41145
0,41068
0,40939
0,40905
0,40823
0,40744
0,4068
0,40633
0,40612
0,40509
0,40419
0,40384
0,40354
0,40294
0,40281
0,40096
0,40076
0,40027
0,40002
0,39917
0,39871
0,39809
0,39756
0,397
0,3961
0,3961
0,39561
0,39505
0,39457
0,39392
0,39356
0,39347
0,39341
0,39338
0,3933
0,39326
0,39246
0,39089
0,39061
0,39045
0,38879
0,38852
0,38827

0,387
0,38659
0,38585
0,38546
0,38437
0,3829
0,38206
0,38172
0,38109
0,38105
0,38075
0,38038
0,3802
0,37944
0,37907
0,37794
0,37784
0,37783
0,37741
0,37686
0,37638
0,37613
0,37592
0,37582
0,37531
0,37465
0,37365
0,37336
0,37314
0,37301
0,37256
0,37129
0,37046
0,36989
0,36778
0,36777
0,36709
0,36672
0,36628
0,36548
0,36514
0,36461
0,36444
0,36326
0,3627
0,36245
0,36234
0,36228
0,36203
0,36121

0,36033
0,36026
0,35865
0,35858
0,35763
0,35753
0,35737
0,3569
0,35628
0,35625
0,35552
0,35447
0,35403
0,35394
0,35387
0,35379
0,35373
0,3534
0,35326
0,35319
0,35128
0,35128
0,34992
0,34989
0,34779
0,34729
0,34654
0,34638
0,3455
0,34427
0,34399
0,34364
0,34343
0,33934
0,33791
0,33766
0,33684
0,33682
0,33573
0,33544
0,33433
0,33429
0,33419
0,33279
0,33208
0,33098
0,33094
0,33016
0,33001
0,32999

0,32981
0,3293
0,32924
0,32906
0,32846
0,32806
0,32719
0,3268
0,32651
0,32585
0,32536
0,32523
0,32523
0,32483
0,32429
0,32406
0,32401
0,32394
0,32381
0,32306
0,32203
0,32072
0,32066
0,32055
0,32013
0,31842
0,31798
0,31788
0,31732
0,3169
0,3169
0,31577
0,31528
0,31463
0,31393
0,31353
0,31333
0,3132
0,31319
0,31314
0,31292
0,31256
0,31222
0,31171
0,311
0,31092
0,31056
0,31022
0,31005
0,30995

0,30949
0,30929
0,30901
0,30775
0,30707
0,30664
0,30585
0,30564
0,30554
0,30519
0,30508
0,30478
0,30476
0,30435
0,30431
0,30388
0,30296
0,30288
0,30154
0,30136
0,30041
0,30025
0,30014
0,29978
0,29891
0,29747
0,29726
0,29724
0,29714
0,29595
0,29407
0,294
0,29327
0,29311
0,29307
0,29215
0,29201
0,29177
0,29122
0,29094
0,29039
0,29027
0,29023
0,2894
0,28924
0,28896
0,28598
0,28543
0,28519
0,28515

0,28495
0,28371
0,2834
0,2831
0,28264
0,28151
0,28146
0,28117
0,28116
0,28034
0,28012
0,27989
0,27965
0,2796
0,2794
0,27928
0,27924
0,27916
0,27845
0,27827
0,27816
0,27788
0,27762
0,27743
0,27735
0,27716
0,27708
0,27706
0,27614
0,27612
0,27597
0,27528
0,27515
0,27509
0,27304
0,27283
0,27282
0,27263
0,272
0,27168
0,27038
0,26984
0,26984
0,26978
0,26812
0,2681
0,26793
0,26788
0,26743
0,26736

0,26725
0,26715
0,26712
0,26681
0,26609
0,26599
0,26568
0,26499
0,26471
0,26466
0,26455
0,26354
0,2627
0,26191
0,26178
0,26176
0,26168
0,2616
0,2607
0,26003
0,25955
0,25872
0,25871
0,25804
0,25754
0,25697
0,25591
0,25535
0,2553
0,25505
0,25448
0,25442
0,25385
0,25379
0,2536
0,25308
0,25286
0,25262
0,25206
0,25184
0,25144
0,25143
0,25085
0,25016
0,24988
0,24951
0,24924
0,24895
0,24883
0,24857

0,24831
0,24773
0,24768
0,2476
0,24734
0,24715
0,24707
0,24681
0,246
0,24573
0,24565
0,24543
0,24525
0,2446
0,24409
0,24401
0,244
0,24349
0,24329
0,24275
0,24249
0,24215
0,24183
0,24159
0,24093
0,24087
0,24042
0,23995
0,23956
0,23945
0,23934
0,23924
0,23912
0,23889
0,23879
0,23878
0,23873
0,23848
0,23741
0,23706
0,23674
0,23409
0,23401
0,23271
0,23253
0,23185
0,23162
0,23158
0,2313
0,23116

0,2311
0,23067
0,23017
0,2301
0,22985
0,22893
0,22852
0,22833
0,2283
0,22829
0,22823
0,22813
0,22744
0,22617
0,22609
0,22574
0,22522
0,22518
0,22507
0,22492
0,22468
0,22425
0,22424
0,22424
0,22355
0,2229
0,22284
0,22215
0,22115
0,22109
0,22073
0,21835
0,21789
0,21764
0,2174
0,21726
0,21712
0,21692
0,21671
0,21659
0,21625
0,21603
0,21588
0,21582
0,21568
0,21564
0,21556
0,21536
0,21482
0,21468

0,21444
0,21436
0,21416
0,21362
0,21346
0,21317
0,21303
0,21293
0,21281
0,21263
0,21252
0,21221
0,21218
0,21202
0,21193
0,21184
0,21172
0,21147
0,2114
0,21118
0,21109
0,21107
0,21079
0,21011
0,2097
0,20926
0,20871
0,20838
0,208
0,20799
0,20794
0,20705
0,20687
0,20686
0,20682
0,20676
0,20654
0,20569
0,20547
0,20547
0,20534
0,20531
0,20523
0,20502
0,20485
0,20381
0,20349
0,20316
0,20274
0,20261

0,20241
0,2023
0,20145
0,20139
0,20124
0,20069
0,20049
0,20042
0,19986
0,19982
0,19973
0,19908
0,19886
0,19838
0,19821
0,19809
0,19785
0,19782
0,19768
0,19766
0,19756
0,19749
0,19745
0,19743
0,19742
0,19736
0,19725
0,19723
0,19697
0,19675
0,1965
0,19642
0,19625
0,19577
0,19554
0,19543
0,19526
0,19523
0,19476
0,19438
0,19412
0,19334
0,193
0,19249
0,19173
0,1911
0,18998
0,18881
0,18879
0,18878

0,1885
0,18818
0,18813
0,18797
0,18751
0,18751
0,187
0,18672
0,18663
0,18658
0,18597
0,18583
0,18568
0,18555
0,18555
0,18503
0,18456
0,18411
0,18372
0,18355
0,1835
0,18301
0,18153
0,18128
0,1808
0,18075
0,18017
0,17982
0,17981
0,17967
0,17931
0,17887
0,17861
0,17829
0,17787
0,17753
0,17714
0,17676
0,17658
0,17648
0,17639
0,17562
0,17537
0,17529
0,17455
0,17448
0,17417
0,17401
0,17398
0,1736

0,17349
0,17272
0,17259
0,1724
0,17204
0,17203
0,17173
0,17144
0,17134
0,17133
0,17065
0,17054
0,17037
0,17016
0,17013
0,17012
0,16971
0,16964
0,16953
0,16947
0,16944
0,16928
0,16911
0,16903
0,1688
0,16855
0,1682
0,168
0,16799
0,16796
0,16787
0,16738
0,16698
0,16676
0,16674
0,16651
0,16646
0,16642
0,16636
0,16616
0,16609
0,16601
0,16596
0,16567
0,1656
0,16553
0,16543
0,16537
0,16499
0,16487

0,1648
0,16435
0,16406
0,16397
0,1639
0,16374
0,1636
0,16321
0,163
0,16275
0,16235
0,162
0,16199
0,16199
0,16154
0,16144
0,16126
0,16122
0,16077
0,15935
0,159
0,15895
0,15888
0,15863
0,15806
0,15798
0,15791
0,15778
0,1574
0,15728
0,15713
0,15694
0,15668
0,1566
0,15588
0,15585
0,15558
0,15554
0,15509
0,15476
0,15464
0,15429
0,15428
0,15427
0,15365
0,15358
0,15351
0,15314
0,1529
0,15258

0,15213
0,15195
0,1517
0,15159
0,15151
0,15093
0,15085
0,15038
0,15019
0,15015
0,14957
0,14956
0,14951
0,14949
0,14872
0,14847
0,14837
0,14828
0,14826
0,14826
0,14789
0,14764
0,14756
0,14747
0,14704
0,147
0,14686
0,14681
0,14672
0,1466
0,14656
0,14648
0,14644
0,14641
0,14627
0,14616
0,14609
0,14594
0,1457
0,14561
0,14555
0,14552
0,1455
0,14523
0,14515
0,14511
0,14504
0,14501
0,14493
0,14478

0,14471
0,14464
0,14455
0,14441
0,14429
0,14376
0,14361
0,14361
0,14342
0,14296
0,14287
0,14285
0,14283
0,14278
0,14276
0,14273
0,14247
0,14234
0,14231
0,14223
0,14221
0,14215
0,14194
0,14176
0,14172
0,14155
0,14147
0,14142
0,14114
0,14095
0,14089
0,14075
0,14071
0,14061
0,14051
0,14013
0,13975
0,13968
0,1395
0,13932
0,1392
0,13918
0,13905
0,13904
0,13901
0,13899
0,13895
0,13888
0,13886
0,13885

0,1388
0,13867
0,13846
0,13838
0,13832
0,13817
0,13803
0,13797
0,13792
0,13783
0,13778
0,13771
0,13745
0,13737
0,13723
0,13688
0,13683
0,13677
0,13675
0,1365
0,13646
0,13618
0,13603
0,13587
0,13586
0,13584
0,13584
0,13499
0,13495
0,13492
0,13472
0,13466
0,13428
0,13413
0,13411
0,13403
0,13398
0,13379
0,13331
0,13295
0,13291
0,13271
0,13263
0,13223
0,13218
0,13197
0,13187
0,13178
0,13173
0,13159

0,13117
0,13092
0,13074
0,13068
0,13067
0,13066
0,13059
0,13018
0,13017
0,1301
0,13004
0,12948
0,12934
0,12901
0,12873
0,12868
0,12866
0,12854
0,12829
0,12805
0,12801
0,12783
0,12744
0,12737
0,12724
0,12705
0,12626
0,12607
0,12571
0,1257
0,12568
0,12543
0,12543
0,1254
0,12492
0,12487
0,12469
0,12466
0,12451
0,12417
0,12383
0,12365
0,12356
0,12335
0,12309
0,12301
0,12292
0,12289
0,12267
0,12266

0,12245
0,12239
0,12206
0,12176
0,1217
0,12154
0,12135
0,12119
0,12071
0,12065
0,12053
0,12041
0,12035
0,12033
0,12005
0,11999
0,11971
0,11966
0,11959
0,11952
0,1194
0,11907
0,11906
0,11891
0,1189
0,11853
0,11834
0,11817
0,118
0,11787
0,11785
0,11781
0,11775
0,11768
0,11764
0,11759
0,11753
0,11749
0,11742
0,11741
0,11733
0,11732
0,11685
0,11684
0,11657
0,11653
0,11648
0,11623
0,11604
0,116

0,11595
0,11578
0,11575
0,11569
0,11554
0,11552
0,11552
0,11549
0,11543
0,11541
0,11538
0,11524
0,11523
0,1152
0,11512
0,11501
0,11461
0,11459
0,11441
0,11432
0,11429
0,11416
0,11405
0,11402
0,11401
0,11363
0,11351
0,11328
0,11301
0,11294
0,11294
0,11271
0,11271
0,11256
0,11253
0,1121
0,1119
0,11161
0,11156
0,11154
0,11148
0,11139
0,1113
0,11118
0,11088
0,11076
0,11014
0,11012
0,11006
0,11004

0,10983
0,10971
0,10969
0,10961
0,10897
0,10882
0,10879
0,10848
0,10844
0,10842
0,10835
0,10827
0,10827
0,10821
0,1078
0,10775
0,10772
0,10755
0,10752
0,10742
0,10738
0,10734
0,10732
0,10723
0,10716
0,1069
0,10666
0,10664
0,10664
0,10664
0,10656
0,10653
0,10652
0,10632
0,10628
0,10626
0,10609
0,10605
0,10601
0,10591
0,10581
0,10575
0,10572
0,10571
0,10562
0,10556
0,10543
0,10537
0,10506
0,10505

0,10504
0,10504
0,10503
0,10502
0,10478
0,10455
0,10444
0,10441
0,10435
0,10428
0,10424
0,10416
0,10413
0,10409
0,104
0,10398
0,10394
0,10393
0,10392
0,10363
0,10342
0,10334
0,10332
0,10323
0,1031
0,1025
0,10238
0,10208
0,10202
0,10199
0,10183
0,10163
0,10158
0,1012
0,10098
0,10091
0,10078
0,10053
0,10019
0,09985
0,0996
0,09954
0,09952
0,0994
0,09923
0,09906
0,09875
0,09849
0,09843
0,09813

0,09809
0,09807
0,09803
0,09799
0,09798
0,09795
0,09786
0,09785
0,09777
0,09768
0,09762
0,09755
0,09744
0,09711
0,09698
0,09698
0,09695
0,0967
0,09656
0,09648
0,09647
0,09632
0,09596
0,09584
0,09558
0,09556
0,09551
0,09547
0,09546
0,09537
0,0949
0,09485
0,09474
0,09471
0,0947
0,09464
0,09456
0,09456
0,09452
0,09445
0,09439
0,09433
0,09424
0,09412
0,09411
0,09401
0,09383
0,09382
0,09369
0,0933

0,09325
0,09306
0,09295
0,0929
0,09282
0,09249
0,09232
0,09216
0,09206
0,09195
0,09172
0,09161
0,09157
0,09138
0,09124
0,09121
0,09115
0,09096
0,0908
0,09069
0,09064
0,09055
0,09046
0,0904
0,0902
0,08979
0,08968
0,08968
0,08965
0,08925
0,08918
0,08894
0,0889
0,08885
0,08882
0,08881
0,08867
0,08855
0,08851
0,08843
0,08842
0,0884
0,08825
0,08822
0,08816
0,08776
0,08773
0,0877
0,08764
0,08757

0,08754
0,08738
0,08731
0,08726
0,08725
0,08702
0,08681
0,08677
0,08633
0,08632
0,08631
0,08614
0,08593
0,08592
0,08589
0,08589
0,08577
0,08571
0,08558
0,08552
0,08548
0,08547
0,08546
0,08544
0,08542
0,08539
0,08526
0,08524
0,08514
0,0851
0,0851
0,08493
0,08491
0,08483
0,08475
0,08465
0,08465
0,08463
0,08462
0,0846
0,08444
0,08442
0,0844
0,0842
0,0839
0,08386
0,08371
0,08361
0,08341
0,08328

0,08324
0,08315
0,08302
0,08302
0,08269
0,08269
0,08267
0,08253
0,08245
0,08245
0,08244
0,08241
0,0823
0,08226
0,08223
0,08212
0,0821
0,08208
0,08191
0,0819
0,08164
0,08161
0,08159
0,08154
0,08144
0,08116
0,08109
0,08088
0,08077
0,08039
0,08035
0,08026
0,08018
0,0801
0,0801
0,08009
0,07993
0,07983
0,07979
0,07976
0,07976
0,07958
0,07957
0,07957
0,07956
0,07951
0,07938
0,07936
0,07927
0,07926

0,07919
0,07906
0,07899
0,07899
0,07894
0,07891
0,07876
0,0787
0,07866
0,07863
0,0786
0,07859
0,07853
0,07847
0,07821
0,07814
0,07801
0,07798
0,07796
0,0779
0,0778
0,07773
0,07766
0,07763
0,07763
0,07762
0,0773
0,07718
0,07717
0,0771
0,07702
0,0769
0,07681
0,07677
0,07665
0,07662
0,07658
0,07647
0,07643
0,07631
0,07619
0,07614
0,0761
0,07605
0,07604
0,07598
0,07585
0,07585
0,07582
0,07568

0,07563
0,07559
0,07549
0,07527
0,07515
0,07515
0,07512
0,0751
0,07502
0,07496
0,07496
0,07466
0,07459
0,07452
0,07448
0,07442
0,07417
0,07413
0,07399
0,07376
0,07375
0,07374
0,0737
0,07365
0,07363
0,07361
0,07359
0,07358
0,07348
0,07337
0,0731
0,07298
0,07297
0,07285
0,07284
0,07276
0,0727
0,07258
0,07249
0,07242
0,07239
0,07232
0,07227
0,07224
0,07223
0,07215
0,07215
0,0721
0,07207
0,07206

0,07204
0,07204
0,07203
0,07195
0,07182
0,0717
0,07159
0,07158
0,0715
0,07146
0,07142
0,0714
0,0713
0,07129
0,07125
0,0712
0,07105
0,07099
0,07095
0,07088
0,07088
0,07079
0,07079
0,07071
0,07063
0,07058
0,07049
0,07036
0,0702
0,07013
0,07009
0,07006
0,06996
0,06992
0,06964
0,06959
0,06956
0,06948
0,06942
0,0694
0,06939
0,06935
0,06933
0,06929
0,06924
0,06912
0,06898
0,06895
0,06891
0,06887

0,06885
0,06885
0,06873
0,0687
0,06868
0,06846
0,06836
0,06834
0,06819
0,06814
0,06812
0,06812
0,06805
0,06804
0,06787
0,06775
0,06774
0,06766
0,06757
0,06752
0,06746
0,06745
0,06737
0,06731
0,06723
0,06718
0,0671
0,06706
0,06704
0,06682
0,0668
0,0667
0,0667
0,06668
0,06664
0,06662
0,0666
0,06654
0,06654
0,06649
0,06648
0,06647
0,06635
0,06633
0,06628
0,06627
0,06625
0,06609
0,06608
0,06599

0,06596
0,06585
0,06581
0,06571
0,0657
0,0656
0,06557
0,06556
0,06554
0,06543
0,0651
0,06504
0,06501
0,06476
0,0647
0,0647
0,06464
0,06441
0,06421
0,06417
0,06403
0,06392
0,06388
0,06382
0,06379
0,06378
0,06374
0,06367
0,0636
0,06347
0,06344
0,06342
0,06337
0,06337
0,06336
0,06335
0,06329
0,06321
0,06311
0,06296
0,06289
0,06287
0,06277
0,06273
0,06271
0,06262
0,06239
0,06237
0,06232
0,06231

0,0623
0,06225
0,06224
0,06222
0,06216
0,06215
0,06212
0,06209
0,06203
0,06193
0,06191
0,0619
0,06182
0,06176
0,06168
0,06153
0,06147
0,06134
0,06133
0,06132
0,06132
0,06122
0,06115
0,06111
0,06105
0,06102
0,06101
0,06089
0,06088
0,06087
0,06079
0,06068
0,06061
0,06058
0,06058
0,06048
0,06027
0,0602
0,0601
0,05997
0,05992
0,05987
0,05985
0,05971
0,05963
0,05961
0,05959
0,05945
0,05944
0,05942

0,05936
0,05932
0,0593
0,05929
0,05928
0,05925
0,05924
0,05921
0,05917
0,05909
0,05908
0,05899
0,05897
0,05888
0,05886
0,05881
0,05864
0,0586
0,05853
0,05848
0,05841
0,05836
0,05834
0,05831
0,05822
0,05817
0,05814
0,05809
0,058
0,05767
0,05762
0,0576
0,0576
0,05759
0,05755
0,0575
0,05745
0,05744
0,05743
0,05742
0,05741
0,0574
0,05739
0,05737
0,05737
0,05717
0,05705
0,05703
0,05702
0,05689

0,05682
0,05682
0,05671
0,05666
0,05666
0,05662
0,05649
0,05648
0,05634
0,05627
0,05626
0,05614
0,05614
0,05608
0,05608
0,05604
0,05601
0,056
0,05598
0,05585
0,05585
0,05582
0,05576
0,0557
0,05565
0,05564
0,05559
0,05546
0,05544
0,05539
0,05538
0,05538
0,05533
0,05519
0,05513
0,05503
0,05502
0,05492
0,0549
0,05487
0,05483
0,05479
0,05478
0,05467
0,0546
0,05459
0,05448
0,05442
0,05432
0,05428

0,05427
0,05416
0,05413
0,05411
0,05409
0,05409
0,05408
0,05404
0,05402
0,054
0,05398
0,05398
0,05394
0,05389
0,05386
0,05382
0,05379
0,05377
0,05361
0,0536
0,05353
0,05336
0,05335
0,0532
0,05319
0,05318
0,05316
0,05311
0,0531
0,05308
0,05304
0,05303
0,05301
0,05292
0,05289
0,05288
0,05286
0,05285
0,05281
0,05278
0,05269
0,05262
0,05257
0,05254
0,05253
0,05251
0,05246
0,05242
0,05237
0,05231

0,0523
0,0523
0,05225
0,05224
0,05214
0,05208
0,05197
0,05194
0,05192
0,05192
0,05184
0,05181
0,05178
0,05177
0,05166
0,05164
0,05162
0,05153
0,05153
0,05153
0,05152
0,05147
0,05145
0,0514
0,05139
0,05139
0,05134
0,05129
0,05117
0,05111
0,05102
0,05097
0,0509
0,05086
0,05085
0,05081
0,05075
0,05074
0,05074
0,05073
0,05064
0,05061
0,05057
0,05053
0,05052
0,05052
0,05043
0,05031
0,05022
0,05011

0,05004
0,04989
0,04976
0,04969
0,04967
0,04964
0,04961
0,04956
0,0495
0,04949
0,04947
0,04939
0,04934
0,04931
0,04915
0,04911
0,04907
0,04899
0,04896
0,04893
0,04891
0,0489
0,04887
0,04878
0,0486
0,04858
0,04858
0,04858
0,04857
0,04856
0,04855
0,04849
0,04845
0,04836
0,04835
0,04834
0,0483
0,04829
0,04828
0,04827
0,04823
0,04822
0,0481
0,04808
0,04806
0,04802
0,04802
0,048
0,04796
0,04795

0,04783
0,04782
0,04776
0,04755
0,04753
0,04742
0,04741
0,0474
0,04737
0,04735
0,04732
0,04726
0,04722
0,04719
0,04717
0,04704
0,04703
0,047
0,04696
0,04689
0,04685
0,04683
0,04683
0,04682
0,04674
0,04671
0,0467
0,04668
0,04668
0,04667
0,04666
0,04652
0,04651
0,0465
0,04641
0,04638
0,04637
0,04637
0,0462
0,04618
0,04617
0,04612
0,04609
0,04607
0,04606
0,04605
0,04601
0,04597
0,04595
0,04595

0,04585
0,04577
0,04576
0,04571
0,04569
0,04562
0,04561
0,0456
0,0456
0,04559
0,04559
0,04541
0,04541
0,0454
0,04539
0,04537
0,04532
0,0453
0,0453
0,04527
0,04527
0,04525
0,04518
0,04515
0,04514
0,04511
0,0451
0,04508
0,04508
0,04503
0,04502
0,04492
0,04486
0,04485
0,04477
0,04476
0,04471
0,04471
0,04469
0,04465
0,0446
0,04457
0,04457
0,04453
0,04449
0,04446
0,04428
0,04428
0,04427
0,04426

0,04425
0,04423
0,04423
0,04413
0,04412
0,04411
0,04409
0,04408
0,04407
0,04405
0,04395
0,04395
0,04388
0,04386
0,04386
0,04385
0,04368
0,04351
0,0435
0,04349
0,04346
0,04342
0,04339
0,04336
0,04332
0,04331
0,04319
0,04314
0,04313
0,04311
0,04311
0,04303
0,04302
0,04294
0,04293
0,0429
0,04288
0,04282
0,04278
0,04269
0,04267
0,04265
0,04265
0,04261
0,04259
0,04256
0,04255
0,04253
0,04247
0,04243

0,04241
0,04239
0,04237
0,04232
0,04232
0,0423
0,04229
0,04223
0,04221
0,0422
0,04206
0,04202
0,04195
0,04194
0,04181
0,0418
0,04176
0,0417
0,04167
0,04166
0,0416
0,04158
0,04152
0,04146
0,04144
0,04134
0,04133
0,04129
0,04121
0,04109
0,04108
0,04107
0,041
0,04094
0,04084
0,04082
0,04082
0,04081
0,04072
0,0407
0,04063
0,04059
0,04054
0,04046
0,04043
0,0404
0,04038
0,04037
0,04034
0,04028

0,04022
0,04019
0,04017
0,04013
0,04012
0,04009
0,04004
0,03999
0,03998
0,03994
0,0399
0,03973
0,0397
0,03963
0,0396
0,03956
0,03955
0,0395
0,03945
0,03945
0,03944
0,03937
0,03919
0,03915
0,03907
0,03893
0,03891
0,03887
0,03883
0,03878
0,03877
0,03875
0,03868
0,03853
0,03848
0,03845
0,03842
0,03816
0,03816
0,03815
0,03813
0,03811
0,03808
0,03807
0,03806
0,03802
0,03802
0,0379
0,03785
0,03784

0,03759
0,03751
0,03746
0,03743
0,03742
0,0374
0,03739
0,03737
0,03731
0,03725
0,03725
0,03715
0,03714
0,0371
0,037
0,03688
0,03682
0,03671
0,03665
0,03653
0,03647
0,03644
0,03637
0,03633
0,03631
0,0363
0,03628
0,03628
0,03622
0,03618
0,03614
0,03612
0,03605
0,03604
0,03598
0,03581
0,03569
0,03569
0,03568
0,03568
0,03563
0,03562
0,03534
0,03529
0,03528
0,03528
0,03527
0,03521
0,0352
0,03515

0,0351
0,03509
0,03497
0,03495
0,03494
0,03494
0,03491
0,0349
0,03488
0,03479
0,03479
0,03474
0,03463
0,03461
0,03458
0,03454
0,03453
0,03444
0,03442
0,0344
0,03438
0,03434
0,03432
0,0343
0,03424
0,03423
0,03423
0,03419
0,03399
0,03393
0,03392
0,03387
0,03383
0,03371
0,03357
0,03353
0,03353
0,03352
0,03352
0,03351
0,0335
0,03349
0,03348
0,03335
0,03327
0,03326
0,03324
0,03324
0,03319
0,03315

0,03306
0,03304
0,03304
0,03303
0,03302
0,03302
0,03301
0,033
0,03288
0,03287
0,03283
0,03275
0,03268
0,03266
0,03265
0,03264
0,03262
0,0325
0,03242
0,03241
0,03232
0,03228
0,03227
0,03225
0,03221
0,03212
0,03205
0,03204
0,03204
0,03203
0,03203
0,03202
0,03199
0,03197
0,03196
0,03188
0,03184
0,03178
0,03178
0,03165
0,03163
0,03162
0,03161
0,03157
0,03153
0,03153
0,03148
0,03134
0,03132
0,0312

0,0311
0,03108
0,031
0,03092
0,03092
0,03091
0,03082
0,03077
0,03075
0,03064
0,0306
0,03058
0,03057
0,03052
0,03039
0,03034
0,03031
0,03026
0,03026
0,03024
0,03023
0,03022
0,03021
0,0302
0,03013
0,03009
0,02989
0,02987
0,02987
0,02983
0,02982
0,0298
0,02979
0,02977
0,02975
0,02968
0,02964
0,02963
0,02961
0,02958
0,02944
0,02932
0,02929
0,02927
0,02925
0,02925
0,02913
0,02911
0,02908
0,029

0,02898
0,02898
0,02895
0,02882
0,0288
0,02866
0,02859
0,02855
0,02852
0,02842
0,02839
0,02829
0,02824
0,02822
0,02816
0,02815
0,02812
0,02811
0,02801
0,028
0,02791
0,0279
0,02778
0,02775
0,0277
0,0276
0,02758
0,02756
0,0275
0,02749
0,02739
0,02737
0,02727
0,02727
0,02726
0,02723
0,02722
0,02715
0,02713
0,02709
0,02708
0,02705
0,02691
0,02691
0,02686
0,02684
0,02681
0,02681
0,02672
0,02672

0,0267
0,02667
0,02667
0,02662
0,02655
0,02651
0,02648
0,02645
0,02629
0,02621
0,02609
0,02609
0,02604
0,02601
0,02595
0,02594
0,02588
0,02575
0,02572
0,02567
0,02547
0,02546
0,02543
0,02543
0,02536
0,02531
0,0253
0,02521
0,0252
0,02518
0,02518
0,02516
0,02515
0,02507
0,02504
0,02503
0,02498
0,02495
0,02487
0,02482
0,02479
0,0247
0,02456
0,02454
0,02451
0,0245
0,02448
0,02448
0,02447
0,02442

0,02441
0,02431
0,02426
0,0242
0,02418
0,02418
0,02417
0,02414
0,02414
0,02409
0,02407
0,02405
0,02402
0,02393
0,02392
0,02391
0,02379
0,02372
0,0237
0,02367
0,02361
0,02345
0,02336
0,02335
0,02327
0,02317
0,02314
0,02309
0,02308
0,02295
0,02292
0,02291
0,02289
0,02284
0,02283
0,02277
0,02276
0,02265
0,02258
0,02258
0,02255
0,02255
0,02248
0,02234
0,02227
0,02226
0,02224
0,02218
0,02211
0,02211

0,02209
0,02207
0,02203
0,02197
0,02187
0,02185
0,02183
0,02183
0,02179
0,02174
0,02164
0,0215
0,02145
0,02144
0,02143
0,0214
0,02136
0,02133
0,02127
0,02122
0,02122
0,0212
0,02109
0,02108
0,02099
0,02093
0,02091
0,02091
0,0209
0,02079
0,02074
0,02072
0,02068
0,02065
0,02063
0,02052
0,02051
0,02051
0,02049
0,02046
0,02044
0,02038
0,02037
0,02035
0,02034
0,02033
0,02024
0,02021
0,02019
0,02016

0,02005
0,02004
0,02
0,01994
0,01982
0,01979
0,01978
0,01976
0,01975
0,0197
0,01968
0,01966
0,01958
0,01953
0,01944
0,01935
0,01925
0,01911
0,01909
0,01908
0,01906
0,019
0,01898
0,01896
0,01893
0,01886
0,01879
0,01879
0,01878
0,01874
0,0187
0,01854
0,01849
0,01847
0,01845
0,01838
0,01837
0,01835
0,01828
0,01826
0,01823
0,01821
0,01819
0,01816
0,01804
0,018
0,01795
0,01778
0,01778
0,01776

0,01774
0,01773
0,01759
0,01752
0,01752
0,01749
0,0174
0,01739
0,01736
0,01731
0,01726
0,01719
0,01714
0,01713
0,01705
0,01694
0,01693
0,01692
0,01691
0,01686
0,01686
0,01684
0,01683
0,01674
0,01671
0,01659
0,01659
0,01656
0,01651
0,01644
0,01641
0,01639
0,01634
0,01628
0,01628
0,01612
0,0161
0,016
0,01596
0,01595
0,01588
0,0158
0,0157
0,01549
0,01545
0,01542
0,01541
0,01534
0,01496
0,01491

0,01487
0,01484
0,01469
0,01467
0,01465
0,01459
0,0145
0,01448
0,01445
0,01445
0,01424
0,01391
0,01384
0,01384
0,01374
0,0137
0,01357
0,01355
0,01355
0,01348
0,01343
0,01337
0,01332
0,01314
0,01313
0,01305
0,01299
0,01299
0,01296
0,01292
0,01291
0,01276
0,01275
0,01275
0,01274
0,01274
0,01269
0,01268
0,01264
0,01258
0,01258
0,01251
0,01242
0,01241
0,01233
0,01222
0,01219
0,0121
0,01205
0,01203

0,0119
0,0118
0,01176
0,01169
0,01165
0,01128
0,0112
0,01113
0,01113
0,01112
0,01111
0,01106
0,01104
0,01104
0,01102
0,01097
0,0108
0,01076
0,01076
0,01073
0,01067
0,01063
0,01051
0,0105
0,01048
0,01045
0,01043
0,01036
0,01034
0,01034
0,01034
0,0103
0,01029
0,01029
0,01025
0,01008
0,01004
0,01003
0,01002
0,00995
0,00994
0,00989
0,00989
0,00981
0,00975
0,00966
0,00958
0,00956
0,00941
0,00939

0,00933
0,00925
0,00924
0,00922
0,00919
0,00911
0,00909
0,00907
0,00898
0,00895
0,00885
0,00883
0,00879
0,00876
0,00872
0,00867
0,00866
0,00864
0,00863
0,00858
0,00857
0,00857
0,00856
0,00846
0,00846
0,0084
0,00836
0,00832
0,00832
0,00829
0,00827
0,00815
0,0081
0,00807
0,00805
0,00801
0,00796
0,00796
0,00789
0,00788
0,00781
0,00779
0,00777
0,00772
0,0076
0,00752
0,00739
0,00728
0,00727
0,00723

0,00711
0,00707
0,00707
0,00705
0,00702
0,00701
0,00698
0,00687
0,00685
0,00685
0,00679
0,00671
0,00666
0,00664
0,00654
0,00652
0,00649
0,0064
0,00625
0,00614
0,00603
0,00586
0,00579
0,00576
0,00571
0,00565
0,00561
0,00542
0,00539
0,00527
0,00524
0,00517
0,0051
0,00508
0,00501
0,005
0,00497
0,00491
0,00485
0,00482
0,00473
0,00471
0,00462
0,00454
0,0045
0,00447
0,00442
0,00437
0,00435
0,0043

0,00427
0,00412
0,00409
0,00407
0,00396
0,00365
0,00351
0,00345
0,0034
0,00339
0,00327
0,0032
0,00313
0,00291
0,00282
0,00281
0,00274
0,00266
0,00254
0,00254
0,00249
0,0022
0,00216
0,00215
0,00214
0,00212
0,00206
0,00204
0,00178
0,0015
0,00129
